# Pitavastatin protects against neomycin-induced ototoxicity through inhibition of endoplasmic reticulum stress

**DOI:** 10.3389/fnmol.2022.963083

**Published:** 2022-08-03

**Authors:** Yunhao Wu, Wei Meng, Ming Guan, Xiaolong Zhao, Chen Zhang, Qiaojun Fang, Yuhua Zhang, Zihui Sun, Mingjing Cai, Dongdong Huang, Xuechun Yang, Yafeng Yu, Yong Cui, Shuangba He, Renjie Chai

**Affiliations:** ^1^State Key Laboratory of Bioelectronics, Jiangsu Province High-Tech Key Laboratory for Bio-Medical Research, Department of Otolaryngology Head and Neck Surgery, School of Life Sciences and Technology, Zhongda Hospital, Advanced Institute for Life and Health, Southeast University, Nanjing, China; ^2^Department of Otorhinolaryngology Head and Neck Surgery, School of Medicine, Nanjing Tongren Hospital, Southeast University, Nanjing, China; ^3^Department of Otolaryngology, Affiliated Hangzhou First People’s Hospital, Zhejiang University School of Medicine, Hangzhou, China; ^4^Department of Otolaryngology Head and Neck Surgery, Sichuan Provincial People’s Hospital, University of Electronic Science and Technology of China, Chengdu, China; ^5^Beijing Key Laboratory of Neural Regeneration and Repair, Department of Neurobiology, Advanced Innovation Center for Human Brain Protection, School of Basic Medical Sciences, Capital Medical University, Beijing, China; ^6^Department of Otolaryngology-Head and Neck Surgery, Guangdong Provincial People’s Hospital, Guangdong Academy of Medical Sciences, Guangzhou, China; ^7^Department of Otolaryngology, First Affiliated Hospital of Soochow University, Suzhou, China; ^8^The Second School of Clinical Medicine, South Medical University, Guangzhou, China; ^9^School of Medicine, South China University of Technology, Guangzhou, China; ^10^Co-Innovation Center of Neuroregeneration, Nantong University, Nantong, China; ^11^Institute for Stem Cell and Regeneration, Chinese Academy of Sciences, Beijing, China; ^12^Beijing Key Laboratory of Neural Regeneration and Repair, Capital Medical University, Beijing, China

**Keywords:** pitavastatin, neomycin, hearing loss, hair cell, endoplasmic reticulum stress

## Abstract

Irreversible injury to inner ear hair cells induced by aminoglycoside antibiotics contributes to the formation of sensorineural hearing loss. Pitavastatin (PTV), a 3-hydroxy-3-methylglutaryl coenzyme A reductase inhibitor, has been reported to exert neuroprotective effects. However, its role in aminoglycoside-induced hearing loss remains unknown. The objectives of this study were to investigate the beneficial effects, as well as the mechanism of action of PTV against neomycin-induced ototoxicity. We found that PTV remarkably reduced hair cell loss in mouse cochlear explants and promoted auditory HEI-OC1 cells survival after neomycin stimulation. We also observed that the auditory brainstem response threshold that was increased by neomycin was significantly reduced by pretreatment with PTV in mice. Furthermore, neomycin-induced endoplasmic reticulum stress in hair cells was attenuated by PTV treatment through inhibition of PERK/eIF2α/ATF4 signaling. Additionally, we found that PTV suppressed the RhoA/ROCK/JNK signal pathway, which was activated by neomycin stimulation in HEI-OC1 cells. Collectively, our results showed that PTV might serve as a promising therapeutic agent against aminoglycoside-induced ototoxicity.

## Introduction

Aminoglycosides are extensively used for serious infections in clinical therapeutics, but the side effect of permanent hearing impairment limits their application. It has been reported that sensory hair cells in the inner ear are the main targets of aminoglycosides (Zhanel et al., 2012). Aminoglycosides enter inner ear compartments which are filled with endolymph by passing through the blood–labyrinth barrier, and they enter hair cells *via* interacting with several cation channels, resulting in the reactive oxygen species accumulation and cell apoptosis (Alharazneh et al., 2011; Stepanyan et al., 2011; Kros and Steyger, 2019). Although in-depth studies have been performed to elucidate the mechanisms responsible for aminoglycoside-induced ototoxicity, most therapeutic strategies to improve outcomes have been frustrated up to now.

The endoplasmic reticulum (ER), which is an intracellular vesicle-like structure that participates in the process of protein folding, plays a critical role in maintaining normal cellular function and homeostasis (Wang and Kaufman, 2016). ER stress occurs when unfolded or misfolded proteins accumulate, leading to an impairment of ER function and perturbation of ER homeostasis (Fernández et al., 2015; Oakes and Papa, 2015; So, 2018). Prolonged ER stress responses can trigger cellular apoptosis and thus play a pivotal role in the pathological process of numerous diseases, including cardiovascular diseases, neurodegenerative diseases, cancer, and metabolic diseases (Urra et al., 2016; Brown et al., 2020; Ghemrawi and Khair, 2020; Radwan et al., 2020). The relationship between ER stress and hearing loss has been investigated for several decades, and aminoglycoside antibiotics have been demonstrated for the induction of hair cell apoptosis accompanied by ER stress. Also, ER stress inhibition exhibits attenuation for aminoglycoside-induced cochlear hair cell death (Jia et al., 2018), thus highlighting the need for further investigation on the relationship between ER stress and aminoglycoside-induced ototoxicity.

Statins that act as antilipemic agents by preventing cholesterol biosynthesis are the inhibitors of 3-hydroxy-3-methylglutaryl coenzyme A (HMG-CoA) reductase. Statins are not only applied for the therapeutics of cardiovascular diseases, but also for the treatment of neurological disorders (Oesterle et al., 2017; Sanz-Cuesta and Saver, 2021), and lots of studies have suggested that statins have potential protective effects against sensorineural hearing loss (Brand et al., 2011; Park et al., 2012; Fernandez et al., 2020; Whitlon, 2022). Pitavastatin (PTV) is a new-generation lipophilic statin that has been reported to exert anti-oxidative, anti-inflammatory, anti-neoplastic, and neuroprotective effects (Kaneyuki et al., 2007; Gbelcová et al., 2017; Cui et al., 2018; Li and Liu, 2022), but the role of PTV in aminoglycoside-induced ototoxicity remains unknown.

In the current research, we studied the protective properties and potential mechanisms of PTV on neomycin-triggered hearing loss by constructing *in vivo* and *in vitro* models. The ultimate goal was to assist in the discovery and development of therapeutic drugs for preventing aminoglycoside-triggered sensorineural hearing loss.

## Materials and methods

### Cell viability assay

HEI-OC1 cells were inoculated into 96-well plates (2 × 10^5^ cells/ml) overnight. Then, the cells were incubated with PTV at different concentrations (0.001, 0.005, 0.01, 0.05, 0.1, and 0.5 μm) for 24 h and challenged with neomycin for the next 24 h. Then, CCK-8 solution (1:10 dilution in DMEM) was given to the cells for 30-min incubation at 37°C. The absorbance value was measured by a Thermo Scientific microplate reader at 450 nm.

### *In vivo* experiments

SPF C57BL/6 mice were purchased from Gempharmatech Co., Ltd. (Nanjing, China). After being allowed to acclimate for 3 days, mice of P28 were intraperitoneal injected PTV (Aladdin, P129617) with a dose of 3 mg/kg. After 2 h, neomycin (Sigma, N6386) at 100 mg/kg was injected intraperitoneally, and then after 30 min, mice were given a single dose of furosemide (Sigma, BP547) at 200 mg/kg by intraperitoneal injection. The measurement of auditory brainstem response (ABR) and counting of cochlear hair cells were performed 2 days later.

### Whole-organ explants culture

The cochleae of P3 wild-type mice were dissected from the inner ear and immersed in bioclean HBSS (Multicell, 311512011) using a stereo microscope. The spiral ganglion, spiral ligament, and stria vascularis of cochleae were removed, and the cochlear basilar membranes were put in dishes that were smeared with Corning^®^ Cell-Tak™ and then cultured with DMEM-F12 medium added with ampicillin (Beyotime, ST008), N2 Supplement (Stemcell, 07152), and SM1 neuronal supplement (Stemcell, 05711) for 12 h in an incubator at 37°C, 5% CO_2_. About 0.01 μm PTV was given to the samples for 12 h, and then, 0.5 mm neomycin with 0.01 μm PTV was given together to the cochleae for another 12 h.

### Auditory brainstem response audiometry

Hearing thresholds of mice were assessed by ABR. ABR experiment was carried out in an acoustic space, and the variation in brain electrical activity in mice in answer to different sounds was recorded by electrodes. After being anesthetized, the mice were kept on a preheating pad (37°C), and the ABR responses were recorded at different frequencies on a Tucker-Davis Technology System III system (Tucker Davies Technologies, Gainesville, FL, United States).

### Immunofluorescence

The tissue or cell samples fixed with 4% paraformaldehyde were permeabilized with 1% Triton X-100. After blocking with QuickBlock™ buffer (Beyotime, P0260) for 1 h, and the primary antibodies against myosin 7a (Abcam, ab150386, 1:200 dilution), cleaved caspase-3 (Abcam, ab32042, 1:150 dilution), GRP78 (Proteintech, 11587-1-AP, 1:200 dilution), and CHOP (Proteintech, 15204-1-AP, 1:200 dilution) were added to the samples at 4°C overnight. Next day after washing three times with phosphate-buffered saline (PBS), the samples were incubated with corresponding secondary antibodies for 1 h at room temperature then washed three times with PBS and incubated with ECL solution and imaged using confocal laser scanning microscope (Zeiss, Germany).

### Flow cytometry

The effect of PTV on neomycin-triggered apoptosis of HEI-OC1 cells was evaluated by an Annexin-V/PI kit (BD, 556419). Briefly, cells were harvested by digestion and centrifugation. Precooling PBS was used to wash the cells three times, and after being suspended by the binding buffer, cells were incubated with 5 μl Annexin V-FITC and 5 μl PI for 20 min at room temperature. The apoptosis was subsequently assayed by flow cytometry (MACSQuant, Germany).

### Terminal deoxynucleotidyl transferase dUTP nick end labeling staining

A terminal deoxynucleotidyl transferase dUTP nick end labeling (TUNEL) BrightGreen apoptosis detection kit (Vazyme, A112-01) was used to determine the apoptosis of HEI-OC1 cells and cochlear explants. Briefly, the samples were fixed and permeabilized, then equilibrated with 1 × Equilibration Buffer for 30 min at room temperature. After incubating with the label solution at 37°C for 1 h, the samples were washed by PBS for 2 times and imaged by a confocal laser scanning microscope.

### Western blot

The total protein of cell samples was extracted using RIPA lysis buffer (Beyotime, P0013C) containing phenylmethanesulfonyl fluoride (1 mm). After quantification by a BCA kit (Beyotime, P0012S), the sodium dodecyl sulfate–polyacrylamide gel electrophoresis (SDS-PAGE) sample loading buffer (Beyotime, P0015) was added to the total protein lysate, and the mixed buffer was boiled at 100°C for 5 min. Total proteins (40 μg) of each group were added to the gels and separated through electrophoresis and then transferred to polyvinylidene fluoride (PVDF) membranes. After being blocked in 5% BSA-PBST for 2 h, the immunoblots were immersed in 5% BSA-PBST, which contained primary antibodies overnight at 4°C. Next day, the bands were washed three times with 0.05% tween-PBS (PBST) and then combined with the corresponding HRP conjugated secondary antibodies and detected by an ECL kit (Vazyme, E411-04) and analyzed by ImageJ software.

### Statistical analysis

All data are analyzed by GraphPad Prism 9 software and are presented as the mean ± standard deviation (SD). Statistical significance was calculated with one-way analysis of variance (ANOVA) followed by Dunnett’s test when comparing more than two groups. A *p*-value < 0.05 indicated a statistically significant difference.

## Results

### Pitavastatin protects against neomycin-induced hair cell damage

**FIGURE 1:**
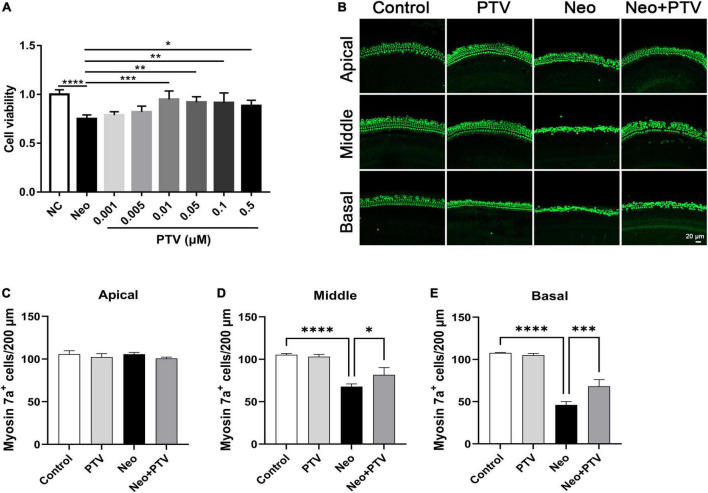
PTV attenuates neomycin-induced HEI-OC1 cell damage and missing of hair cells in cochlear explants *in vitro*. **(A)** The CCK8 method was used to determine the protective effect of PTV (0.001, 0.005, 0.01, 0.05, 0.1, and 0.5 μm) on neomycin-induced HEI-OC1 cell injury (*n* = 6). **(B)** Immunostaining of hair cells in cochlear explants in the apical, middle, and basal turns with anti-myosin 7a antibody. Scale bars = 20 μm. **(C–E)** Counting of the amount of hair cells per 200 μm in inner ear in the apical, middle and basal turns (*n* = 3). *p < 0.05, **p < 0.01, ***p < 0.001, ****p < 0.0001.

To confirm whether PTV has a beneficial effect on neomycin-triggered hair cell injury, auditory HEI-OC1 cells were administrated with different doses of PTV (0.001, 0.005, 0.01, 0.05, 0.1, and 0.5 μm) prior to neomycin (2 mm) treatment. PTV showed significant attenuation of cell injury triggered by neomycin at doses of 0.01–0.5 μm, and PTV at 0.01 μm exhibited the best protective effect, so premedication of 0.01 μm PTV for 24 h was chosen as the best administration scheme (Figure 1A). We also measured the influence of PTV on whole-organ cochlear explant cultures from P3 mice after neomycin treatment. Immunostaining results indicated that neomycin treatment resulted in an obvious missing of hair cells in cochleae in the middle and basal turns and that PTV pretreatment distinctly prevented neomycin-stimulated inner ear hair cell loss (Figures 1B–E). The above results disclose that PTV has effective protection against neomycin-triggered hair cell injury.

### Pitavastatin alleviates neomycin-triggered hearing loss *in vivo*

**FIGURE 2:**
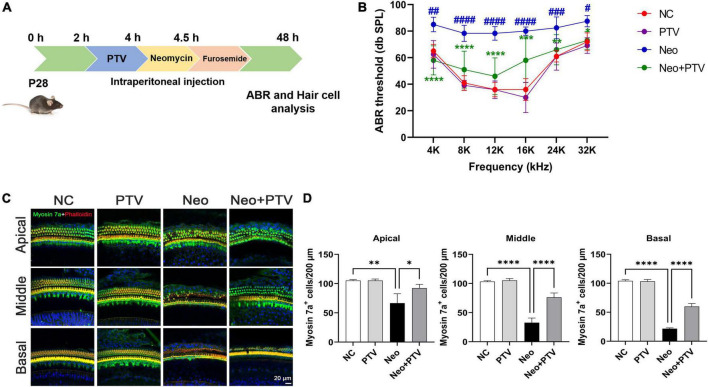
PTV promotes auditory function recovery and prevents neomycin-triggered hair cell loss *in vivo*. **(A)** Procedure of the experiments *in vivo*. **(B)** Auditory function of mice was detected using ABR method (*n* = 6). **(C)** Immunofluorescence of mouse cochlear hair cells stained with myosin 7a and phalloidin in the apical, middle, and basal turns. Scale bars = 20 μm. **(D)** Counting of the amount of cochlear hair cells per 200 μm in mice (*n* = 3). ^#^*p* < 0.05, ^##^*p* < 0.01, ^###^*p* < 0.001, ^####^*p* < 0.0001 vs. NC group; *p < 0.05, **p < 0.01, ****p < 0.0001 vs. Neo group.

Next, we investigated the protective property of PTV on neomycin-triggered hearing loss by establishing an acute neomycin-induced ototoxicity model according to the previous study (He et al., 2020). C57BL/6 mice (P28) were intraperitoneally injected with 3 mg/kg PTV, and after 2 h, mice were given an intraperitoneal injection of 100 mg/kg neomycin in conjunction with 200 mg/kg furosemide (Figure 2A). ABR measurements were employed to determine the auditory function of mice, and the results suggested that neomycin treatment led to an obvious elevation of ABR thresholds, whereas PTV administration strongly attenuated this effect (Figure 2B). By counting cochlear hair cells, we found a massive loss of cochlear hair cells in the neomycin plus furosemide group, whereas PTV had an apparent promotion for hair cell survival (Figures 2C,D). Collectively, the data reveal that PTV attenuates neomycin-triggered hearing loss *in vivo*.

### Pitavastatin attenuates neomycin-triggered apoptosis in HEI-OC1 cells

**FIGURE 3:**
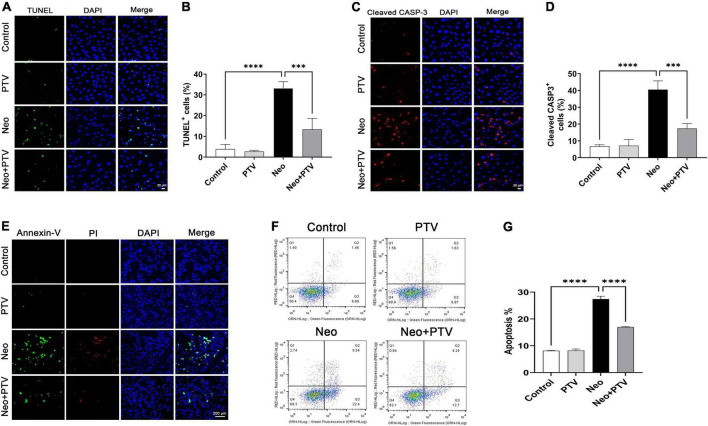
Effects of PTV on neomycin-triggered HEI-OC1 cell apoptosis. **(A,C)** TUNEL staining and cleaved CASP-3 immunostaining of HEI-OC1 cells. Scale bars = 20 μm. **(B,D)** Statistics of the proportions of TUNEL or cleaved CASP-3 highlighted HEI-OC1 cells in **(A,C)** (*n* = 3). **(E)** Annexin-V/PI staining in PTV and neomycin treatment HEI-OC1 cells. Scale bars = 200 μm. **(F)** Apoptosis detection of HEI-OC1 cells by flow cytometry. **(G)** Statistics of the proportions of apoptotic HEI-OC1 cells in different groups (*n* = 3). ^***^p < 0.001, ^****^p < 0.0001.

To explore the protective effects of PTV against neomycin-induced HEI-OC1 cell damage, TUNEL and cleaved caspase-3 (cleaved CASP-3) dying were conducted to detect apoptosis. We observed that neomycin stimulation for 24 h dramatically increased the count of TUNEL and cleaved CASP-3-positive cells, whereas PTV group exhibited an effective improvement (Figures 3A–D). Annexin V-FITC/PI kit was employed to further confirm the benefit of PTV on the apoptosis triggered by neomycin, and immunostaining and flow-cytometric assays exhibited that neomycin significantly induced cell apoptosis, whereas PTV administration showed a notable attenuation in the apoptosis of HEI-OC1 cells (Figures 3E–G). Our results indicate that PTV attenuates HEI-OC1 cell apoptosis induced by neomycin.

### Pitavastatin reduces apoptosis of cochlear hair cells after neomycin treatment

**FIGURE 4:**
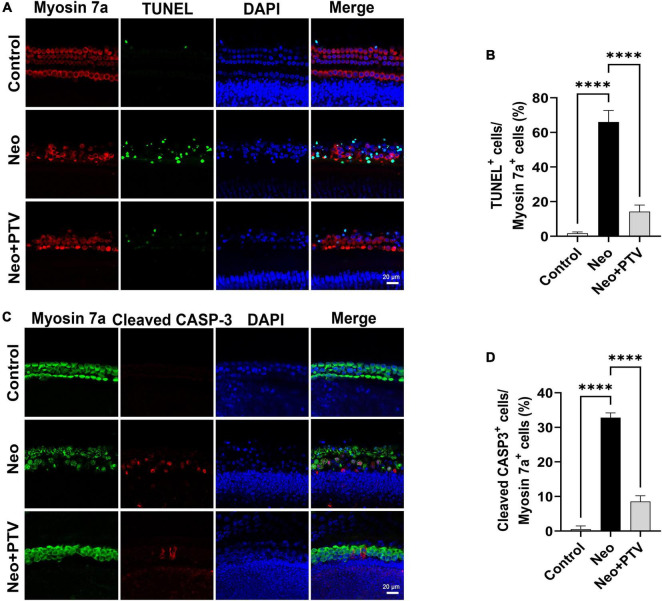
Effects of PTV on apoptosis of cochlear hair cell in whole-organ explants induced by neomycin. **(A)** Immunostaining with TUNEL and myosin 7a labeling in the middle turns in cochlear explants. Scale bars = 20 μm. **(B)** Statistics of the proportions of TUNEL highlighted hair cells in **(A)**. **(C)** Immunostaining with cleaved CASP-3 and myosin 7a in the middle turns in cochlear explants. Scale bars = 20 μm. **(D)** Statistics of the proportions of cleaved CASP-3 highlighted hair cells in **(C)**. ^****^p < 0.0001.

We further explored the impact of PTV on neomycin-triggered apoptosis of hair cells in cochlear explant cultures. After being dissected from P3 mice, the cochlear explants were cultured at 37°C and 5% CO_2_ for 12 h and then pretreated with 0.01 μm PTV for 12 h followed by neomycin (0.5 mm) treatment together with PTV for another 12 h. Using TUNEL and cleaved CASP-3 staining, we found that the amount of TUNEL-positive hair cells that were marked by myosin 7a and the amount of cleaved CASP-3 staining hair cells in model group were prominently higher than the control group, whereas PTV administration clearly decreased the amount of apoptotic hair cells, which is in accordance with the results discussed above in HEI-OC1 cells (Figures 4A–D). Collectively, the data demonstrate that PTV suppresses the apoptotic cascade under aminoglycosides stimulation.

### Pitavastatin inhibits neomycin-induced endoplasmic reticulum stress in cochlear hair cells

**FIGURE 5:**
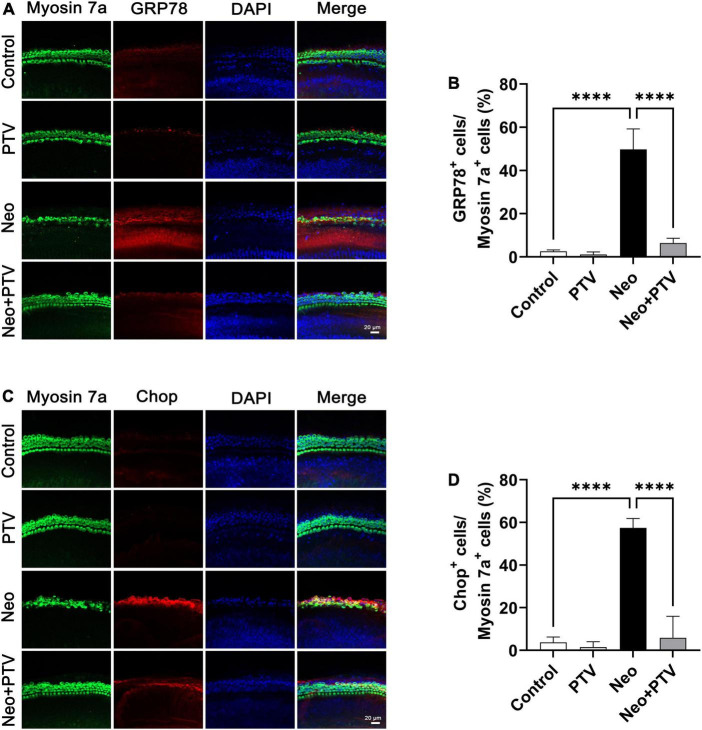
Impacts of PTV on neomycin-triggered ER stress in cochlear hair cells. **(A)** Immunostaining with myosin 7a and GRP78 in middle turns in cochlear explants. Scale bars = 20 μm. **(B)** Statistics of the proportions of GRP78 highlighted hair cells in **(A)**. **(C)** Immunostaining with myosin 7a and CHOP in middle turns in cochlear explants. Scale bars = 20 μm. **(D)** Statistics of the proportions of CHOP highlighted hair cells in **(C)**. ^****^p < 0.0001.

It has been reported that ER stress plays a pivotal role in aminoglycoside-induced hair cell apoptosis (Jia et al., 2018), so we next determined the expression of ER stress-relevant proteins GRP78 and CHOP after neomycin stimulation. We discovered that the level of GRP78 together with CHOP was remarkably higher than the control group after neomycin treatment. PTV administration could inhibit ER stress by suppressing the elevated expression of GRP78 and CHOP induced by neomycin (Figures 5A–D), and this further indicates that PTV can ameliorate neomycin-induced ER stress.

### Pitavastatin suppresses neomycin-induced endoplasmic reticulum stress by inhibiting PERK/eIF2α/ATF4 signaling in HEI-OC1 cells

**FIGURE 6:**
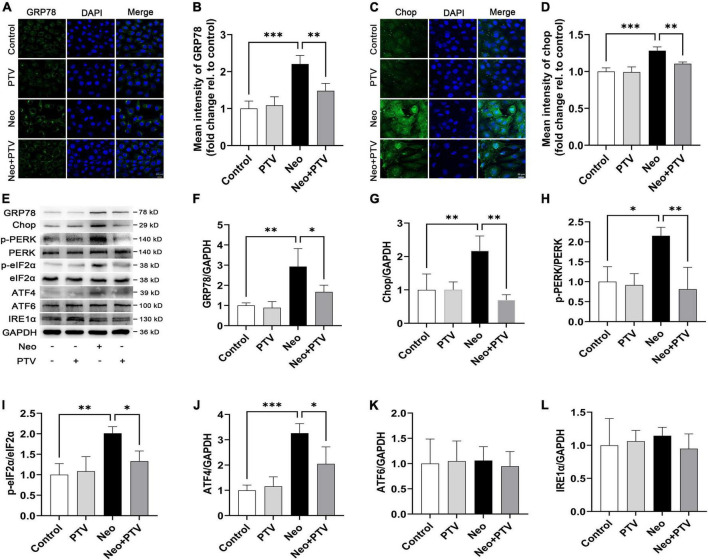
PTV alleviates neomycin-induced ER stress by inhibiting PERK/eIF2α/ATF4 signaling. **(A,C)** Immunofluorescence of HEI-OC1 cells with anti-GRP78 and anti-CHOP antibodies. Scale bars = 20 μm. **(B,D)** Statistics of the mean intensity of GRP78 and CHOP in HEI-OC1 cells in **(A,C)**. **(E)** Western blot analysis of GRP78, CHOP, p-PERK, PERK, p-eIF2α, eIF2α, ATF4, ATF6, IRE1α, and GAPDH in HEI-OC1 cells with PTV pretreatment followed by neomycin exposure. **(F–L)** Quantification of the protein expression in **(E)** with ImageJ (*n* = 3). *p < 0.05, ^**^p < 0.01, ^***^p < 0.001.

To explore the molecular mechanism by which PTV inhibits neomycin-triggered ER stress, we examined the three classical signal pathways (PERK signaling, IRE1α signaling, and ATF6 signaling) that are involved in ER stress. First, by immunostaining, we confirmed that PTV could prevent the high expression of GRP78 and CHOP induced by neomycin in HEI-OC1 cells (Figures 6A–D). Next, western blot analysis revealed that the expression levels of p-PERK, p-eIF2α, ATF4, GRP78, and CHOP were markedly increased after neomycin stimulation in cells, which was strongly inhibited by PTV treatment (Figures 6E–J). Also, there was no obvious change in IRE1α or ATF6 expression after PTV and neomycin treatment (Figures 6K,L). Together, the results above suggest that PTV attenuates neomycin-induced ER stress mainly by restricting PERK/eIF2α/ATF4 signaling in HEI-OC1 cells.

### Pitavastatin significantly inhibits the RhoA/ROCK signaling pathway activated by neomycin

**FIGURE 7:**
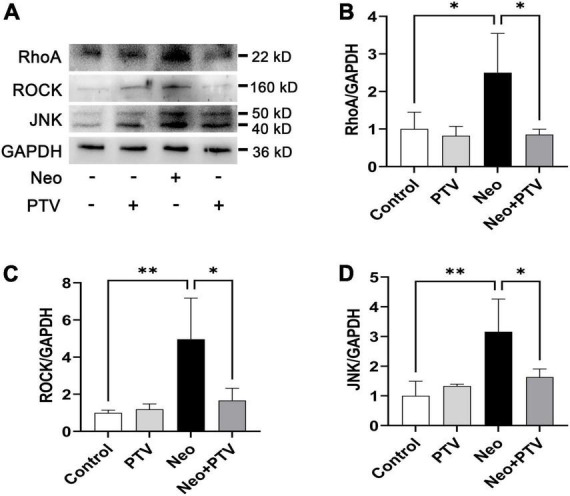
PTV inhibits neomycin-triggered activation of the Rho/ROCK signaling pathway. **(A)** Western blot analysis of RhoA, ROCK, JNK, and GAPDH in HEI-OC1 cells with PTV pretreatment followed by neomycin exposure. **(B–D)** Quantification of the protein expression in **(A)** with ImageJ (*n* = 3). *p < 0.05, ^**^p < 0.01.

Previous research has shown that PTV exerts its neuroprotective effects mainly through the inhibition of Rho/ROCK signaling pathway (Hamano et al., 2012). PTV is a competitive inhibitor of the HMG-CoA reductases that activate the Rho/ROCK signaling pathway, but whether PTV protects against neomycin-induced ototoxicity through the inhibition of the Rho/ROCK signaling pathway remains unclear. In this study, we also tested the change of Rho/ROCK signaling after PTV administration and neomycin treatment in HEI-OC1 cells. Western blot results showed that the expression levels of the key factors in Rho signaling, including RhoA, ROCK, and JNK, were prominently upregulated after neomycin stimulation, whereas PTV pretreatment strongly downregulated the elevated expression of RhoA, ROCK, and JNK induced by neomycin (Figures 7A–D). These data suggest that PTV protects against neomycin-induced ototoxicity by inhibiting the Rho/ROCK signaling pathway.

## Discussion

Aminoglycoside antibiotics are used to treat gram-negative bacterial infections, but their application is restricted by the severe side effects of ototoxicity and vestibular toxicity. Aminoglycosides can accumulate in cochlear hair cells and are hard to metabolize, which may lead to irreversible damage of cochlear hair cells and result in permanent hearing loss. In the present study, we show that the HMG-CoA reductase inhibitor PTV could efficiently attenuate neomycin-triggered ototoxicity. By *in vitro* and *in vivo* studies, we demonstrated that PTV protected against neomycin-induced apoptosis of cochlear hair cells, and the protective effect might be in connection with the inhibition of ER stress. We further confirmed that PTV exerted anti-apoptotic effects and suppressed PERK/eIF2α/ATF4 signaling-mediated ER stress by inhibiting Rho/ROCK signaling.

PTV is a novel synthetic lipophilic statin with greater safety, tolerability, and fewer adverse effects compared with conventional statins and is commonly used for the treatment of hypercholesteremia (Hoy, 2017; Chan et al., 2019; Adams et al., 2020). Previous research showed that PTV exhibited efficient neuroprotective effects independent of its antilipemic effect (Kozuki et al., 2011; Kurata et al., 2011a,b). However, there is no investigation in regard to the beneficial effect of PTV on aminoglycoside-triggered hearing loss. In this study, by establishing an acute neomycin-induced ototoxicity model, we observed that PTV could effectively mitigate neomycin-triggered hearing loss *in vivo* (Figure 2). We also found that PTV protected against auditory HEI-OC1 cell and cochlear explant injury triggered by neomycin *in vitro* (Figure 1). These results suggested that PTV might be a promising agent for the prevention of aminoglycoside-triggered ototoxicity.

Apoptosis of hair cells leading to hearing loss is the key factor in the “ototoxicity” of aminoglycosides (Wu et al., 2021). Using TUNEL staining, we found that the proportions of TUNEL/myosin 7a double-positive cells in cochlear explants were remarkably increased after neomycin treatment, confirming that neomycin may cause cochlear hair cell death through apoptosis. In addition, there was very less TUNEL staining observed in the PTV pretreatment group (Figures 4A,B). Cleaved CASP-3 is considered a universal marker of apoptosis due to its critical role in the pathogenesis of cell apoptosis (Wang et al., 2021). In our study, we also found that PTV could significantly reduce the increased numbers and proportions of apoptotic hair cells induced by neomycin (Figures 4C,D), which is consistent with our *in vitro* results in HEI-OC1 cells (Figure 3). Collectively, these results demonstrate that PTV exerts its beneficial effect on neomycin-triggered hair cell injury by inhibiting the occurrence of apoptosis.

It is well documented that ER stress is triggered by the disruption of homeostasis in the ER and results in the activation of the unfolded protein response (UPR) (Lu et al., 2014; Fu et al., 2021). When the unfolded or misfolded proteins in the ER accumulate and exceed a tolerable threshold, the function of ER may be lost and be difficult to restore leading to cellular dysfunction and apoptosis (Gorman et al., 2012; Verfaillie et al., 2013; Hu et al., 2019). There are three classical signal pathways involved in UPR, including PERK signaling, IRE1 signaling, and transcription factor ATF6 signaling (Walter and Ron, 2011). GRP78, which is a type of peptide-binding protein that prevents the aggravation of protein folding, has been reported to be a key regulator of ER stress and UPR activation (Adams et al., 2019). Changes in the microenvironment due to physiological processes or to pathological conditions, such as hypoxia, viral, or bacterial infections and drugs, can induce ER stress, thus causing GRP78 to separate from the sensors (PERK, IRE1, and ATF6) and further activate downstream signaling (Hollien and Weissman, 2006; Díaz-Bulnes et al., 2020; Kapadia et al., 2021; Mazel-Sanchez et al., 2021). It was observed that neomycin treatment significantly increased the expression levels of GRP78 and CHOP in cochlear hair cells, which indicated that neomycin could activate ER stress (Figure 5). We also found that PTV pretreatment effectively alleviated neomycin-induced ER stress through inhibition of the PERK/eIF2α/ATF4 signaling pathway (Figure 6). These results manifest that PTV is able to suppress neomycin-induced hair cell apoptosis by inhibiting ER stress *via* mediation of the PERK/eIF2α/ATF4 signaling pathway.

As an HMG-CoA reductase inhibitor, PTV has been reported to exert its neuroprotective effect *via* the inhibition of Rho/ROCK signaling (Hamano et al., 2012, 2020). It has also been reported that ROCK inhibitor has neuroprotective and regenerative effects on synaptic pathways by promoting synapse formation in cochlear hair cells (Koizumi et al., 2020). By western blot assays, we confirmed that PTV could decrease the elevated expression of RhoA, ROCK, and JNK induced by neomycin (Figure 7). Thus, PTV appears to attenuate neomycin-induced ototoxicity by inactivation of Rho/ROCK signaling.

## Conclusion

In summary, we show that PTV can ameliorate neomycin-triggered cochlear hair cell injury and hearing loss by inhibiting RhoA/ROCK signaling and can suppress ER stress by blocking the PERK/eIF2α/ATF4 pathway. The findings indicate that PTV may serve as a promising agent for the prevention of aminoglycoside-induced ototoxicity.

## Data availability statement

The original contributions presented in the study are included in the article/Supplementary material, further inquiries can be directed to the corresponding author/s.

## Ethics statement

The animal study was reviewed and approved by the Animal Care and Use Committee of Southeast University.

## Author contributions

YW, RC, SH, and XZ designed the whole experiments. YW, YY, WM, and MG conducted the most part of the experiments and analyzed the data. CZ, QF, and YZ conducted part of the *in vitro* experiments. DH, MC, and ZS conducted part of the *in vivo* experiments. XY and YC drafted the manuscript. All authors contributed to the article and approved the submitted version.
