# Supplementary material for: Pitavastatin protects against neomycin-induced ototoxicity through inhibition of endoplasmic reticulum stress
Source: Front Mol Neurosci. 2022 Aug 3;15:963083. doi: 10.3389/fnmol.2022.963083 (PMC9381809; doi:10.3389/fnmol.2022.963083)

Supplementary Material

## Supplementary Figures

Repeat 1 Repeat 2 Repeat 3

GRP78
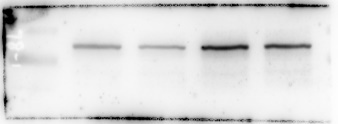

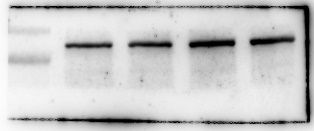

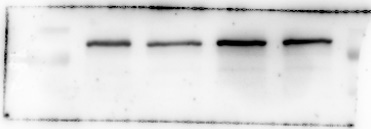


Chop
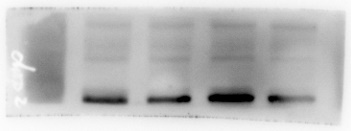

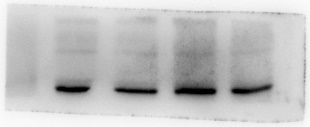

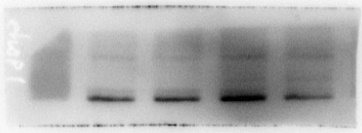


p-PERK
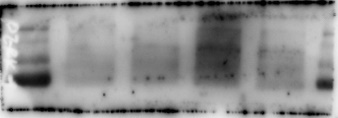

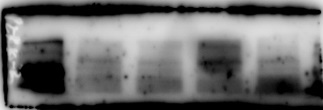

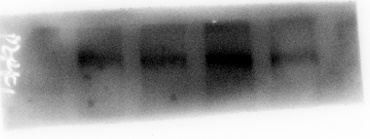


PERK
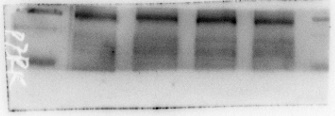

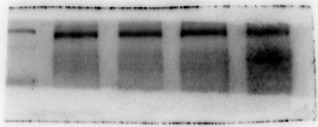

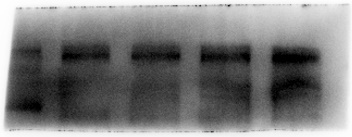


p-eIF2α
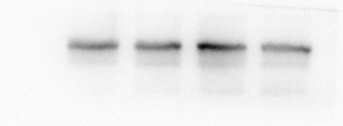






eIF2α
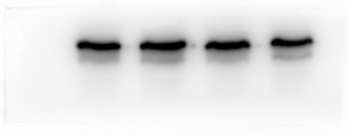

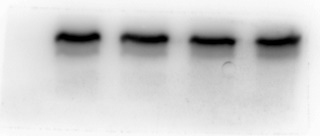

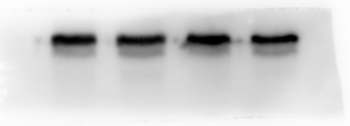


ATF4
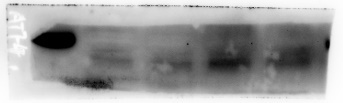

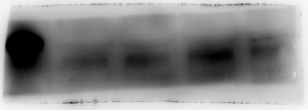

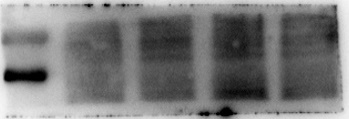


ATF6
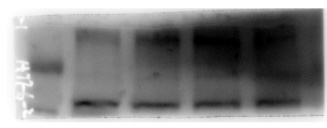

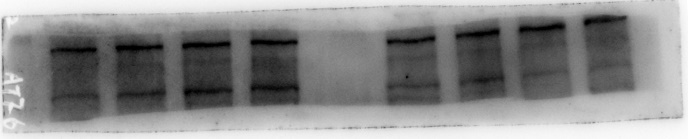


IRE1α
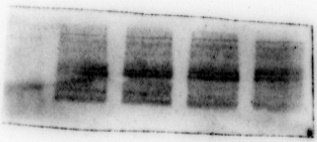

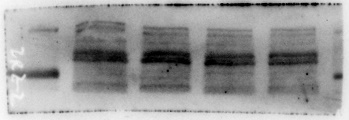

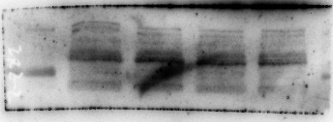


GAPDH




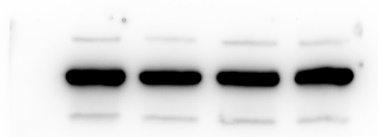


RhoA
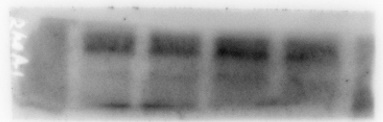

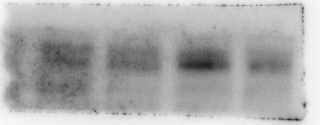

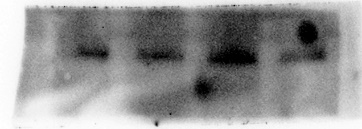


ROCK
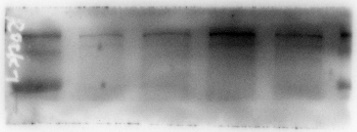

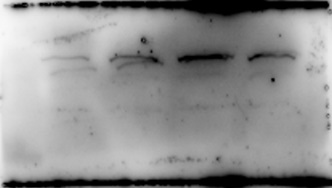

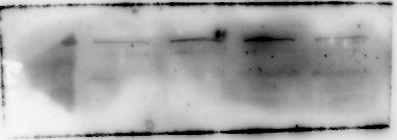


JNK
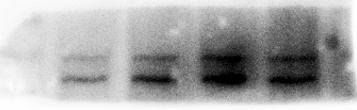

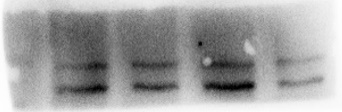

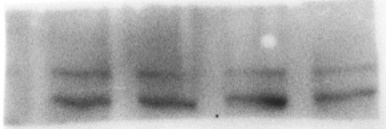


GAPDH
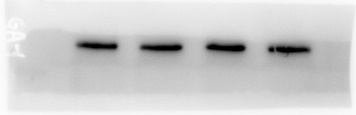

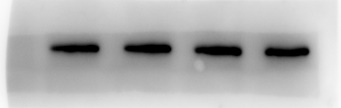

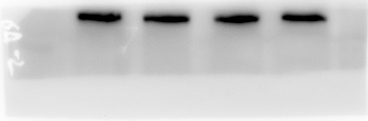

Supplement: Supplementary file 1 [file Data_Sheet_1.DOCX]
