# Supplementary figures and images for: Pitavastatin protects against neomycin-induced ototoxicity through inhibition of endoplasmic reticulum stress (part 1 of 2)
Source: Front Mol Neurosci. 2022 Aug 3;15:963083. doi: 10.3389/fnmol.2022.963083 (PMC9381809; doi:10.3389/fnmol.2022.963083)

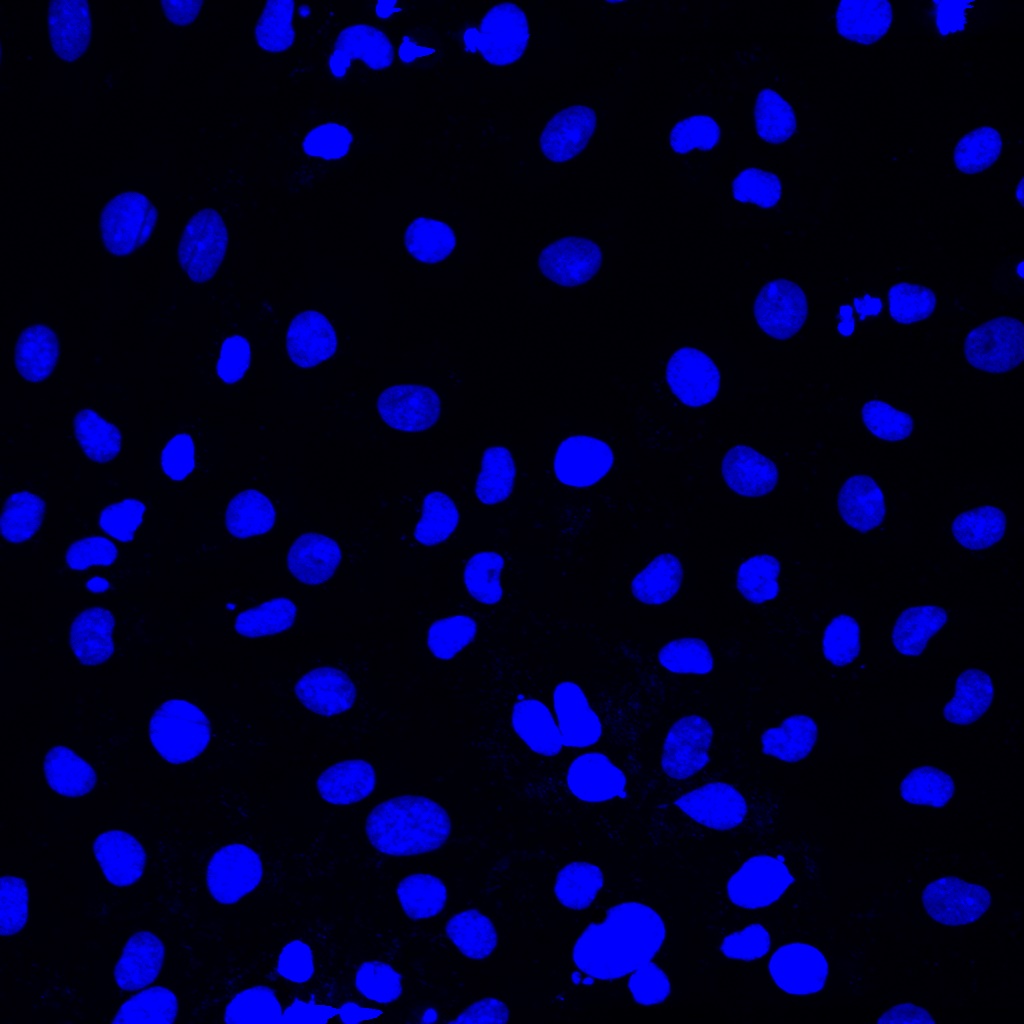

Supplement: Supplementary file 2 [file Data_Sheet_2.ZIP › Original data Fig. 1-3/Fig. 3/1.jpg]

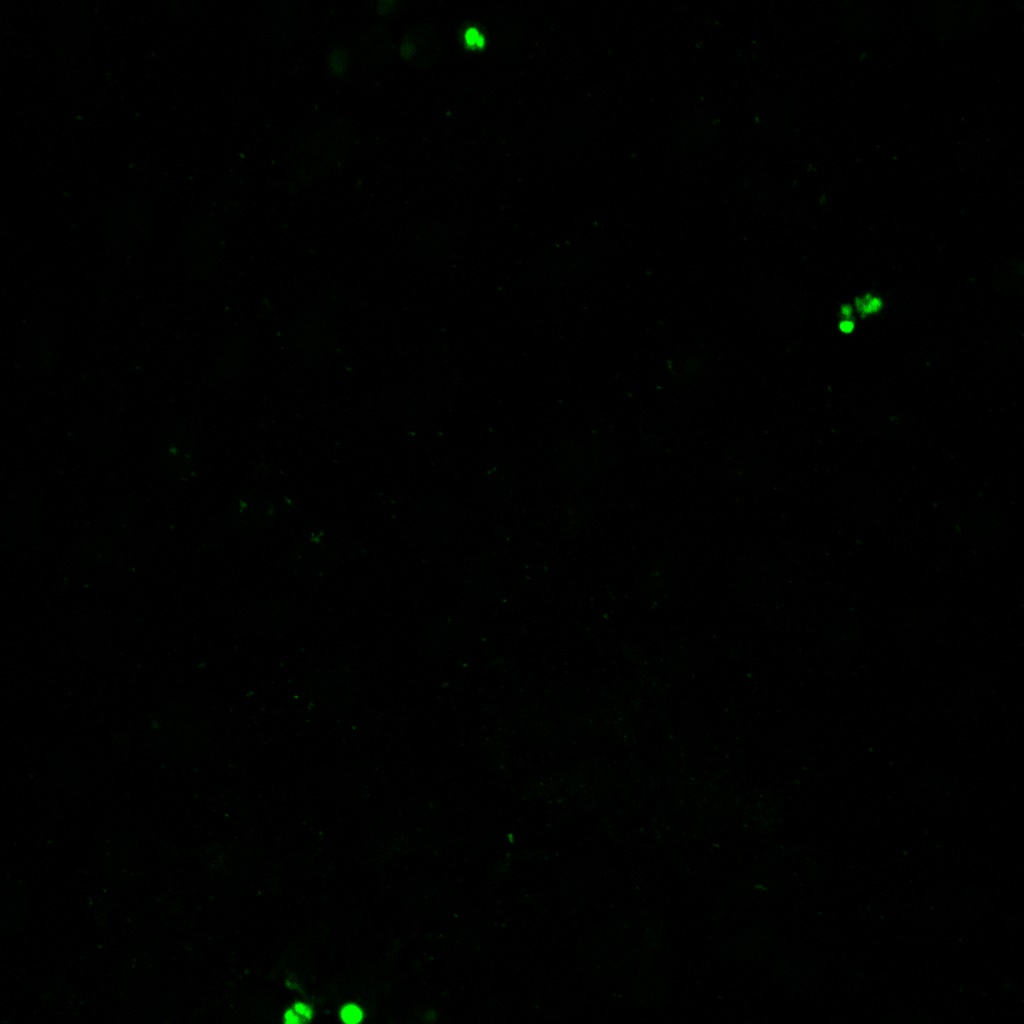

Supplement: Supplementary file 2 [file Data_Sheet_2.ZIP › Original data Fig. 1-3/Fig. 3/2.jpg]

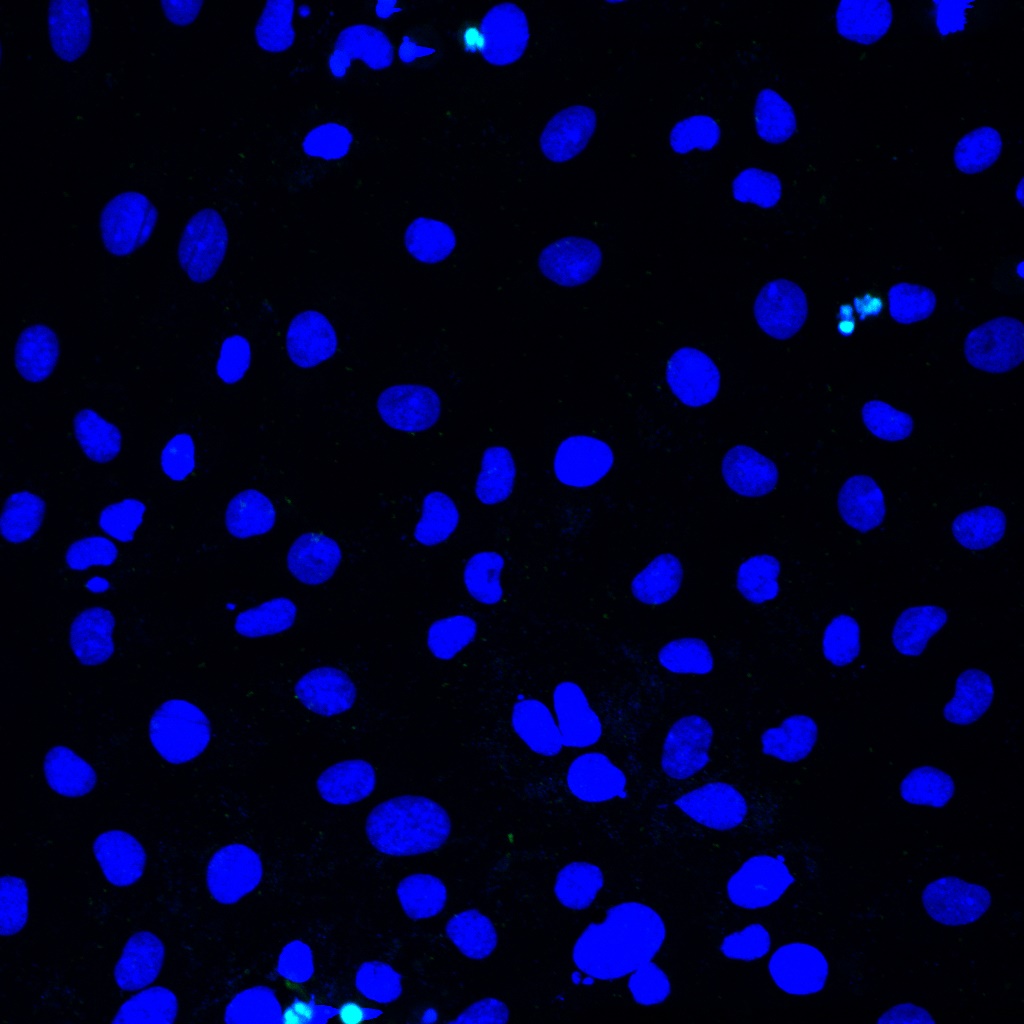

Supplement: Supplementary file 2 [file Data_Sheet_2.ZIP › Original data Fig. 1-3/Fig. 3/3.jpg]

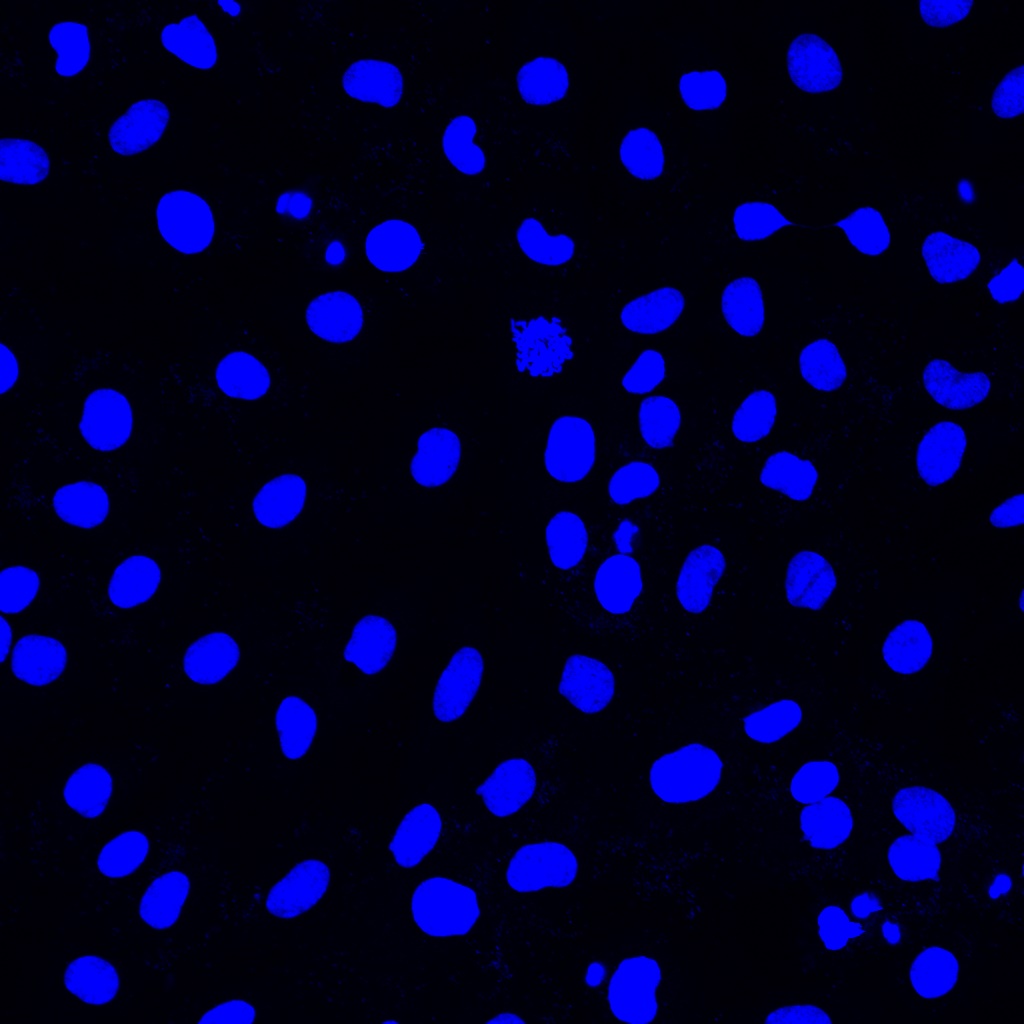

Supplement: Supplementary file 2 [file Data_Sheet_2.ZIP › Original data Fig. 1-3/Fig. 3/4.jpg]

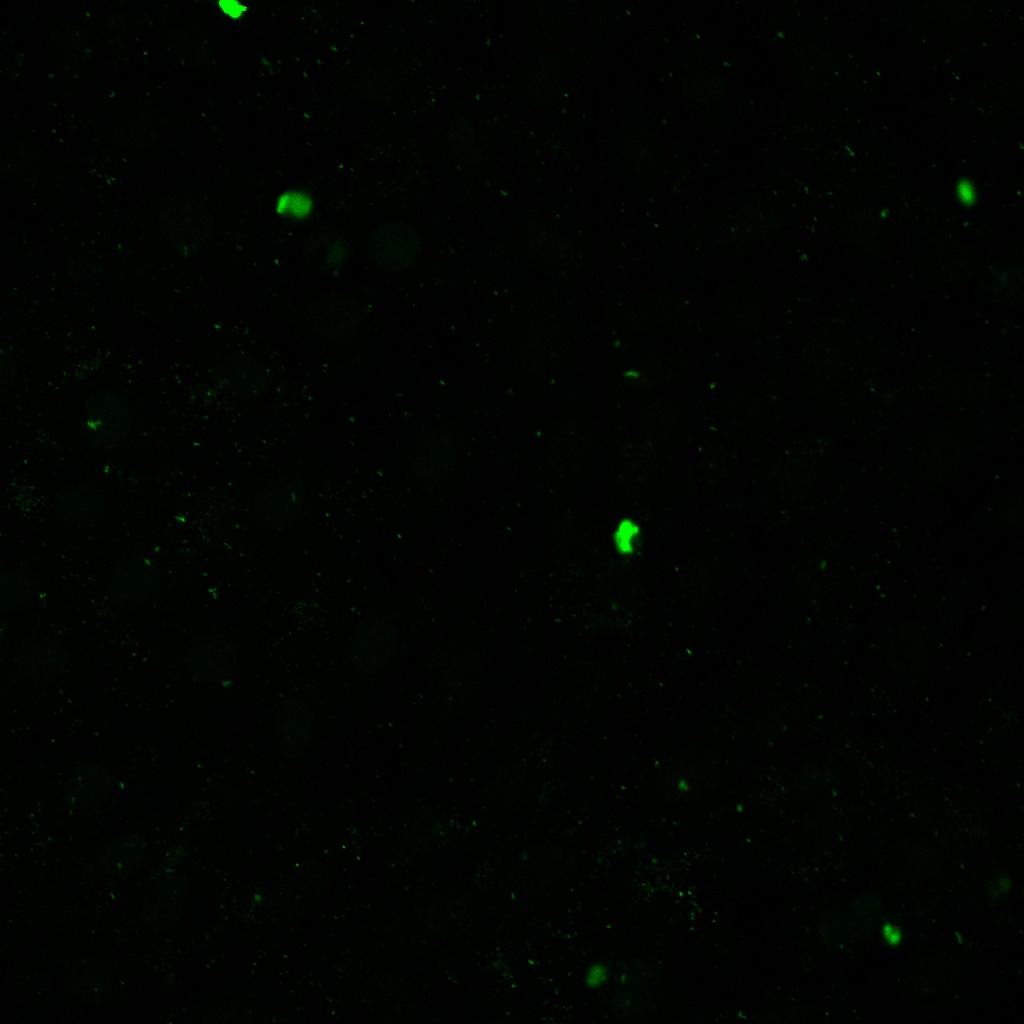

Supplement: Supplementary file 2 [file Data_Sheet_2.ZIP › Original data Fig. 1-3/Fig. 3/5.jpg]

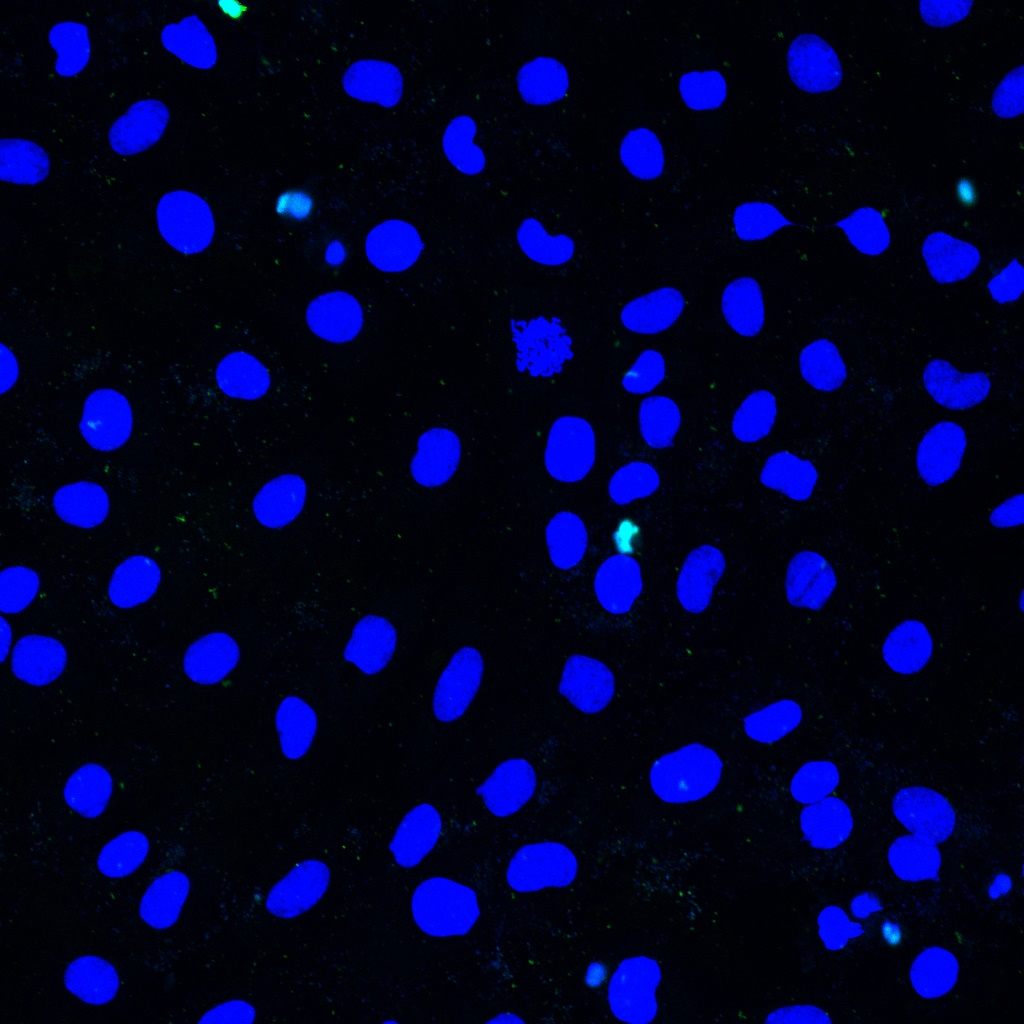

Supplement: Supplementary file 2 [file Data_Sheet_2.ZIP › Original data Fig. 1-3/Fig. 3/6.jpg]

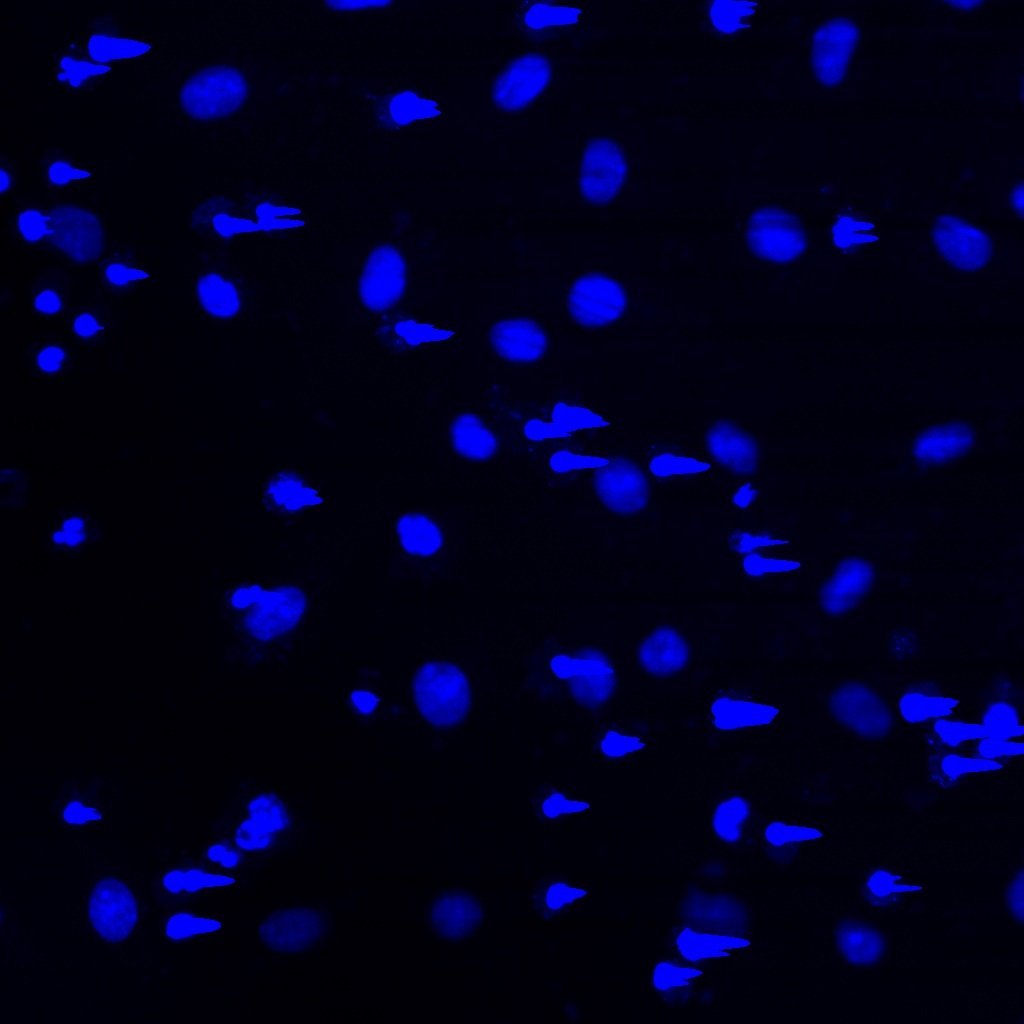

Supplement: Supplementary file 2 [file Data_Sheet_2.ZIP › Original data Fig. 1-3/Fig. 3/7.jpg]

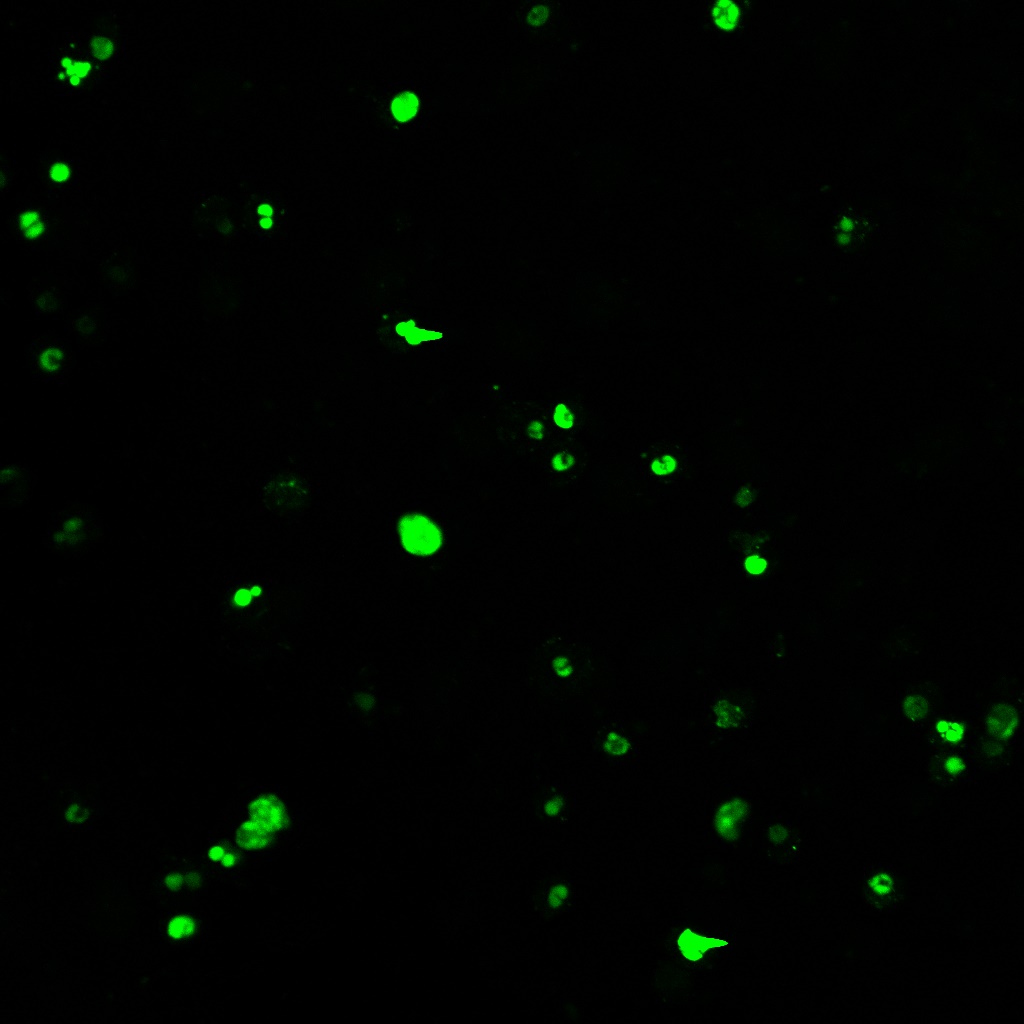

Supplement: Supplementary file 2 [file Data_Sheet_2.ZIP › Original data Fig. 1-3/Fig. 3/8.jpg]

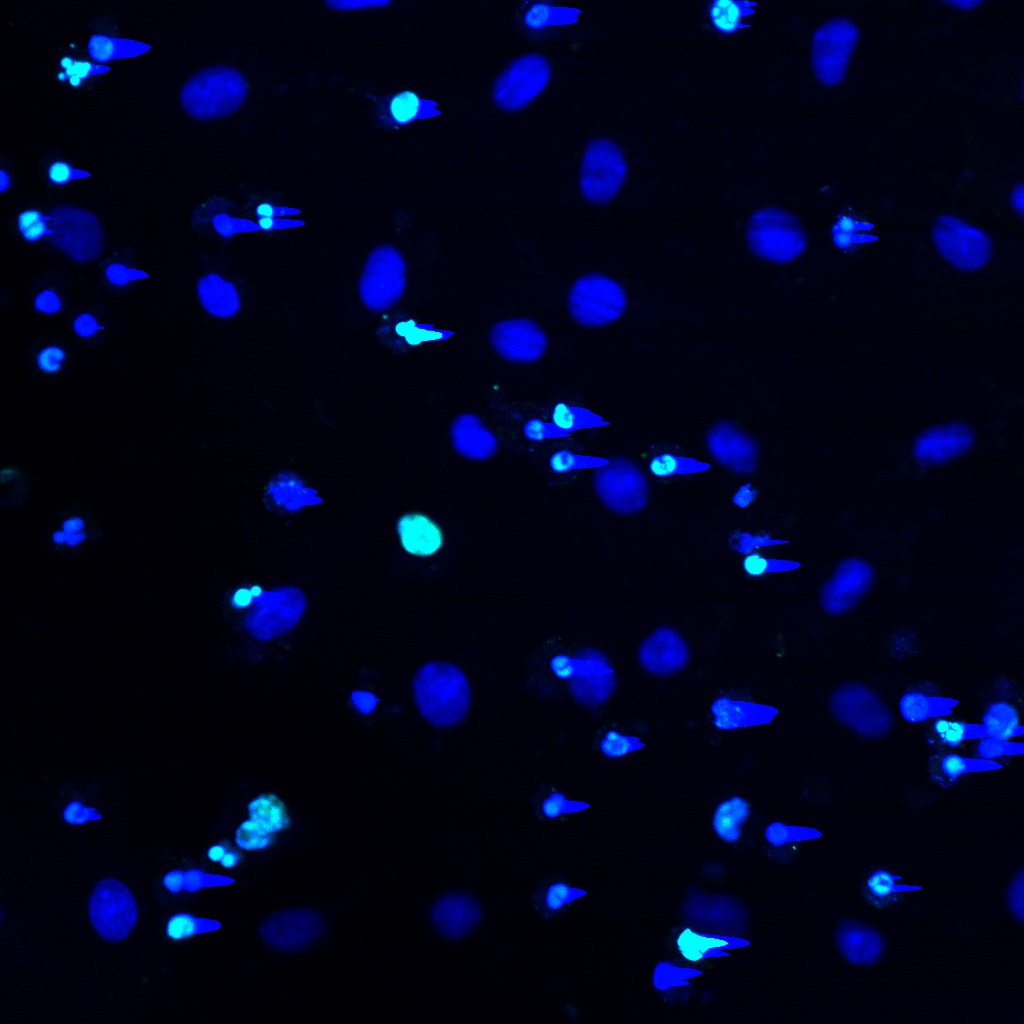

Supplement: Supplementary file 2 [file Data_Sheet_2.ZIP › Original data Fig. 1-3/Fig. 3/9.jpg]

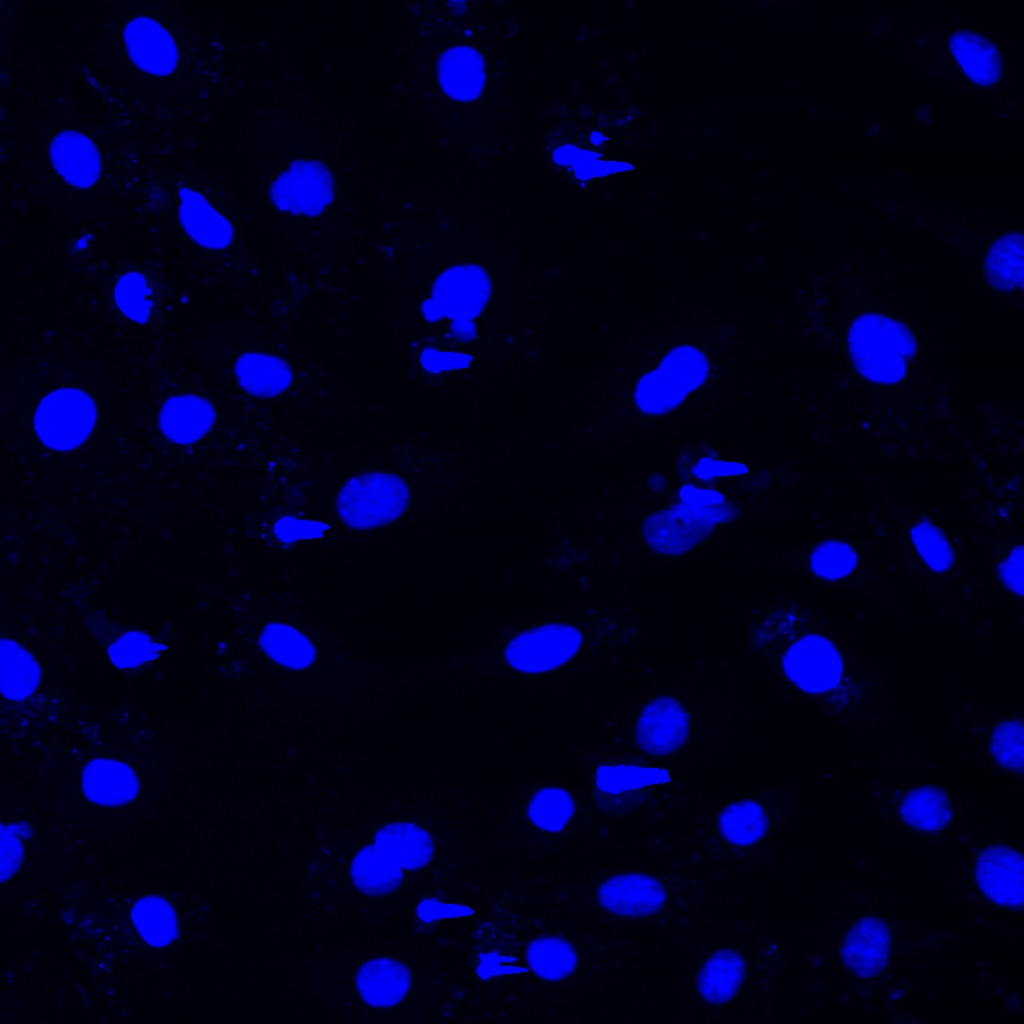

Supplement: Supplementary file 2 [file Data_Sheet_2.ZIP › Original data Fig. 1-3/Fig. 3/10.jpg]

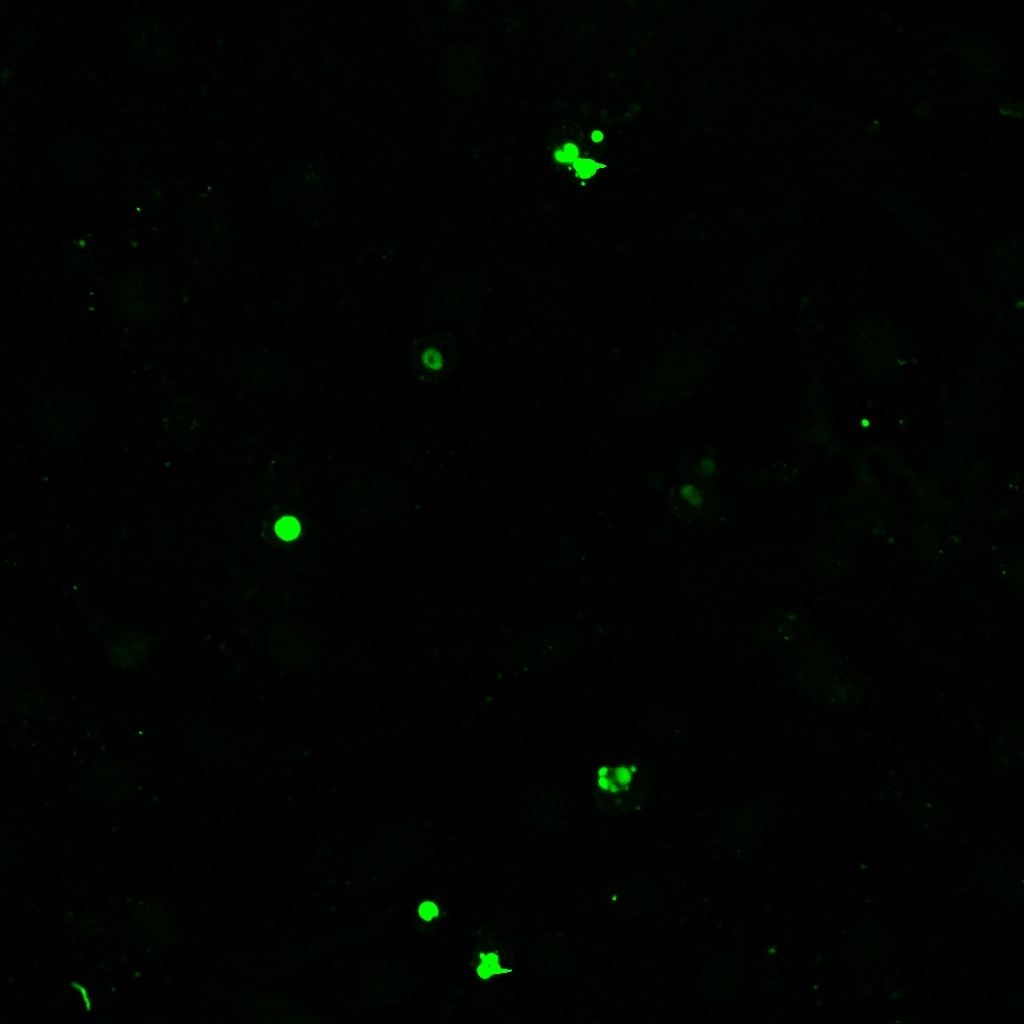

Supplement: Supplementary file 2 [file Data_Sheet_2.ZIP › Original data Fig. 1-3/Fig. 3/11.jpg]

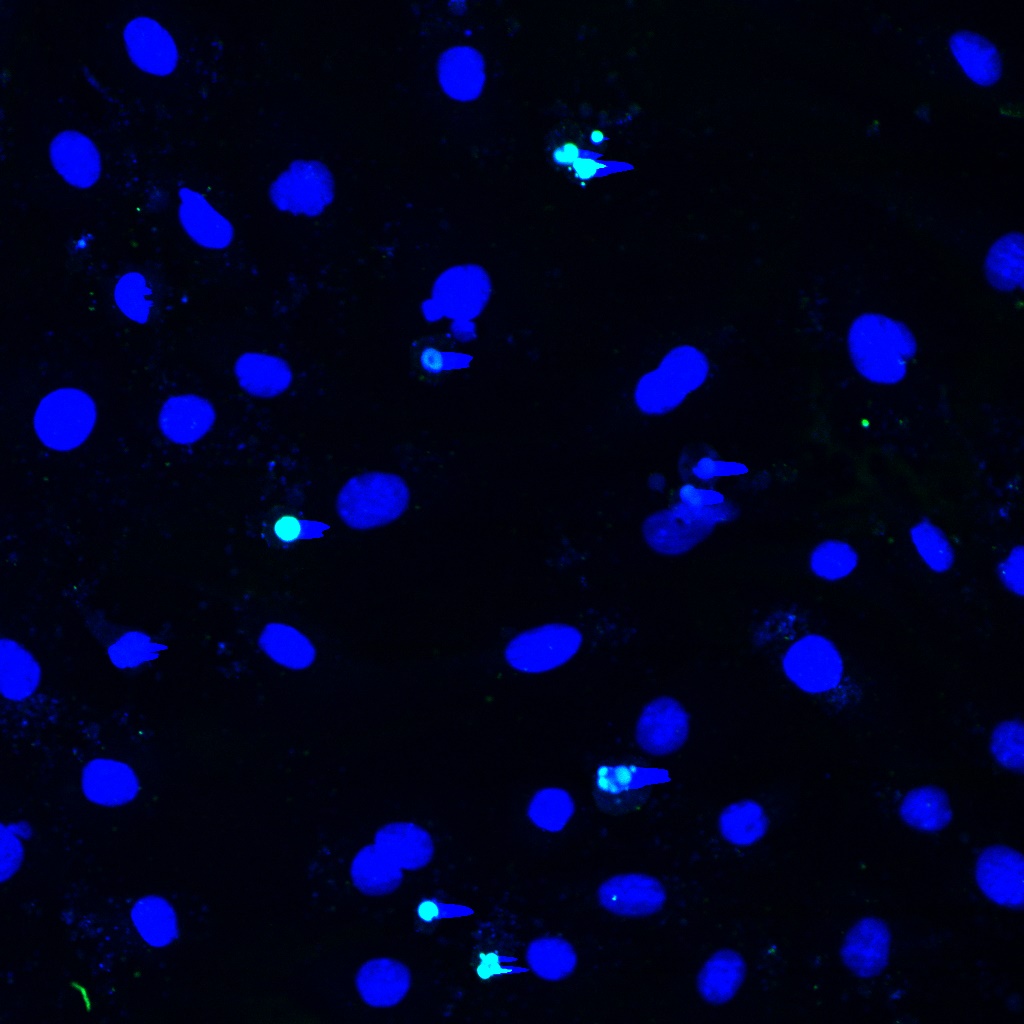

Supplement: Supplementary file 2 [file Data_Sheet_2.ZIP › Original data Fig. 1-3/Fig. 3/12.jpg]

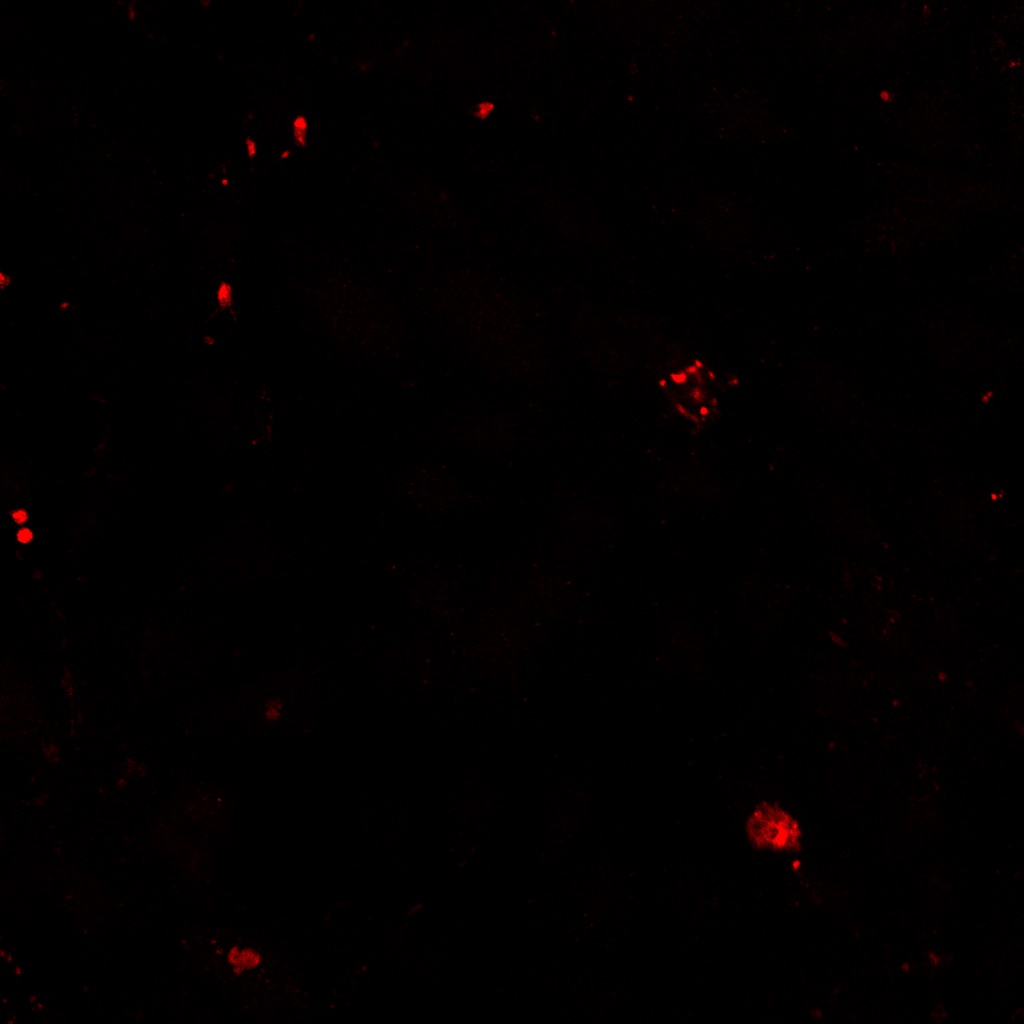

Supplement: Supplementary file 2 [file Data_Sheet_2.ZIP › Original data Fig. 1-3/Fig. 3/13.jpg]

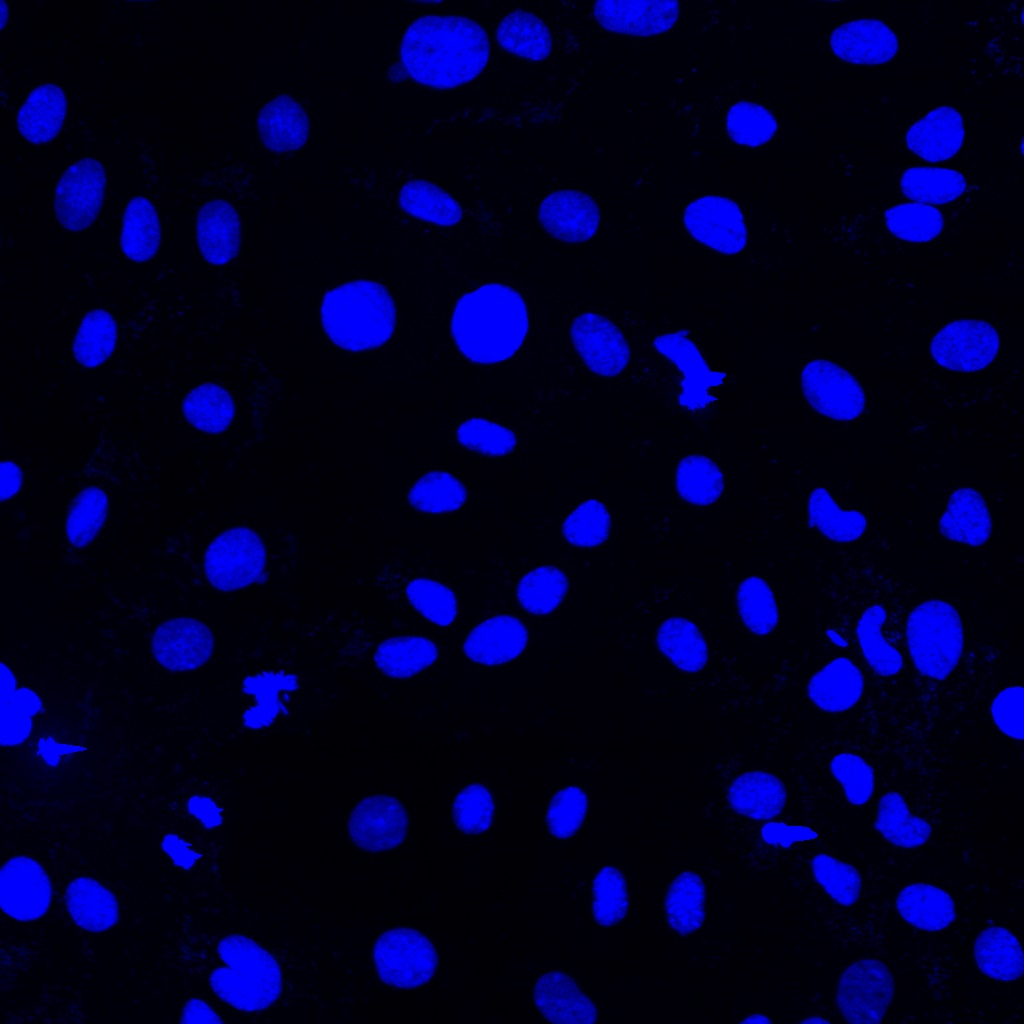

Supplement: Supplementary file 2 [file Data_Sheet_2.ZIP › Original data Fig. 1-3/Fig. 3/14.jpg]

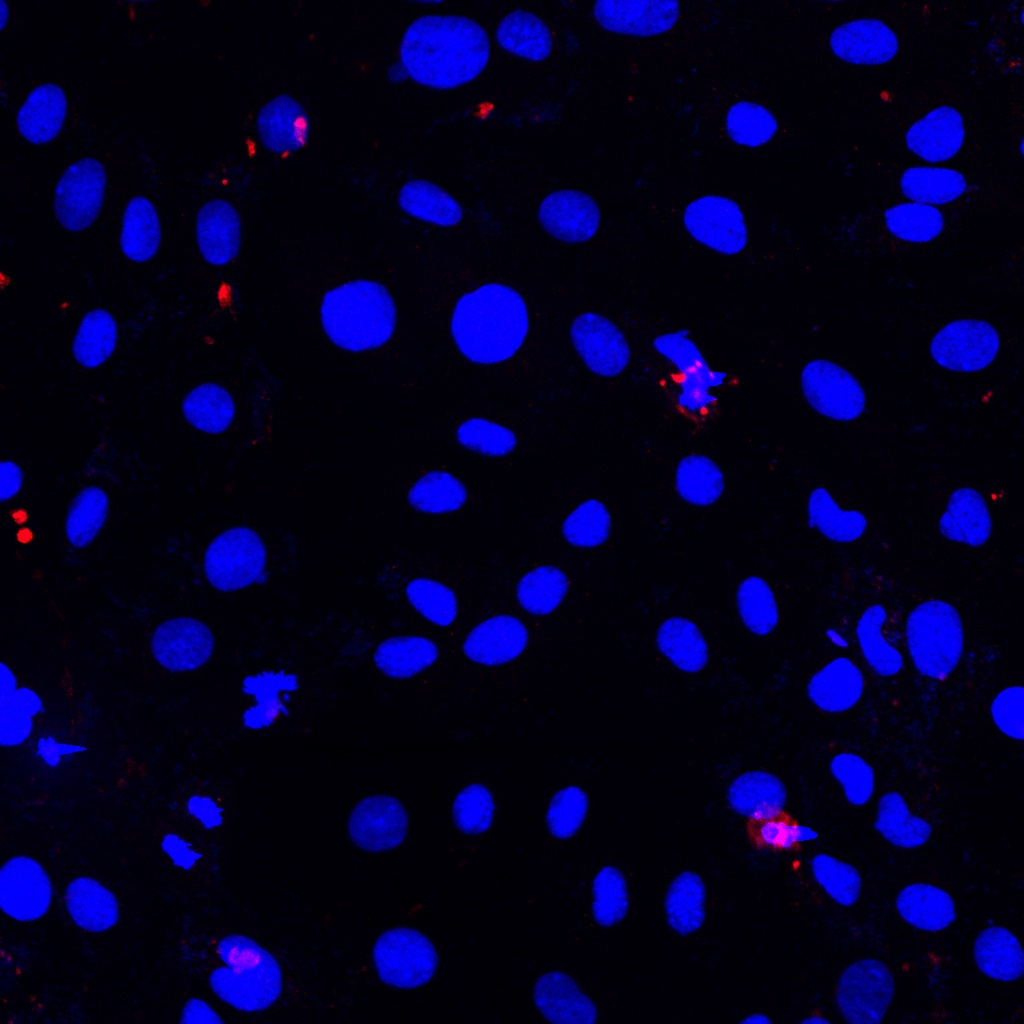

Supplement: Supplementary file 2 [file Data_Sheet_2.ZIP › Original data Fig. 1-3/Fig. 3/15.jpg]

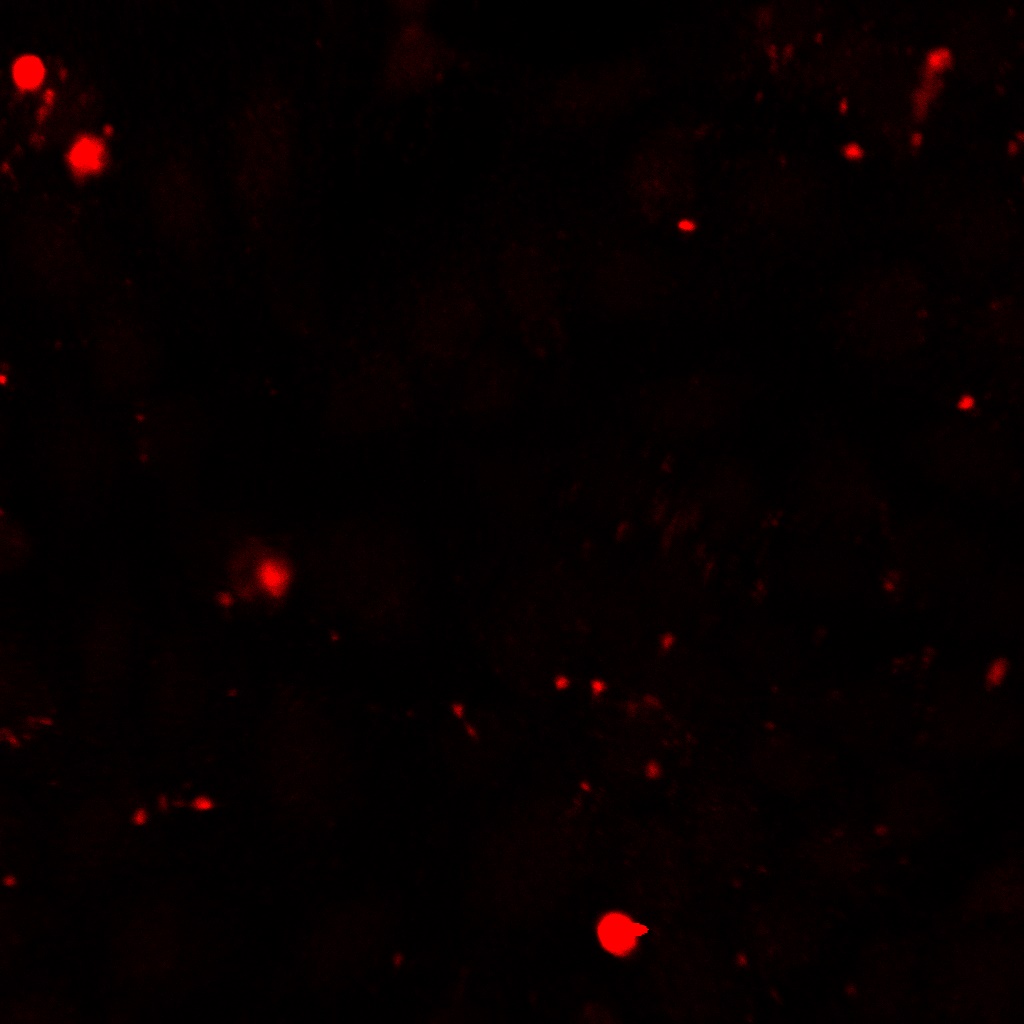

Supplement: Supplementary file 2 [file Data_Sheet_2.ZIP › Original data Fig. 1-3/Fig. 3/16.jpg]

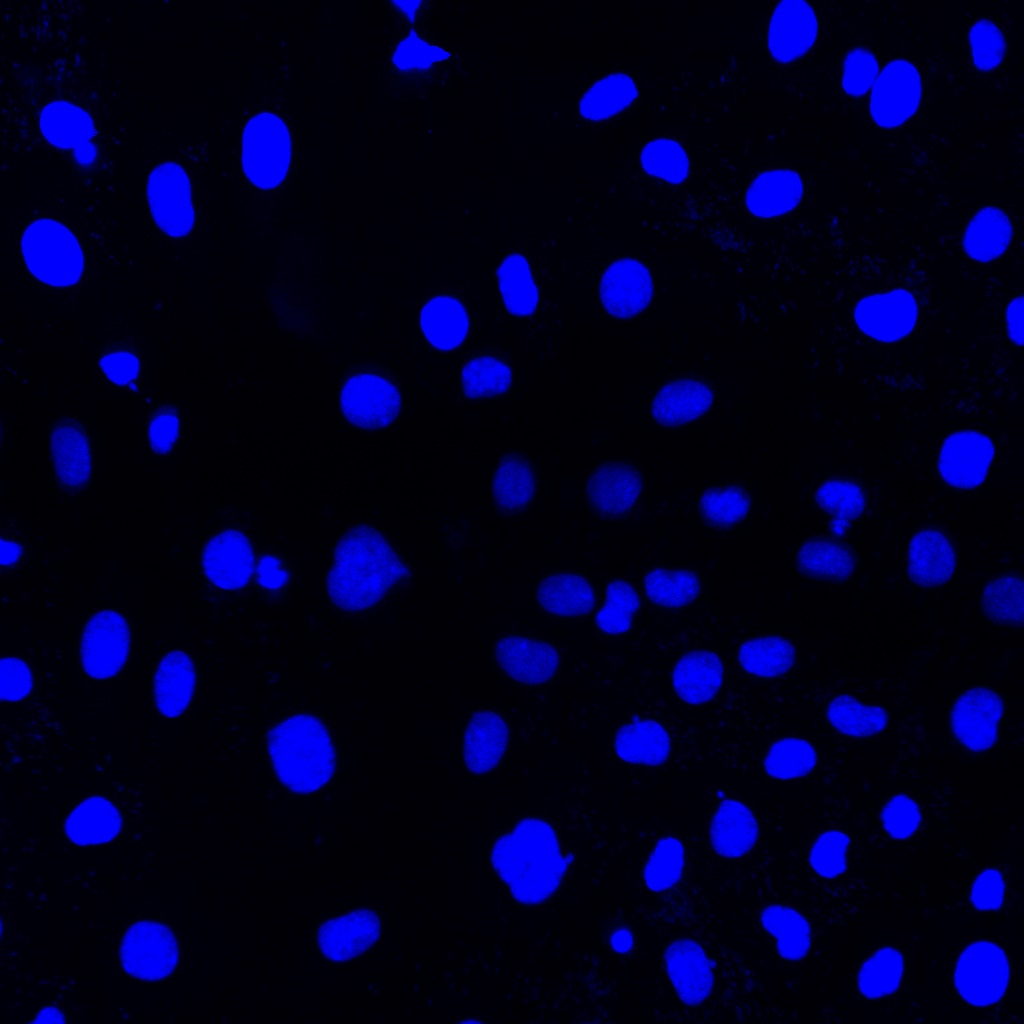

Supplement: Supplementary file 2 [file Data_Sheet_2.ZIP › Original data Fig. 1-3/Fig. 3/17.jpg]

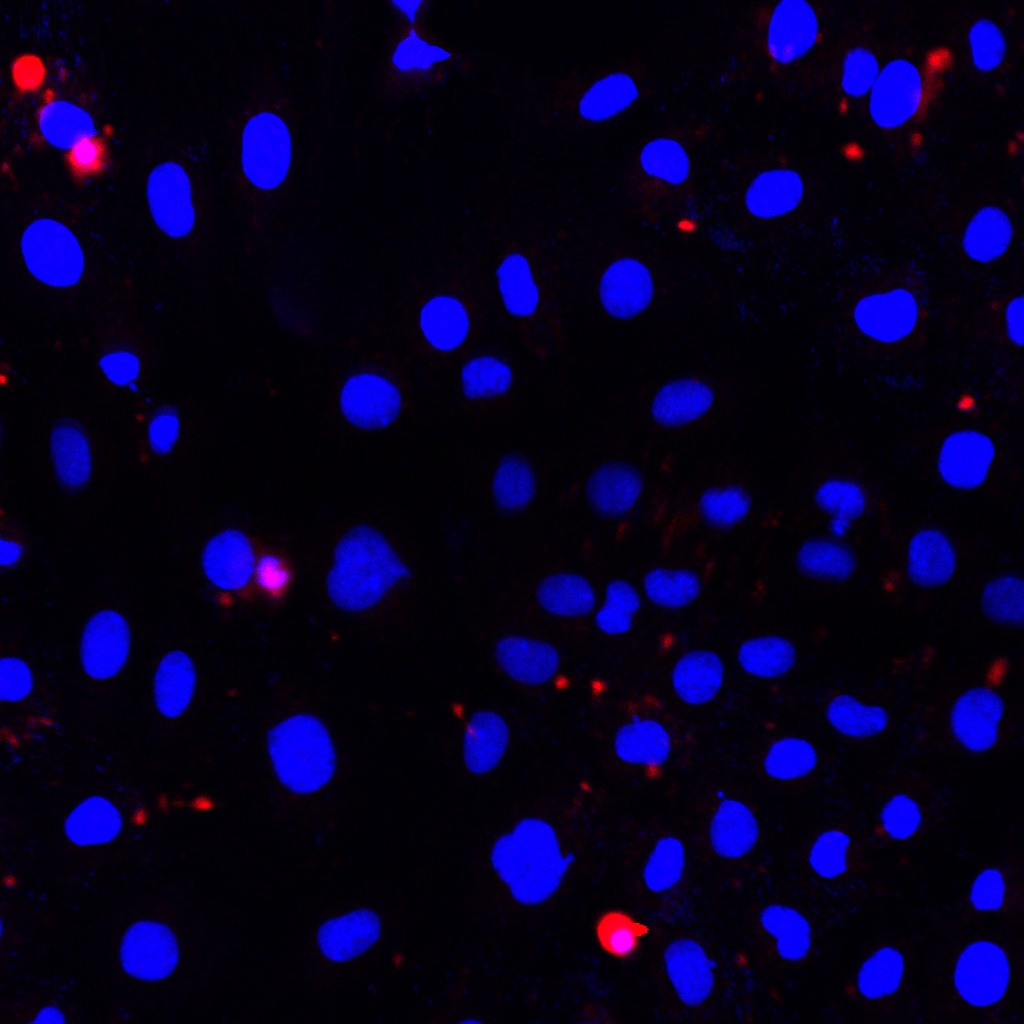

Supplement: Supplementary file 2 [file Data_Sheet_2.ZIP › Original data Fig. 1-3/Fig. 3/18.jpg]

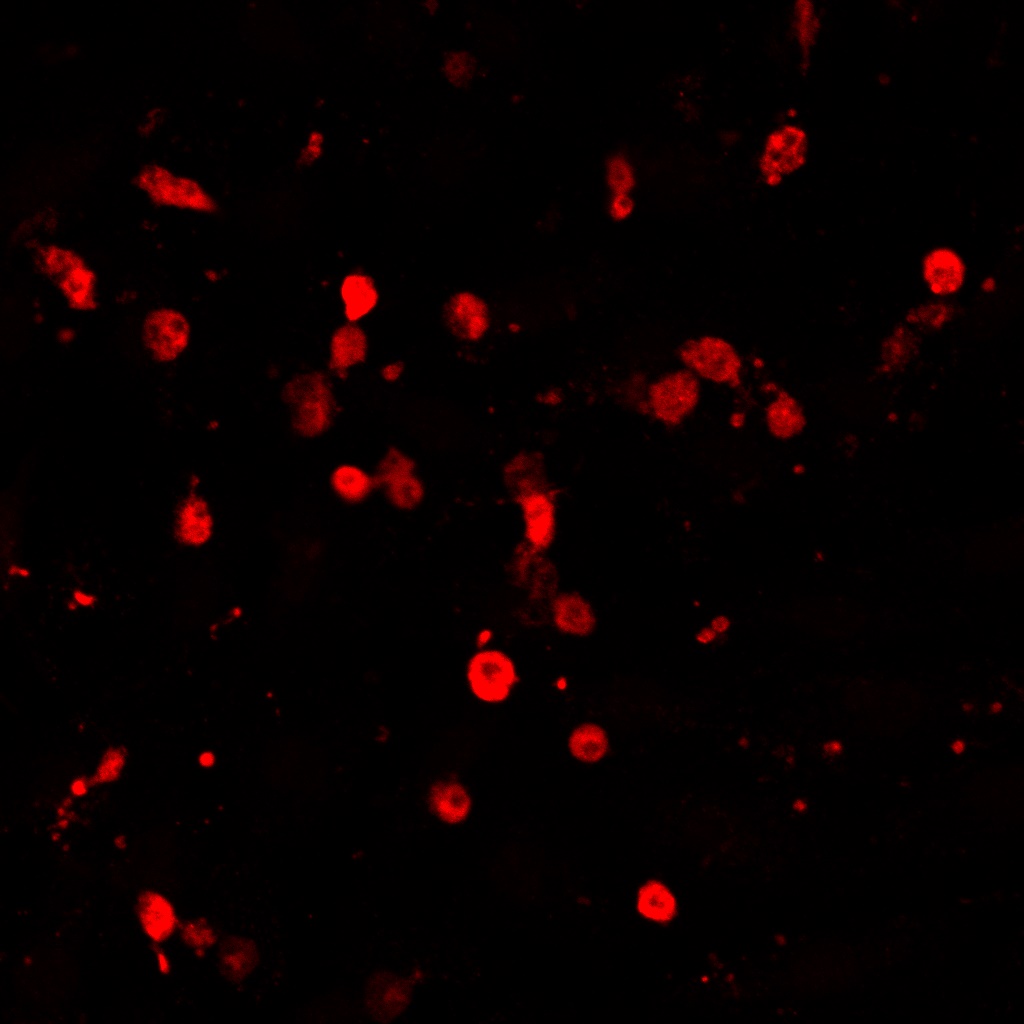

Supplement: Supplementary file 2 [file Data_Sheet_2.ZIP › Original data Fig. 1-3/Fig. 3/19.jpg]

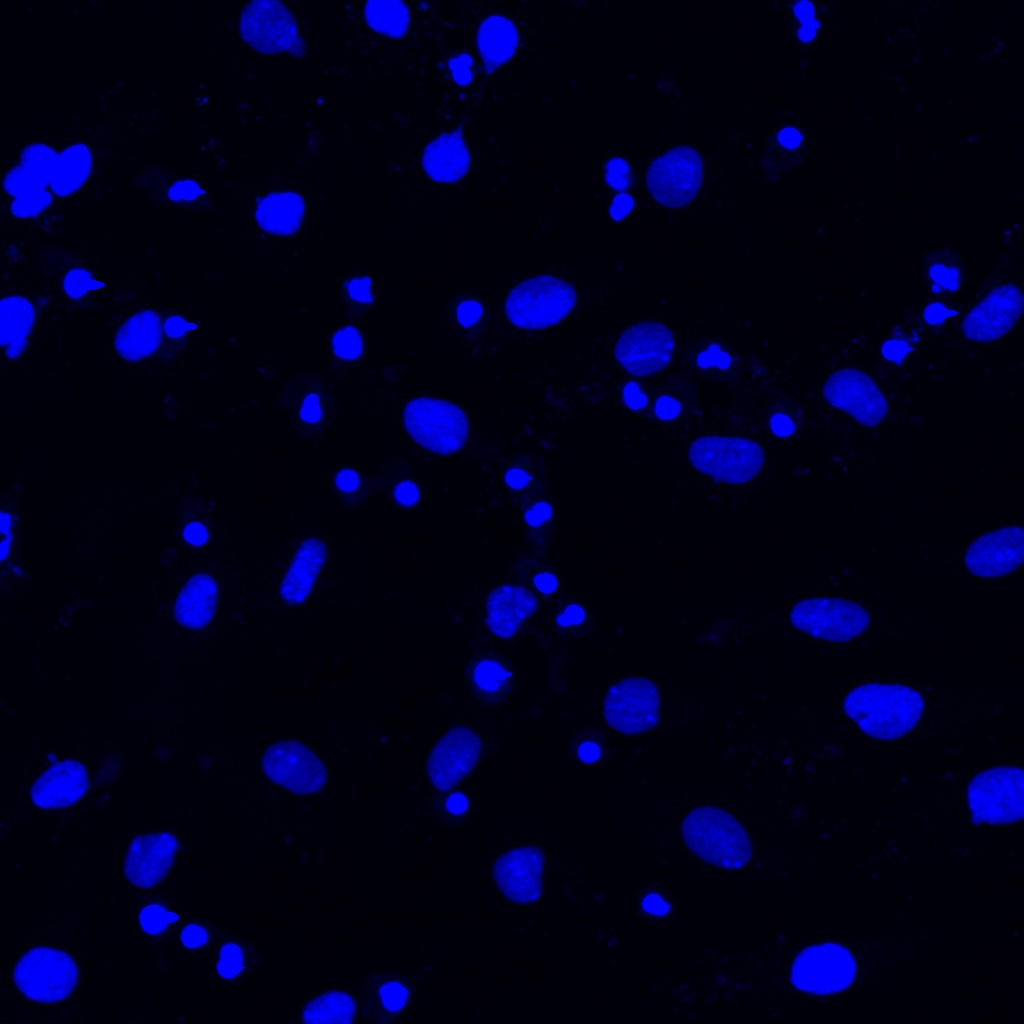

Supplement: Supplementary file 2 [file Data_Sheet_2.ZIP › Original data Fig. 1-3/Fig. 3/20.jpg]

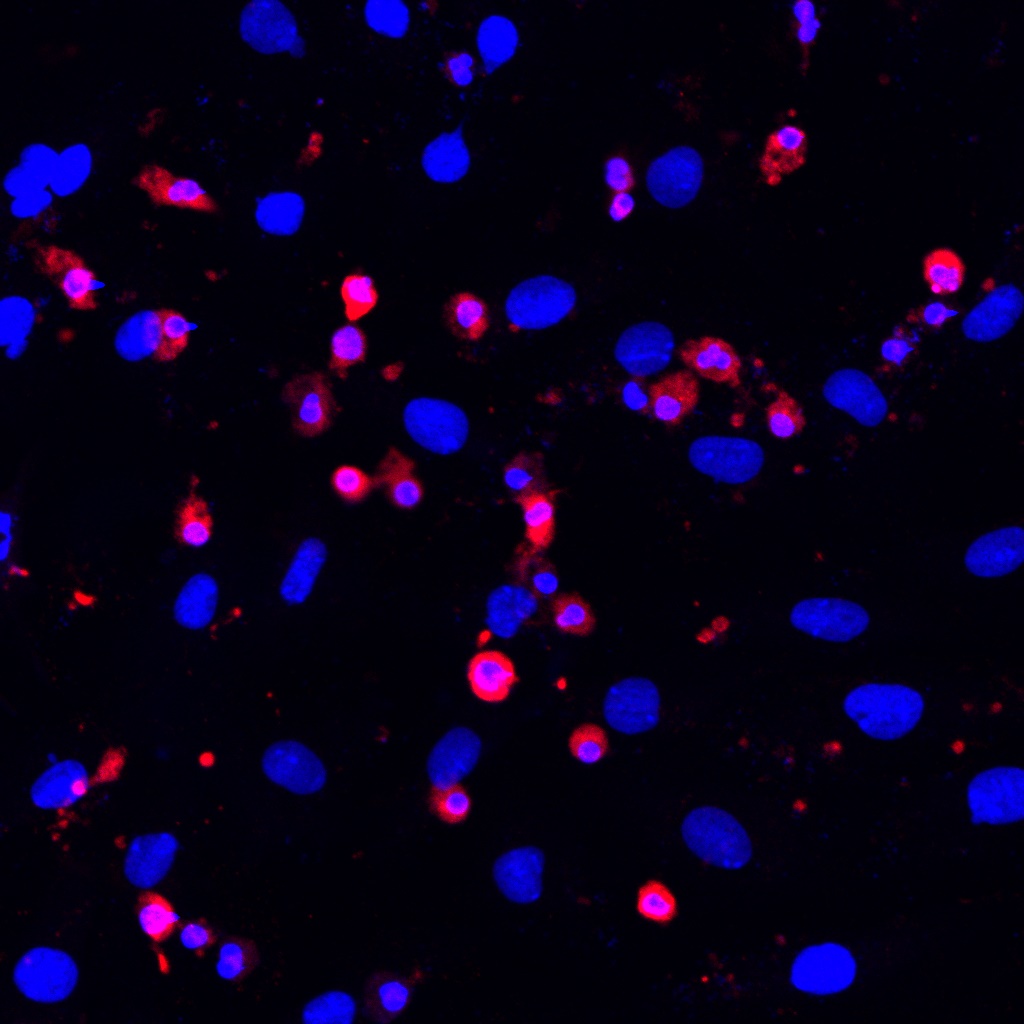

Supplement: Supplementary file 2 [file Data_Sheet_2.ZIP › Original data Fig. 1-3/Fig. 3/21.jpg]

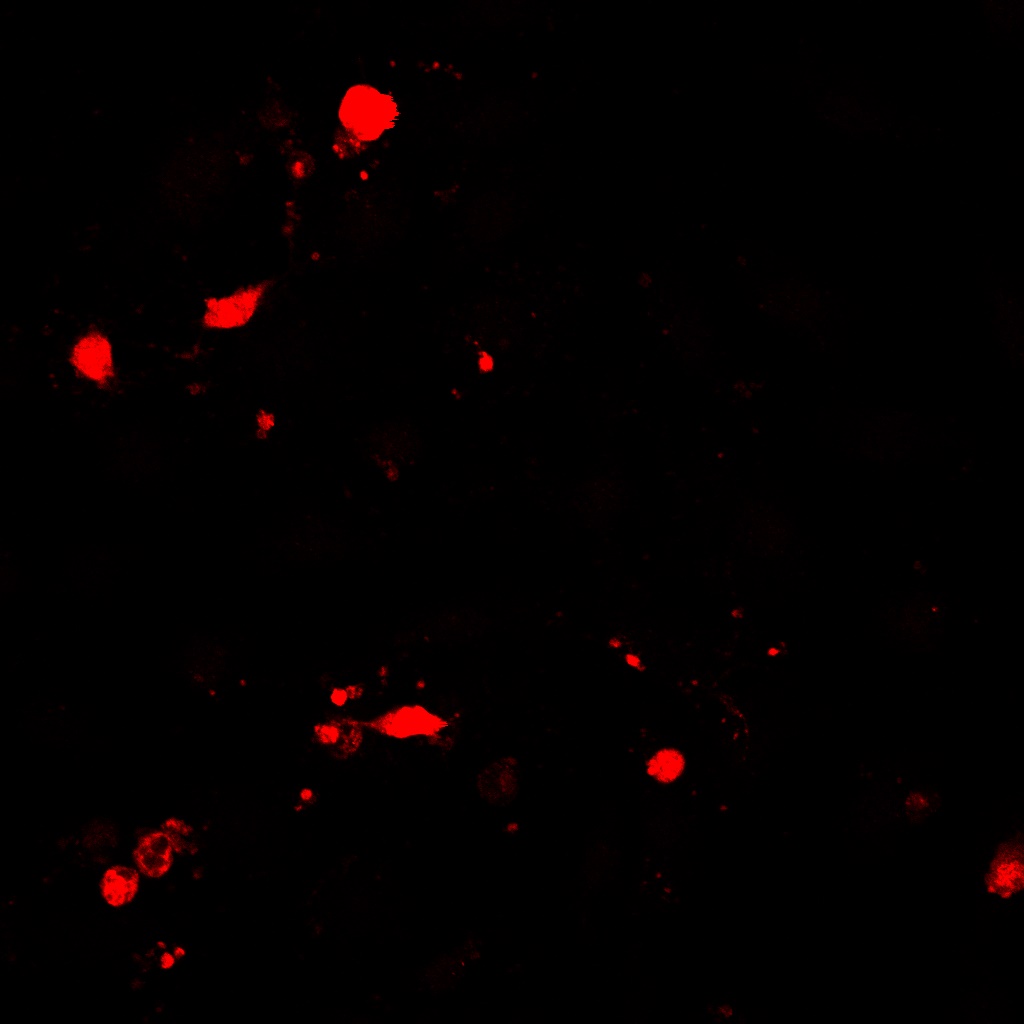

Supplement: Supplementary file 2 [file Data_Sheet_2.ZIP › Original data Fig. 1-3/Fig. 3/22.jpg]

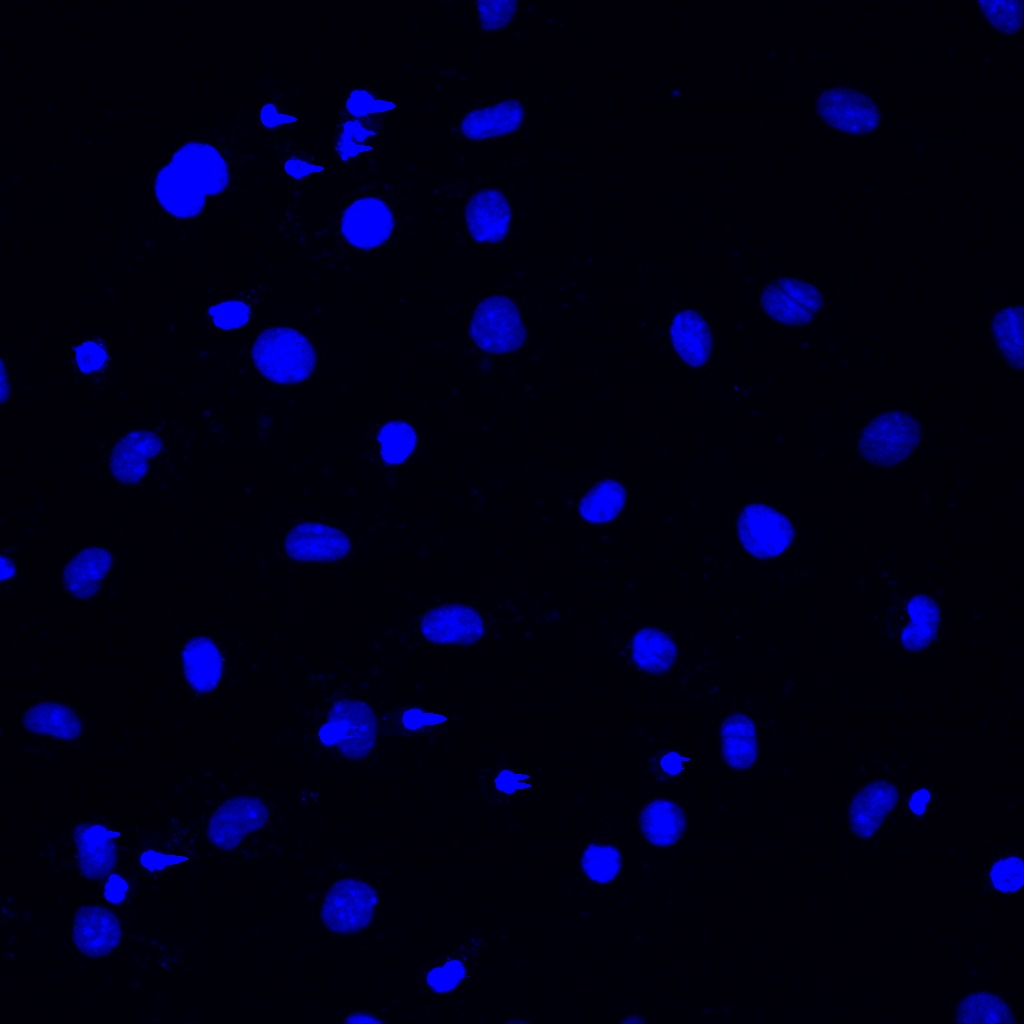

Supplement: Supplementary file 2 [file Data_Sheet_2.ZIP › Original data Fig. 1-3/Fig. 3/23.jpg]

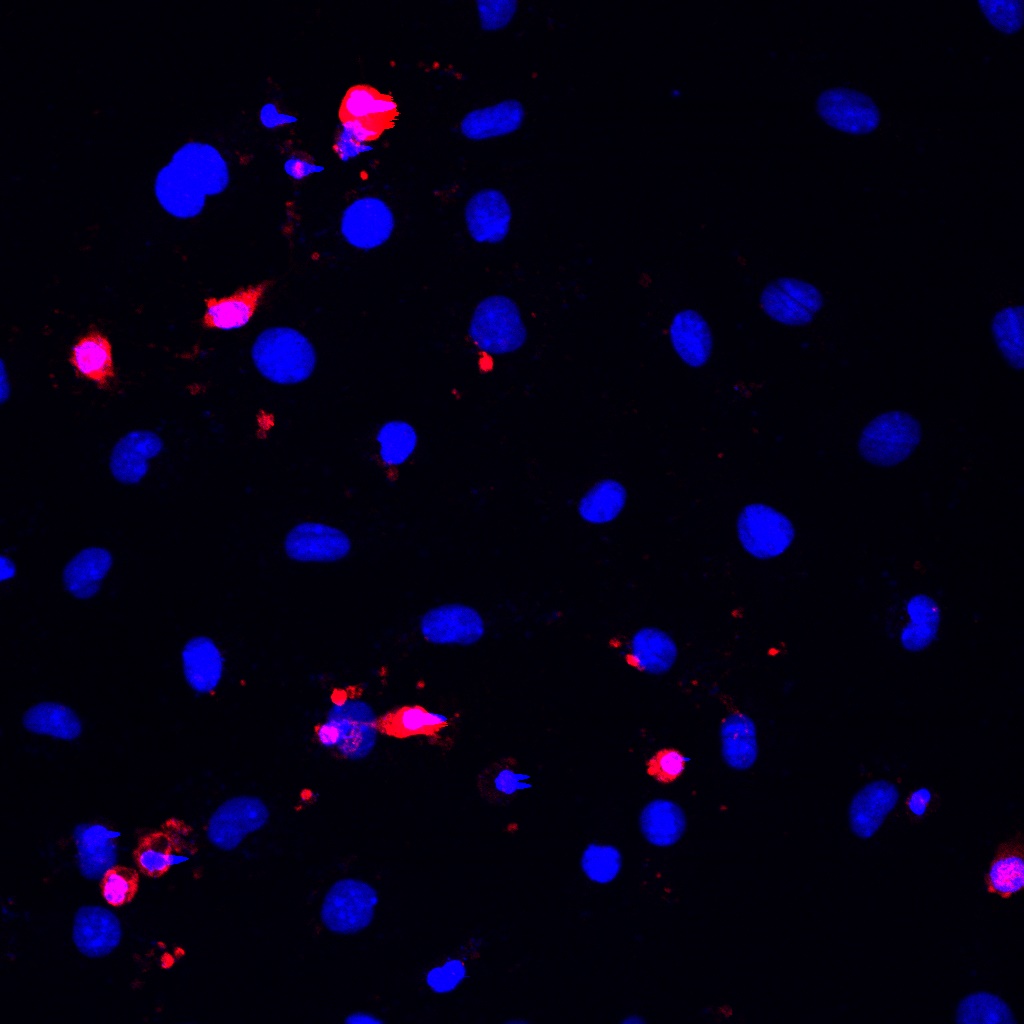

Supplement: Supplementary file 2 [file Data_Sheet_2.ZIP › Original data Fig. 1-3/Fig. 3/24.jpg]

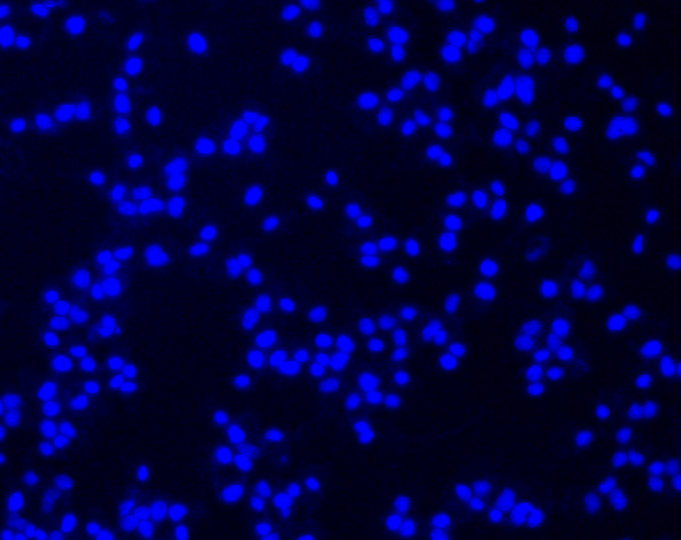

Supplement: Supplementary file 2 [file Data_Sheet_2.ZIP › Original data Fig. 1-3/Fig. 3/27.tif]

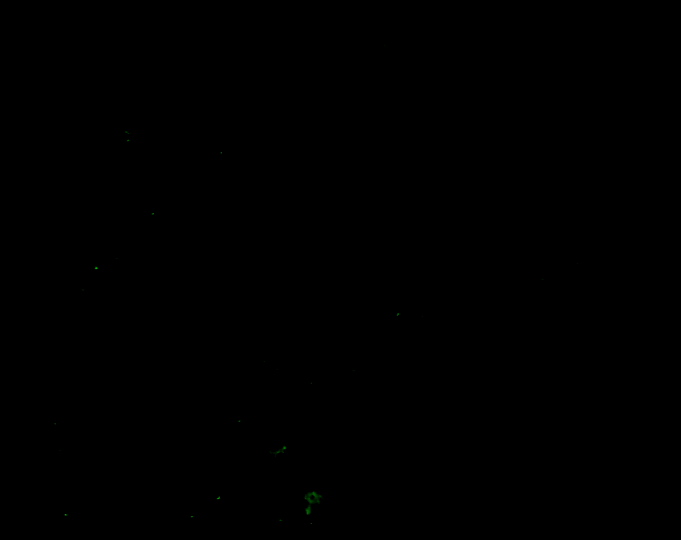

Supplement: Supplementary file 2 [file Data_Sheet_2.ZIP › Original data Fig. 1-3/Fig. 3/25.tif]

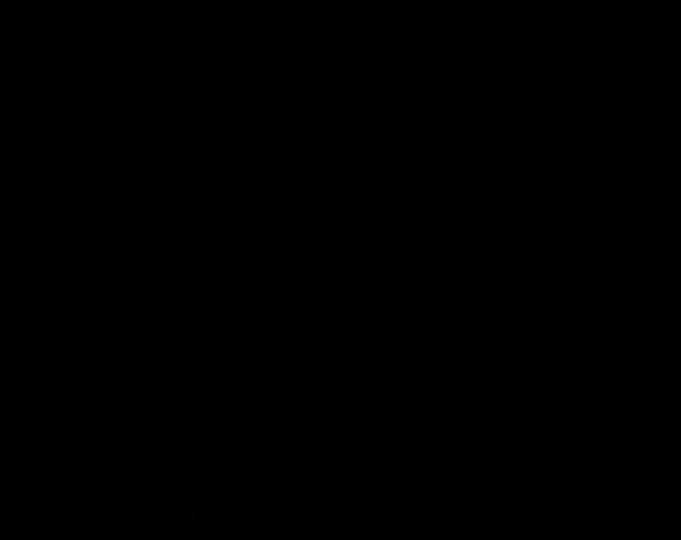

Supplement: Supplementary file 2 [file Data_Sheet_2.ZIP › Original data Fig. 1-3/Fig. 3/26.tif]

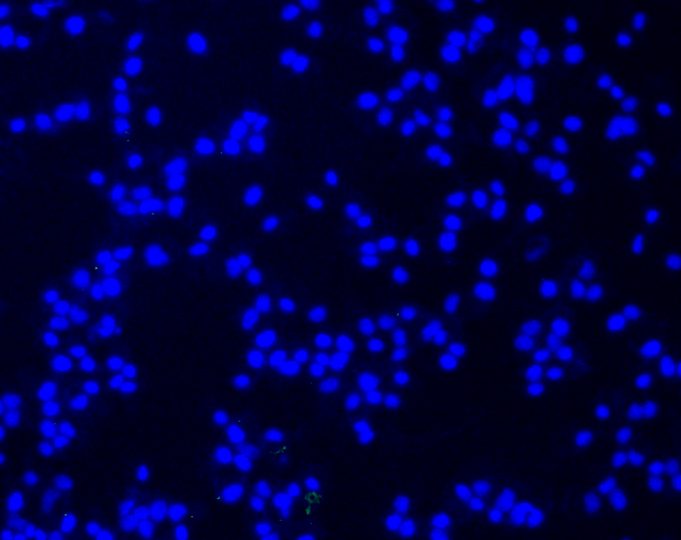

Supplement: Supplementary file 2 [file Data_Sheet_2.ZIP › Original data Fig. 1-3/Fig. 3/28.tif]

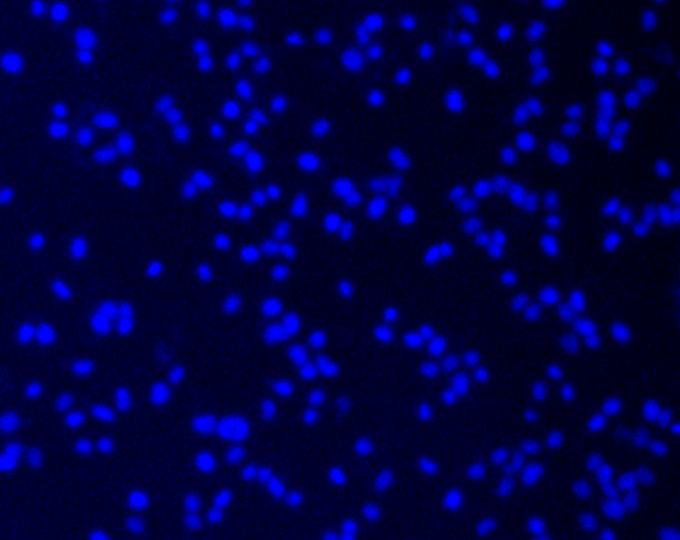

Supplement: Supplementary file 2 [file Data_Sheet_2.ZIP › Original data Fig. 1-3/Fig. 3/31.tif]

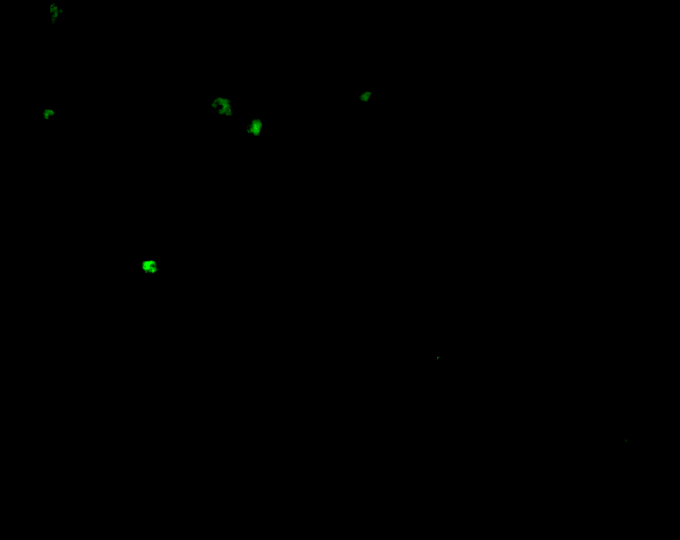

Supplement: Supplementary file 2 [file Data_Sheet_2.ZIP › Original data Fig. 1-3/Fig. 3/29.tif]

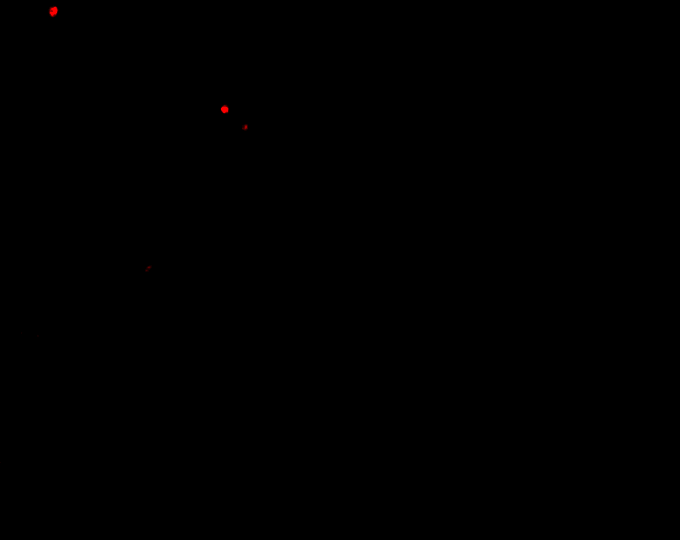

Supplement: Supplementary file 2 [file Data_Sheet_2.ZIP › Original data Fig. 1-3/Fig. 3/30.tif]

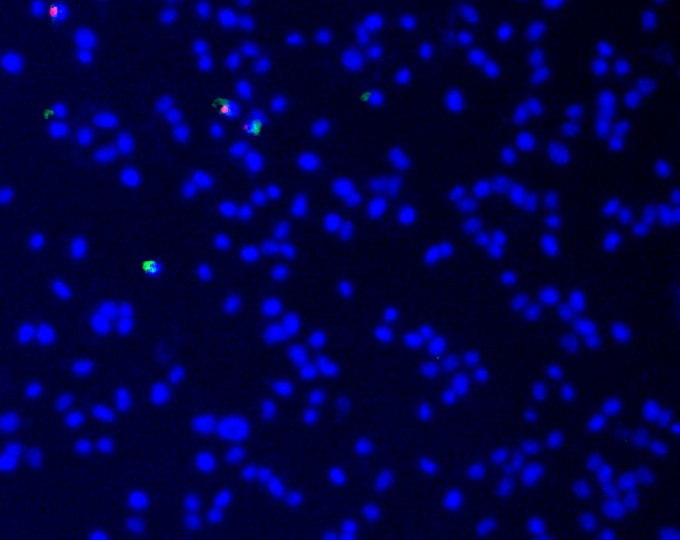

Supplement: Supplementary file 2 [file Data_Sheet_2.ZIP › Original data Fig. 1-3/Fig. 3/32.tif]

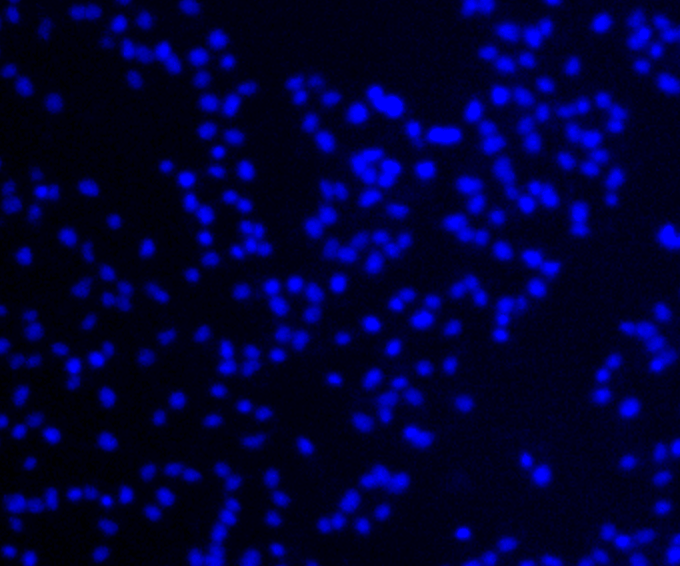

Supplement: Supplementary file 2 [file Data_Sheet_2.ZIP › Original data Fig. 1-3/Fig. 3/35.tif]

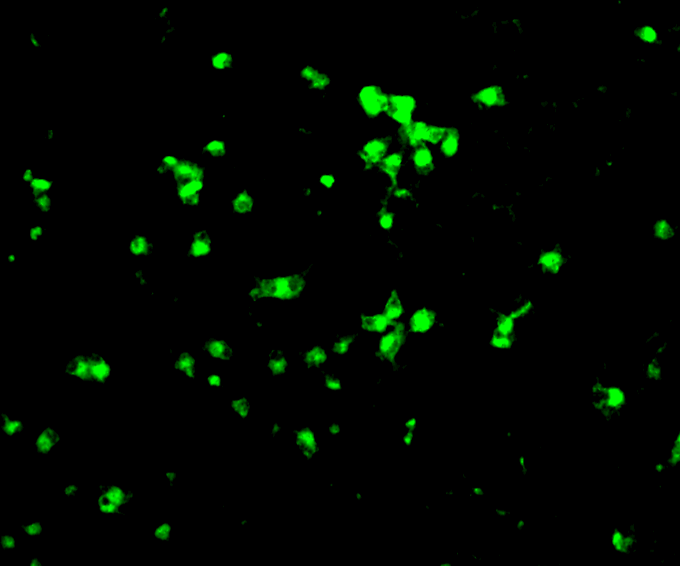

Supplement: Supplementary file 2 [file Data_Sheet_2.ZIP › Original data Fig. 1-3/Fig. 3/33.tif]

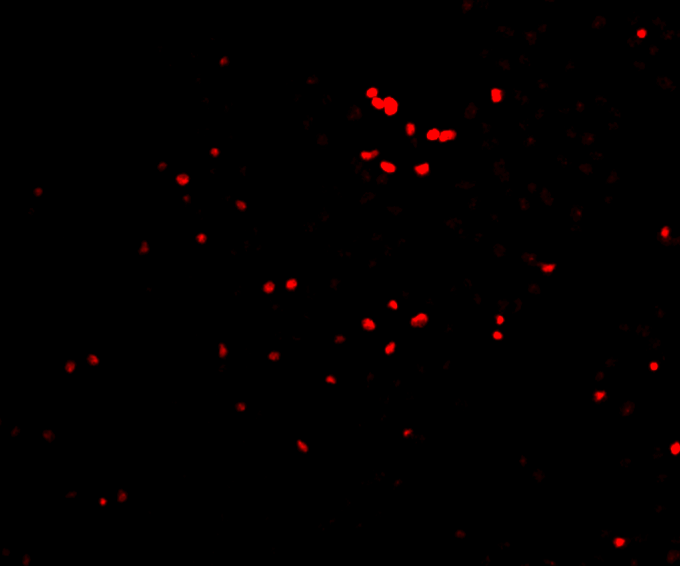

Supplement: Supplementary file 2 [file Data_Sheet_2.ZIP › Original data Fig. 1-3/Fig. 3/34.tif]

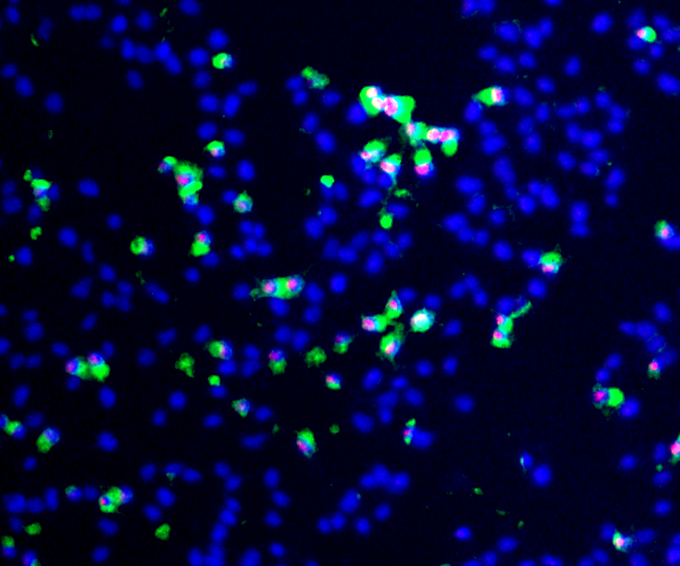

Supplement: Supplementary file 2 [file Data_Sheet_2.ZIP › Original data Fig. 1-3/Fig. 3/36.tif]

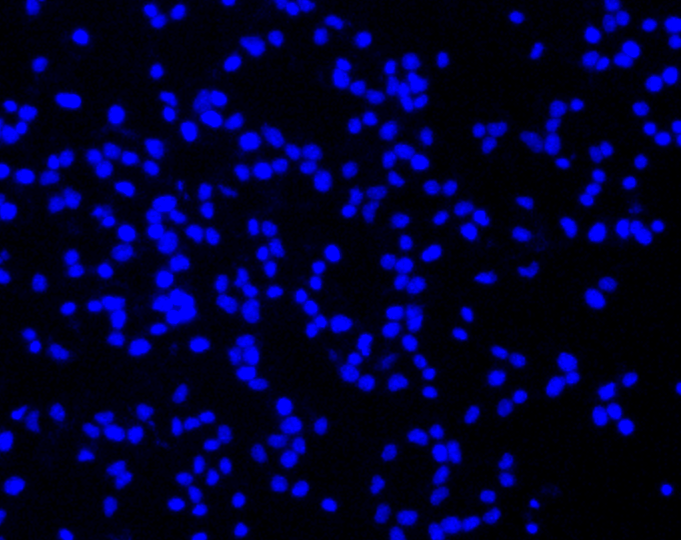

Supplement: Supplementary file 2 [file Data_Sheet_2.ZIP › Original data Fig. 1-3/Fig. 3/39.tif]

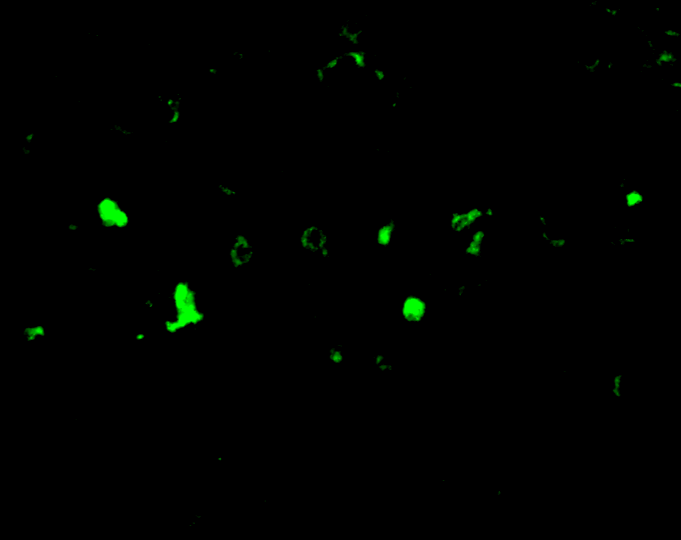

Supplement: Supplementary file 2 [file Data_Sheet_2.ZIP › Original data Fig. 1-3/Fig. 3/37.tif]

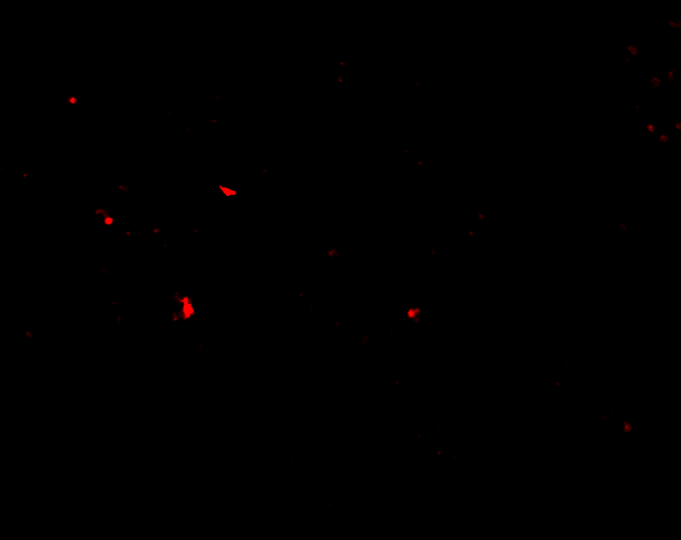

Supplement: Supplementary file 2 [file Data_Sheet_2.ZIP › Original data Fig. 1-3/Fig. 3/38.tif]

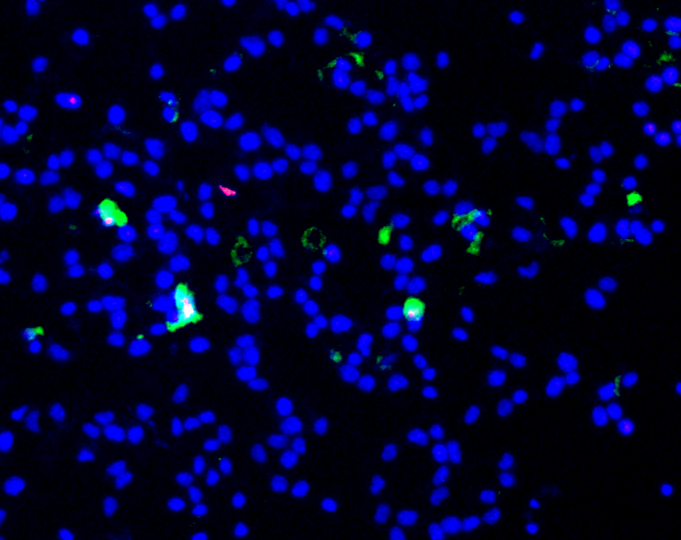

Supplement: Supplementary file 2 [file Data_Sheet_2.ZIP › Original data Fig. 1-3/Fig. 3/40.tif]

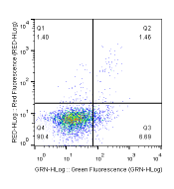

Supplement: Supplementary file 2 [file Data_Sheet_2.ZIP › Original data Fig. 1-3/Fig. 3/41.tif]

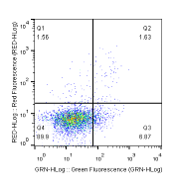

Supplement: Supplementary file 2 [file Data_Sheet_2.ZIP › Original data Fig. 1-3/Fig. 3/42.tif]

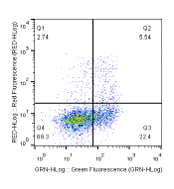

Supplement: Supplementary file 2 [file Data_Sheet_2.ZIP › Original data Fig. 1-3/Fig. 3/43.tif]

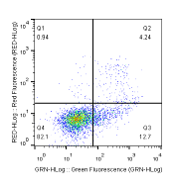

Supplement: Supplementary file 2 [file Data_Sheet_2.ZIP › Original data Fig. 1-3/Fig. 3/44.tif]

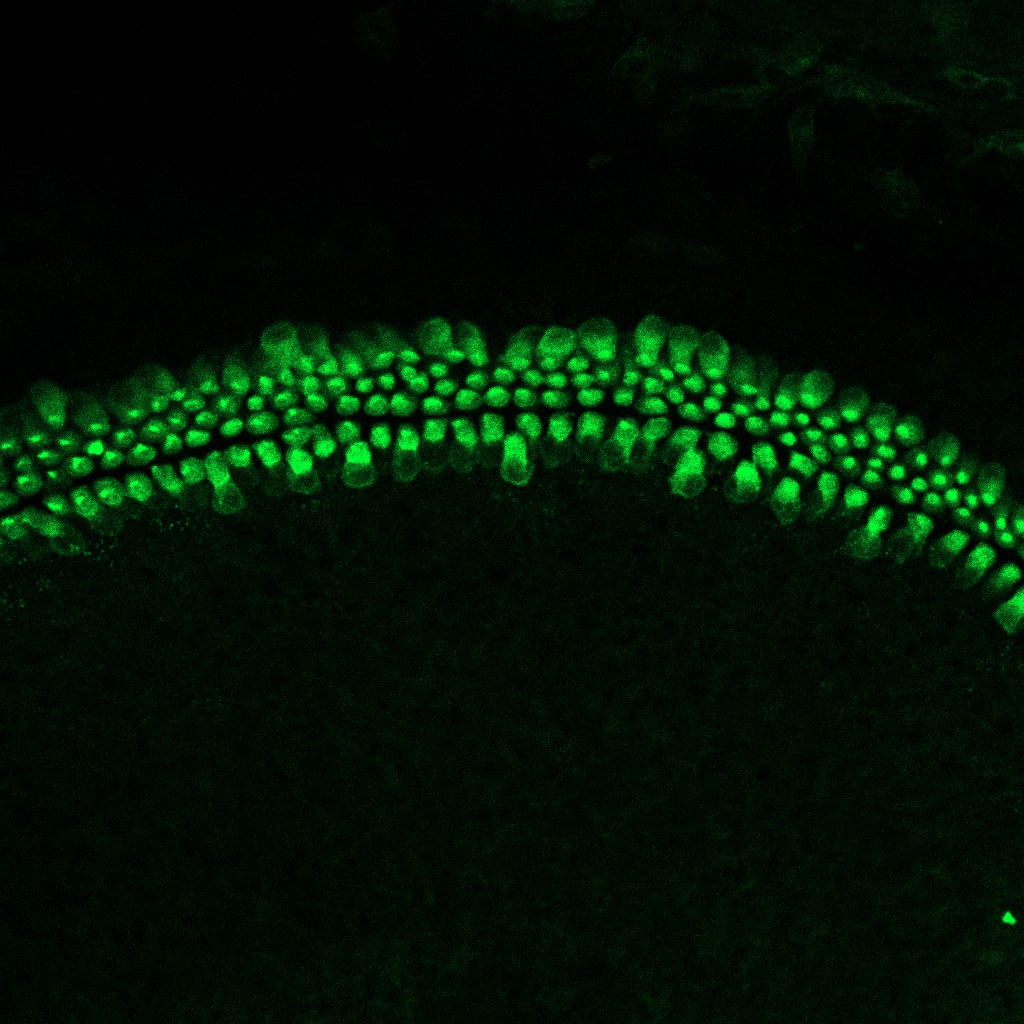

Supplement: Supplementary file 2 [file Data_Sheet_2.ZIP › Original data Fig. 1-3/Fig. 1/1.jpg]

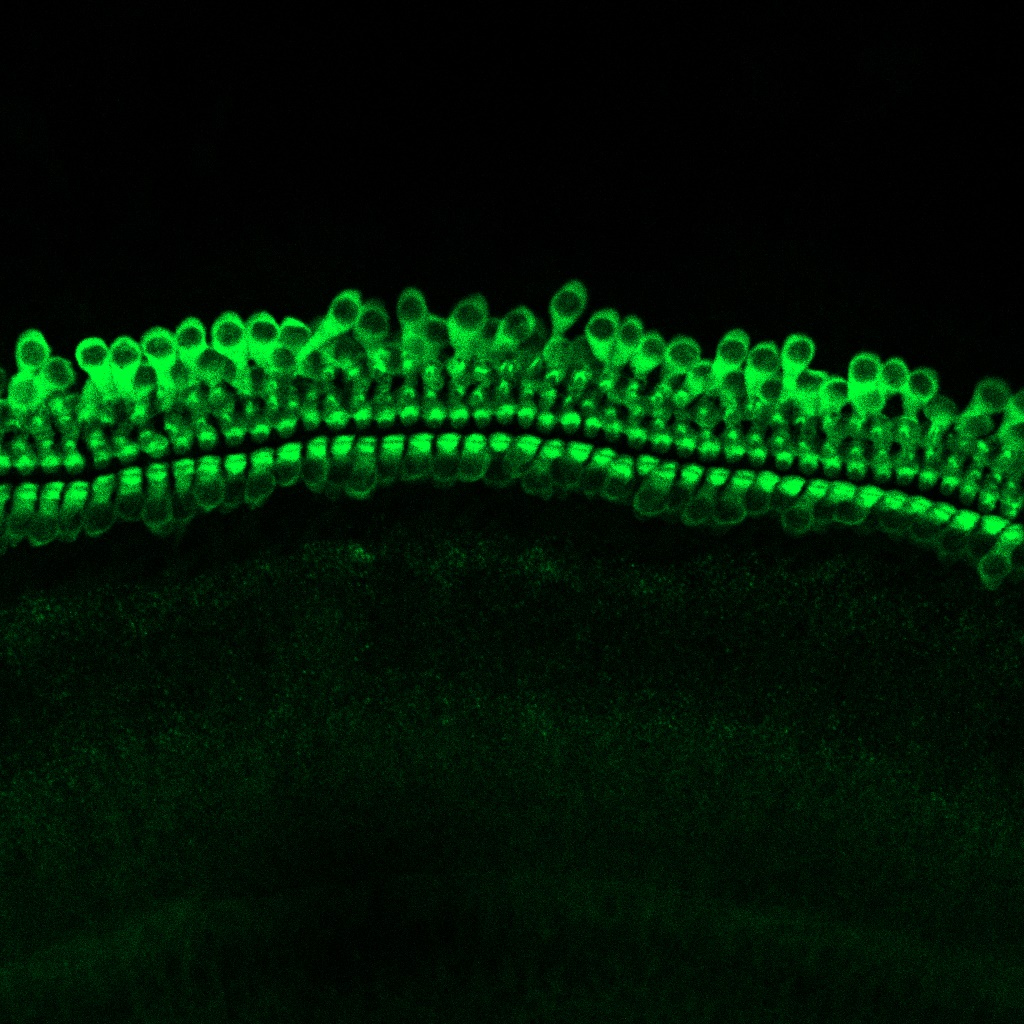

Supplement: Supplementary file 2 [file Data_Sheet_2.ZIP › Original data Fig. 1-3/Fig. 1/2.jpg]

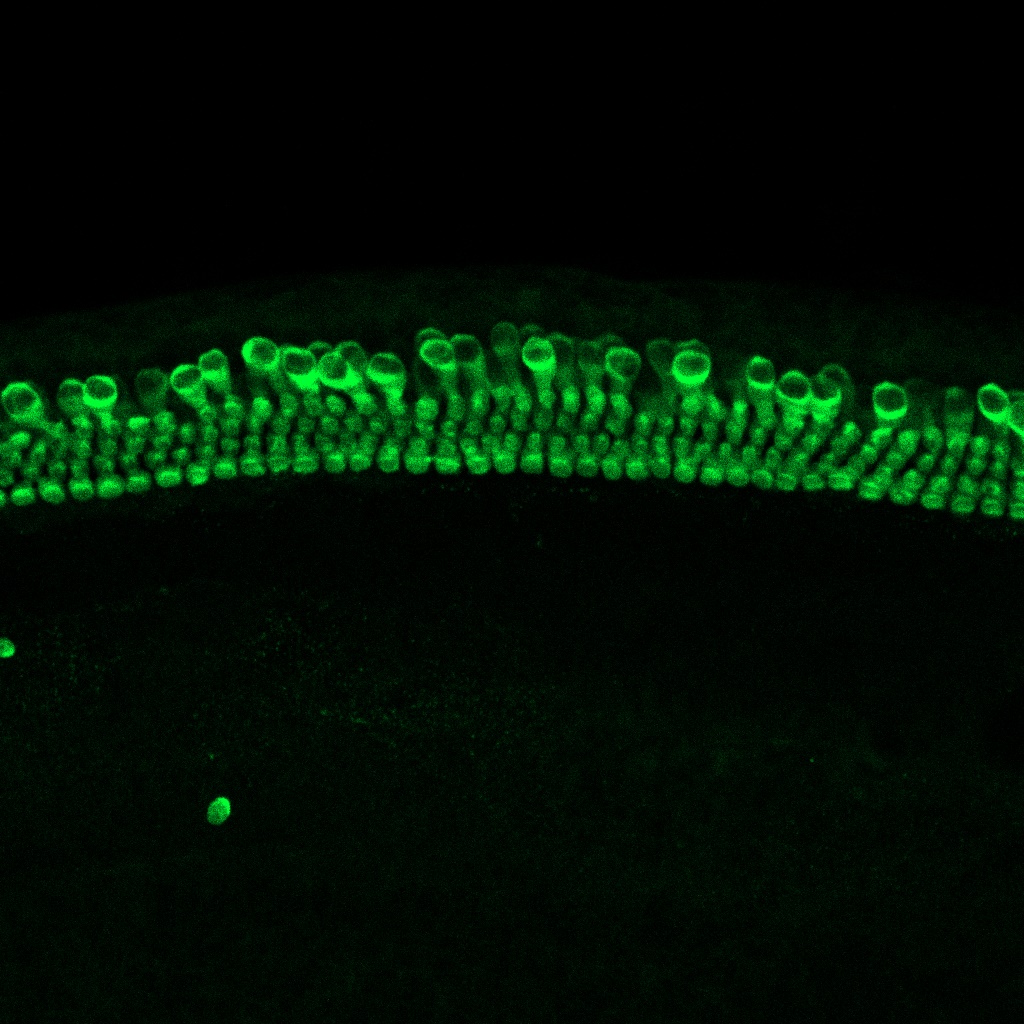

Supplement: Supplementary file 2 [file Data_Sheet_2.ZIP › Original data Fig. 1-3/Fig. 1/3.jpg]

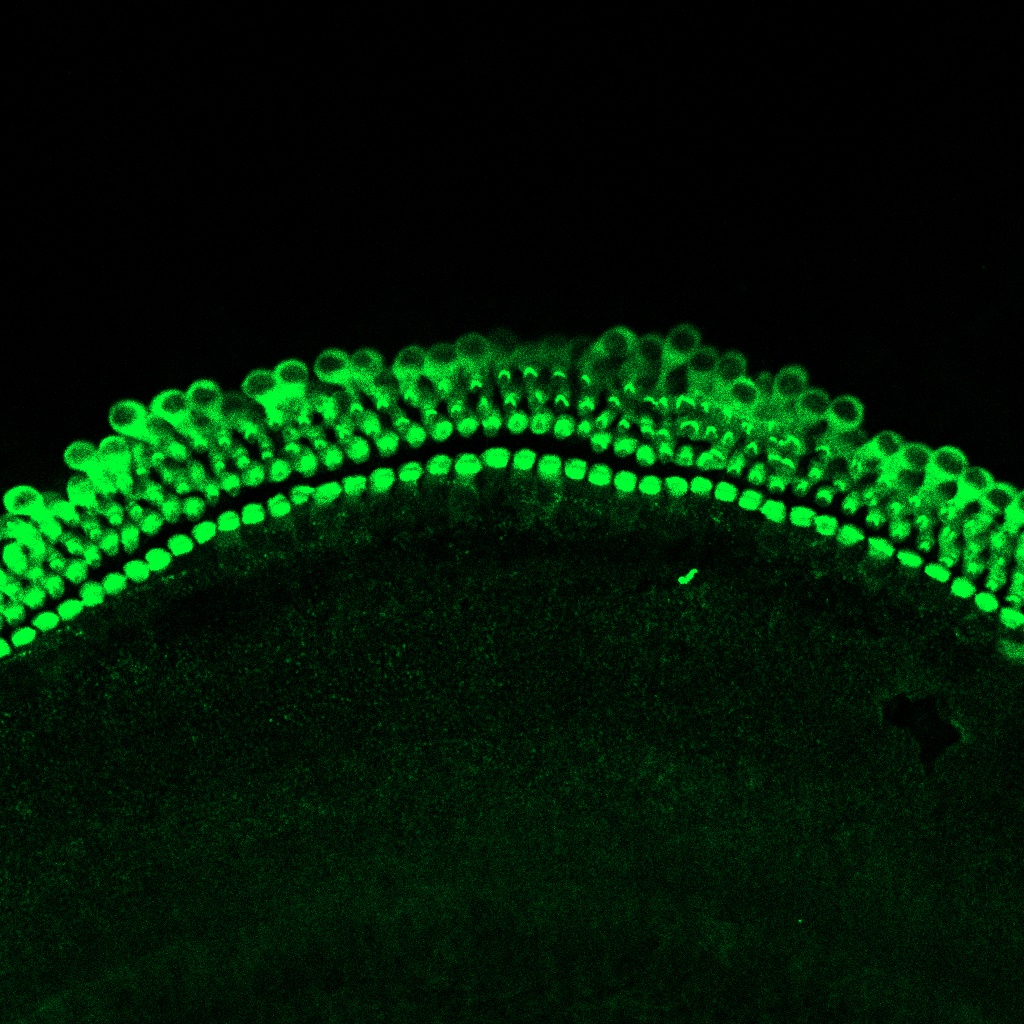

Supplement: Supplementary file 2 [file Data_Sheet_2.ZIP › Original data Fig. 1-3/Fig. 1/4.jpg]

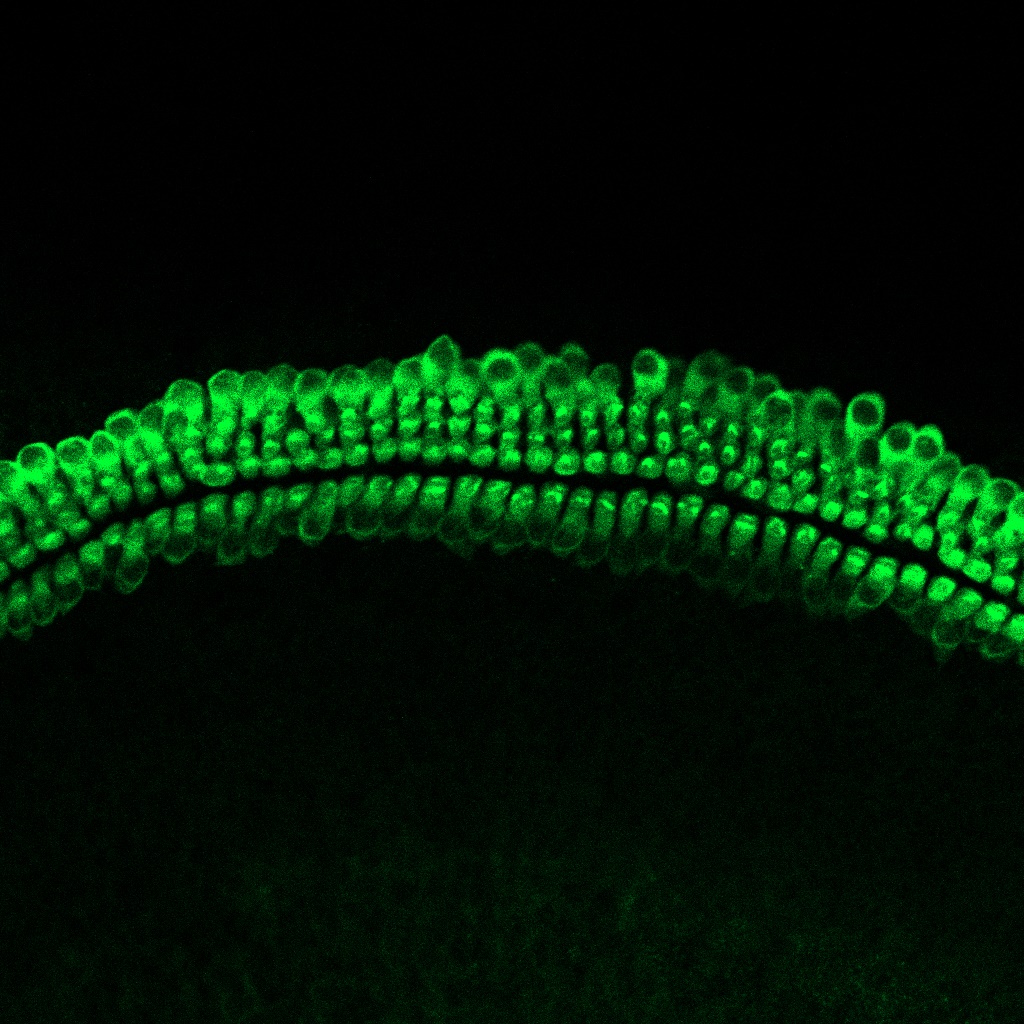

Supplement: Supplementary file 2 [file Data_Sheet_2.ZIP › Original data Fig. 1-3/Fig. 1/5.jpg]

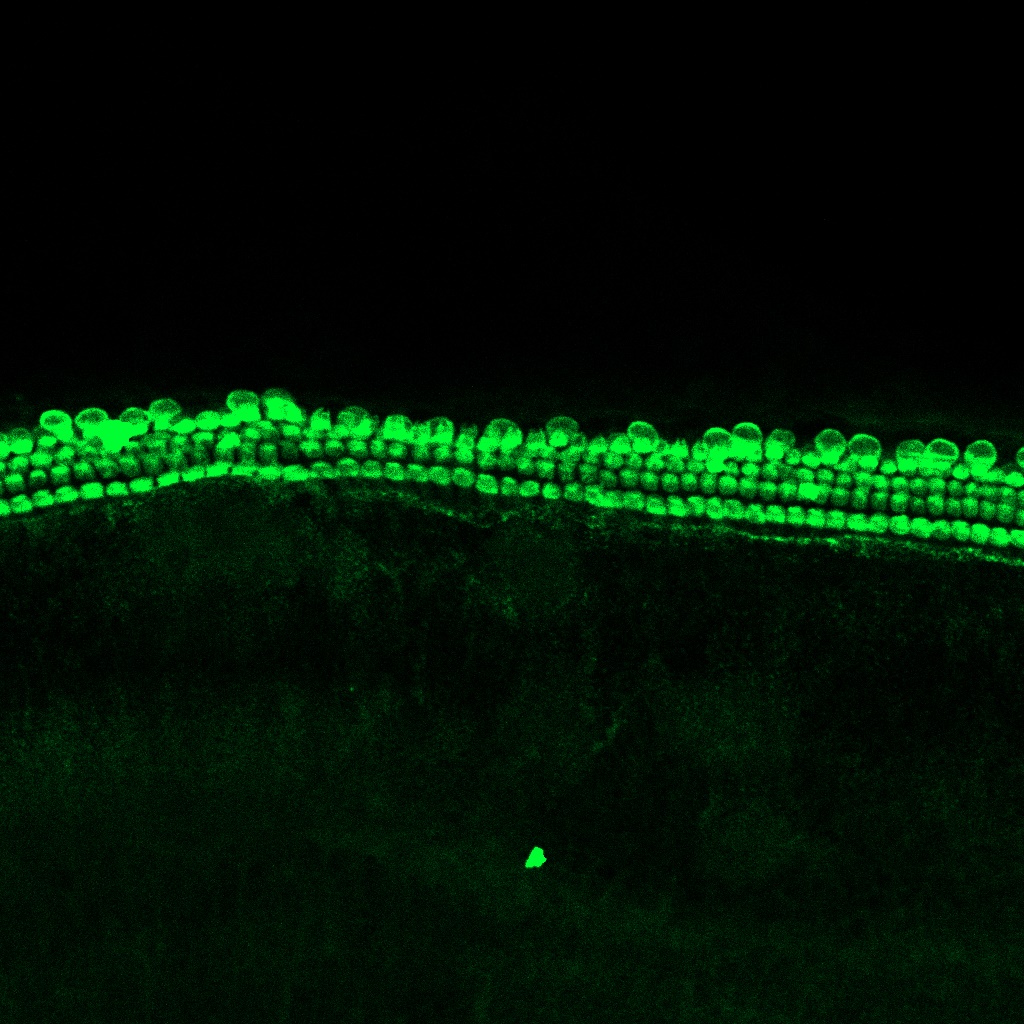

Supplement: Supplementary file 2 [file Data_Sheet_2.ZIP › Original data Fig. 1-3/Fig. 1/6.jpg]

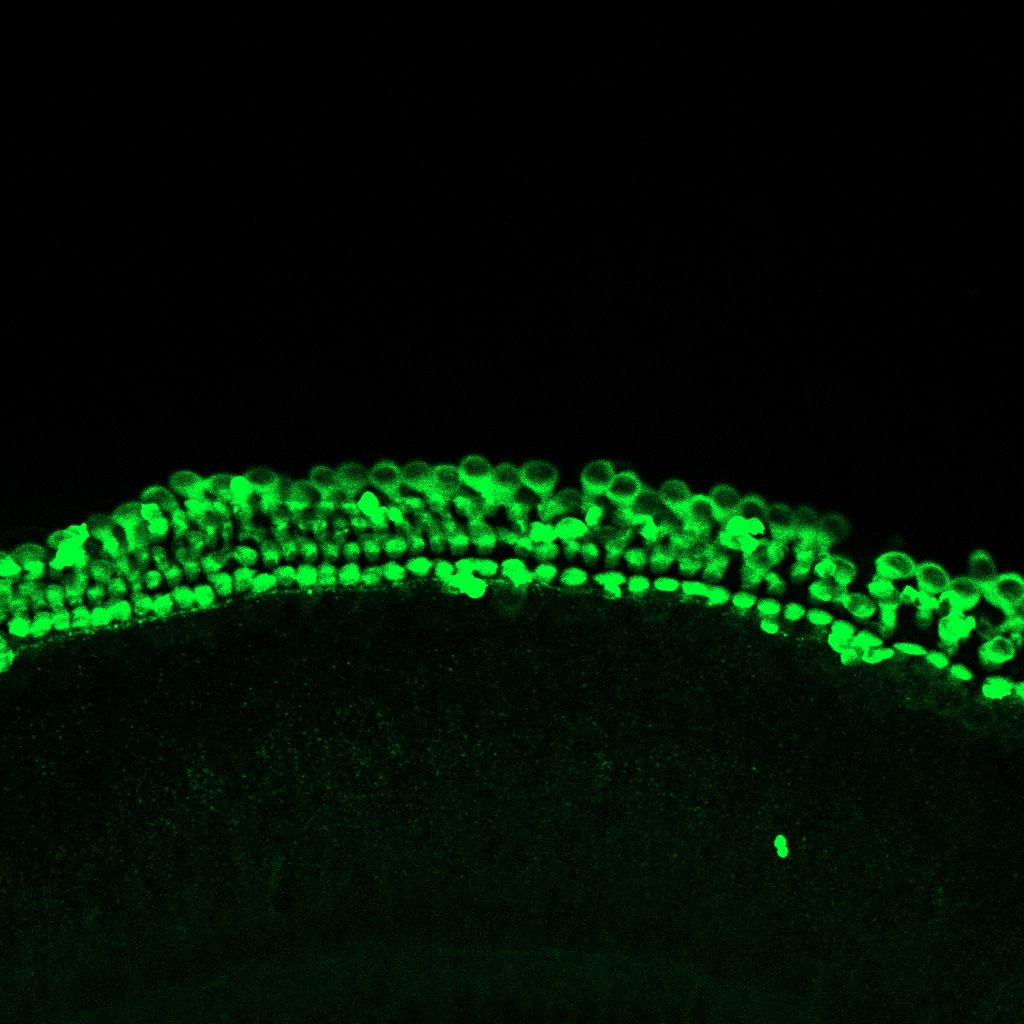

Supplement: Supplementary file 2 [file Data_Sheet_2.ZIP › Original data Fig. 1-3/Fig. 1/7.jpg]

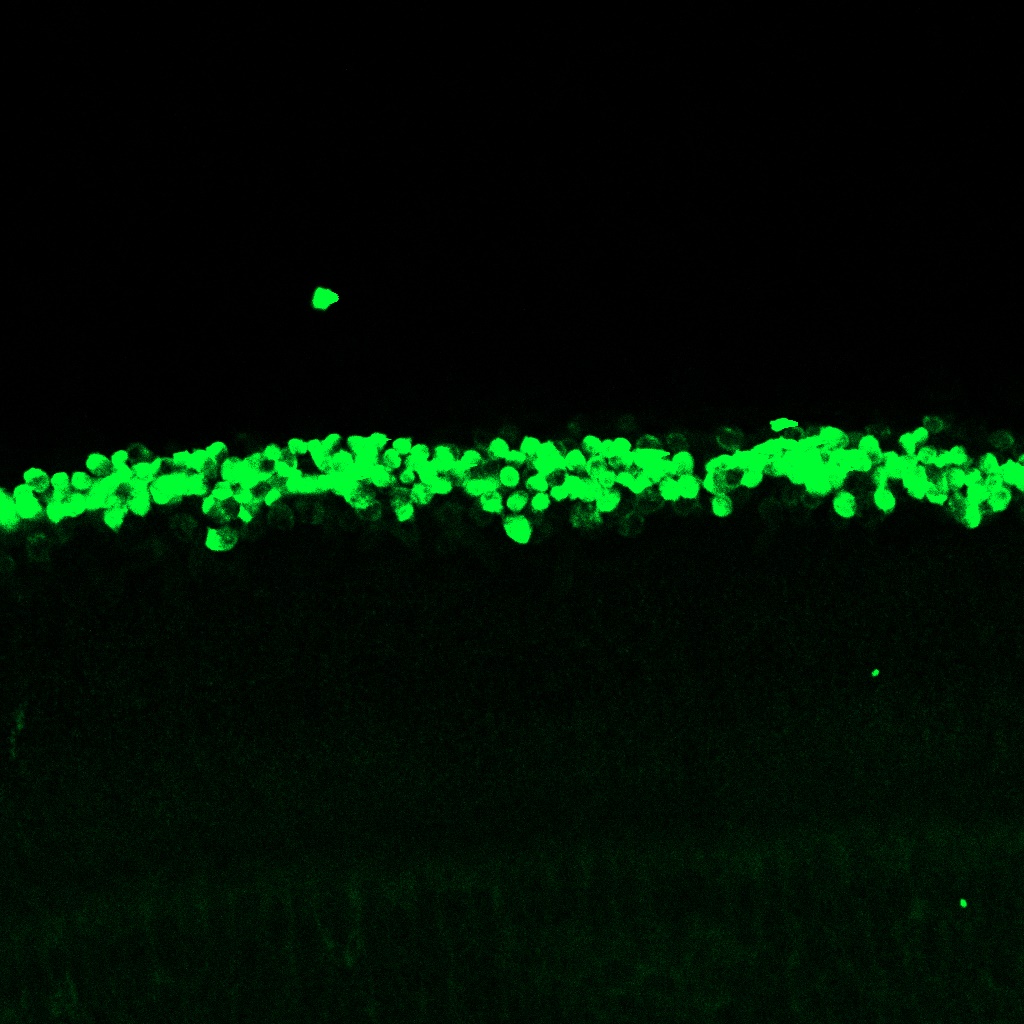

Supplement: Supplementary file 2 [file Data_Sheet_2.ZIP › Original data Fig. 1-3/Fig. 1/8.jpg]

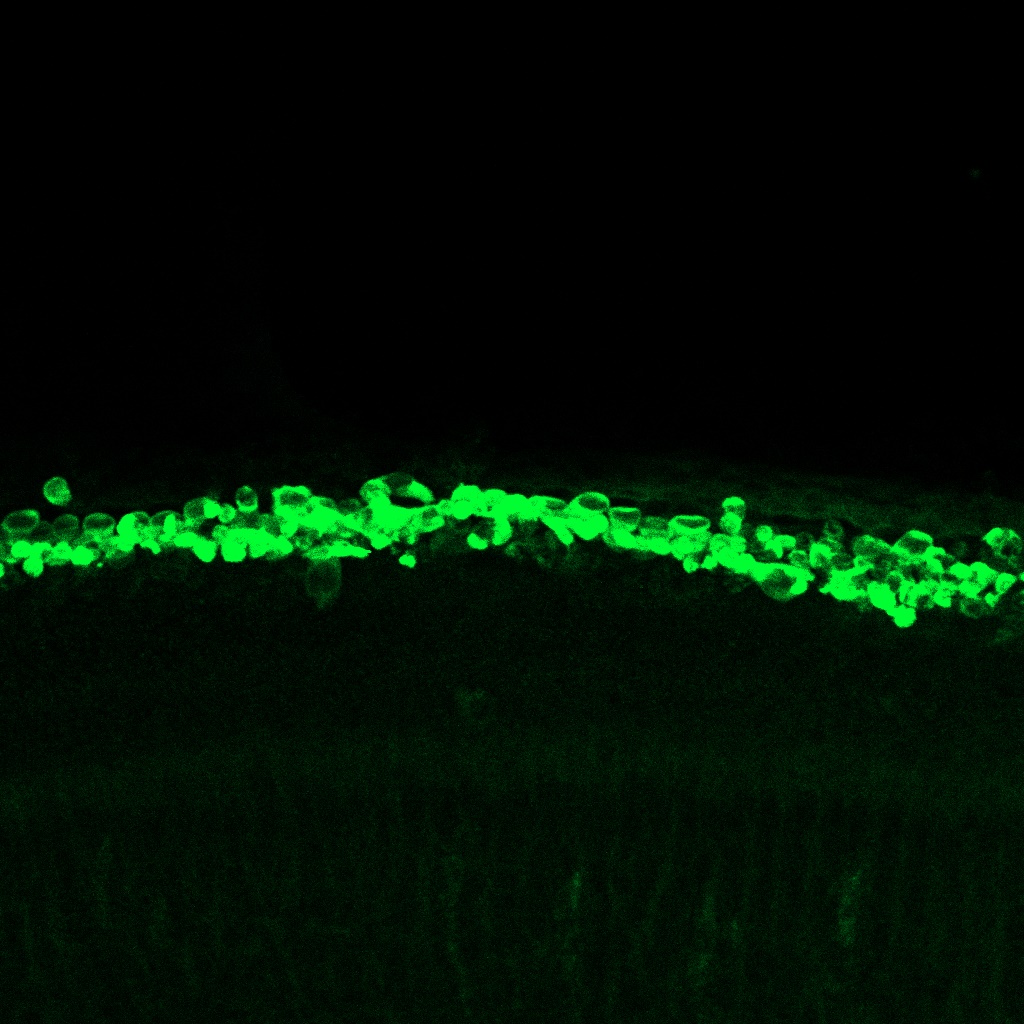

Supplement: Supplementary file 2 [file Data_Sheet_2.ZIP › Original data Fig. 1-3/Fig. 1/9.jpg]

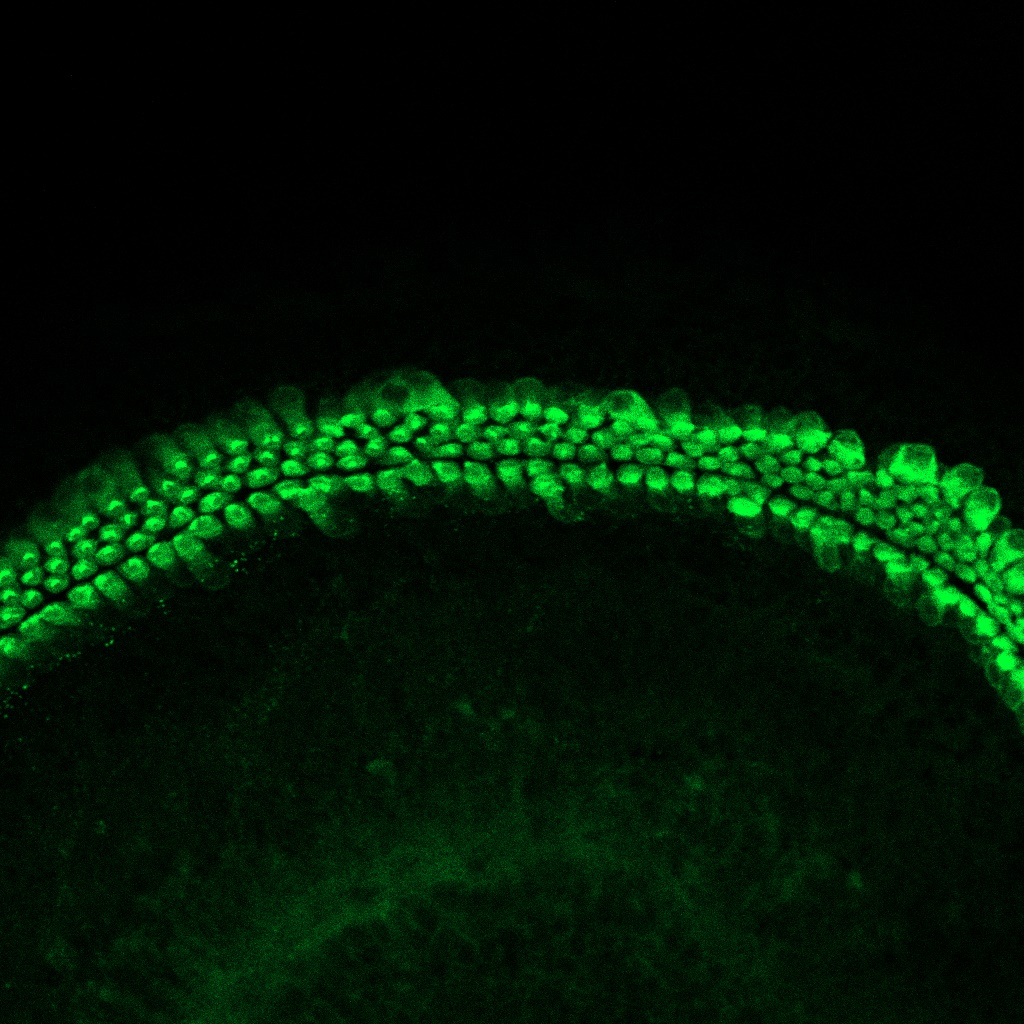

Supplement: Supplementary file 2 [file Data_Sheet_2.ZIP › Original data Fig. 1-3/Fig. 1/10.jpg]

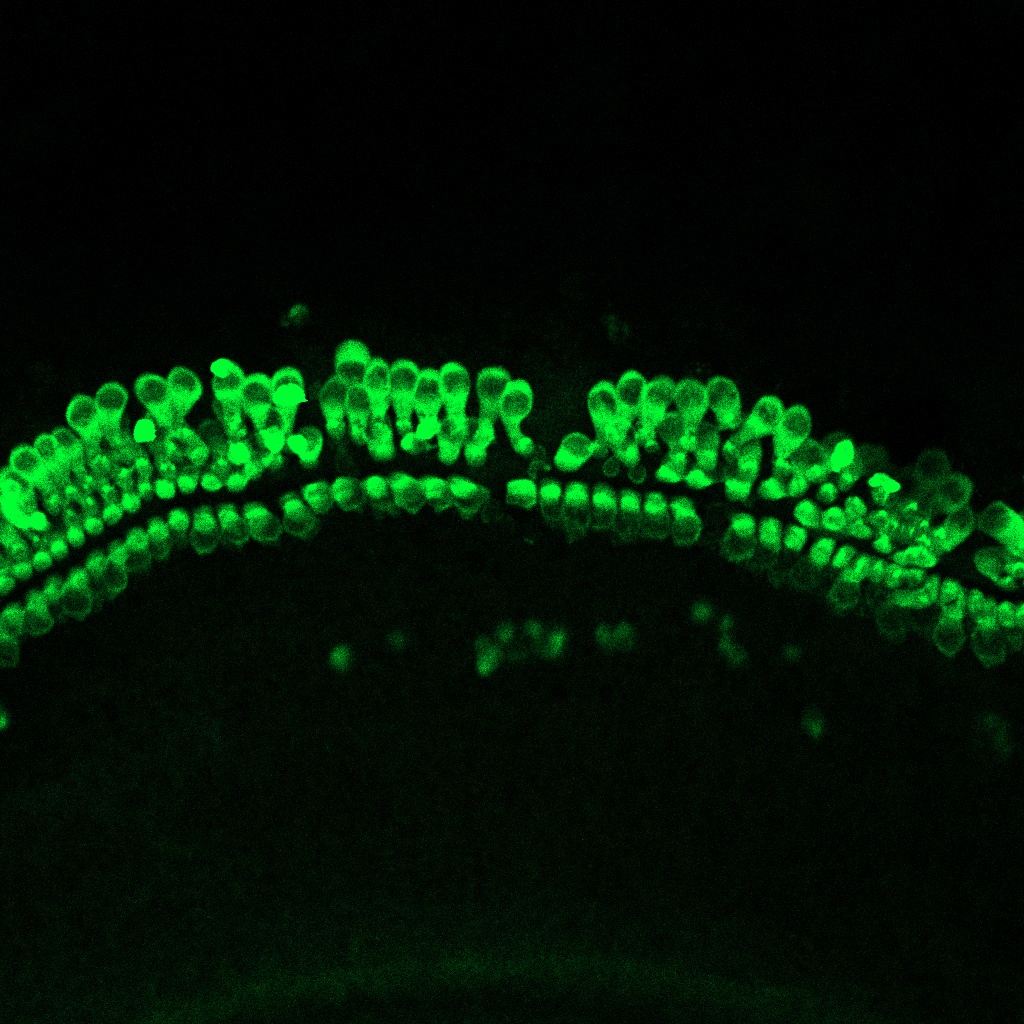

Supplement: Supplementary file 2 [file Data_Sheet_2.ZIP › Original data Fig. 1-3/Fig. 1/11.jpg]

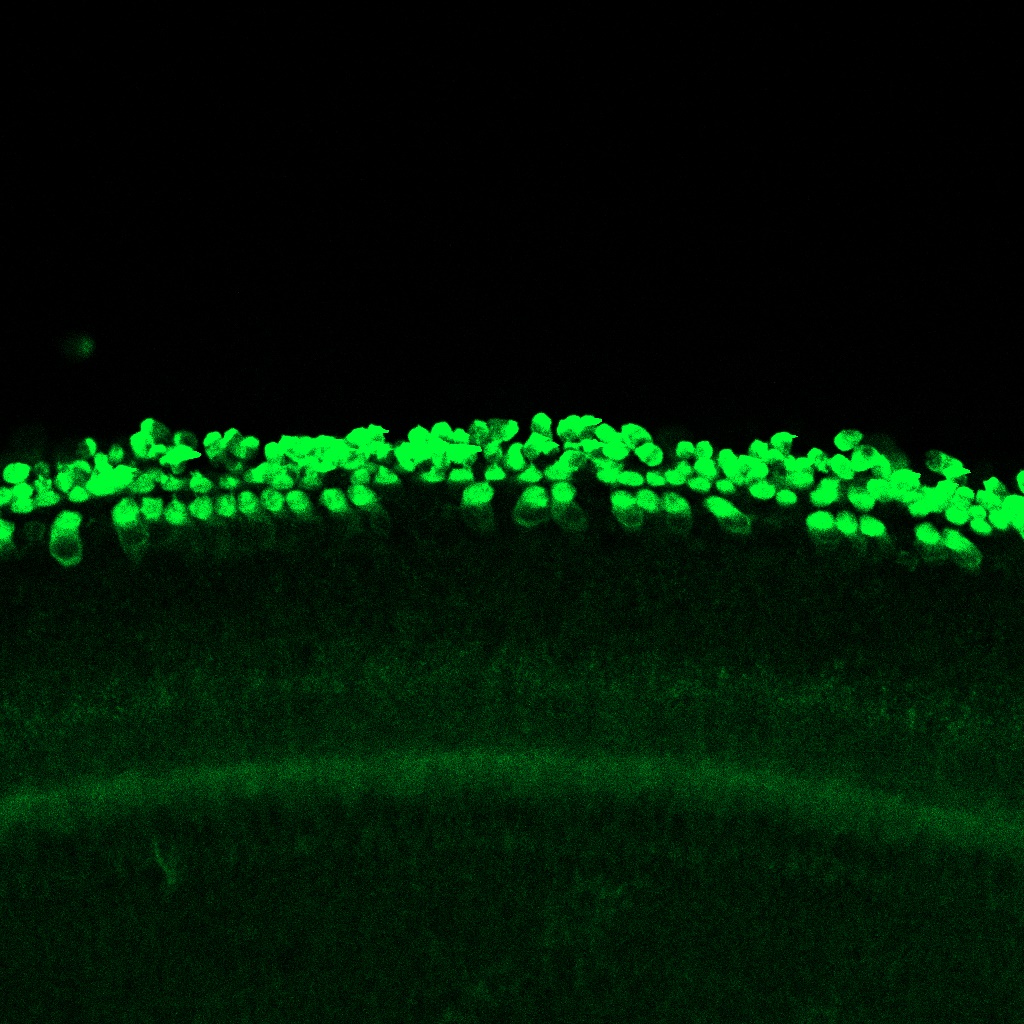

Supplement: Supplementary file 2 [file Data_Sheet_2.ZIP › Original data Fig. 1-3/Fig. 1/12.jpg]

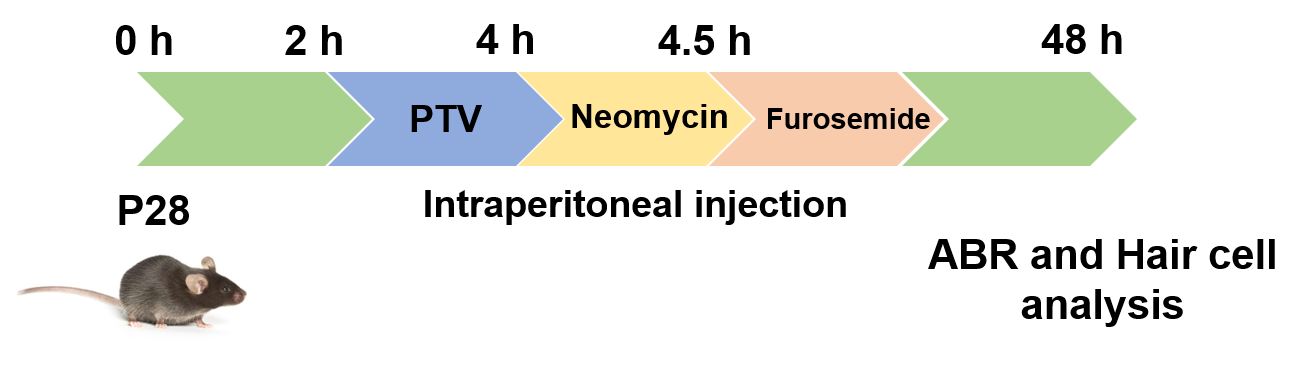

Supplement: Supplementary file 2 [file Data_Sheet_2.ZIP › Original data Fig. 1-3/Fig. 2/Fig. 2A.tif]

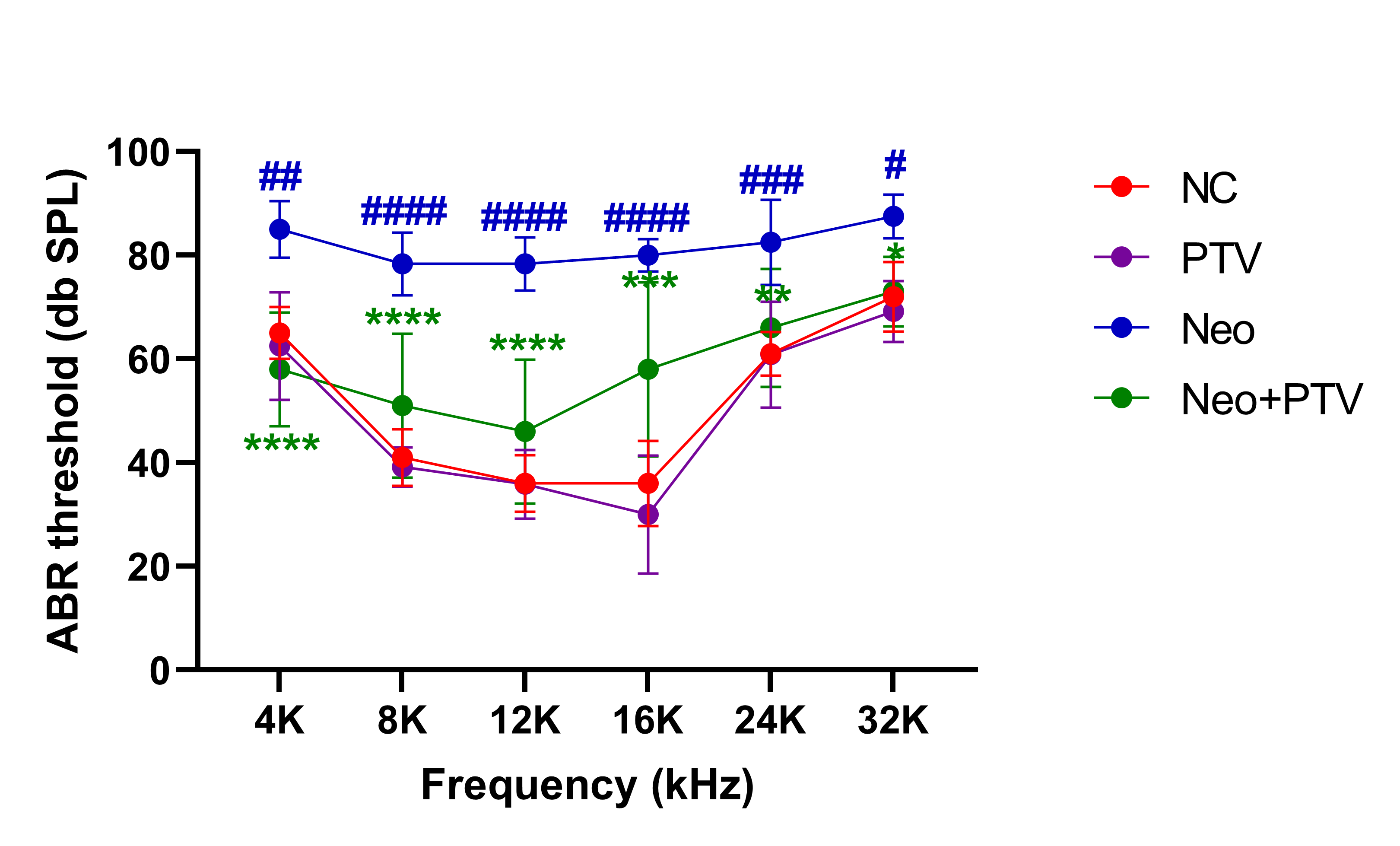

Supplement: Supplementary file 2 [file Data_Sheet_2.ZIP › Original data Fig. 1-3/Fig. 2/Fig. 2B.tif]

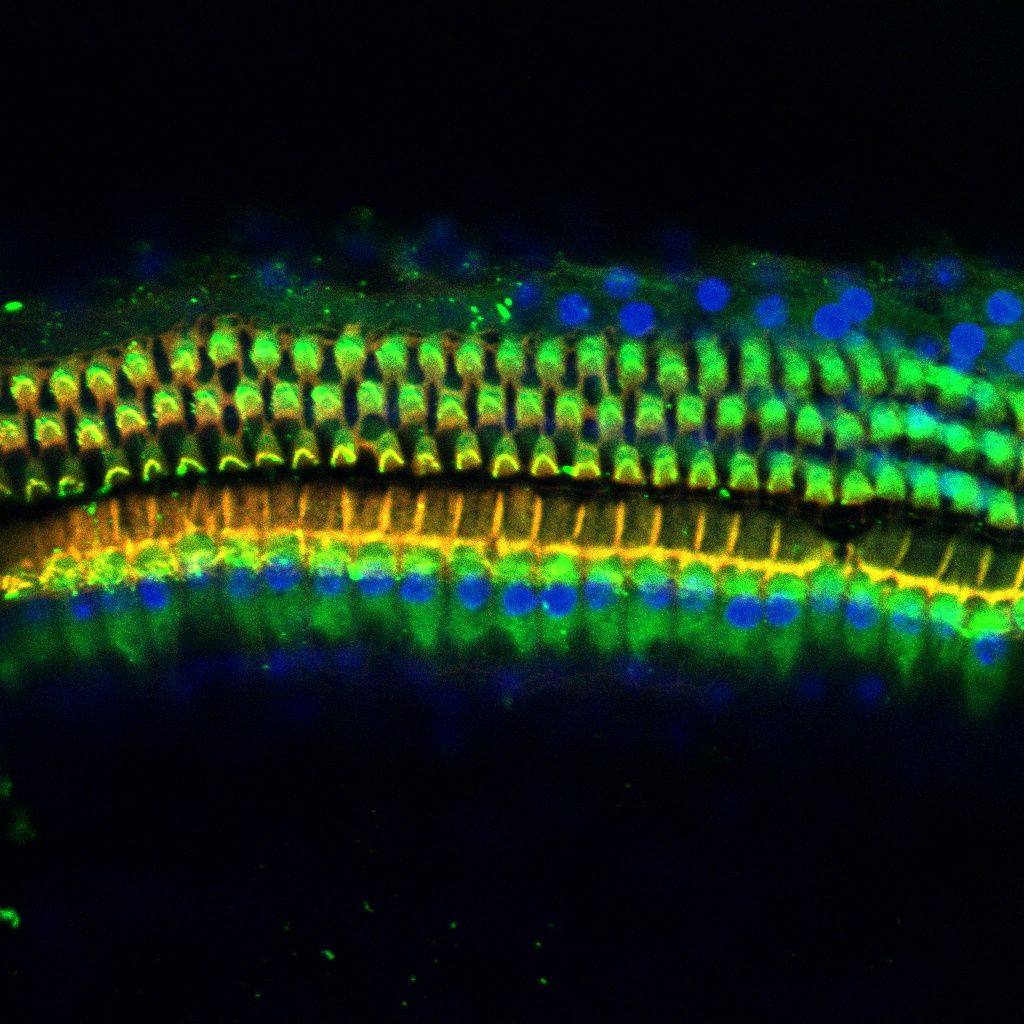

Supplement: Supplementary file 2 [file Data_Sheet_2.ZIP › Original data Fig. 1-3/Fig. 2/1.jpg]

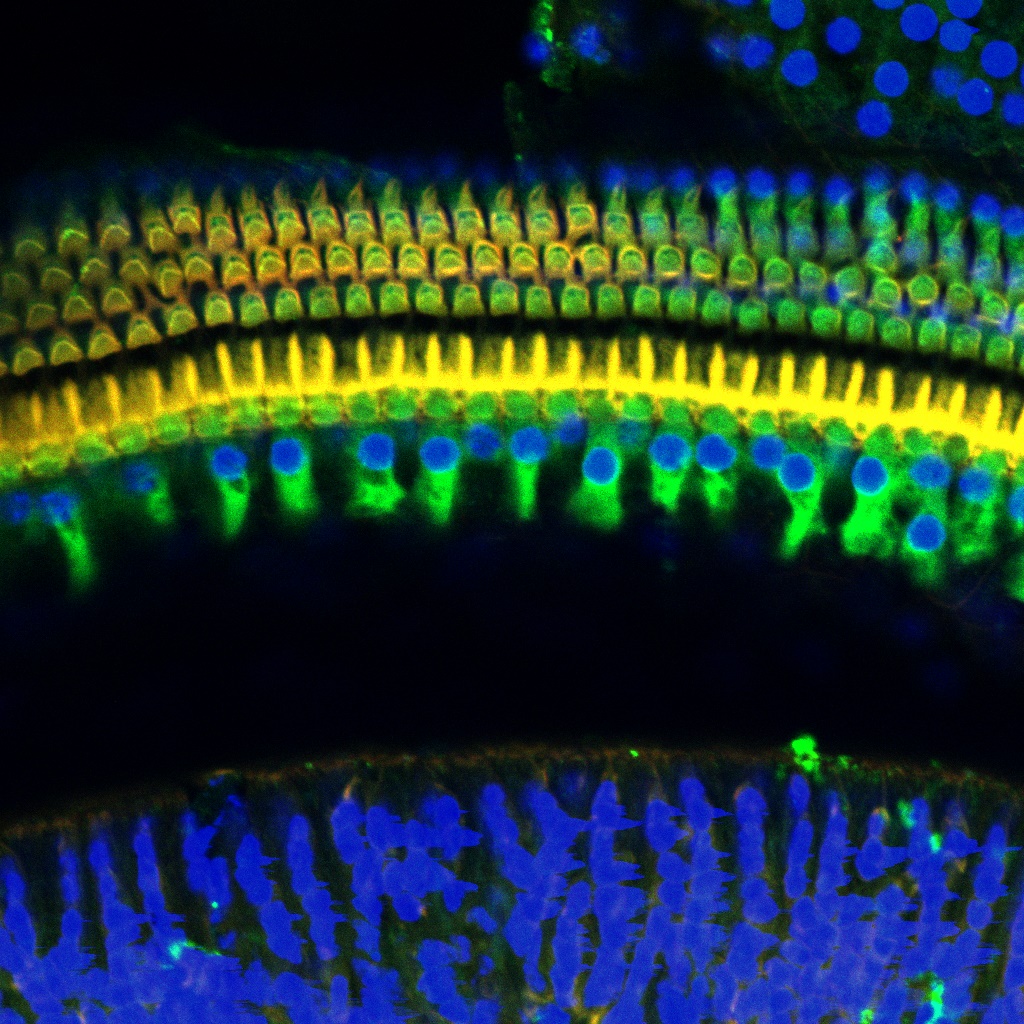

Supplement: Supplementary file 2 [file Data_Sheet_2.ZIP › Original data Fig. 1-3/Fig. 2/2.jpg]

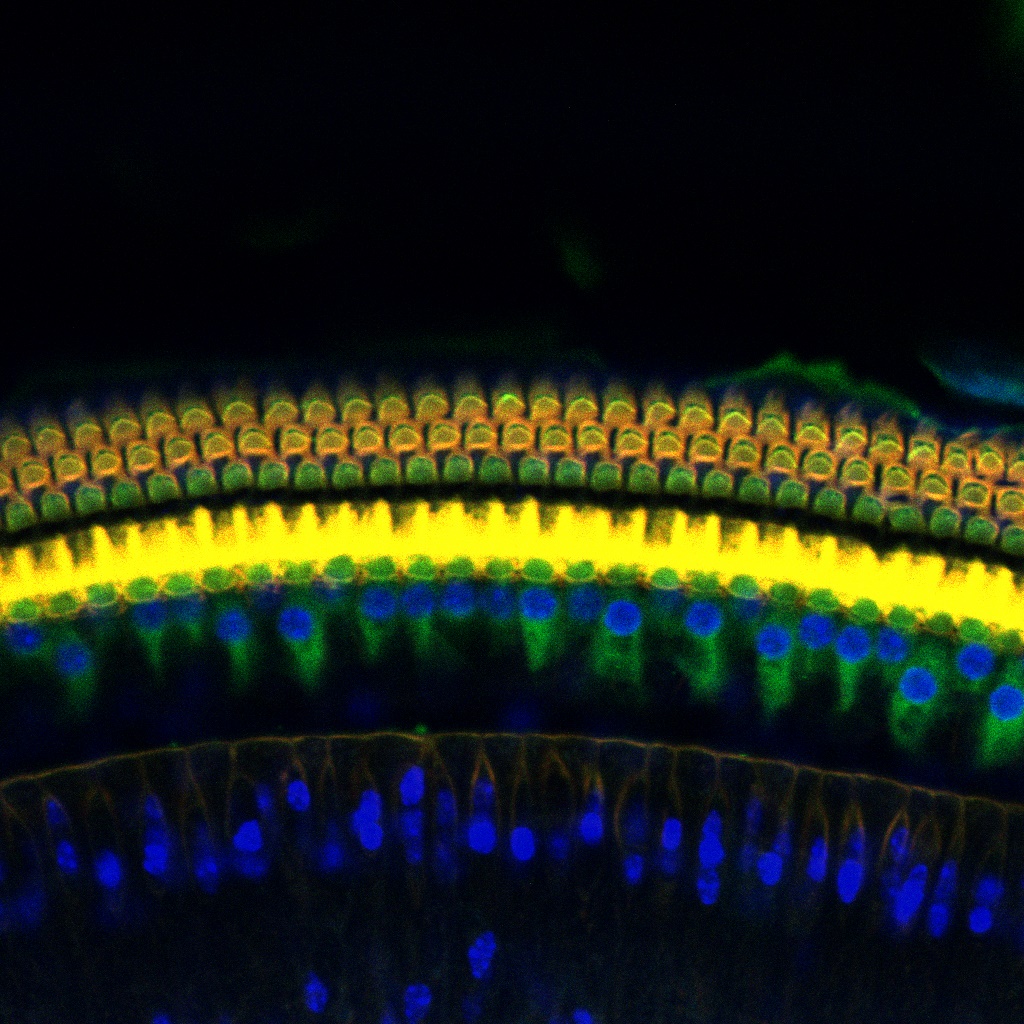

Supplement: Supplementary file 2 [file Data_Sheet_2.ZIP › Original data Fig. 1-3/Fig. 2/3.jpg]

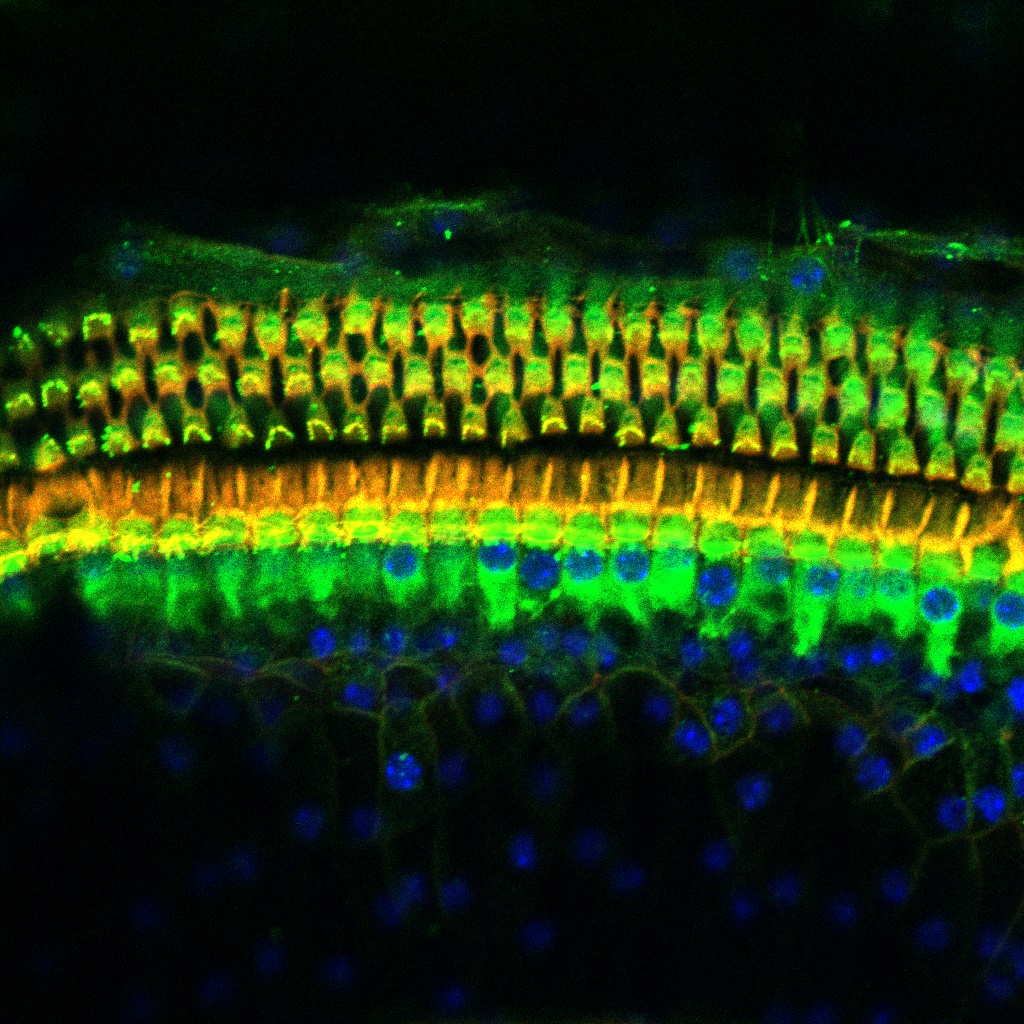

Supplement: Supplementary file 2 [file Data_Sheet_2.ZIP › Original data Fig. 1-3/Fig. 2/4.jpg]

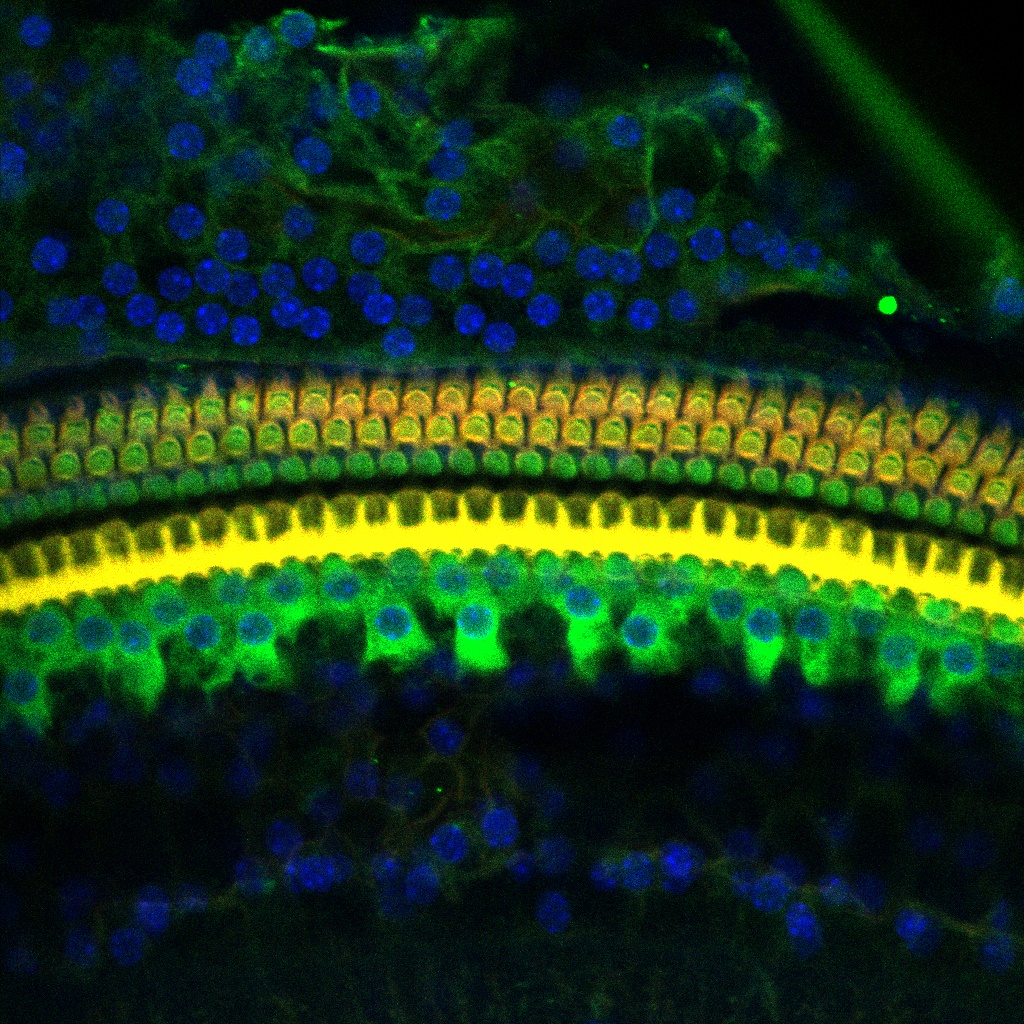

Supplement: Supplementary file 2 [file Data_Sheet_2.ZIP › Original data Fig. 1-3/Fig. 2/6.jpg]

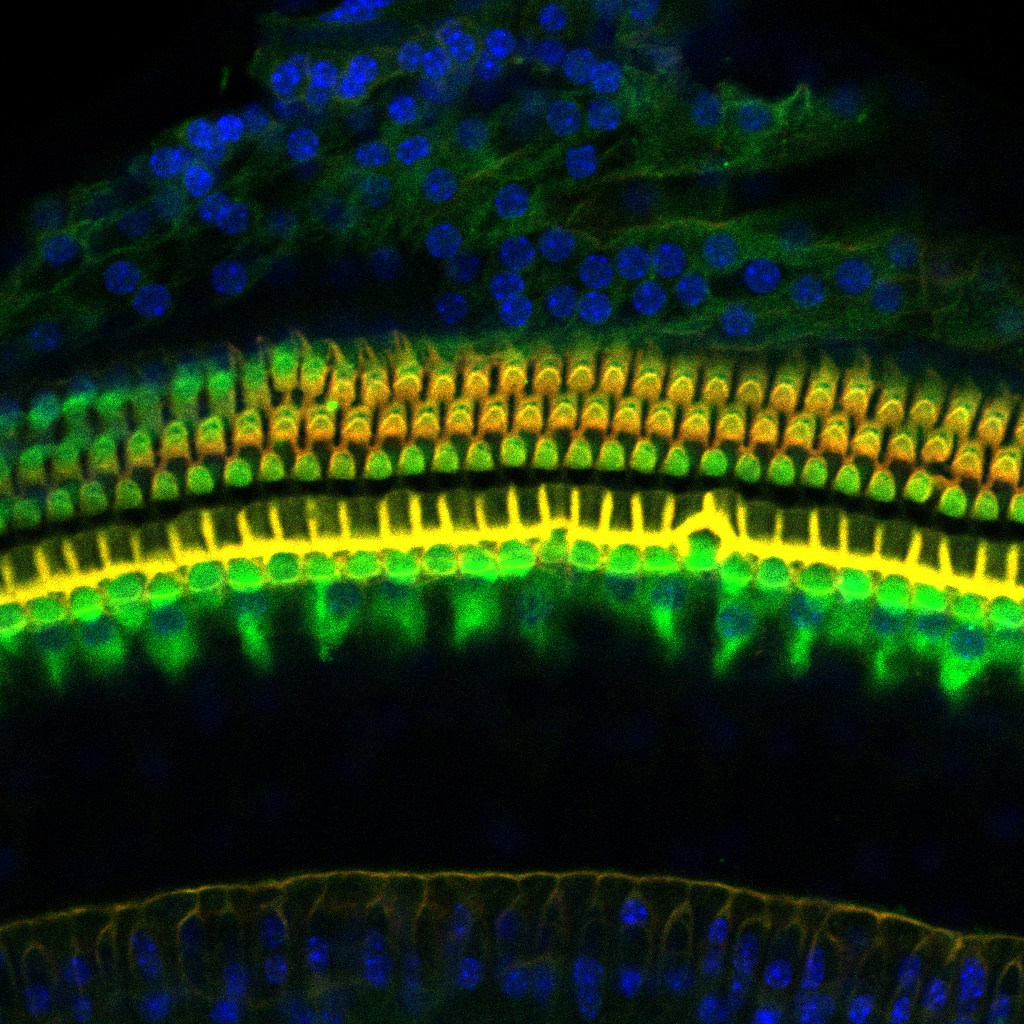

Supplement: Supplementary file 2 [file Data_Sheet_2.ZIP › Original data Fig. 1-3/Fig. 2/5.jpg]

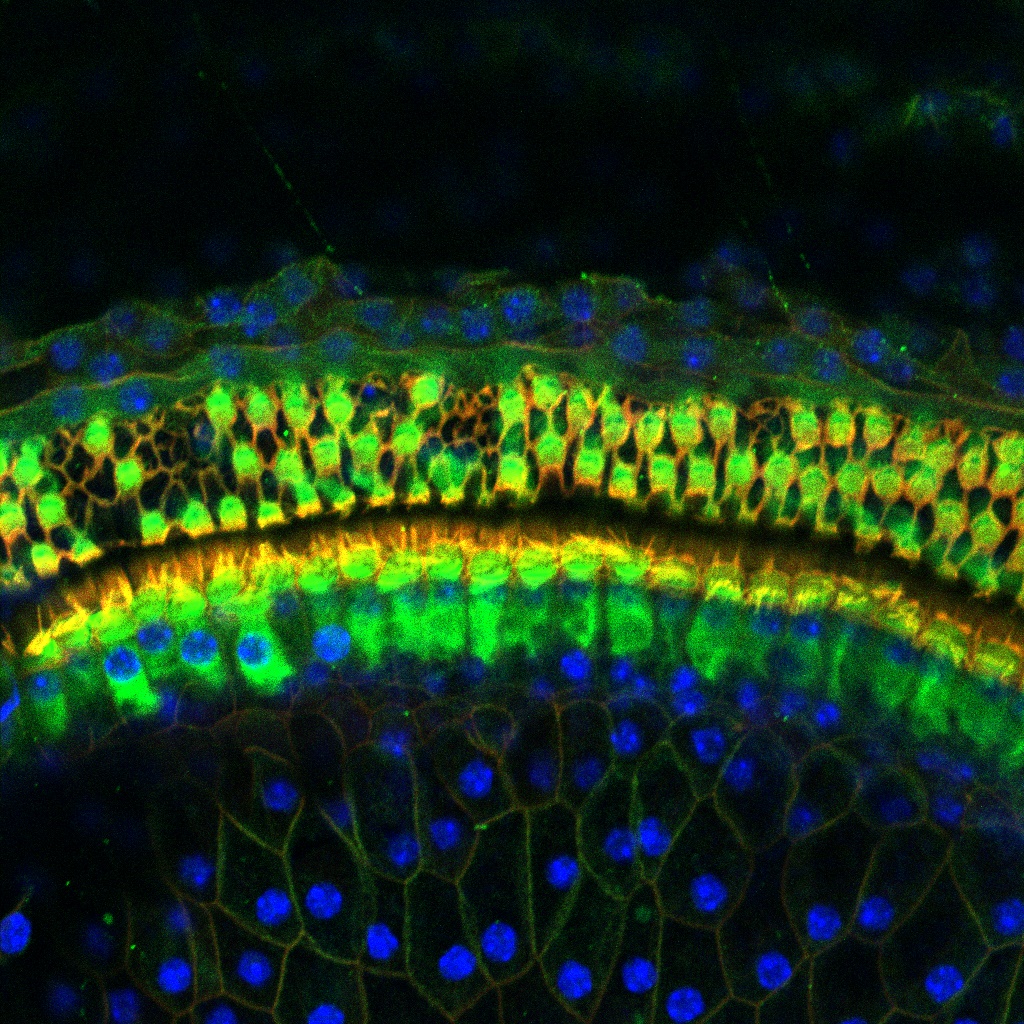

Supplement: Supplementary file 2 [file Data_Sheet_2.ZIP › Original data Fig. 1-3/Fig. 2/7.jpg]

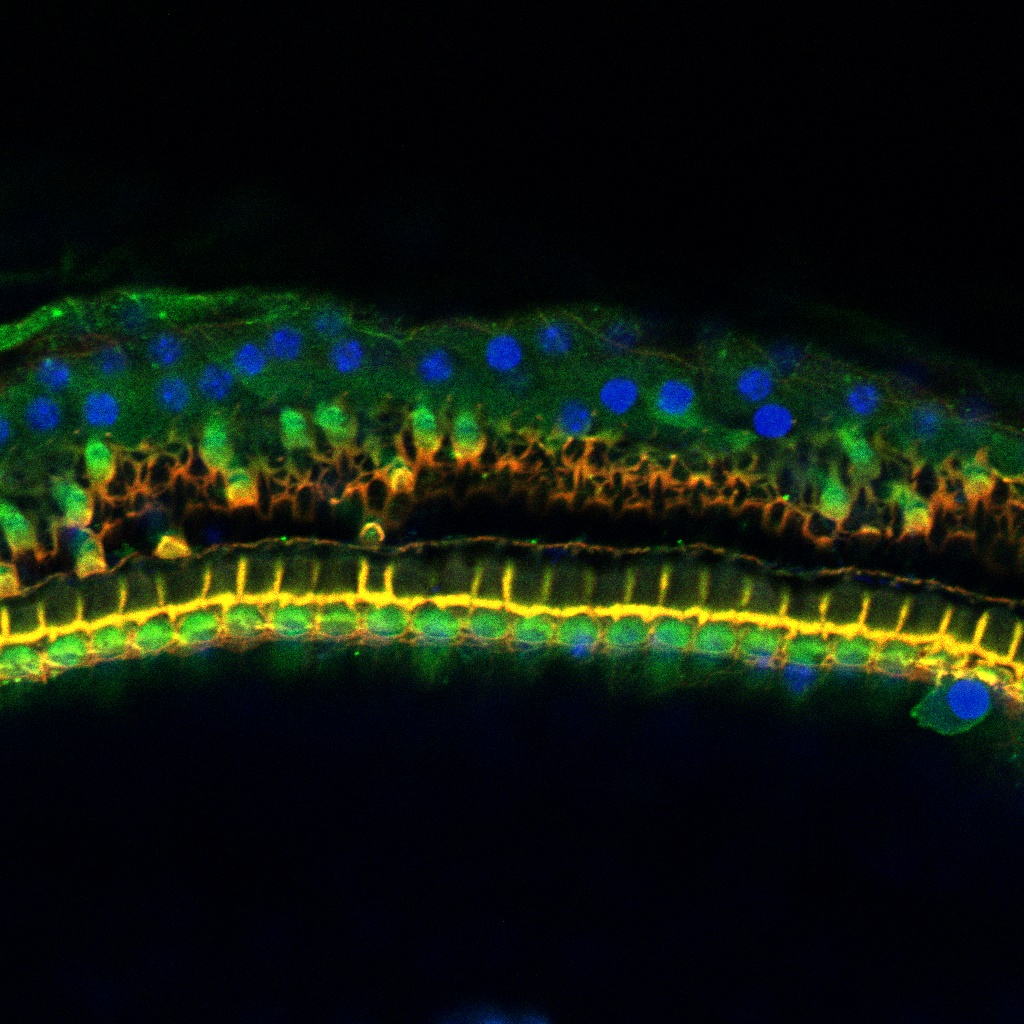

Supplement: Supplementary file 2 [file Data_Sheet_2.ZIP › Original data Fig. 1-3/Fig. 2/8.jpg]

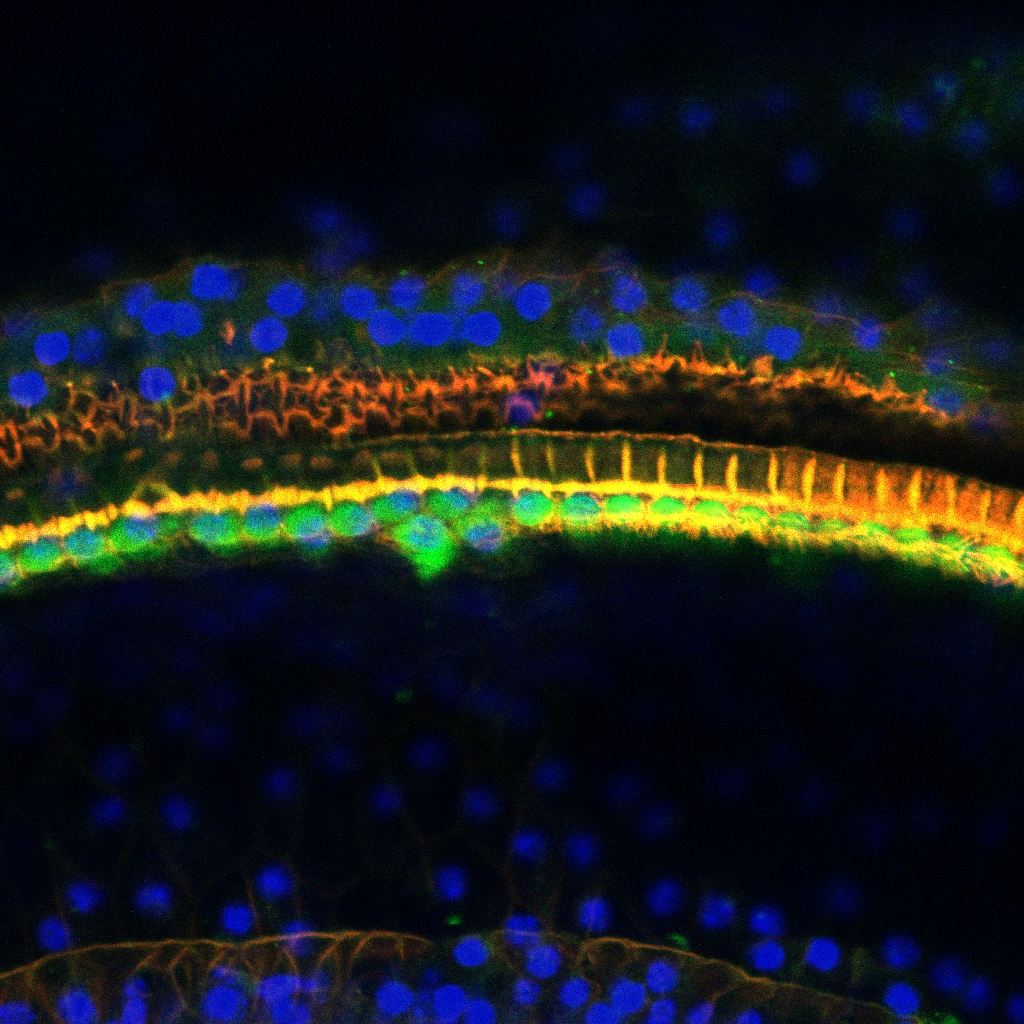

Supplement: Supplementary file 2 [file Data_Sheet_2.ZIP › Original data Fig. 1-3/Fig. 2/9.jpg]

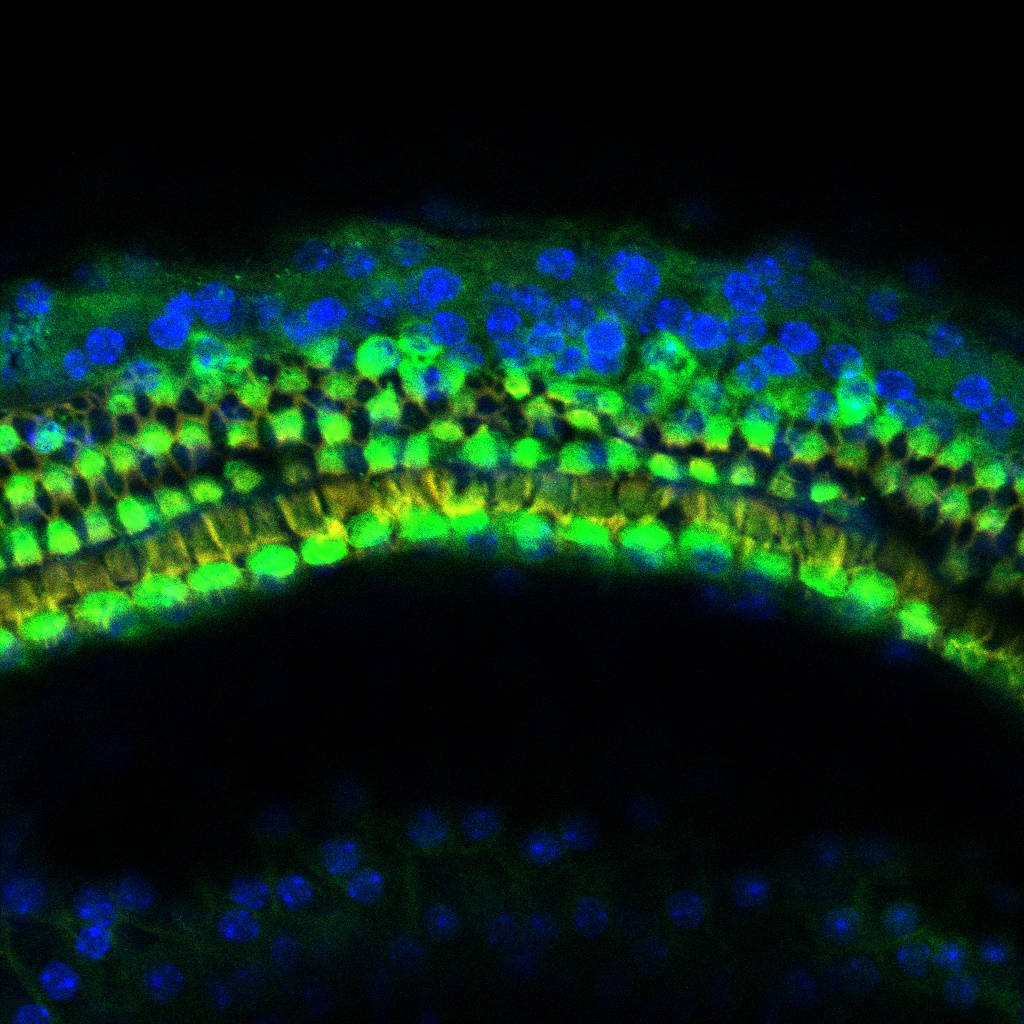

Supplement: Supplementary file 2 [file Data_Sheet_2.ZIP › Original data Fig. 1-3/Fig. 2/10.jpg]

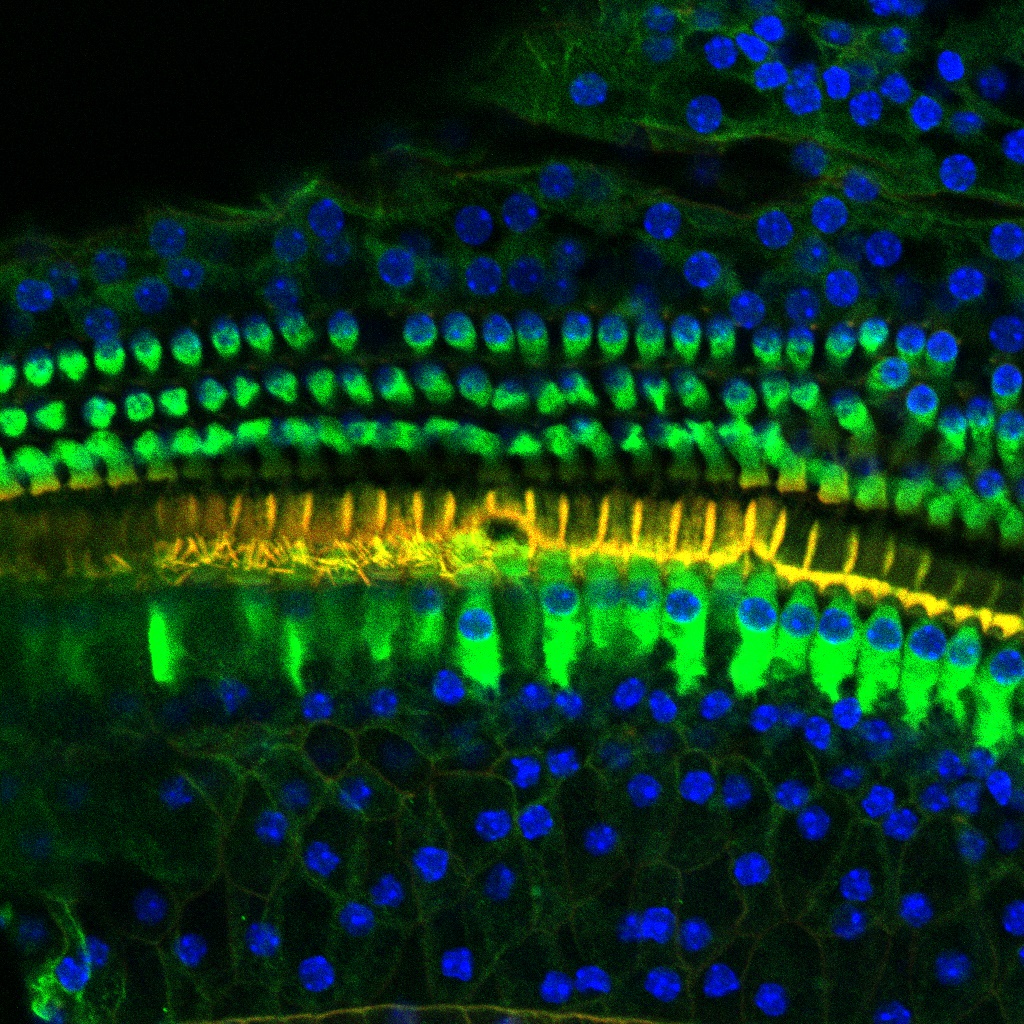

Supplement: Supplementary file 2 [file Data_Sheet_2.ZIP › Original data Fig. 1-3/Fig. 2/11.jpg]

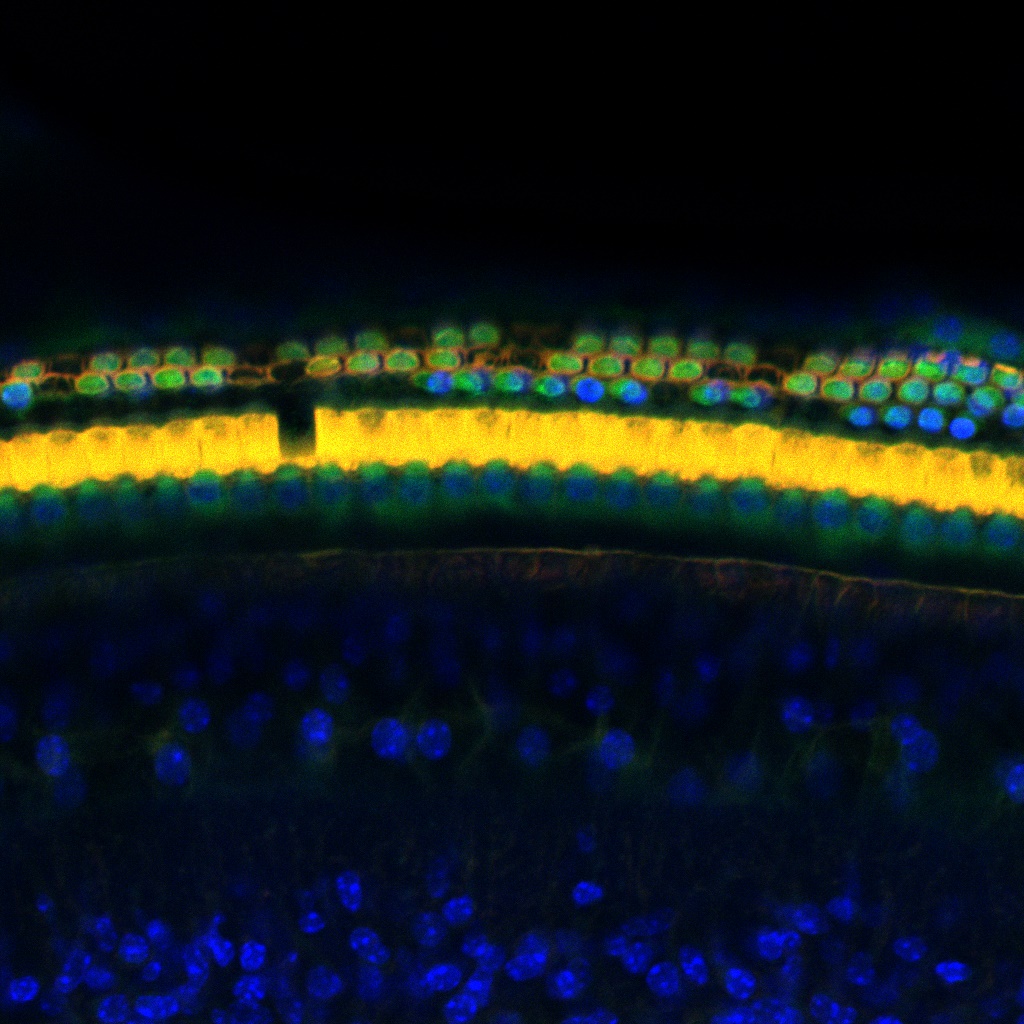

Supplement: Supplementary file 2 [file Data_Sheet_2.ZIP › Original data Fig. 1-3/Fig. 2/12.jpg]

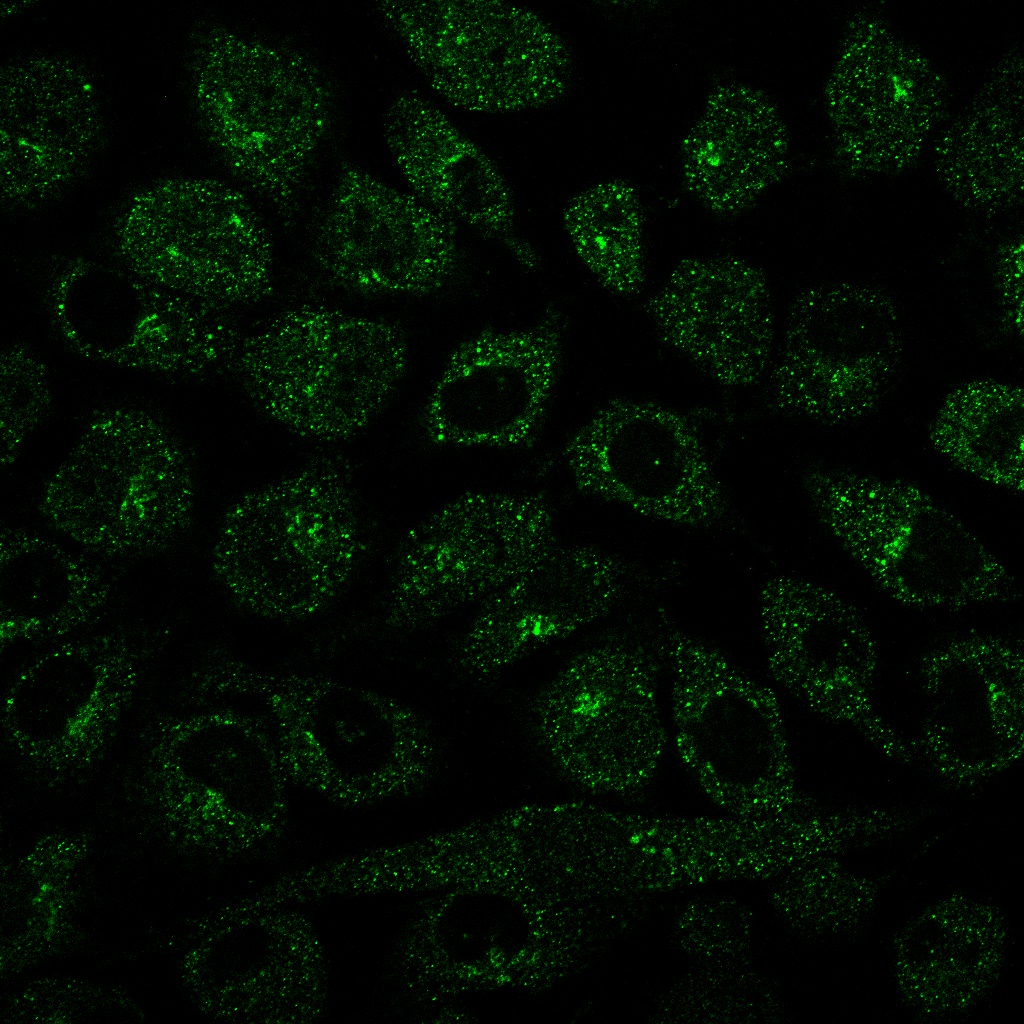

Supplement: Supplementary file 3 [file Data_Sheet_3.ZIP › Original data Fig. 4-7/Fig. 6/1.jpg]

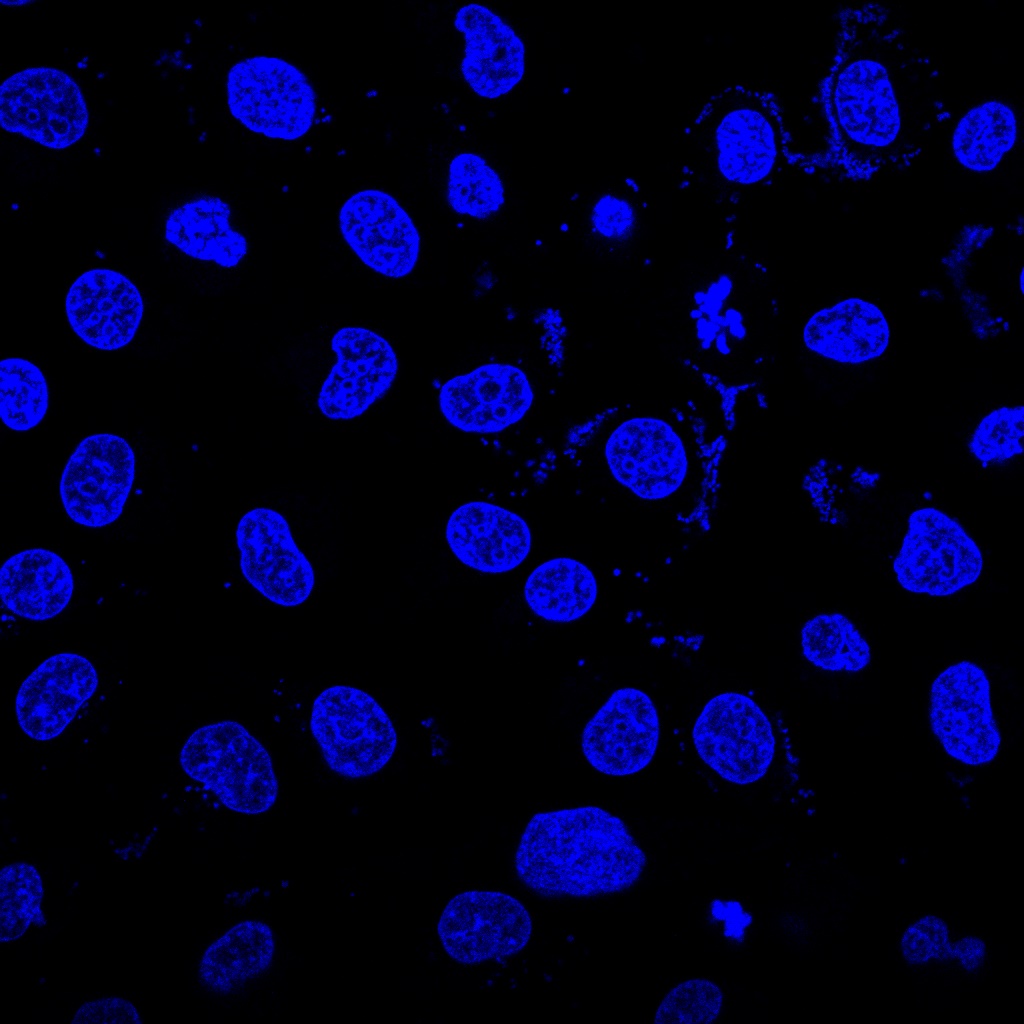

Supplement: Supplementary file 3 [file Data_Sheet_3.ZIP › Original data Fig. 4-7/Fig. 6/2.jpg]

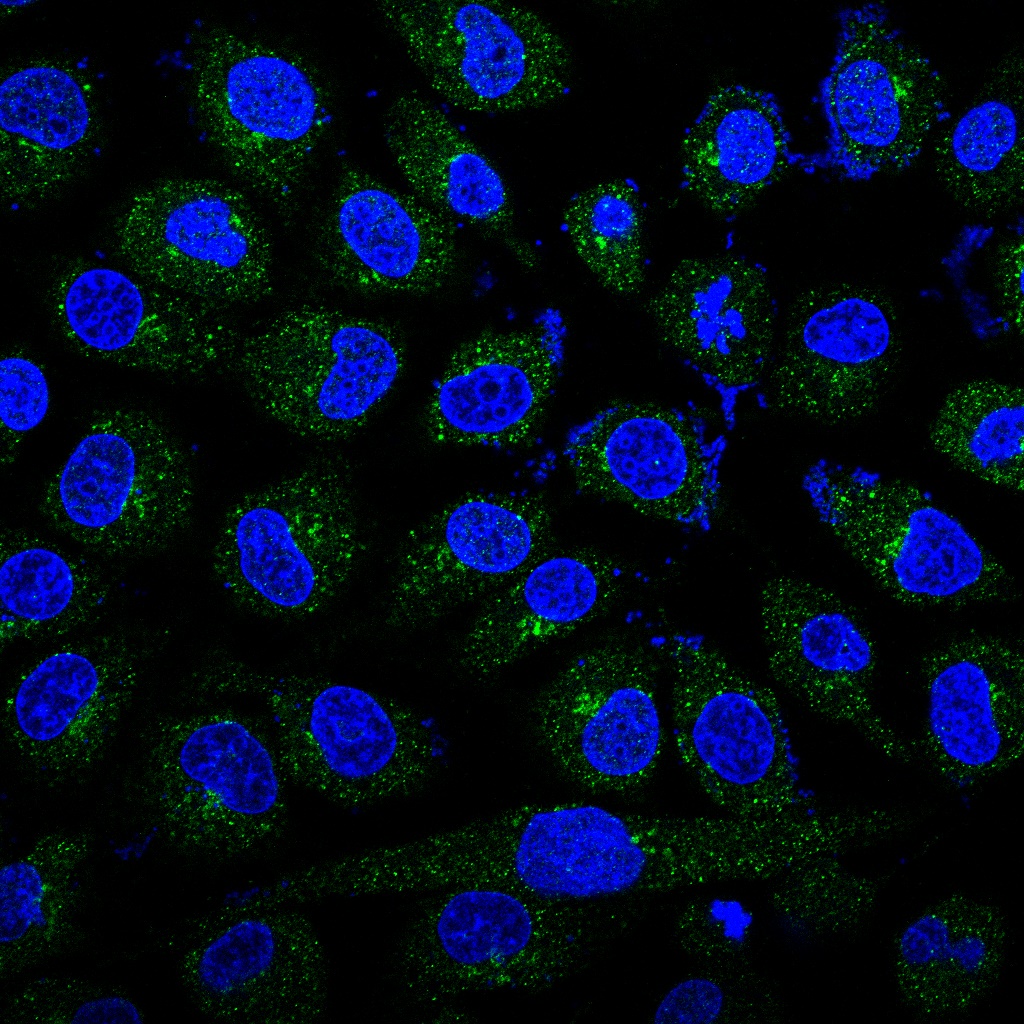

Supplement: Supplementary file 3 [file Data_Sheet_3.ZIP › Original data Fig. 4-7/Fig. 6/3.jpg]

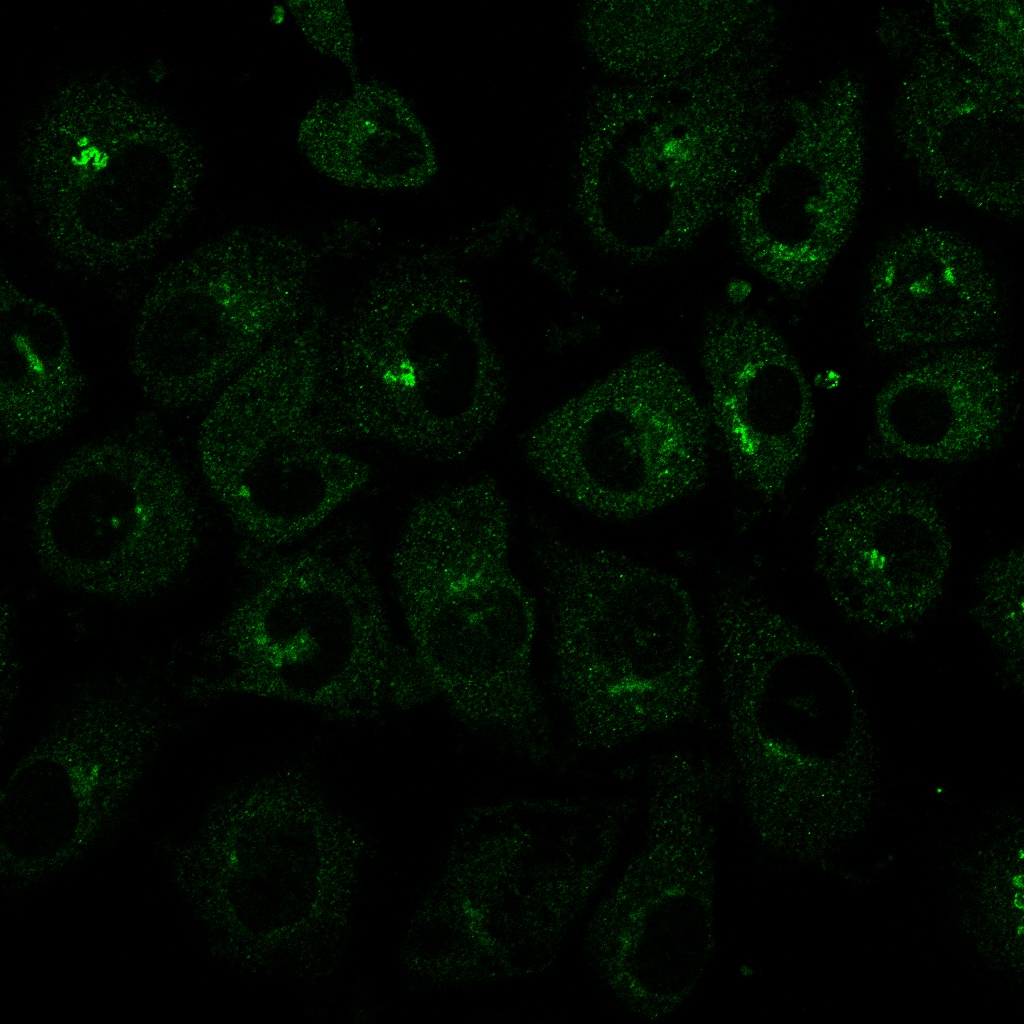

Supplement: Supplementary file 3 [file Data_Sheet_3.ZIP › Original data Fig. 4-7/Fig. 6/4.jpg]

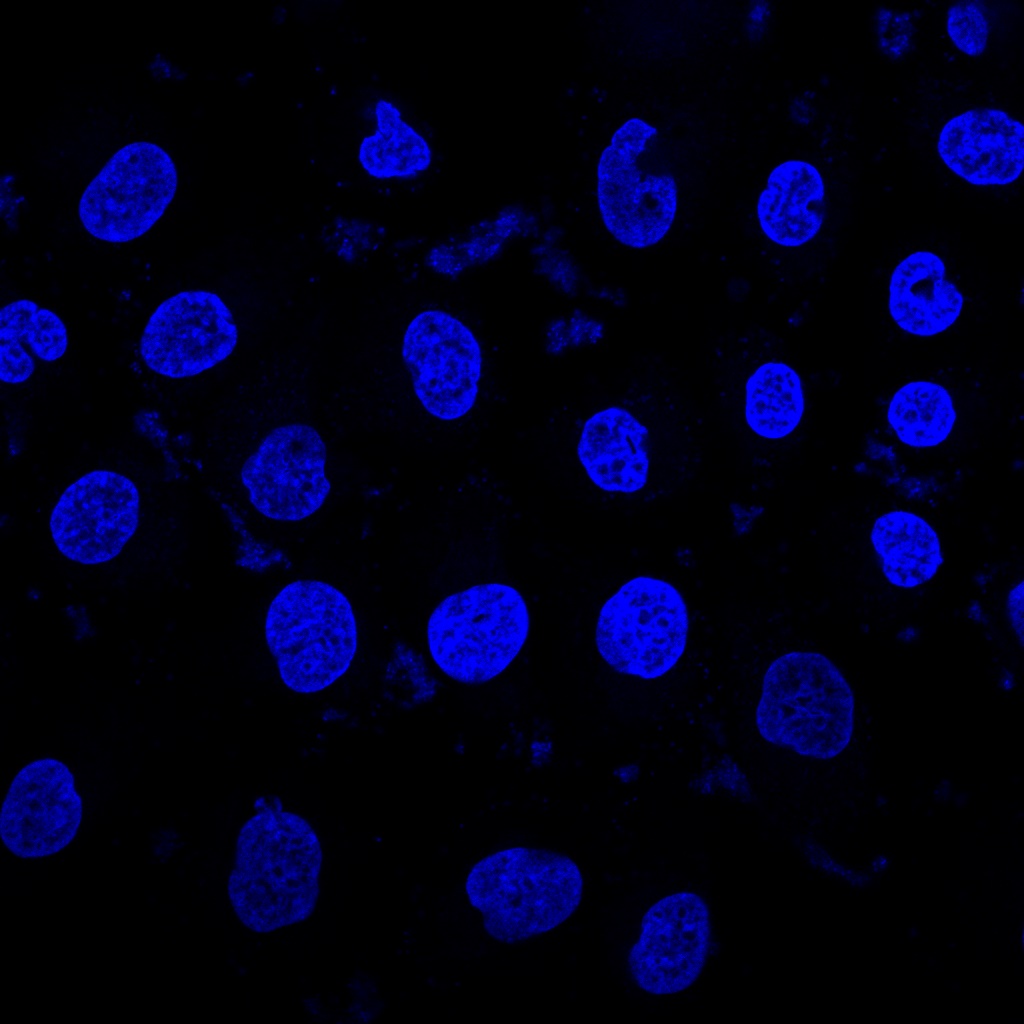

Supplement: Supplementary file 3 [file Data_Sheet_3.ZIP › Original data Fig. 4-7/Fig. 6/5.jpg]

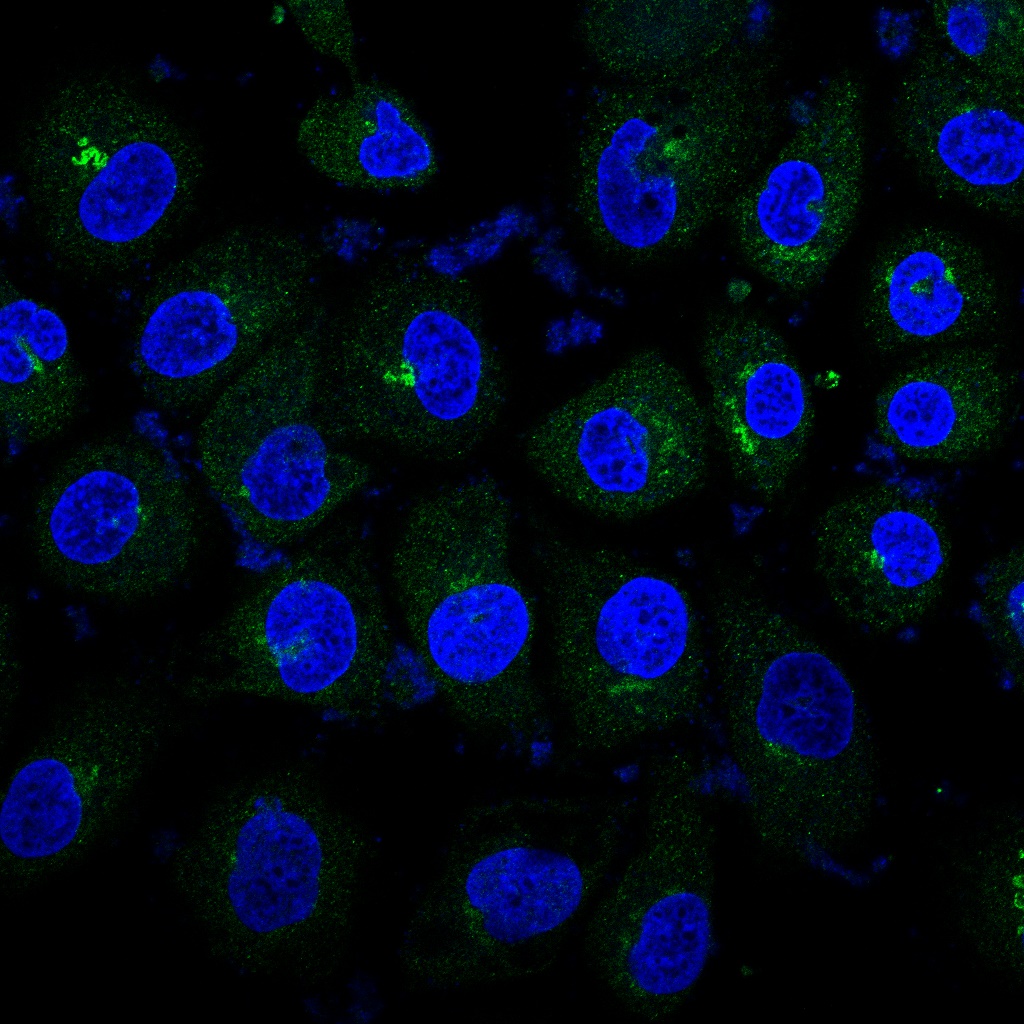

Supplement: Supplementary file 3 [file Data_Sheet_3.ZIP › Original data Fig. 4-7/Fig. 6/6.jpg]

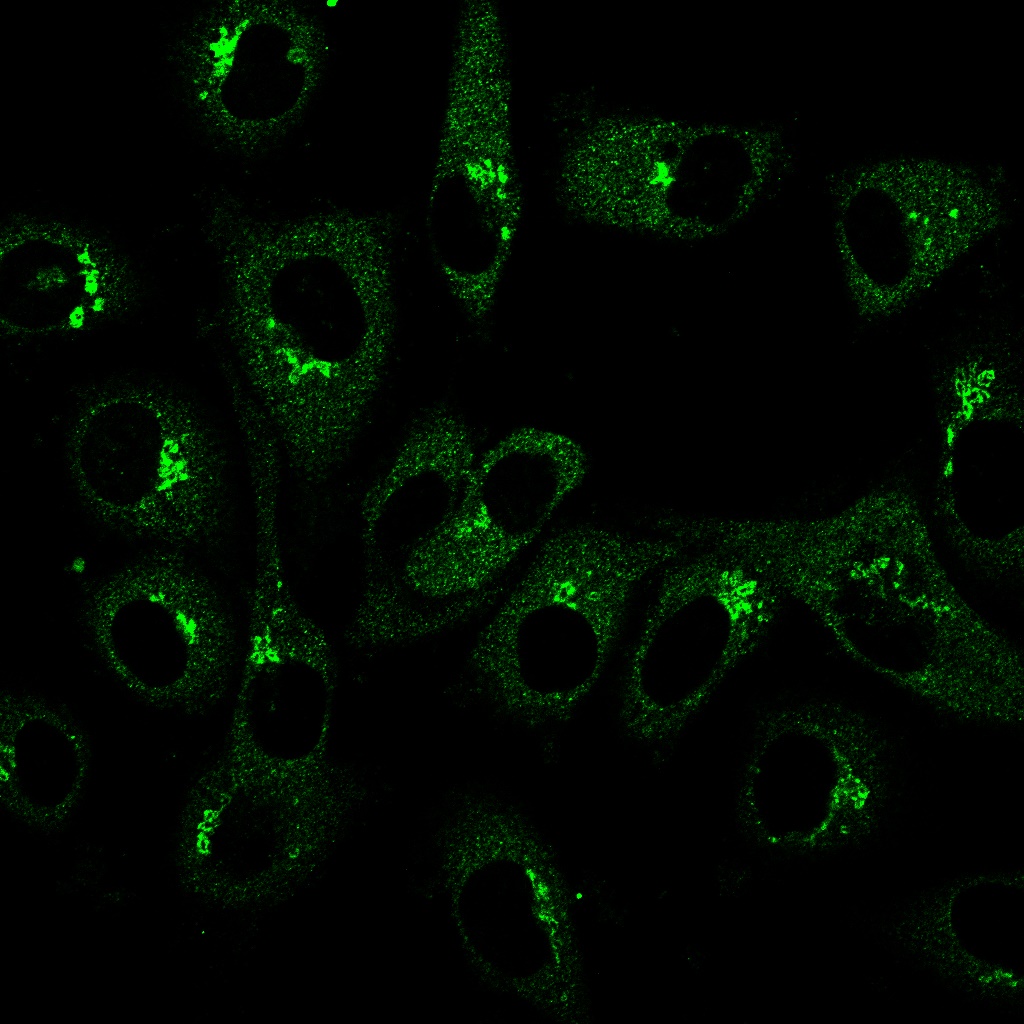

Supplement: Supplementary file 3 [file Data_Sheet_3.ZIP › Original data Fig. 4-7/Fig. 6/7.jpg]

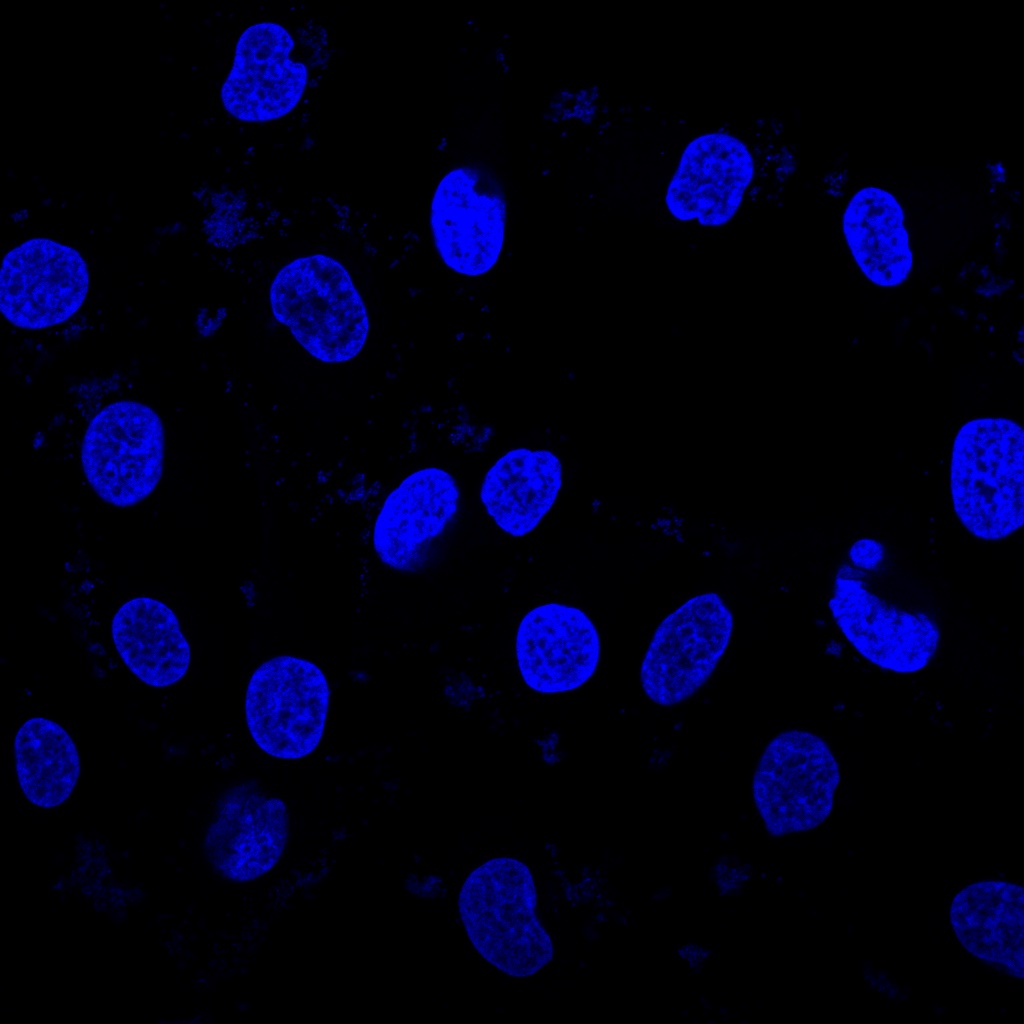

Supplement: Supplementary file 3 [file Data_Sheet_3.ZIP › Original data Fig. 4-7/Fig. 6/8.jpg]

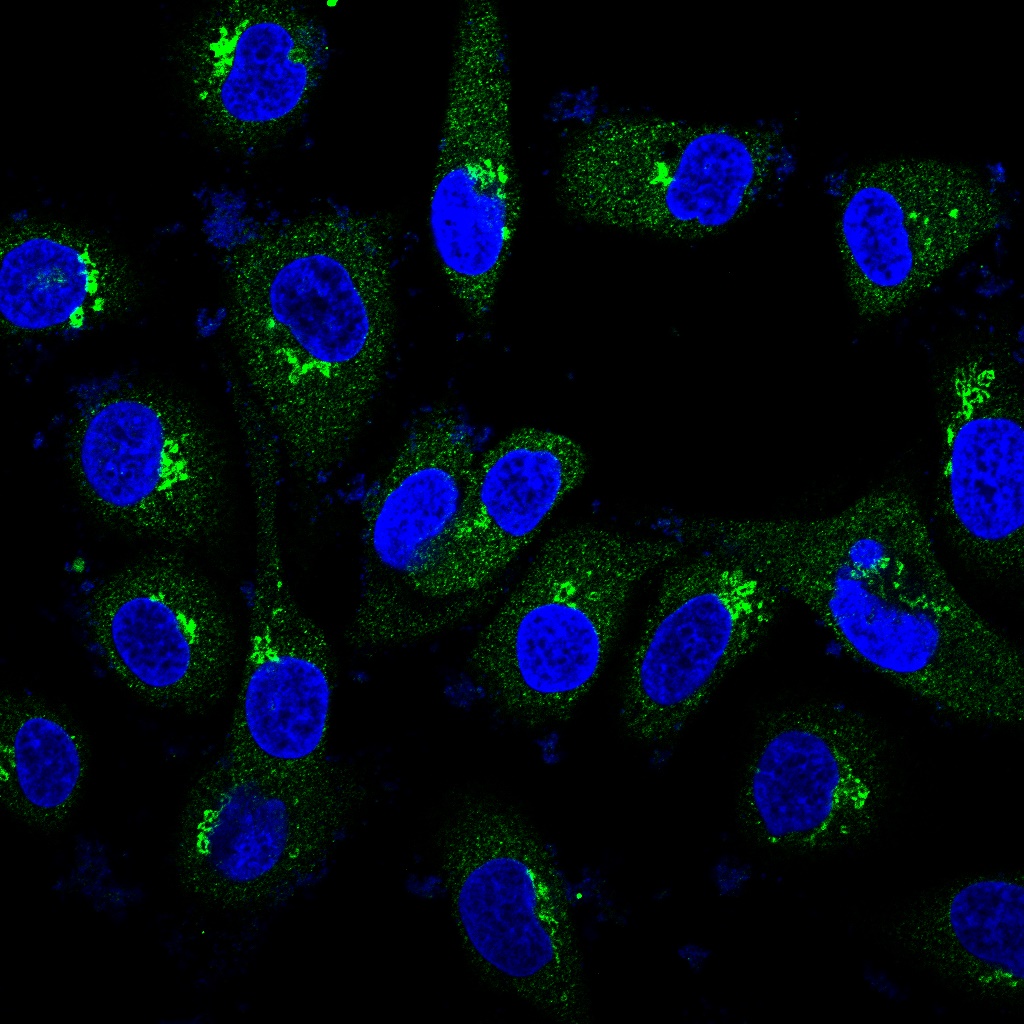

Supplement: Supplementary file 3 [file Data_Sheet_3.ZIP › Original data Fig. 4-7/Fig. 6/9.jpg]

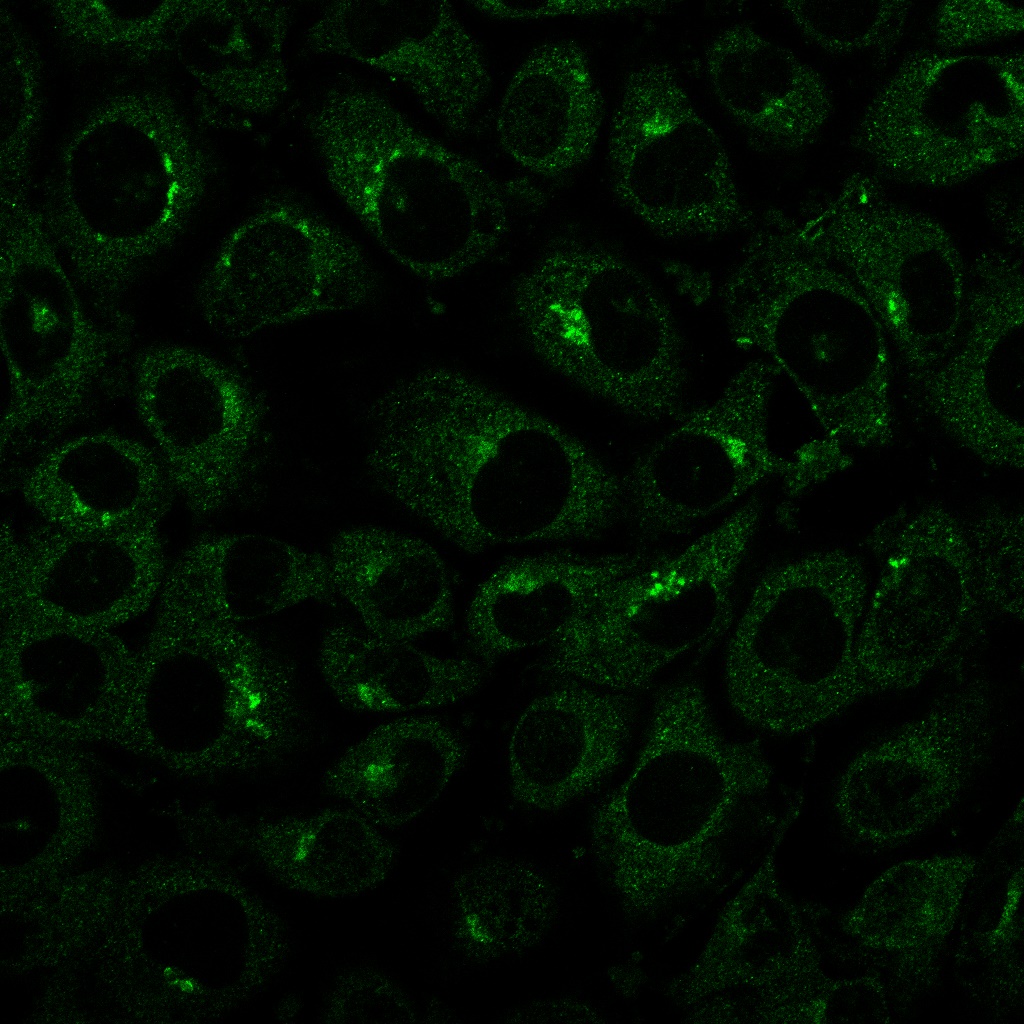

Supplement: Supplementary file 3 [file Data_Sheet_3.ZIP › Original data Fig. 4-7/Fig. 6/10.jpg]

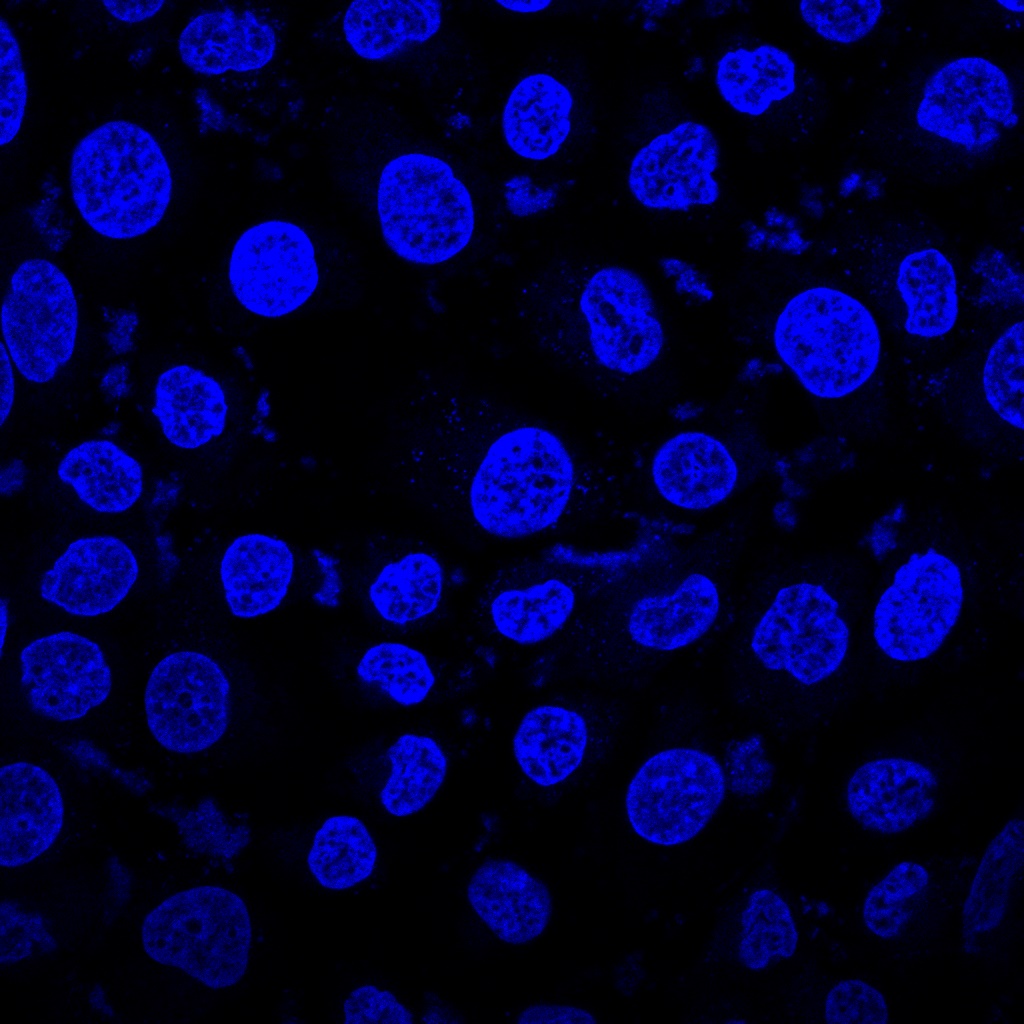

Supplement: Supplementary file 3 [file Data_Sheet_3.ZIP › Original data Fig. 4-7/Fig. 6/11.jpg]

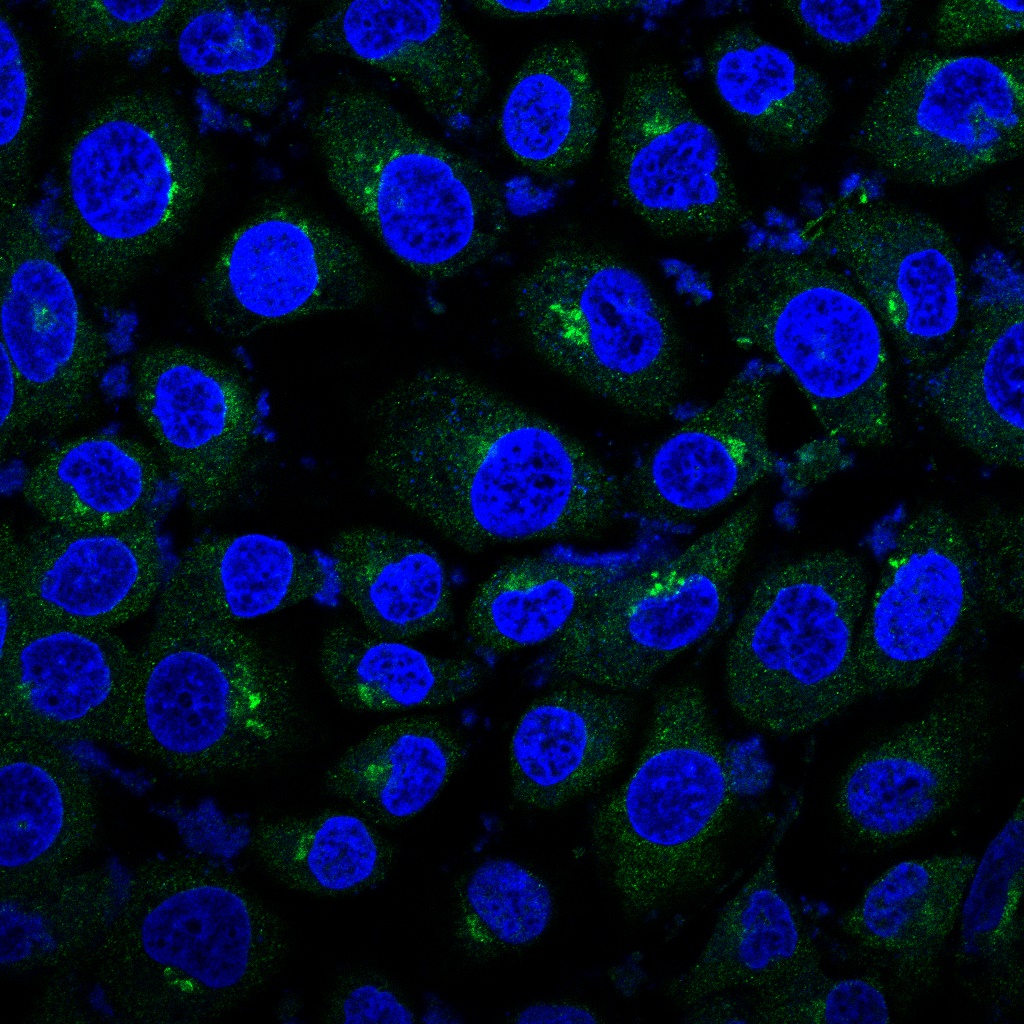

Supplement: Supplementary file 3 [file Data_Sheet_3.ZIP › Original data Fig. 4-7/Fig. 6/12.jpg]

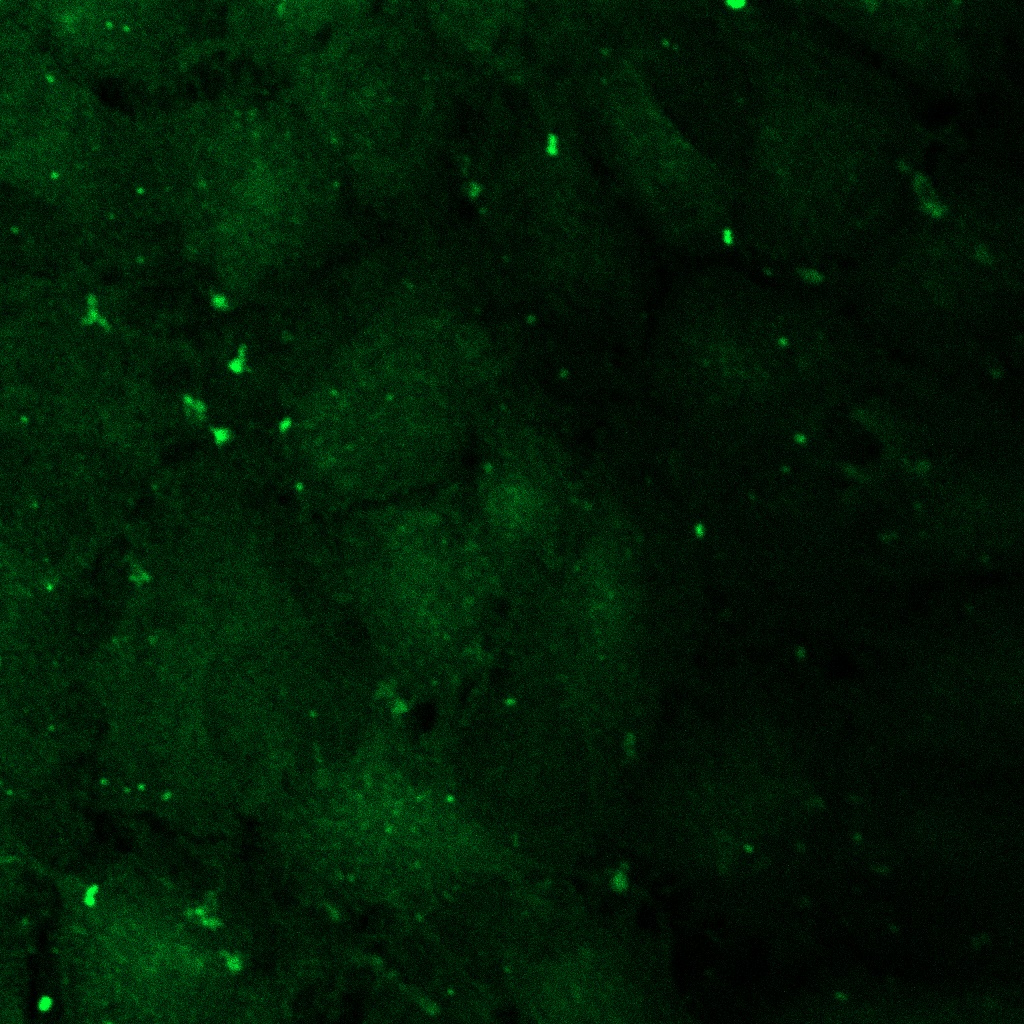

Supplement: Supplementary file 3 [file Data_Sheet_3.ZIP › Original data Fig. 4-7/Fig. 6/13.jpg]

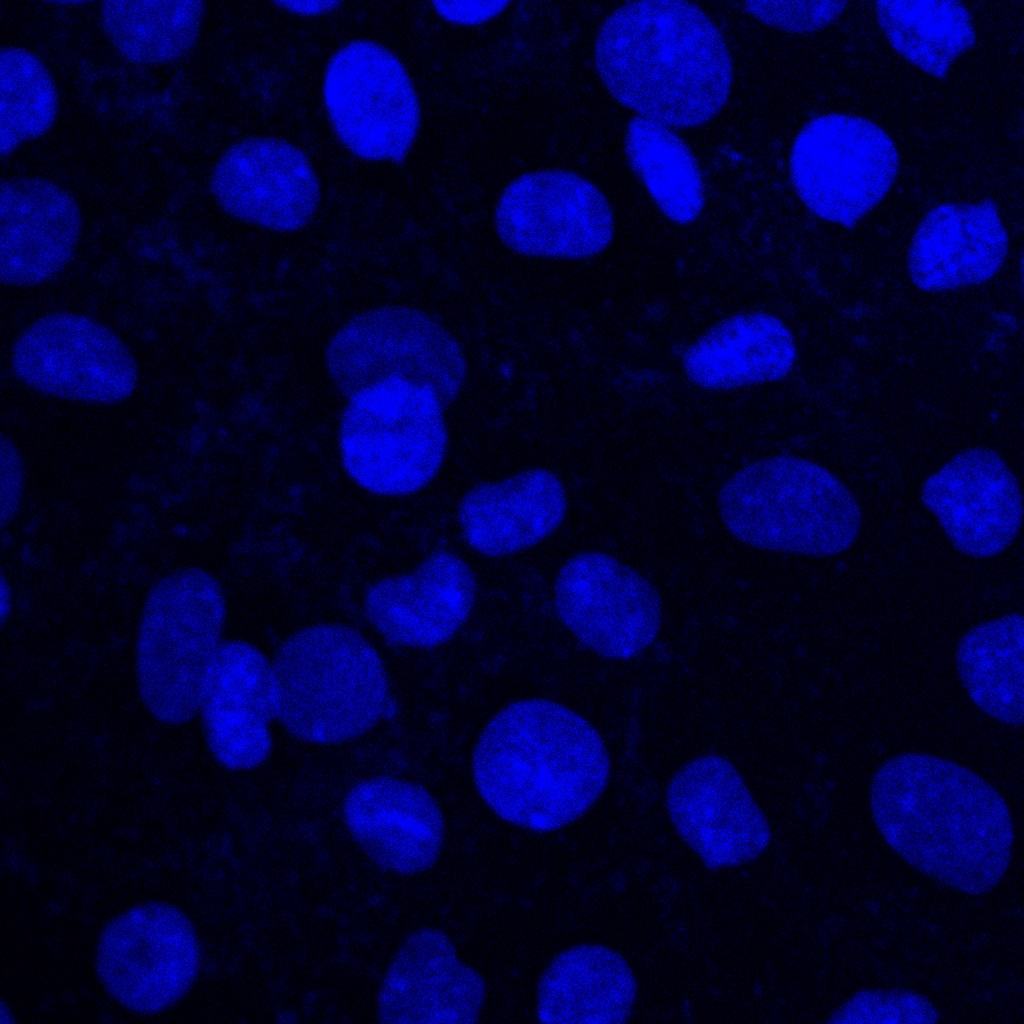

Supplement: Supplementary file 3 [file Data_Sheet_3.ZIP › Original data Fig. 4-7/Fig. 6/14.jpg]

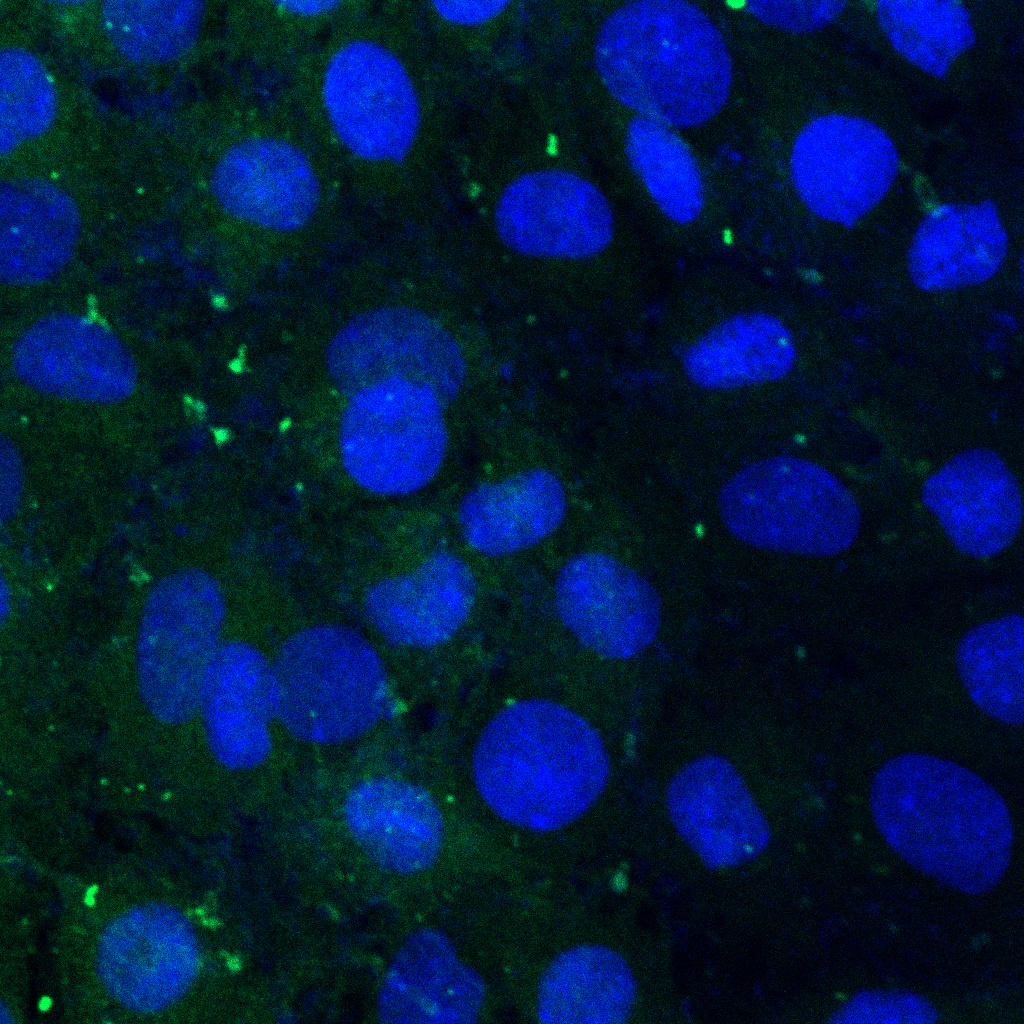

Supplement: Supplementary file 3 [file Data_Sheet_3.ZIP › Original data Fig. 4-7/Fig. 6/15.jpg]

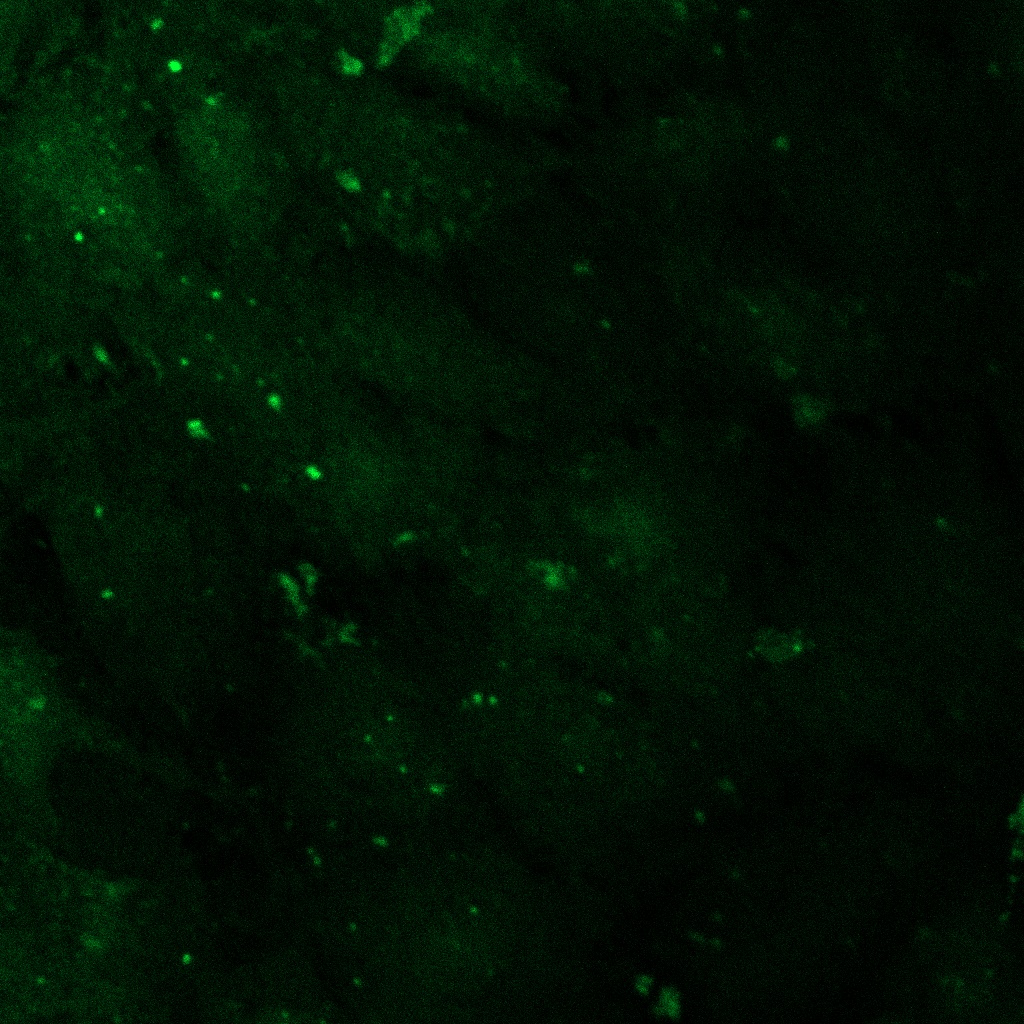

Supplement: Supplementary file 3 [file Data_Sheet_3.ZIP › Original data Fig. 4-7/Fig. 6/16.jpg]

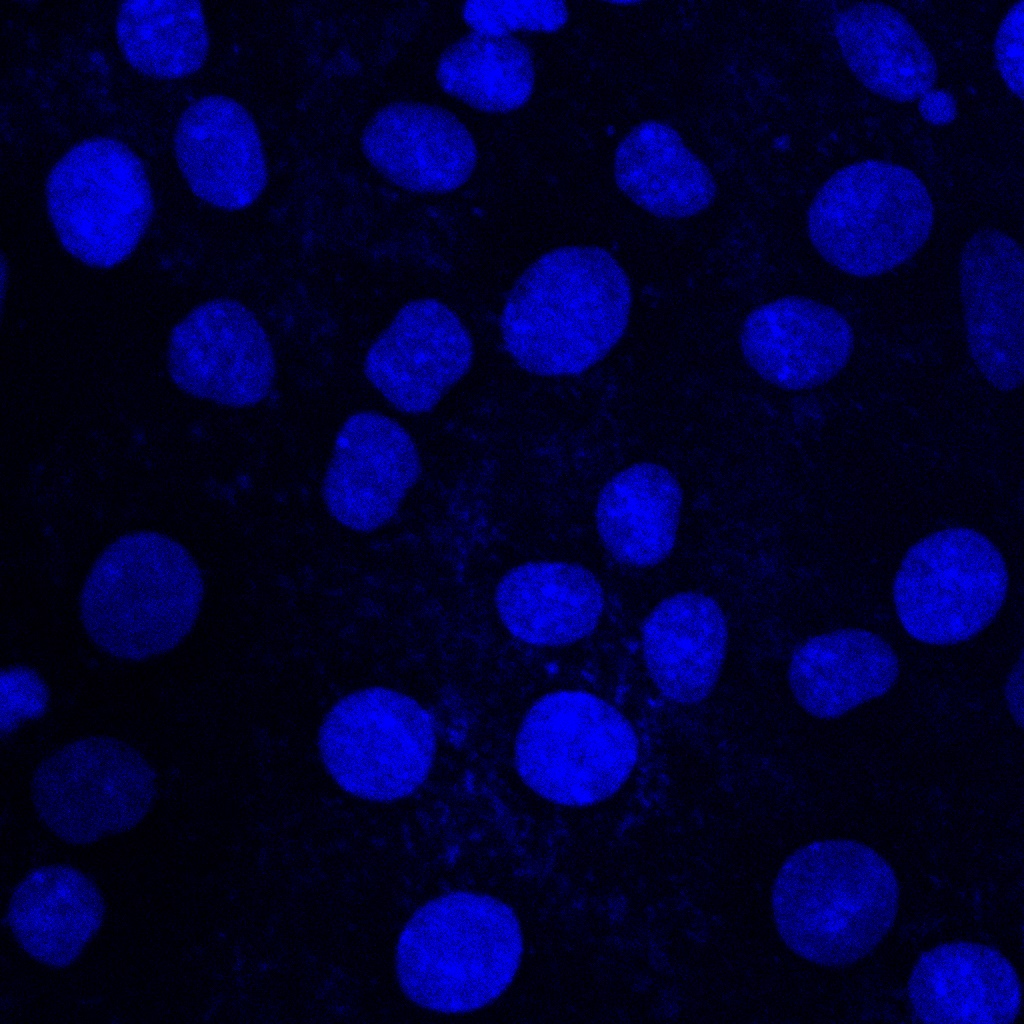

Supplement: Supplementary file 3 [file Data_Sheet_3.ZIP › Original data Fig. 4-7/Fig. 6/17.jpg]

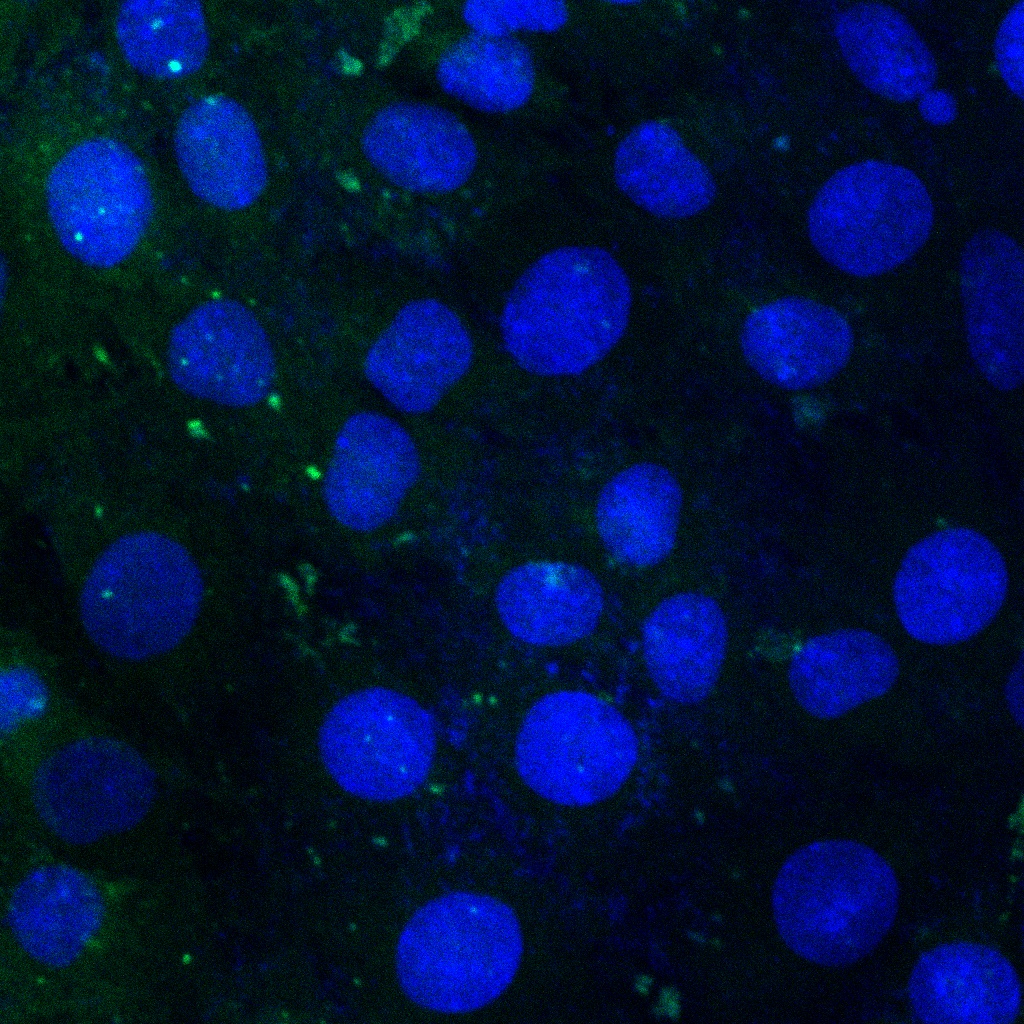

Supplement: Supplementary file 3 [file Data_Sheet_3.ZIP › Original data Fig. 4-7/Fig. 6/18.jpg]

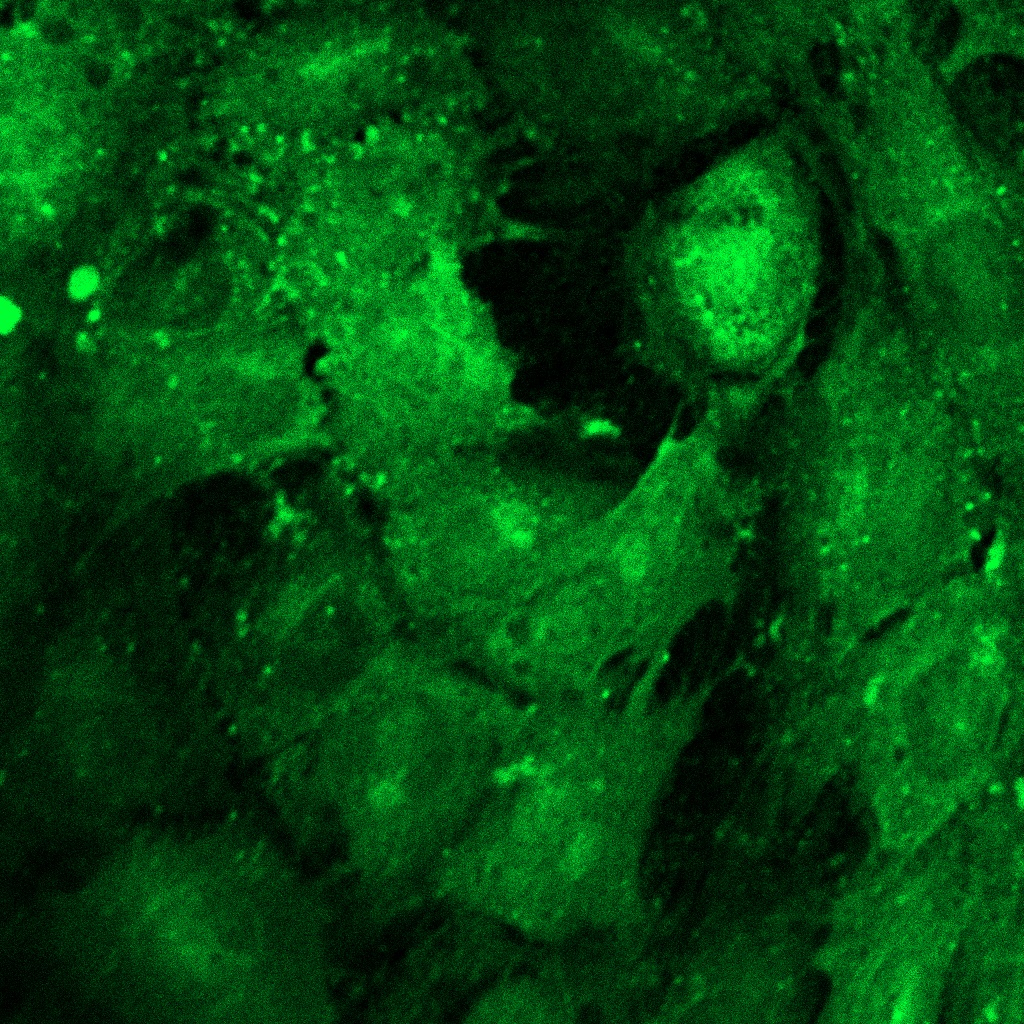

Supplement: Supplementary file 3 [file Data_Sheet_3.ZIP › Original data Fig. 4-7/Fig. 6/19.jpg]

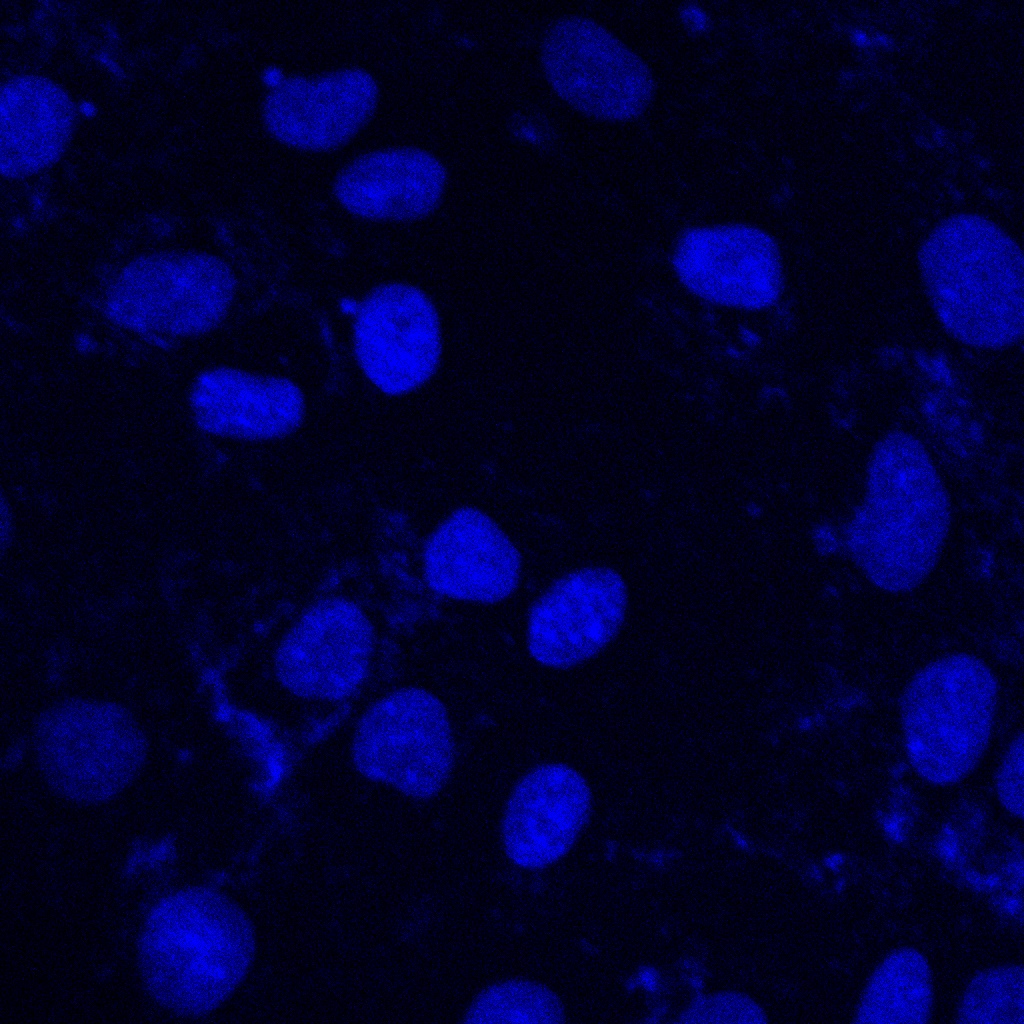

Supplement: Supplementary file 3 [file Data_Sheet_3.ZIP › Original data Fig. 4-7/Fig. 6/20.jpg]

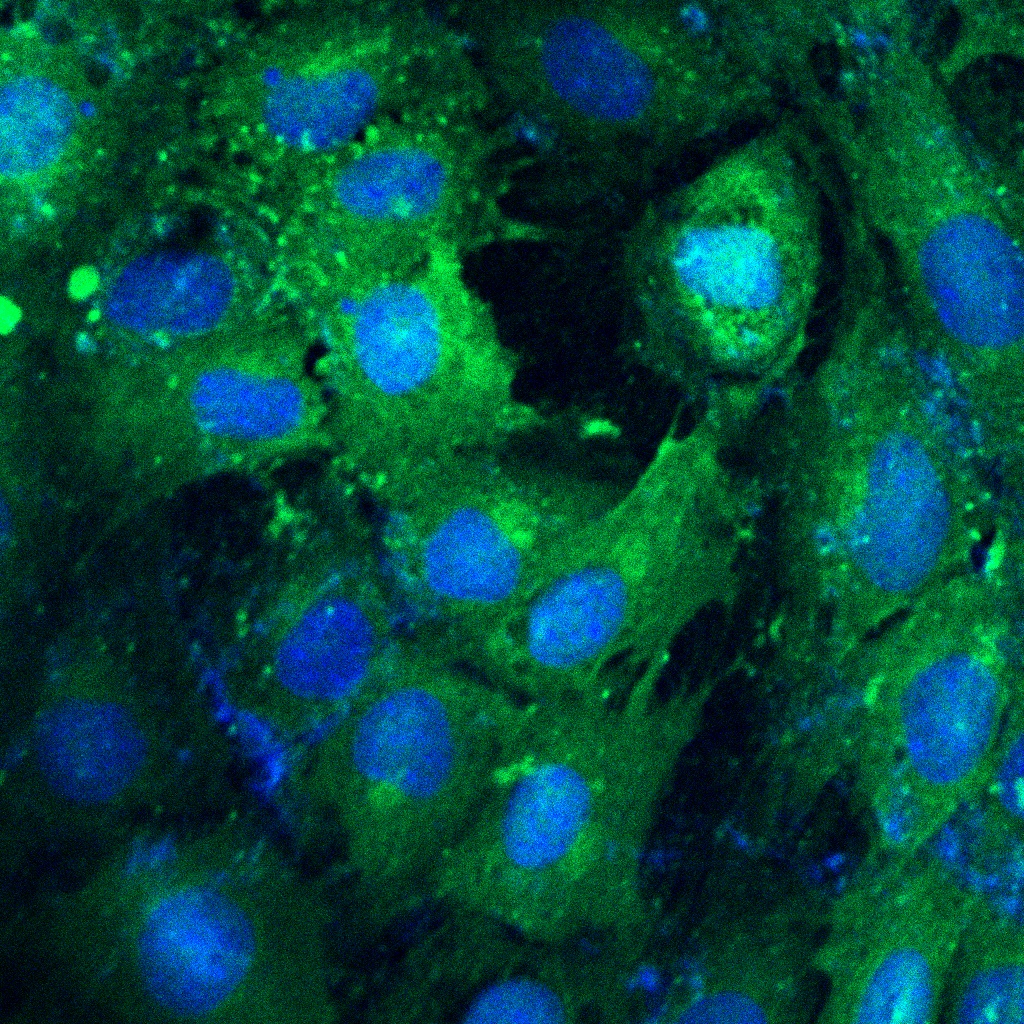

Supplement: Supplementary file 3 [file Data_Sheet_3.ZIP › Original data Fig. 4-7/Fig. 6/21.jpg]

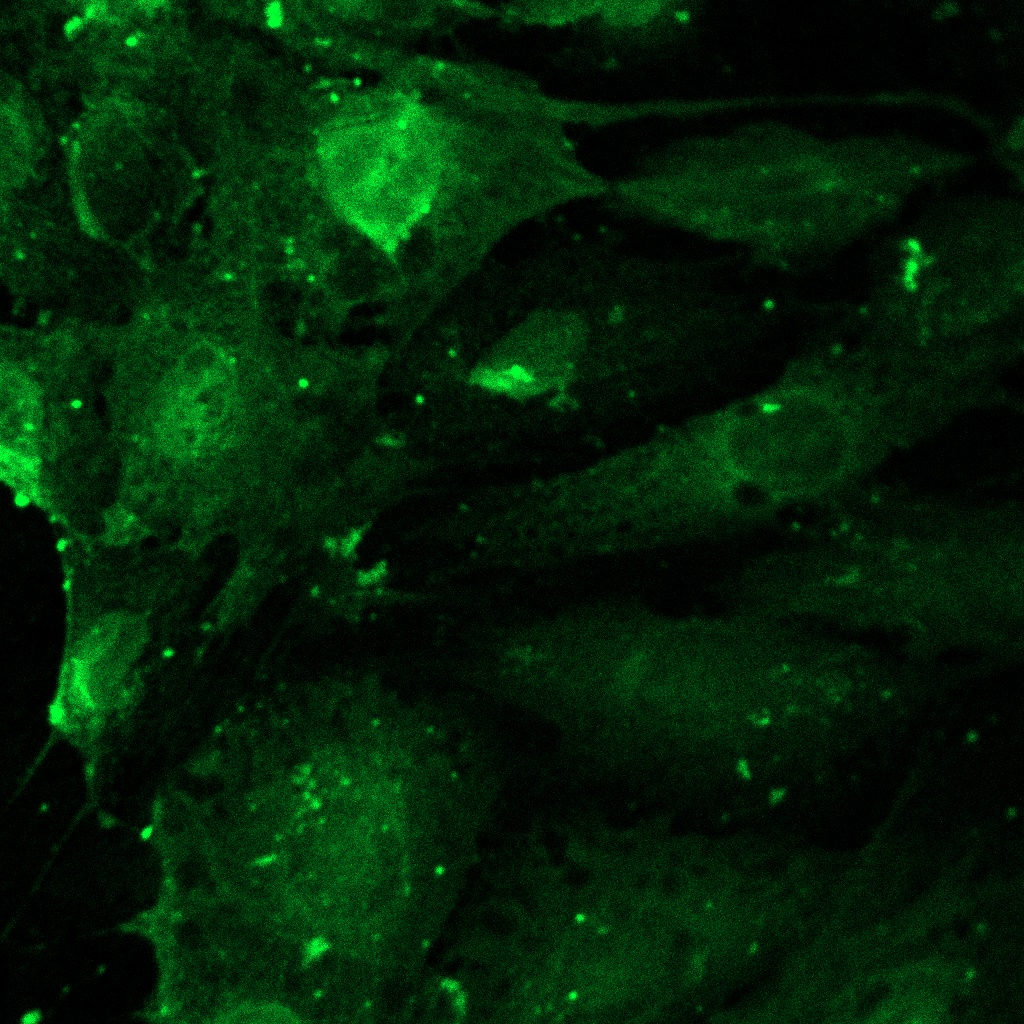

Supplement: Supplementary file 3 [file Data_Sheet_3.ZIP › Original data Fig. 4-7/Fig. 6/22.jpg]

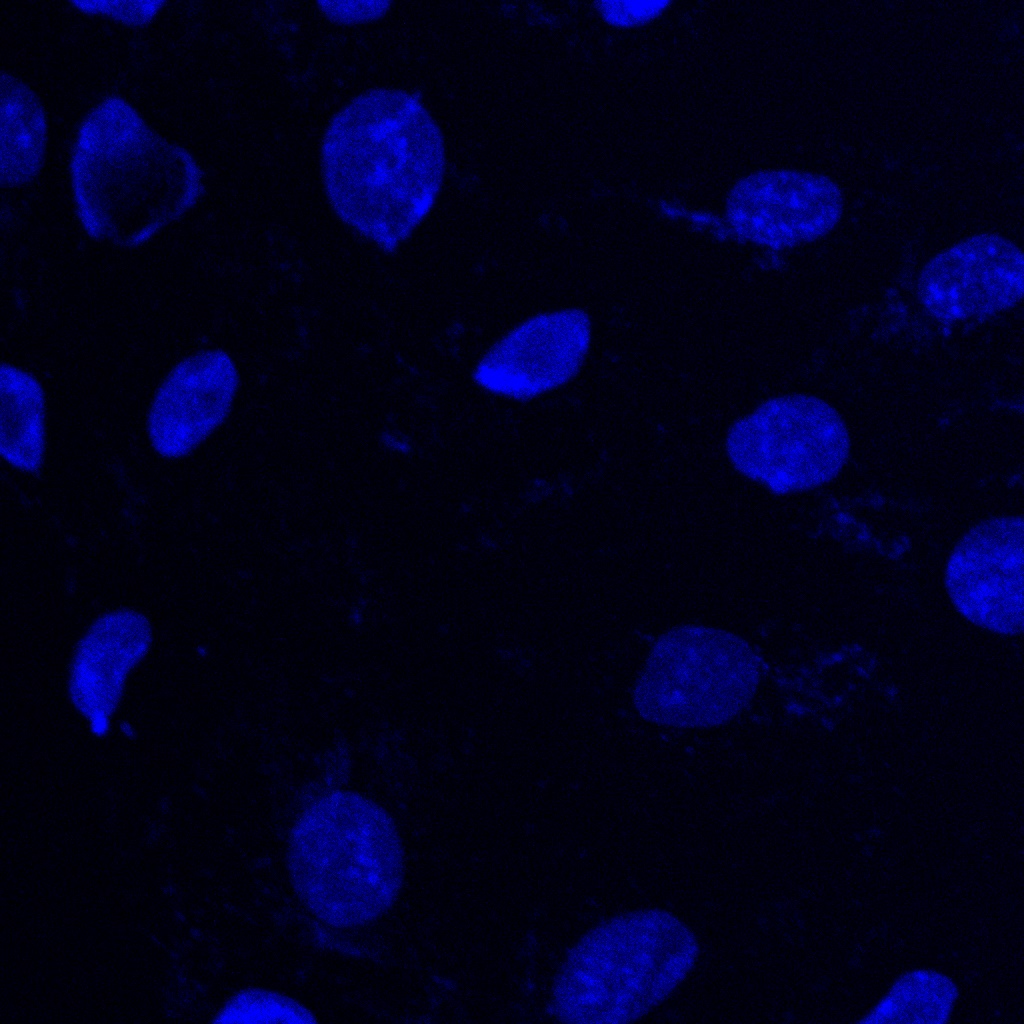

Supplement: Supplementary file 3 [file Data_Sheet_3.ZIP › Original data Fig. 4-7/Fig. 6/23.jpg]

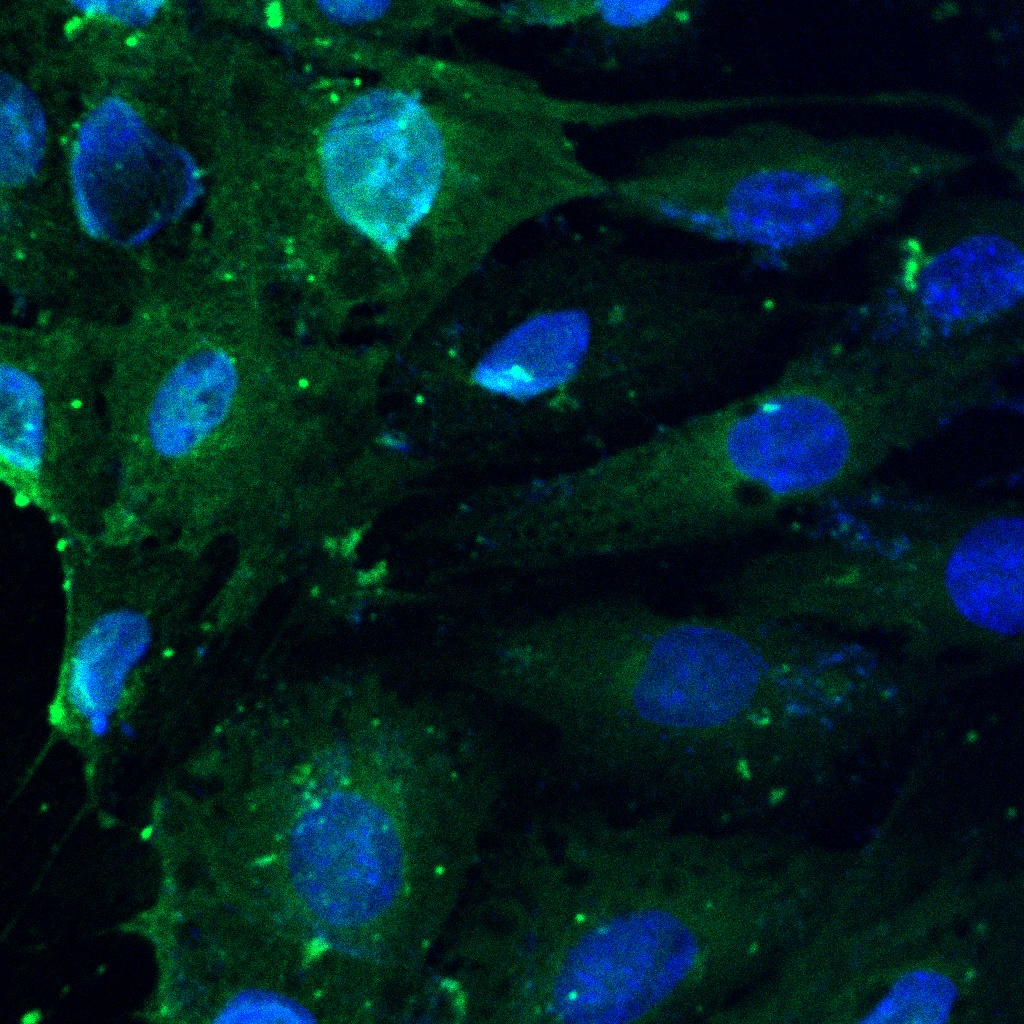

Supplement: Supplementary file 3 [file Data_Sheet_3.ZIP › Original data Fig. 4-7/Fig. 6/24.jpg]

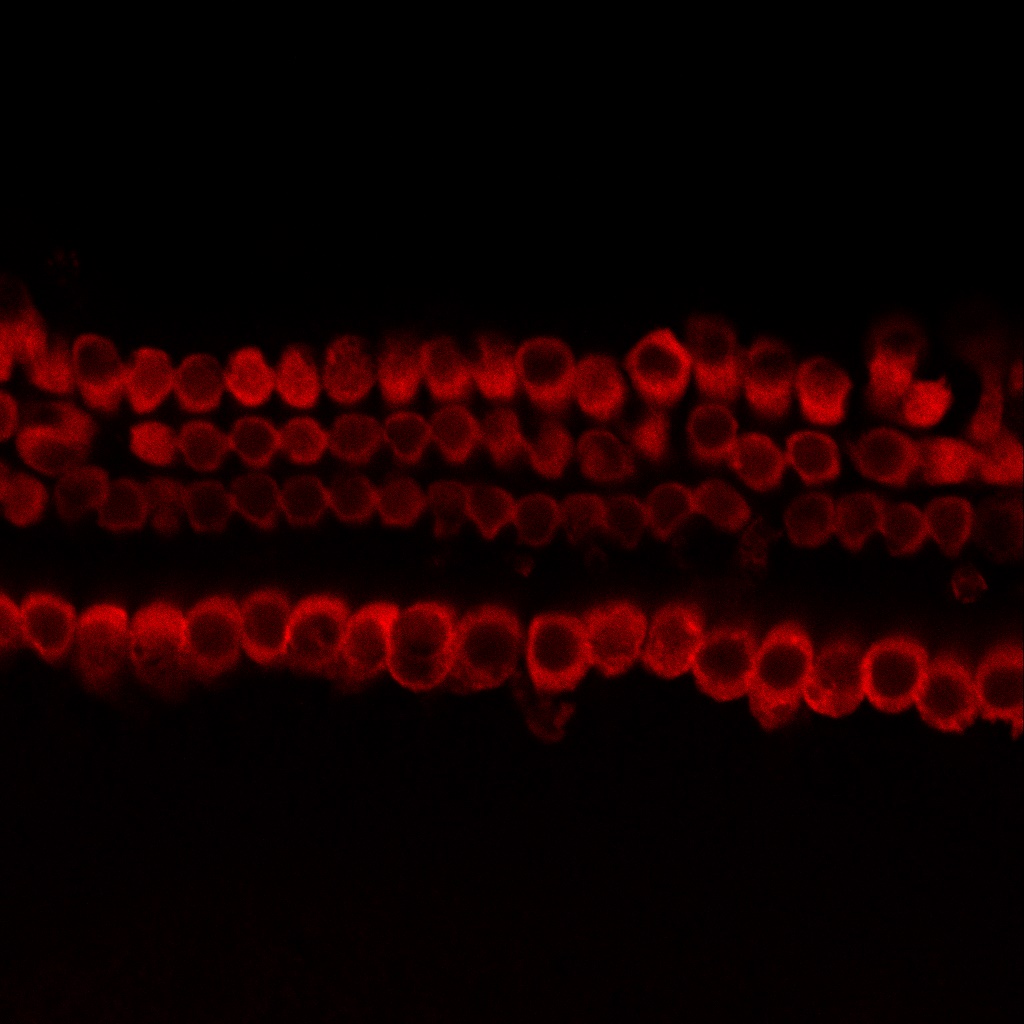

Supplement: Supplementary file 3 [file Data_Sheet_3.ZIP › Original data Fig. 4-7/Fig. 4/1.jpg]

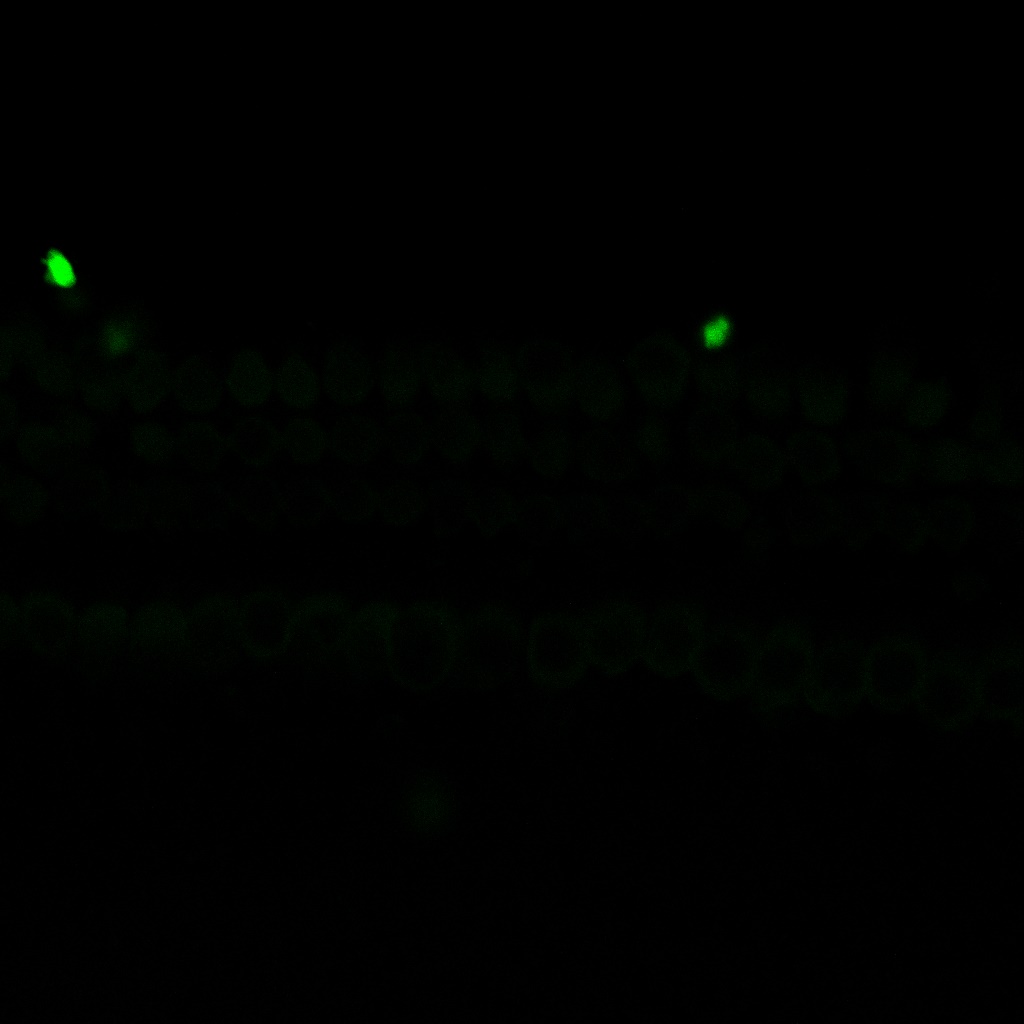

Supplement: Supplementary file 3 [file Data_Sheet_3.ZIP › Original data Fig. 4-7/Fig. 4/2.jpg]

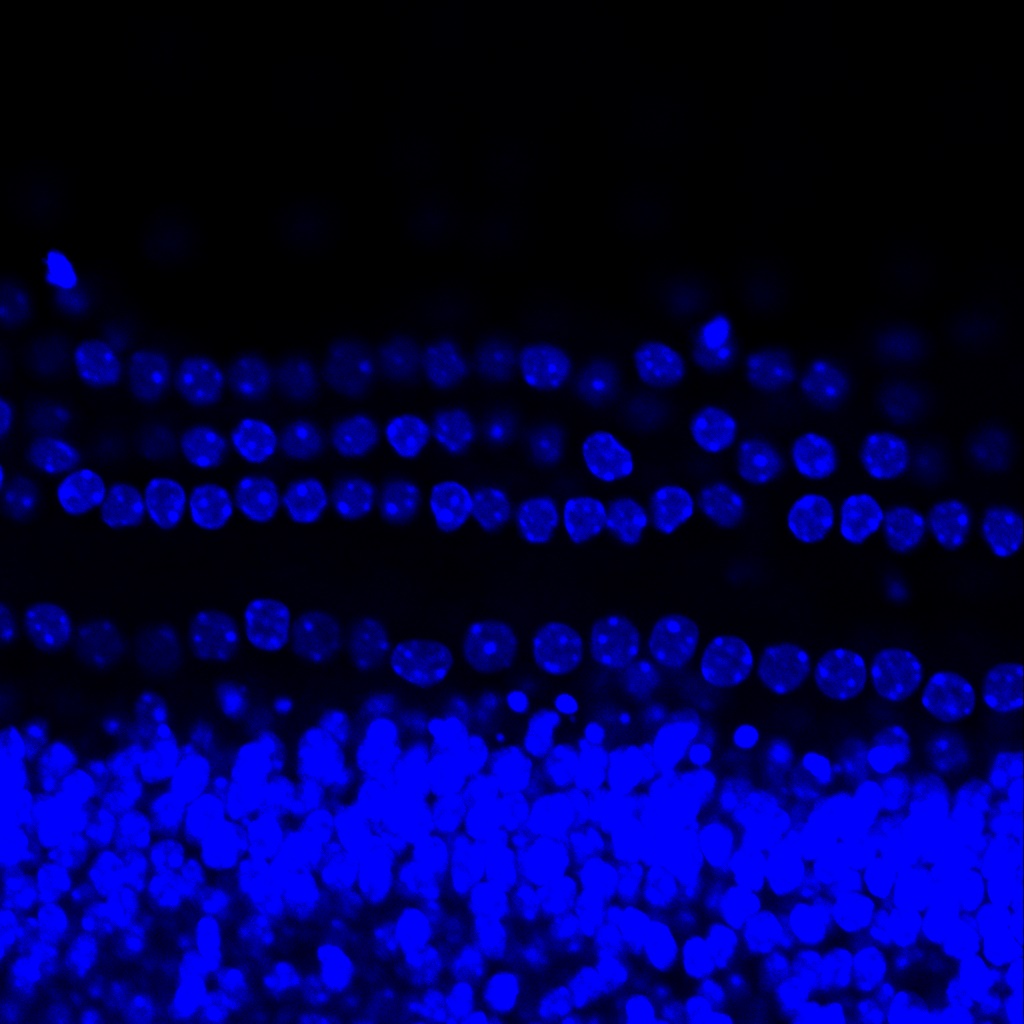

Supplement: Supplementary file 3 [file Data_Sheet_3.ZIP › Original data Fig. 4-7/Fig. 4/3.jpg]

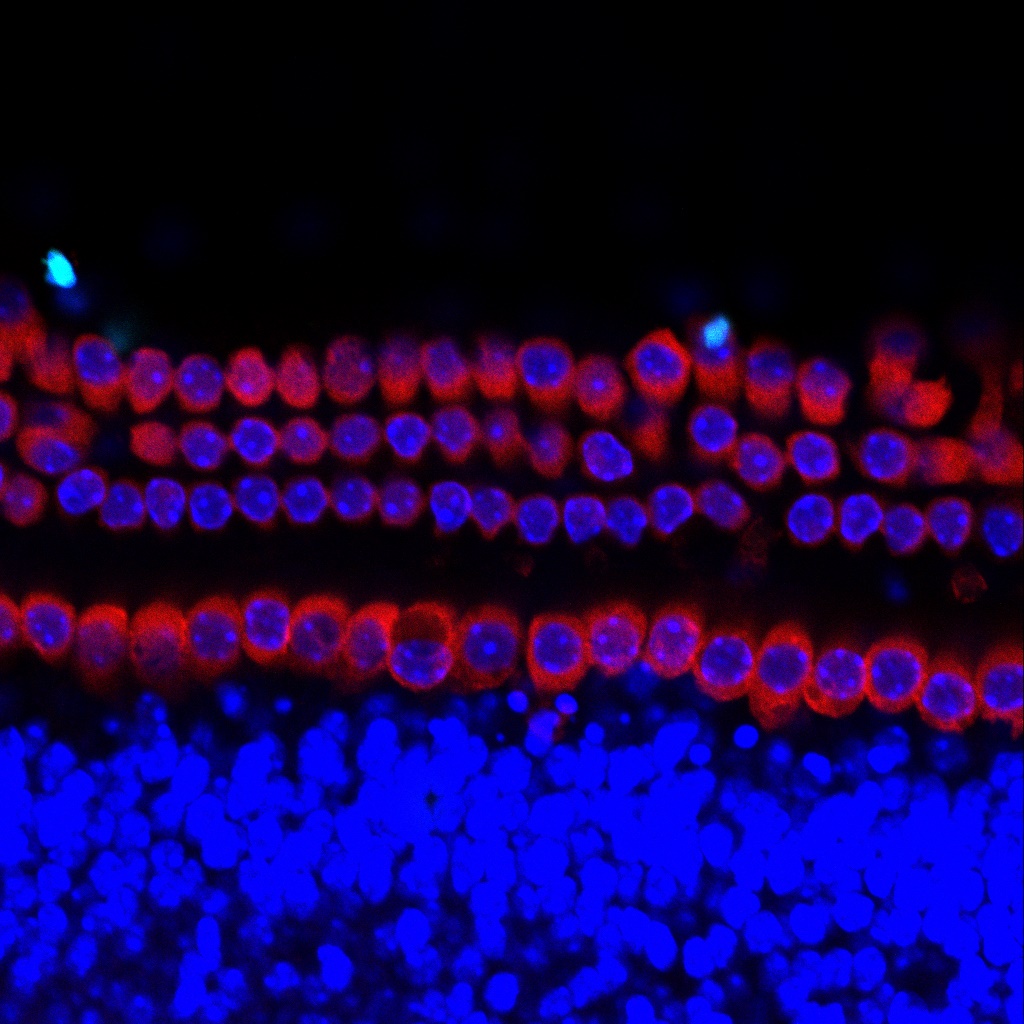

Supplement: Supplementary file 3 [file Data_Sheet_3.ZIP › Original data Fig. 4-7/Fig. 4/4.jpg]

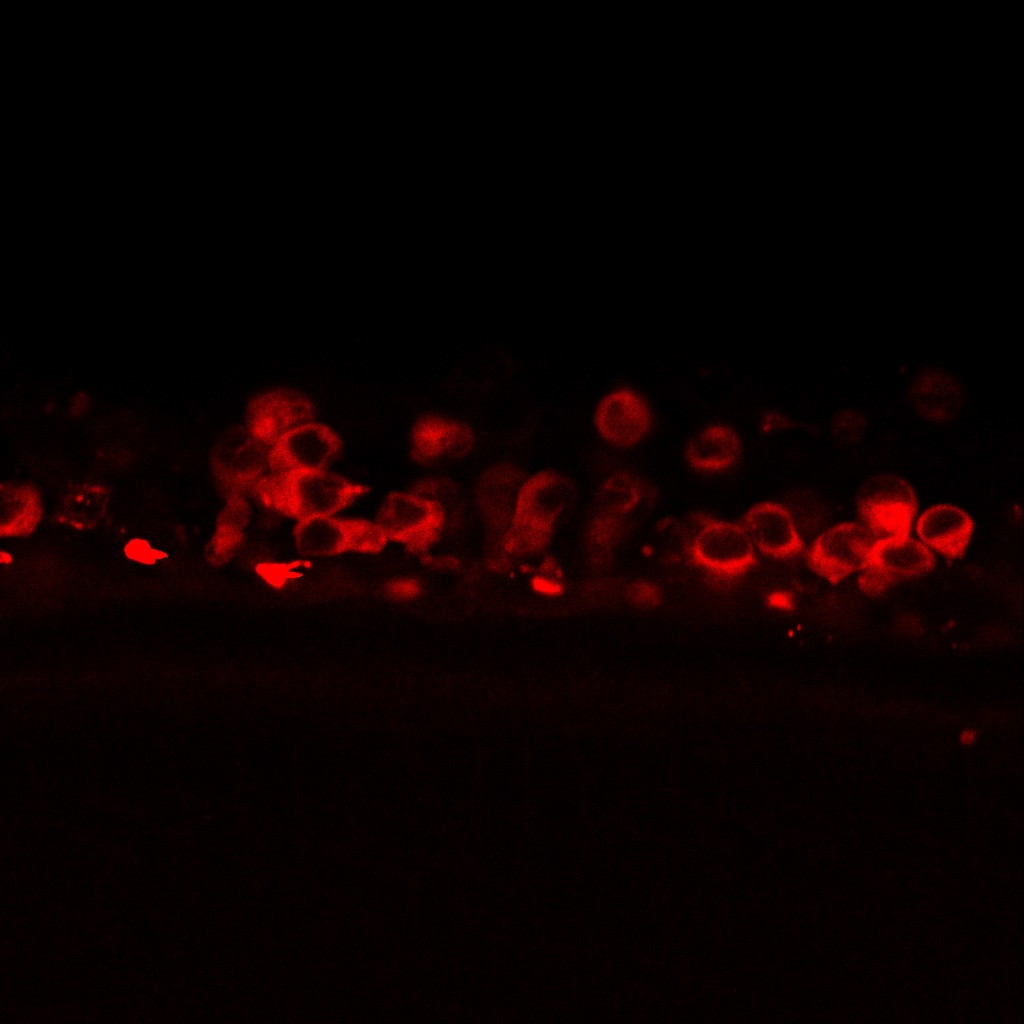

Supplement: Supplementary file 3 [file Data_Sheet_3.ZIP › Original data Fig. 4-7/Fig. 4/5.jpg]

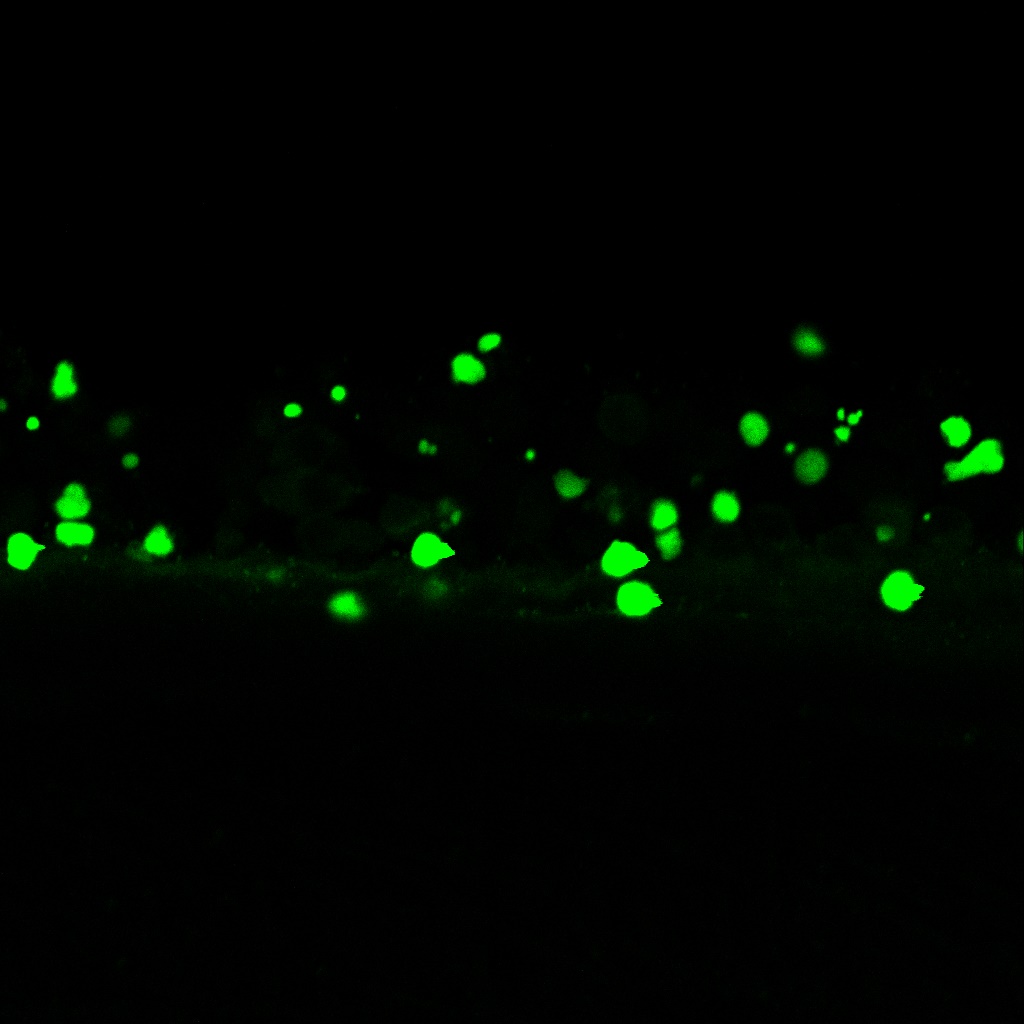

Supplement: Supplementary file 3 [file Data_Sheet_3.ZIP › Original data Fig. 4-7/Fig. 4/6.jpg]
