# Supplementary figures and images for: Pitavastatin protects against neomycin-induced ototoxicity through inhibition of endoplasmic reticulum stress (part 2 of 2)
Source: Front Mol Neurosci. 2022 Aug 3;15:963083. doi: 10.3389/fnmol.2022.963083 (PMC9381809; doi:10.3389/fnmol.2022.963083)

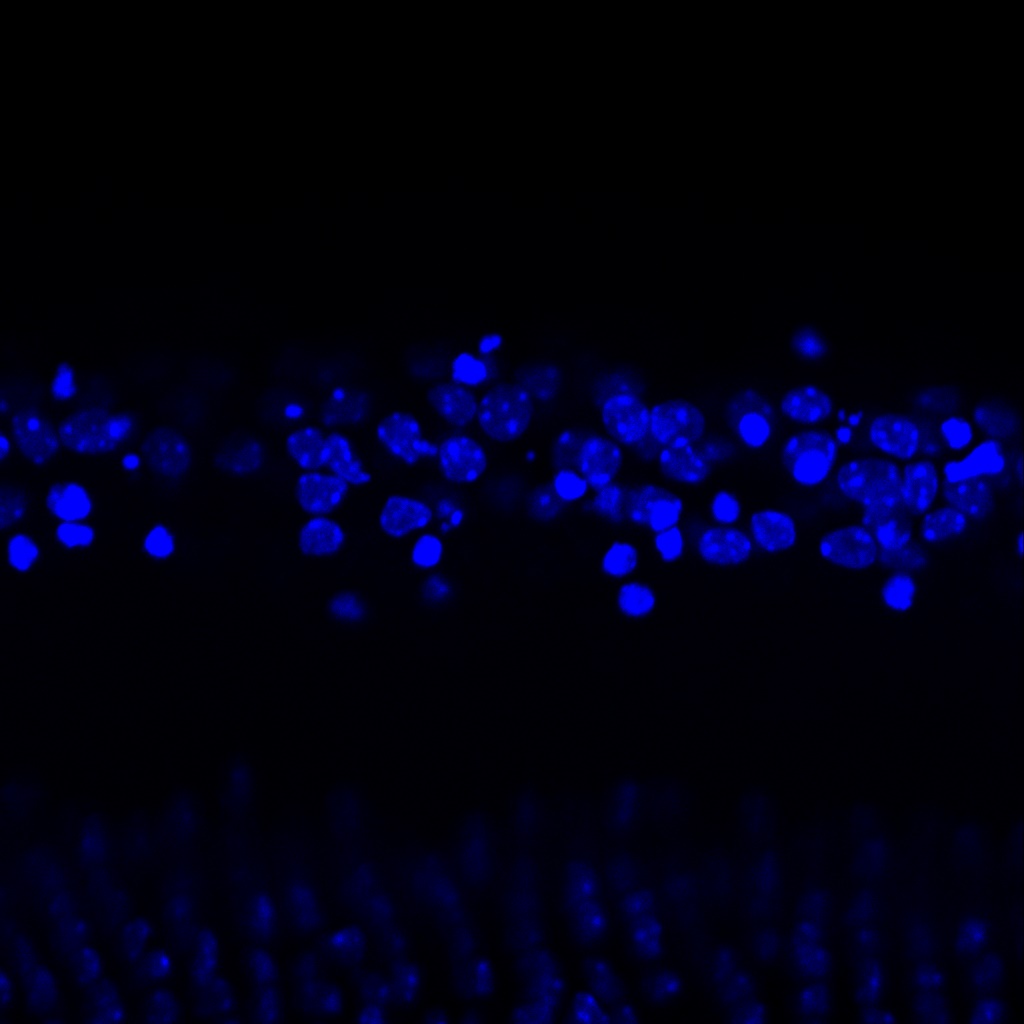

Supplement: Supplementary file 3 [file Data_Sheet_3.ZIP › Original data Fig. 4-7/Fig. 4/7.jpg]

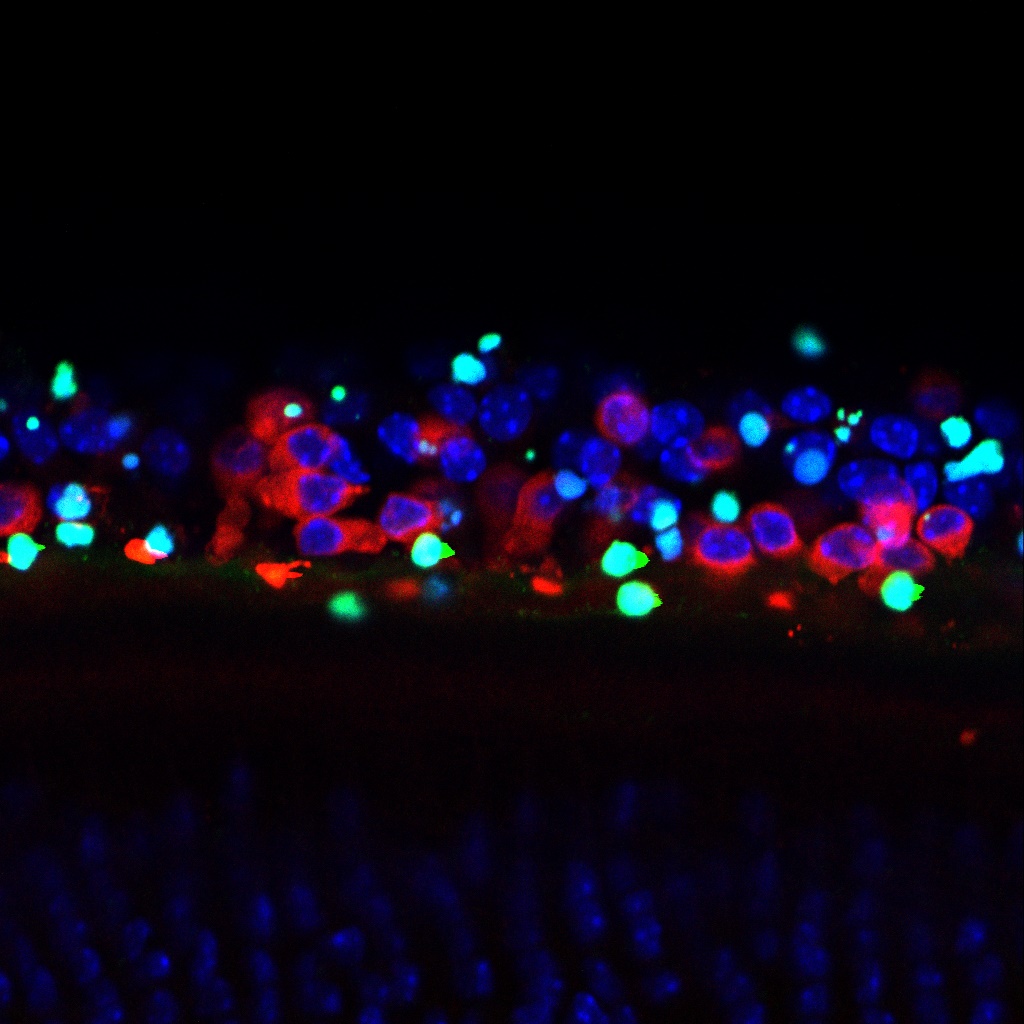

Supplement: Supplementary file 3 [file Data_Sheet_3.ZIP › Original data Fig. 4-7/Fig. 4/8.jpg]

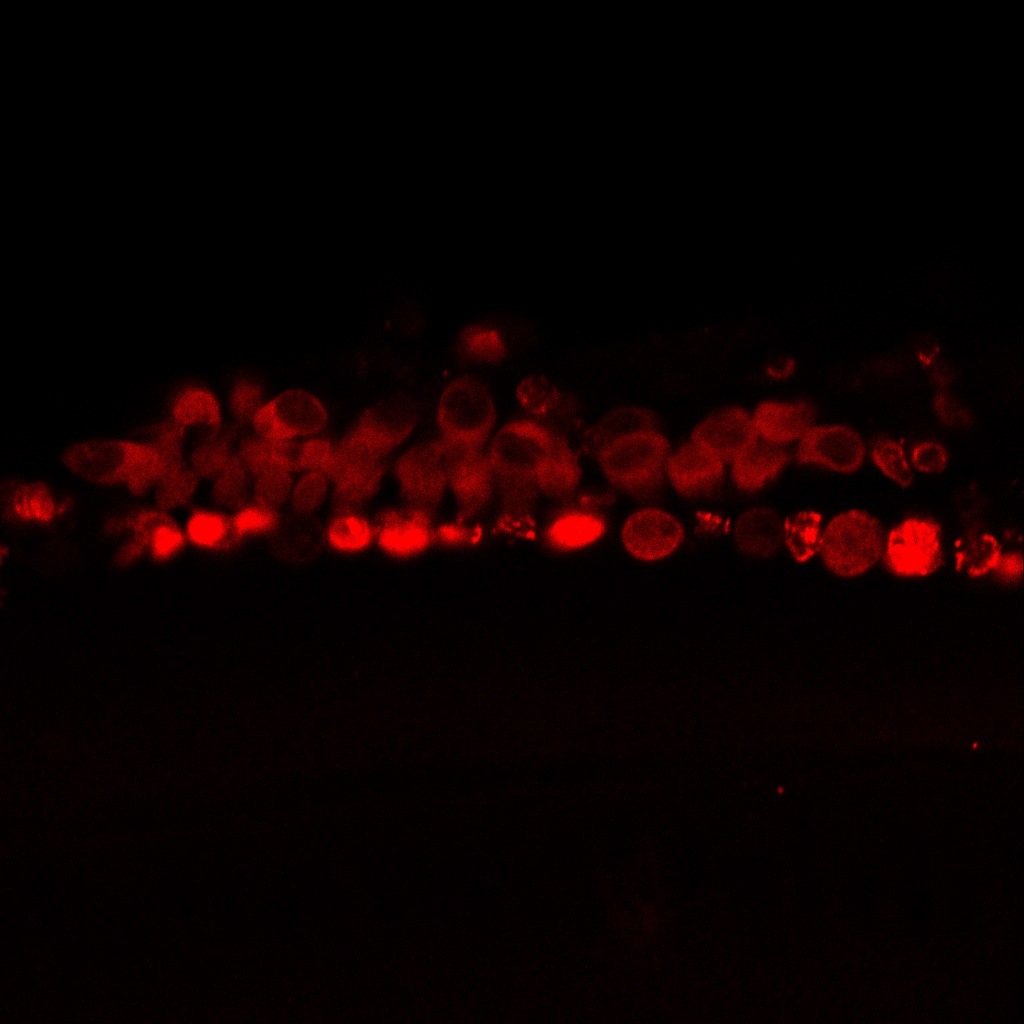

Supplement: Supplementary file 3 [file Data_Sheet_3.ZIP › Original data Fig. 4-7/Fig. 4/9.jpg]

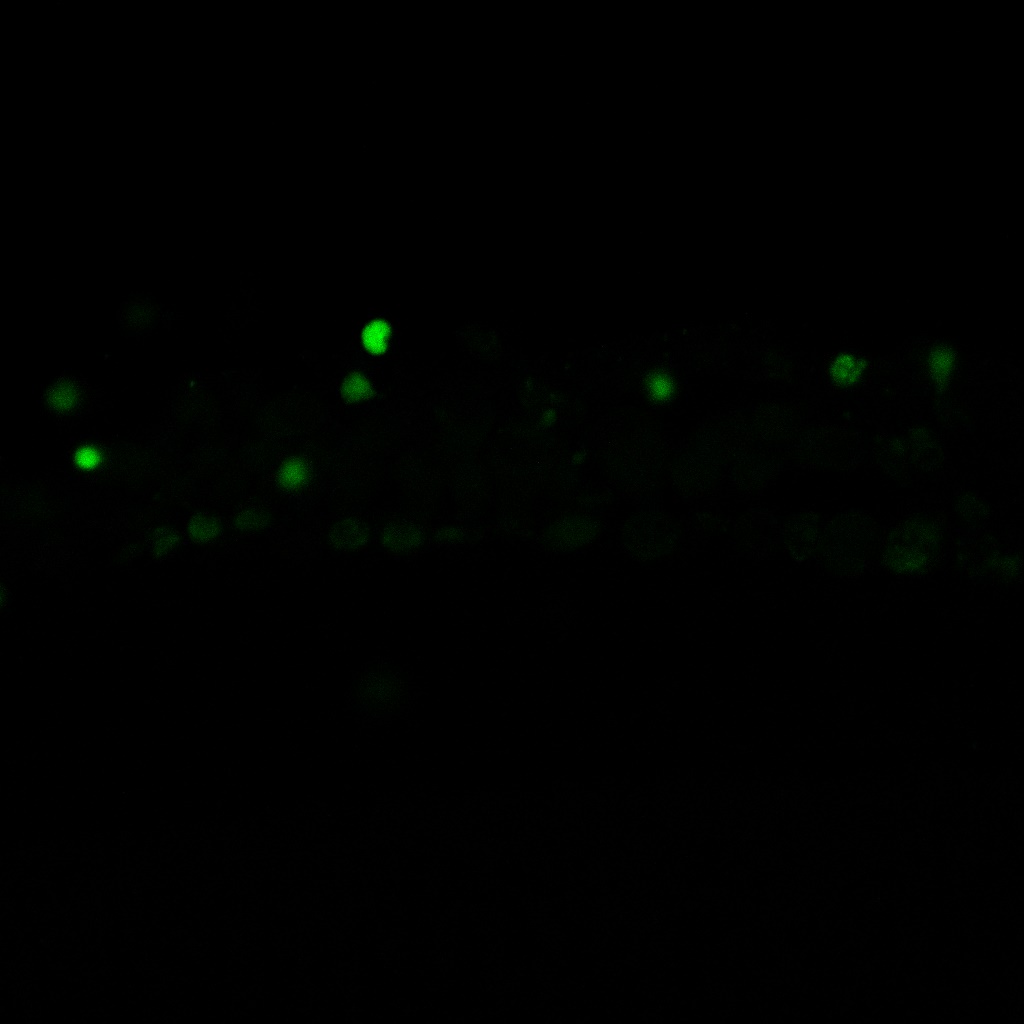

Supplement: Supplementary file 3 [file Data_Sheet_3.ZIP › Original data Fig. 4-7/Fig. 4/10.jpg]

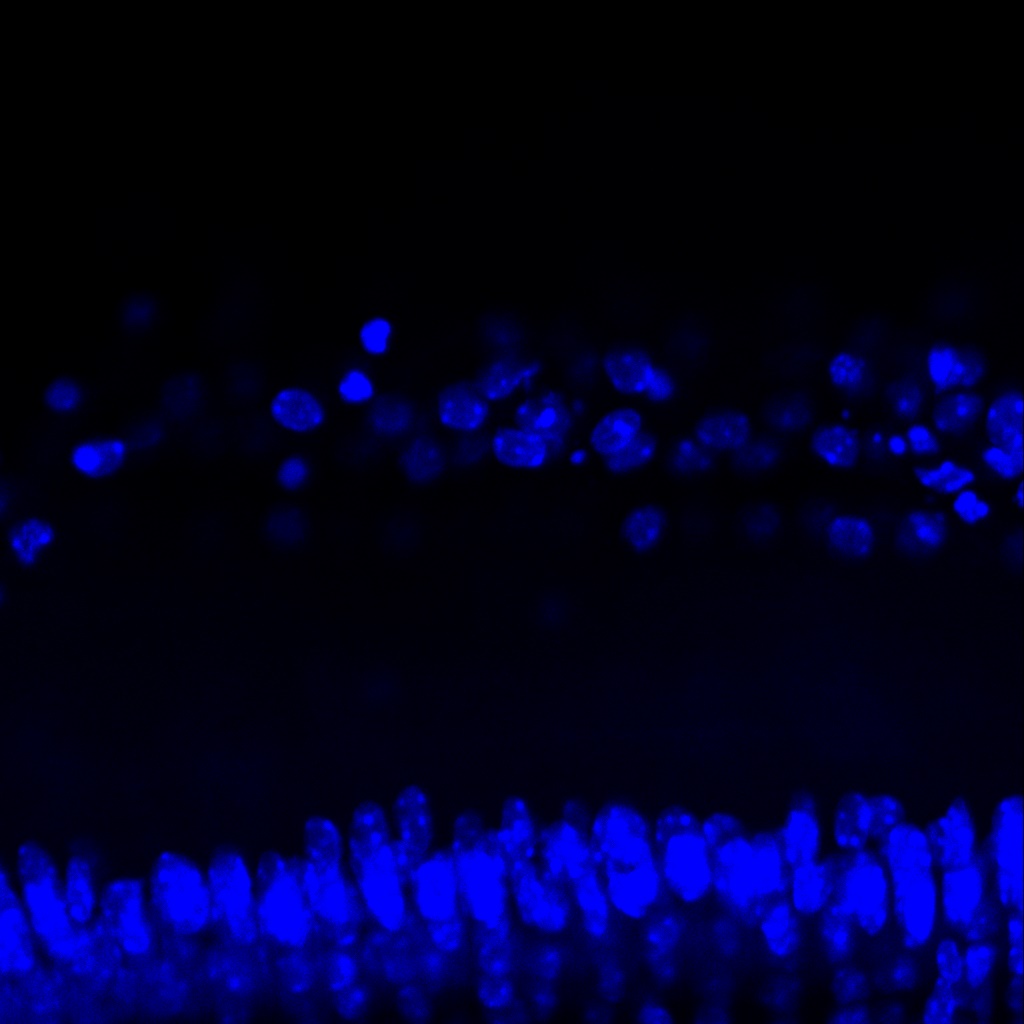

Supplement: Supplementary file 3 [file Data_Sheet_3.ZIP › Original data Fig. 4-7/Fig. 4/11.jpg]

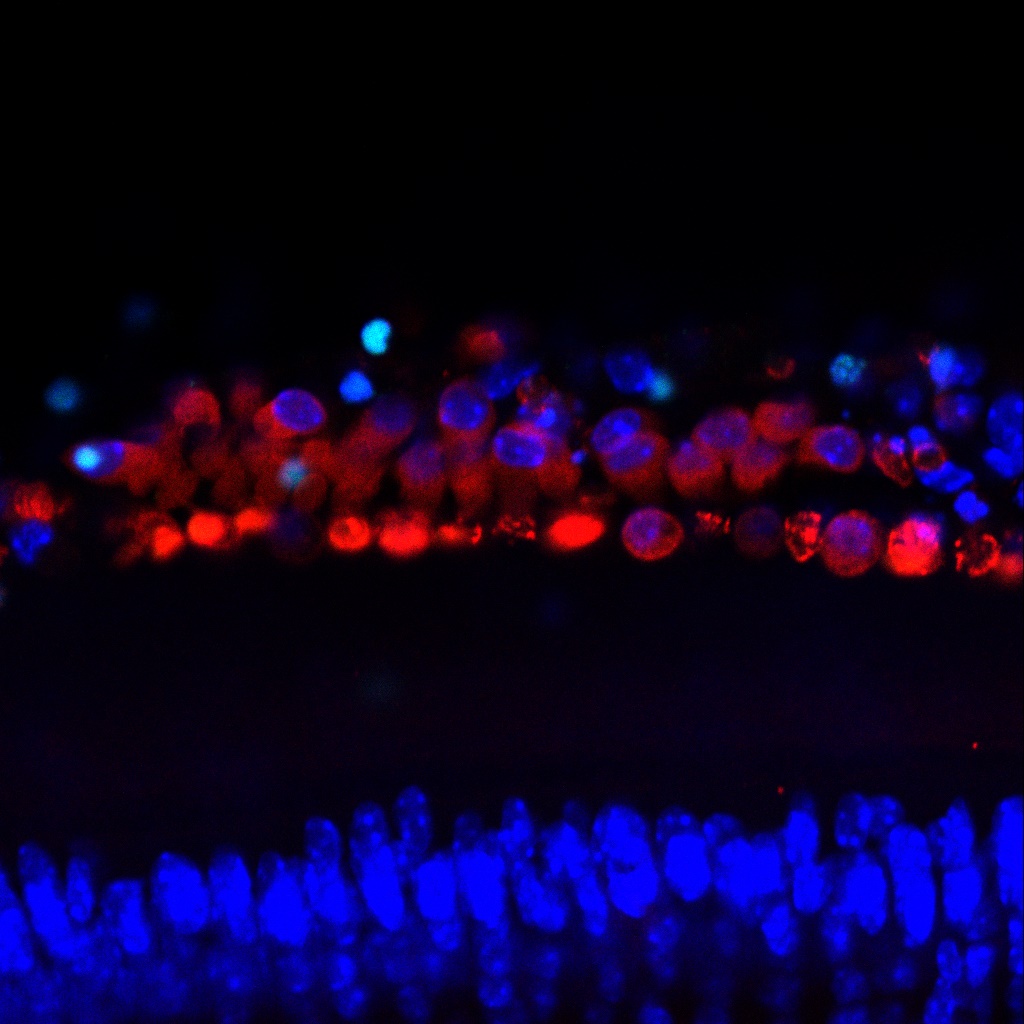

Supplement: Supplementary file 3 [file Data_Sheet_3.ZIP › Original data Fig. 4-7/Fig. 4/12.jpg]

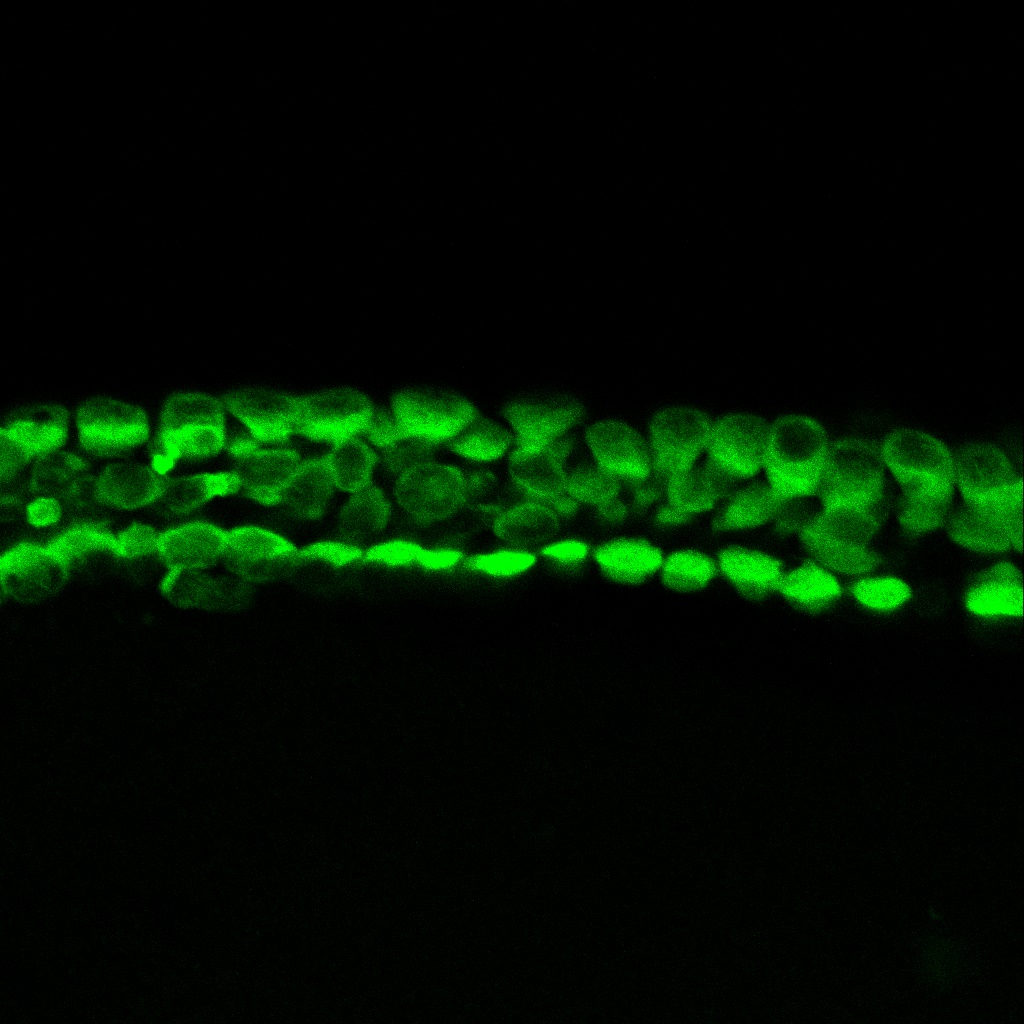

Supplement: Supplementary file 3 [file Data_Sheet_3.ZIP › Original data Fig. 4-7/Fig. 4/13.jpg]

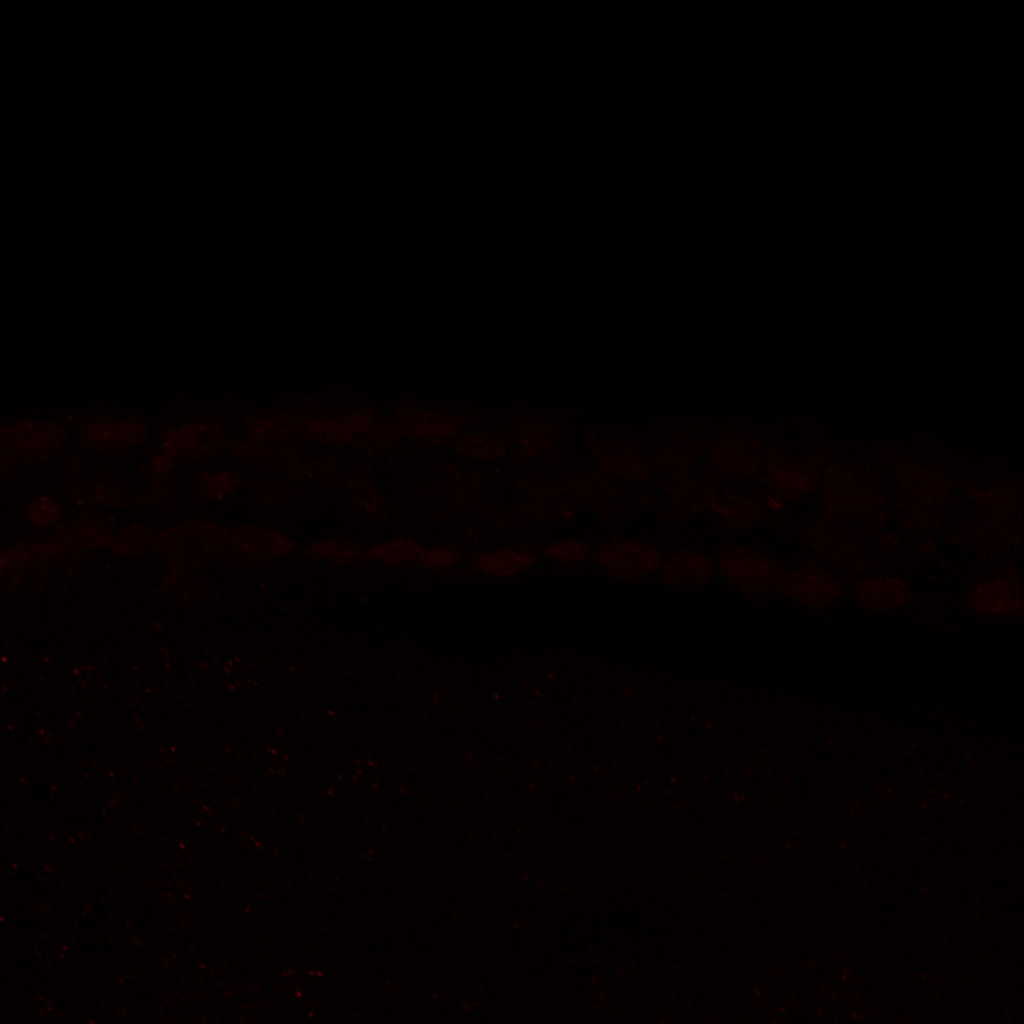

Supplement: Supplementary file 3 [file Data_Sheet_3.ZIP › Original data Fig. 4-7/Fig. 4/14.jpg]

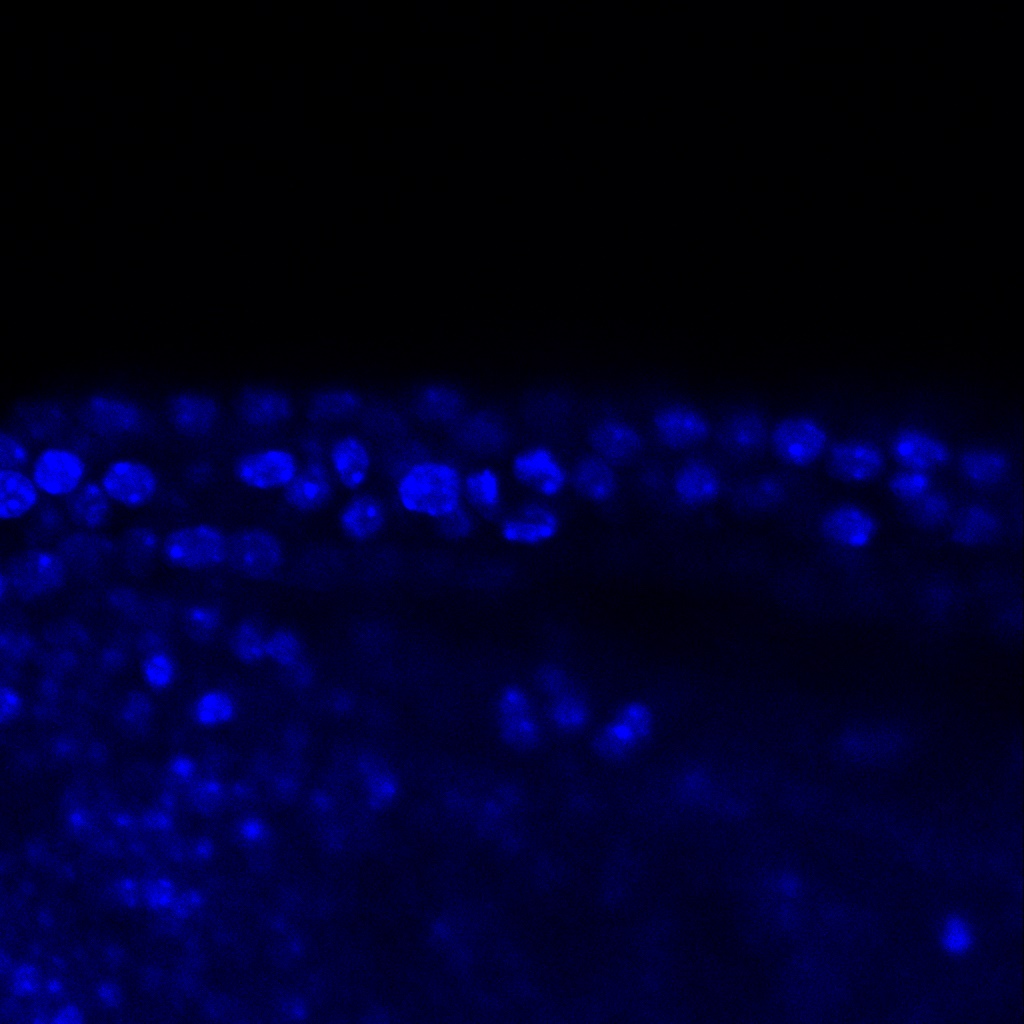

Supplement: Supplementary file 3 [file Data_Sheet_3.ZIP › Original data Fig. 4-7/Fig. 4/15.jpg]

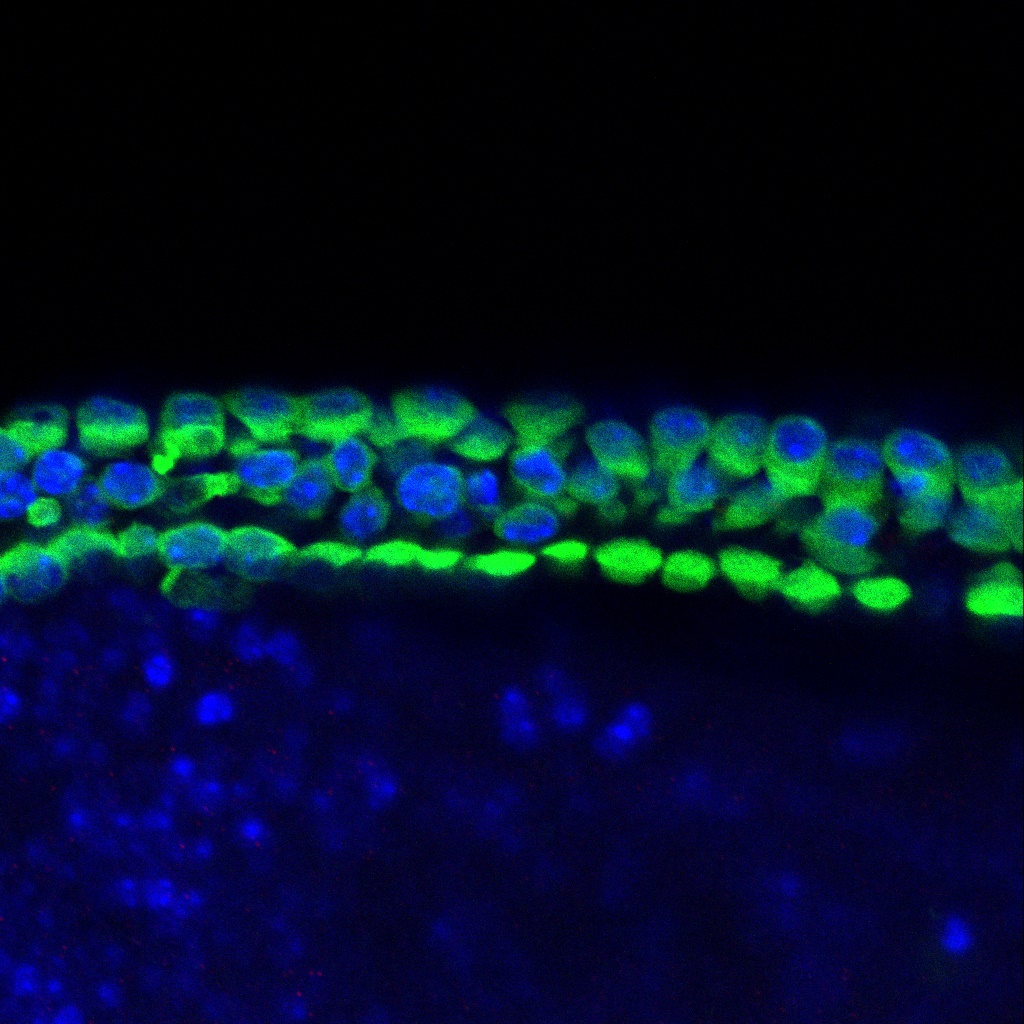

Supplement: Supplementary file 3 [file Data_Sheet_3.ZIP › Original data Fig. 4-7/Fig. 4/16.jpg]

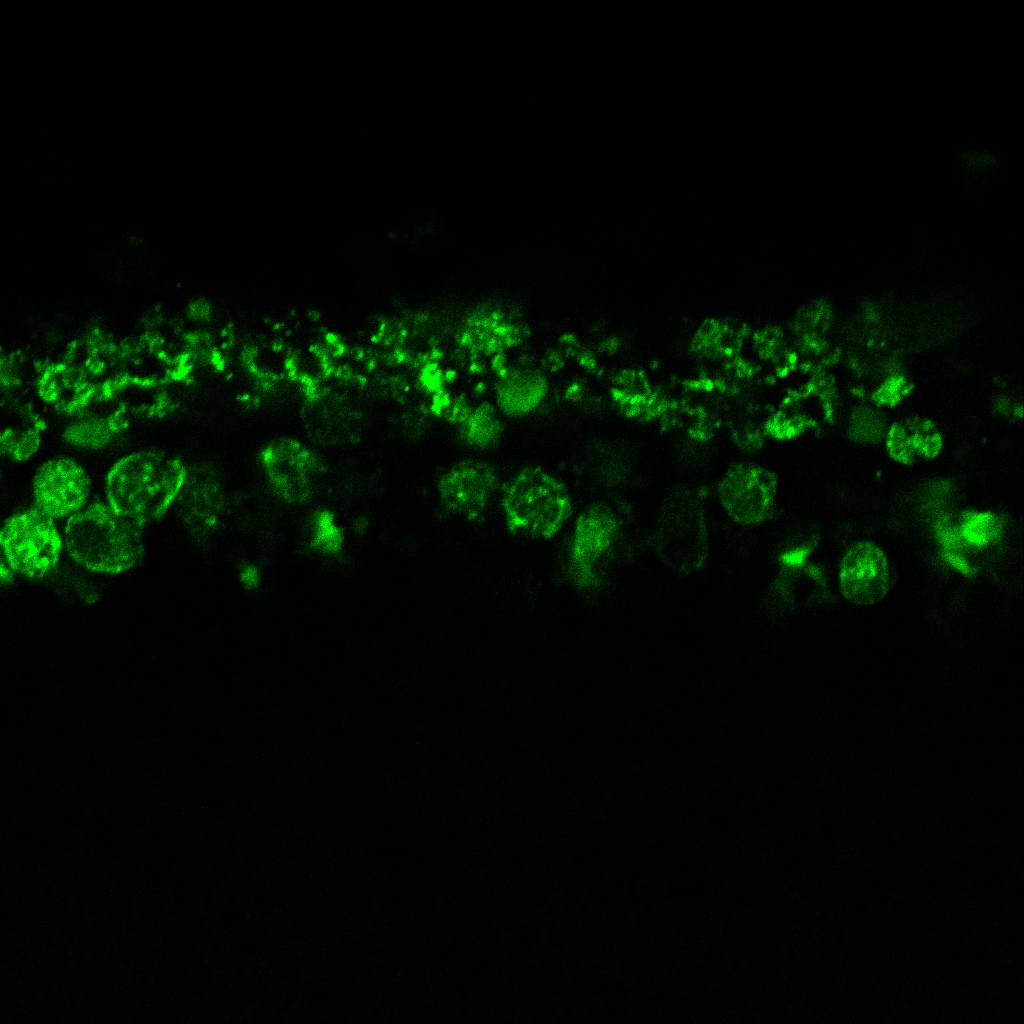

Supplement: Supplementary file 3 [file Data_Sheet_3.ZIP › Original data Fig. 4-7/Fig. 4/17.jpg]

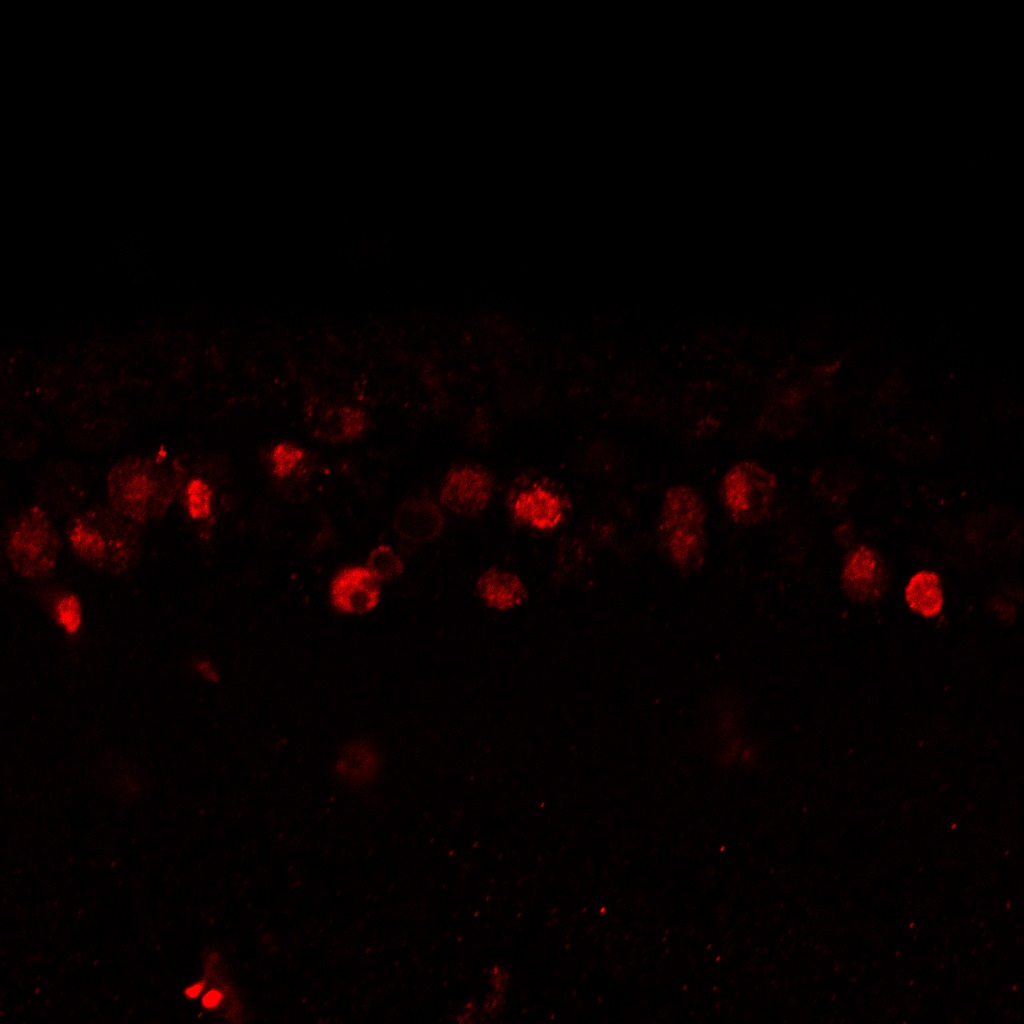

Supplement: Supplementary file 3 [file Data_Sheet_3.ZIP › Original data Fig. 4-7/Fig. 4/18.jpg]

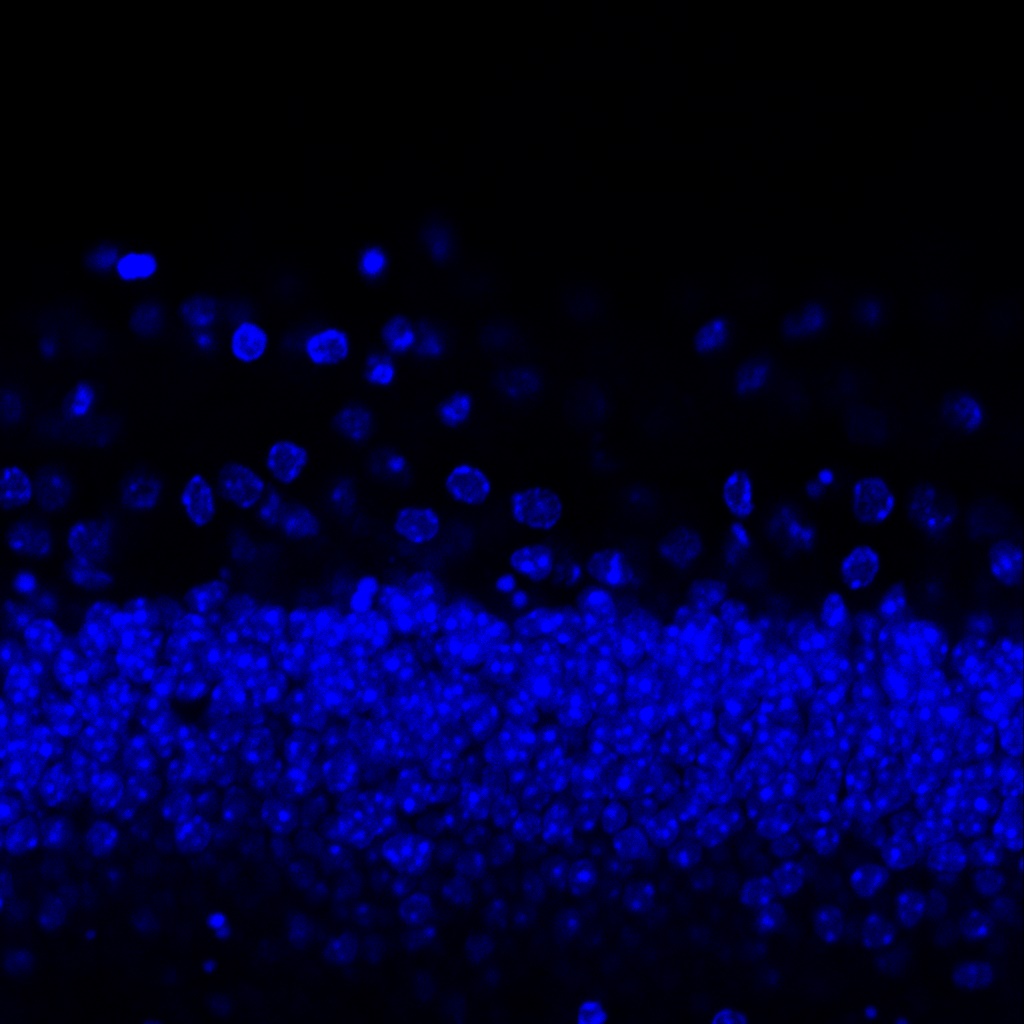

Supplement: Supplementary file 3 [file Data_Sheet_3.ZIP › Original data Fig. 4-7/Fig. 4/19.jpg]

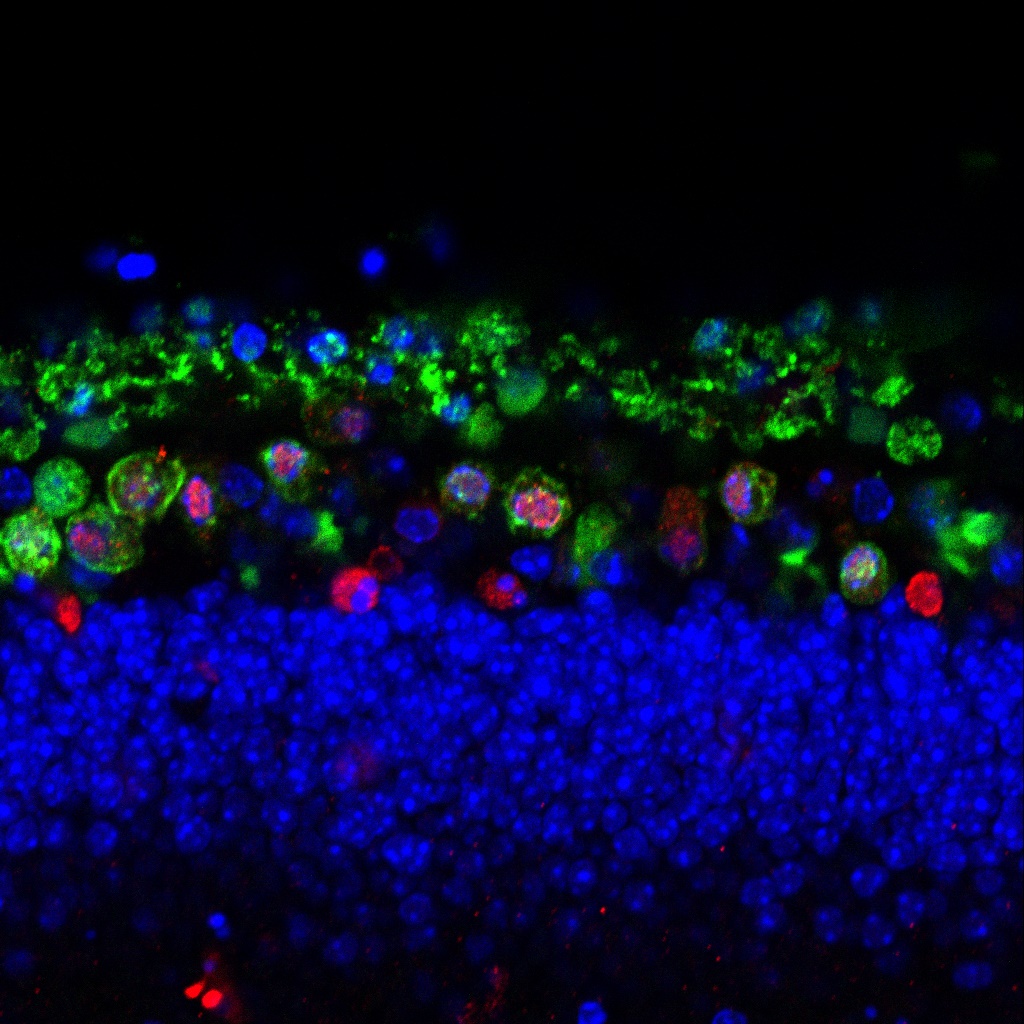

Supplement: Supplementary file 3 [file Data_Sheet_3.ZIP › Original data Fig. 4-7/Fig. 4/20.jpg]

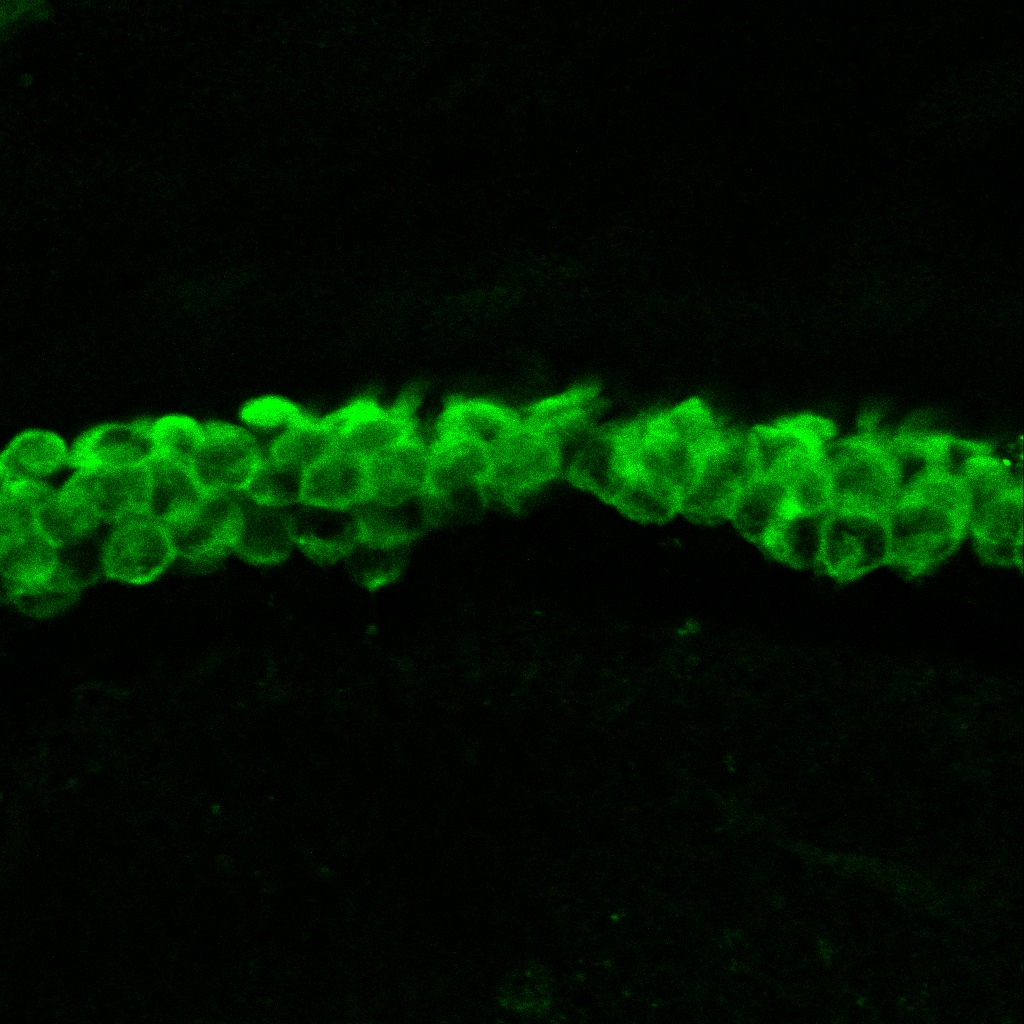

Supplement: Supplementary file 3 [file Data_Sheet_3.ZIP › Original data Fig. 4-7/Fig. 4/21.jpg]

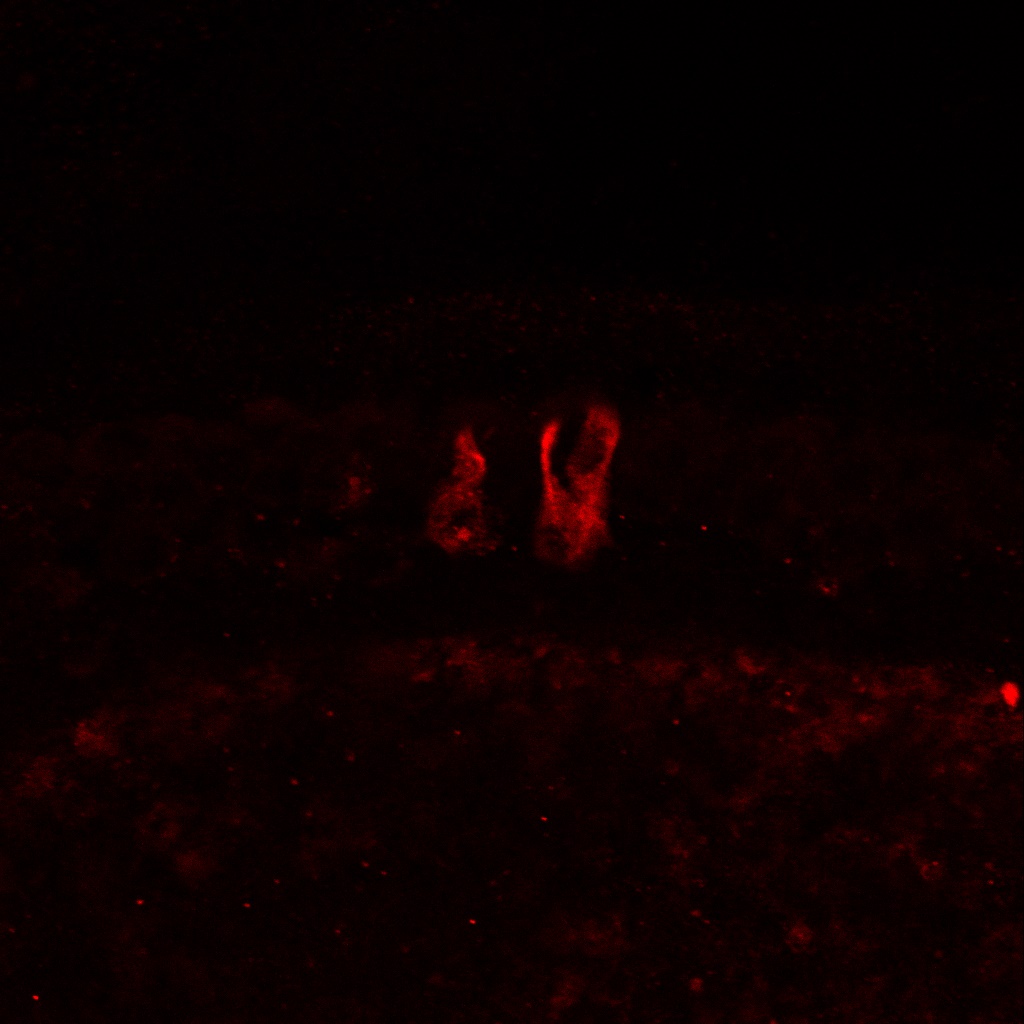

Supplement: Supplementary file 3 [file Data_Sheet_3.ZIP › Original data Fig. 4-7/Fig. 4/22.jpg]

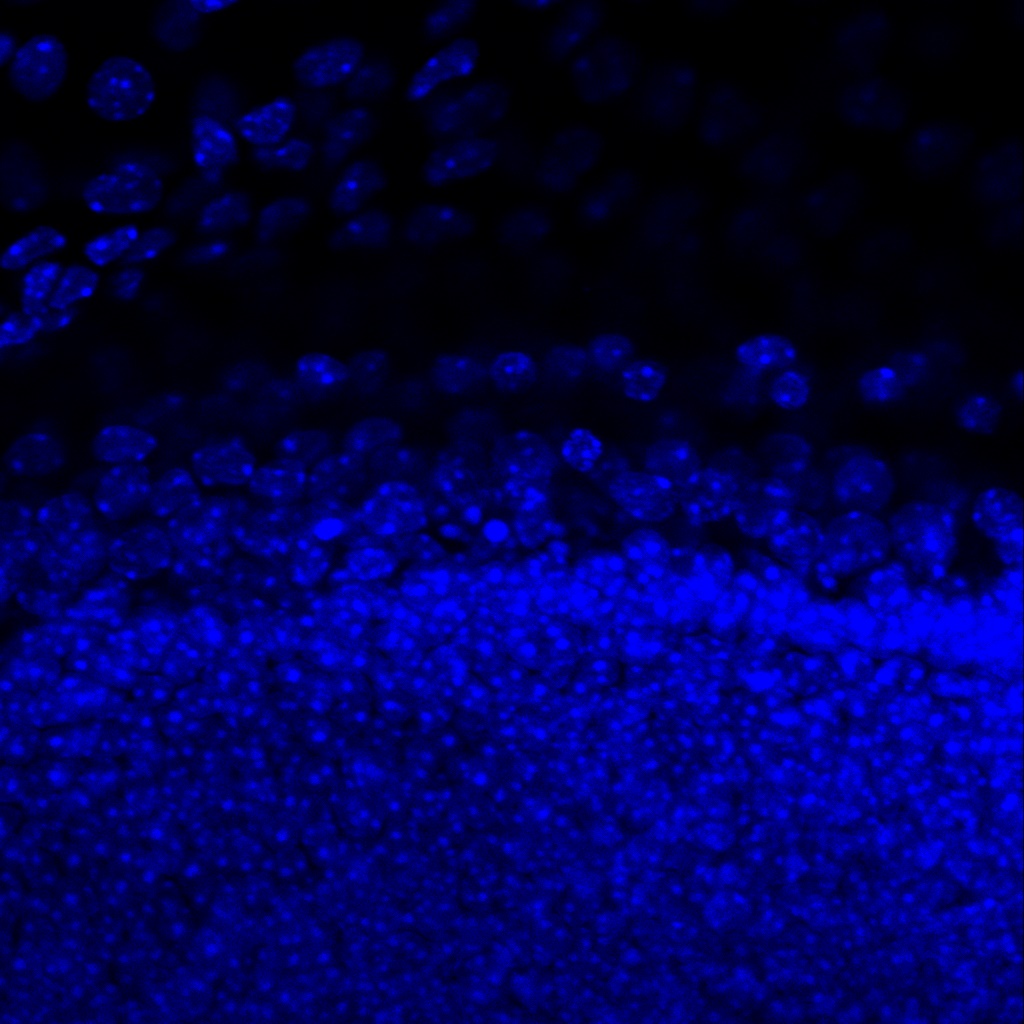

Supplement: Supplementary file 3 [file Data_Sheet_3.ZIP › Original data Fig. 4-7/Fig. 4/23.jpg]

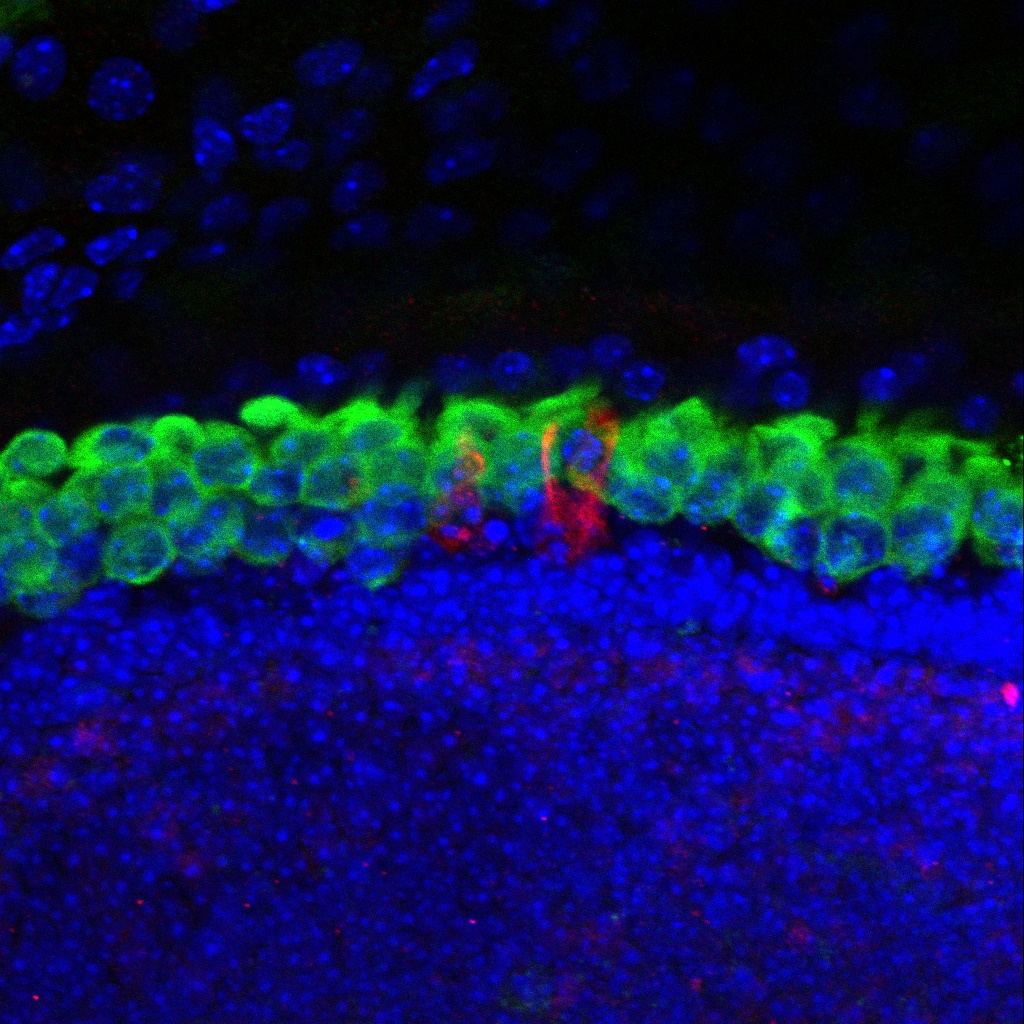

Supplement: Supplementary file 3 [file Data_Sheet_3.ZIP › Original data Fig. 4-7/Fig. 4/24.jpg]

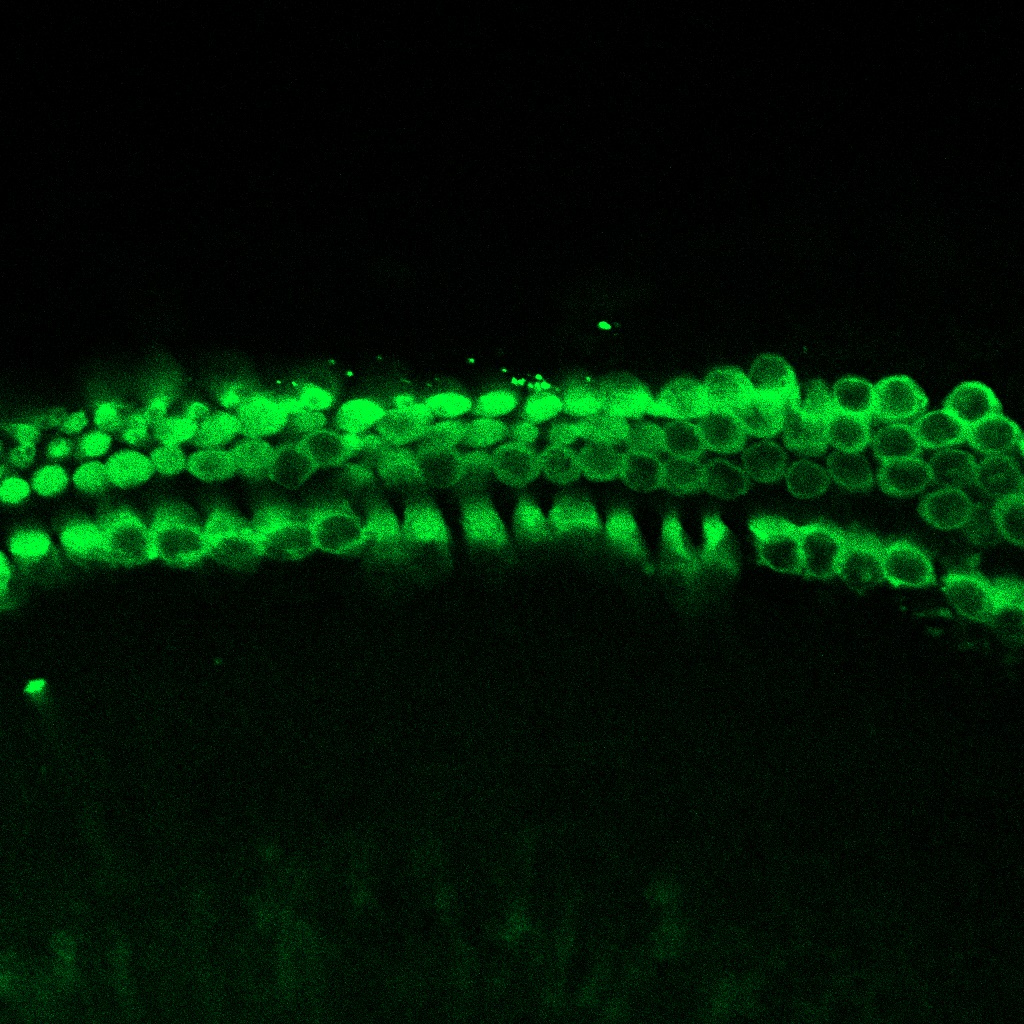

Supplement: Supplementary file 3 [file Data_Sheet_3.ZIP › Original data Fig. 4-7/Fig. 5/1.jpg]

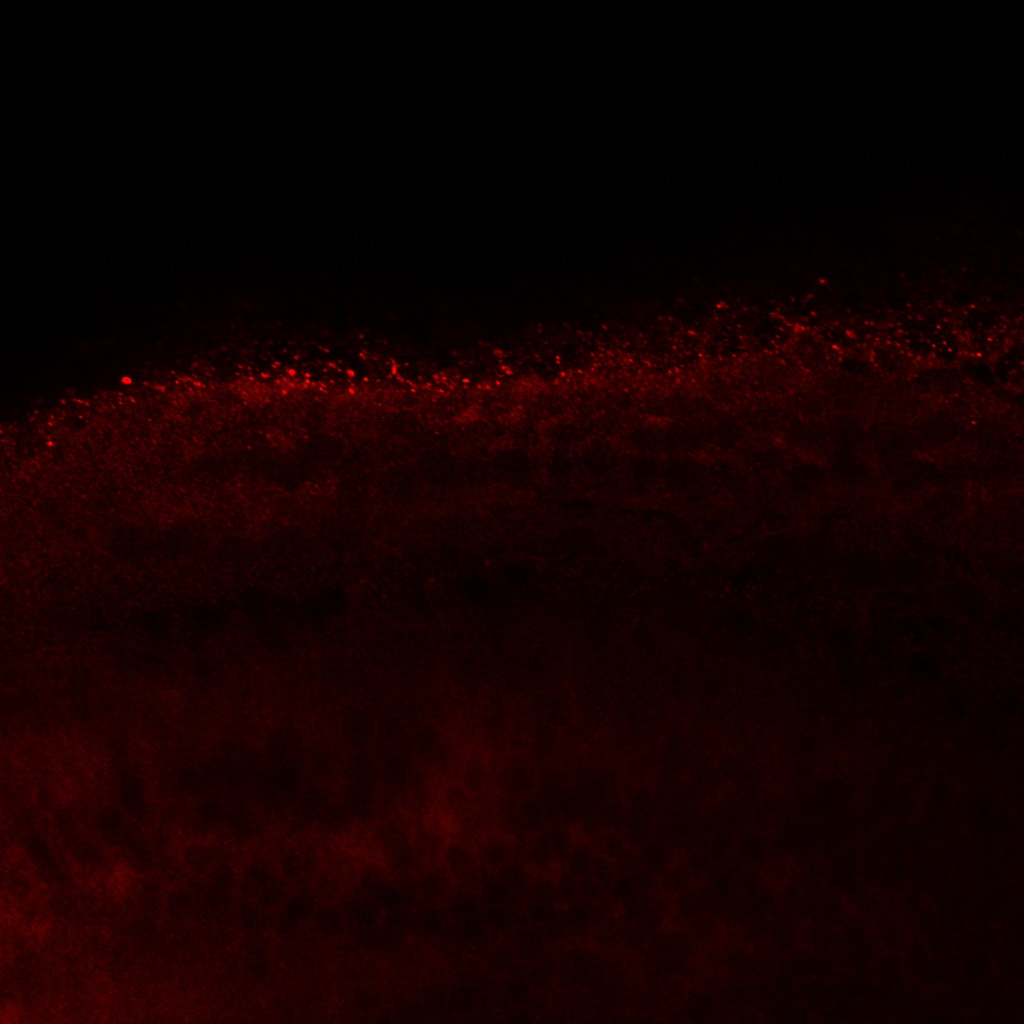

Supplement: Supplementary file 3 [file Data_Sheet_3.ZIP › Original data Fig. 4-7/Fig. 5/2.jpg]

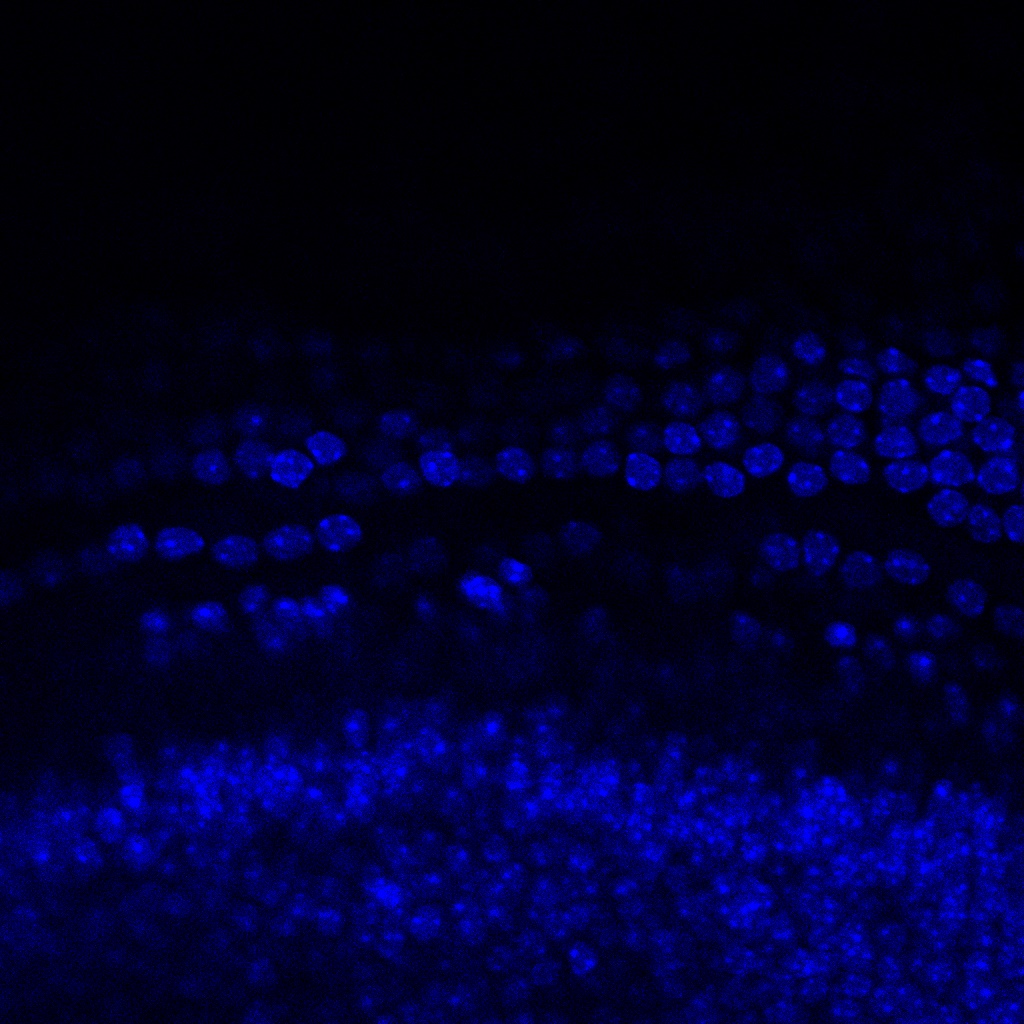

Supplement: Supplementary file 3 [file Data_Sheet_3.ZIP › Original data Fig. 4-7/Fig. 5/3.jpg]

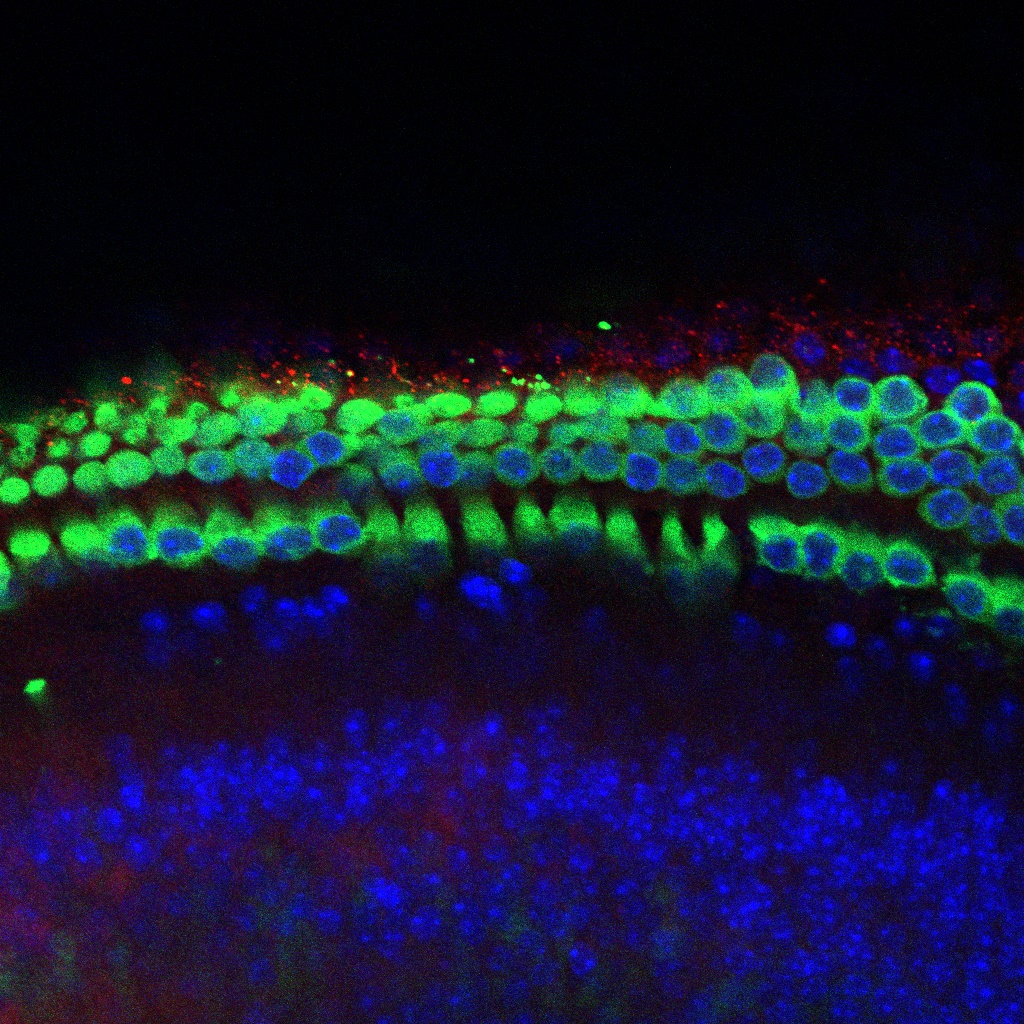

Supplement: Supplementary file 3 [file Data_Sheet_3.ZIP › Original data Fig. 4-7/Fig. 5/4.jpg]

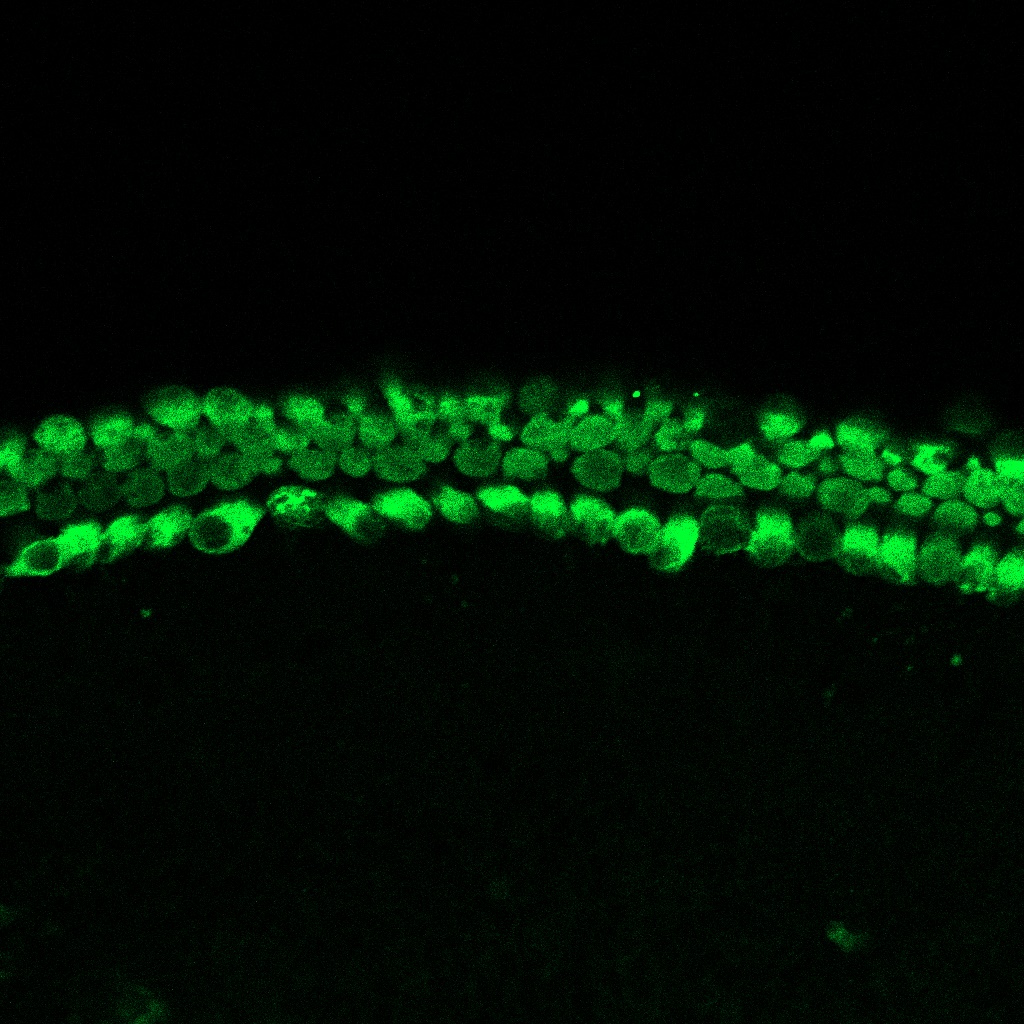

Supplement: Supplementary file 3 [file Data_Sheet_3.ZIP › Original data Fig. 4-7/Fig. 5/5.jpg]

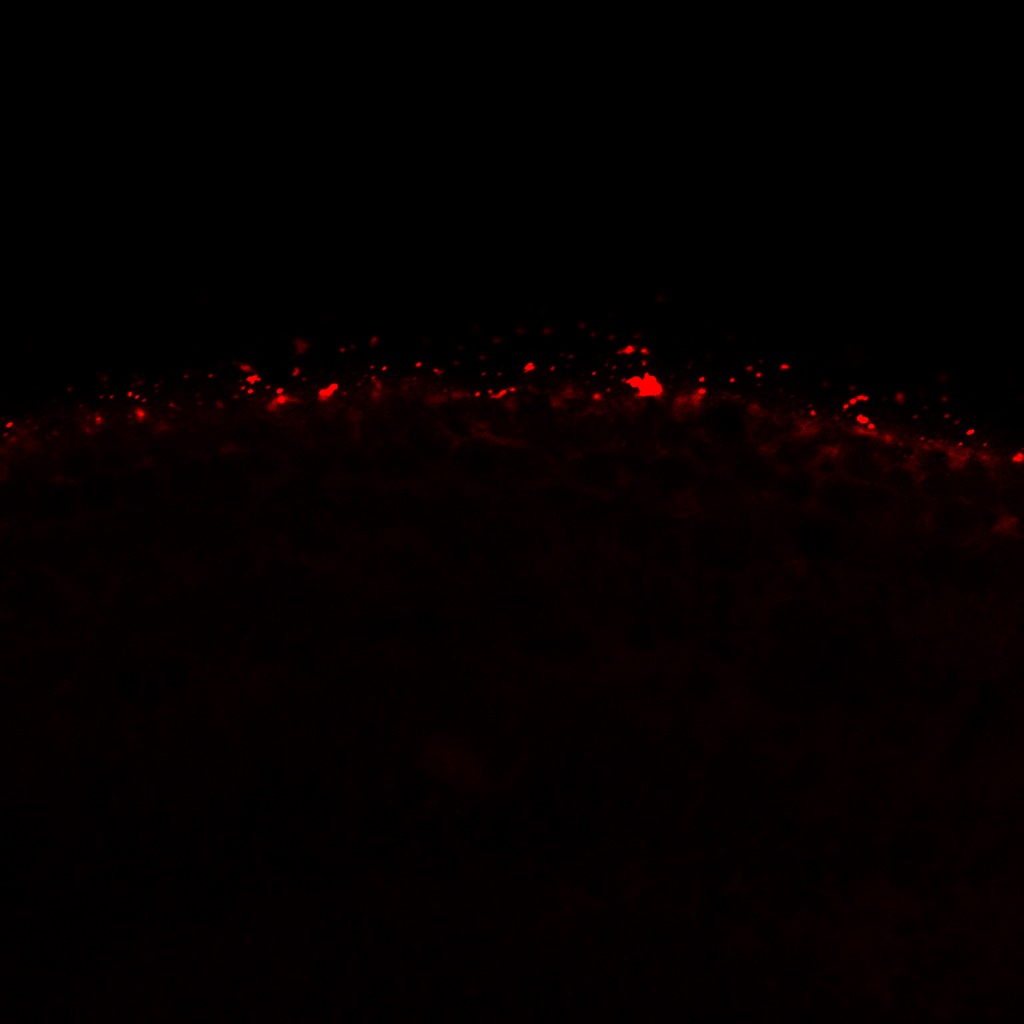

Supplement: Supplementary file 3 [file Data_Sheet_3.ZIP › Original data Fig. 4-7/Fig. 5/6.jpg]

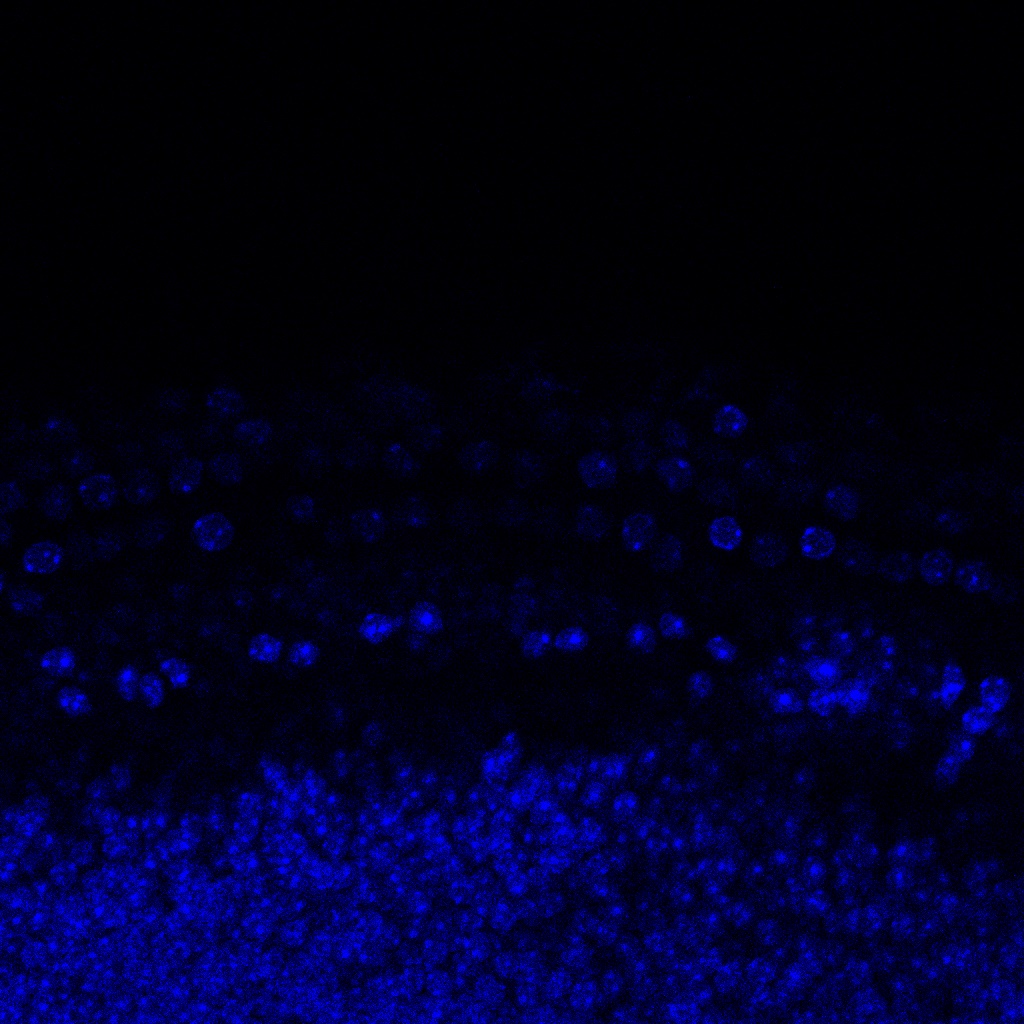

Supplement: Supplementary file 3 [file Data_Sheet_3.ZIP › Original data Fig. 4-7/Fig. 5/7.jpg]

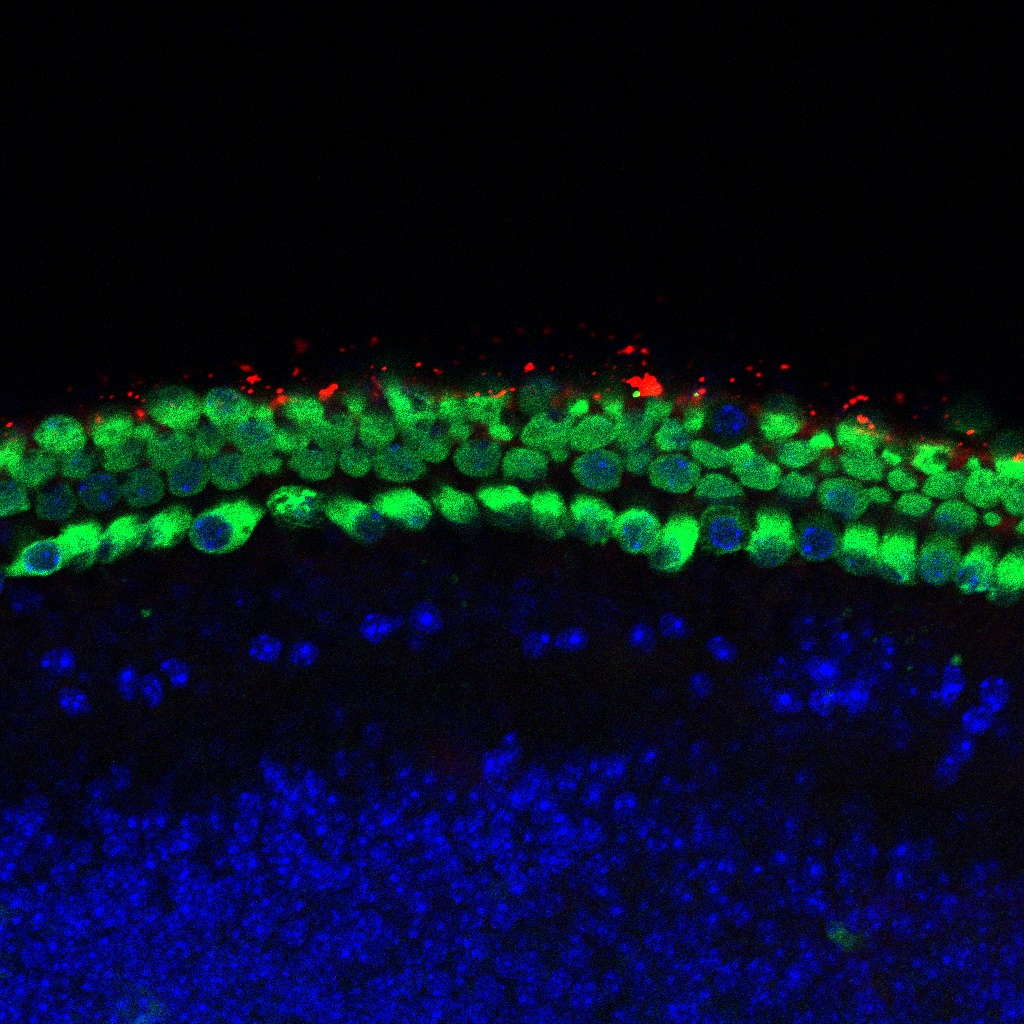

Supplement: Supplementary file 3 [file Data_Sheet_3.ZIP › Original data Fig. 4-7/Fig. 5/8.jpg]

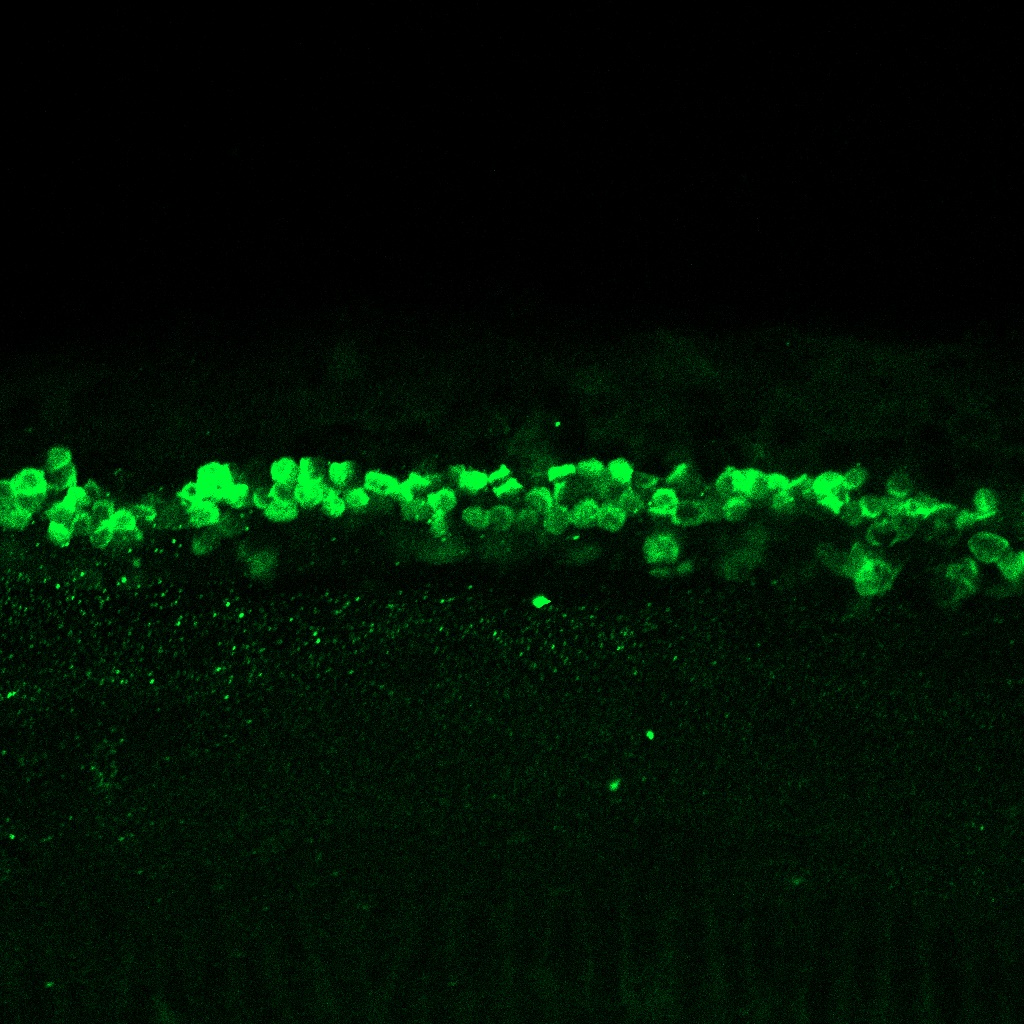

Supplement: Supplementary file 3 [file Data_Sheet_3.ZIP › Original data Fig. 4-7/Fig. 5/9.jpg]

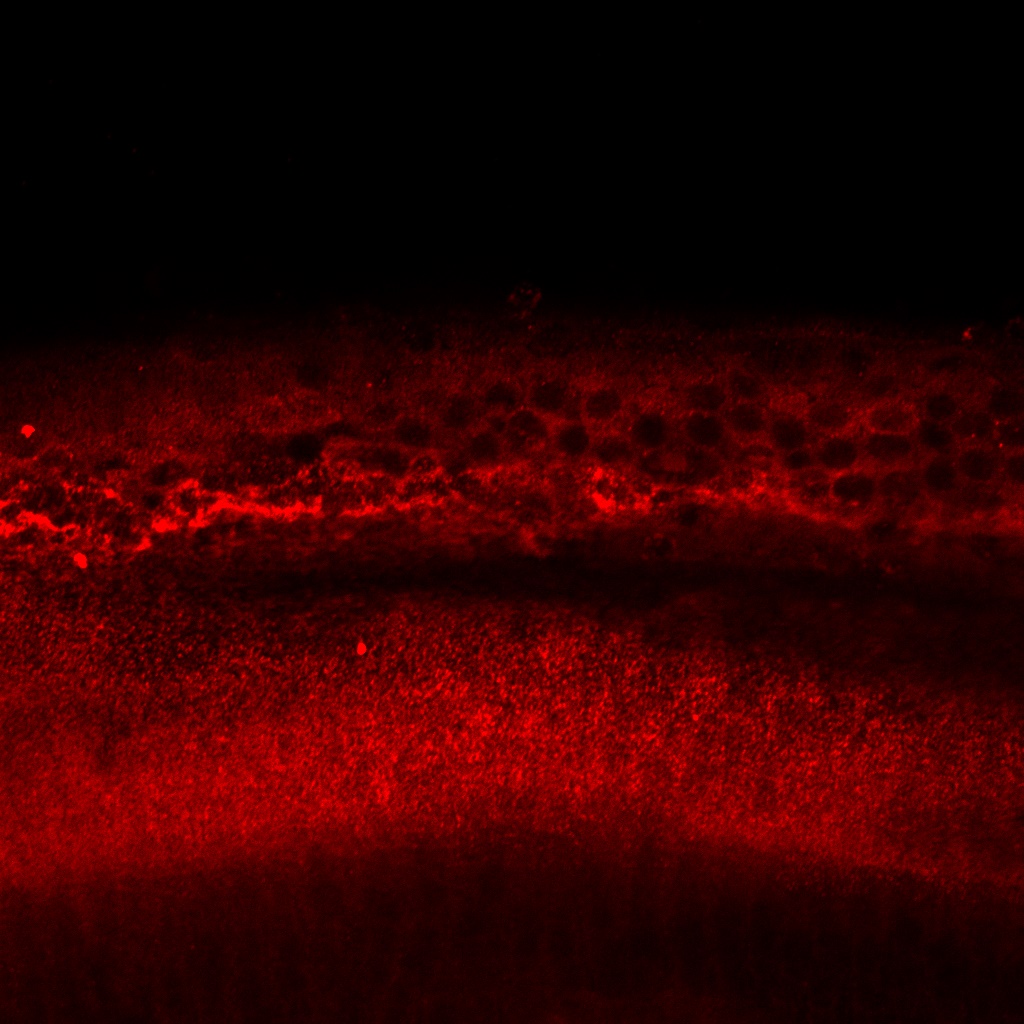

Supplement: Supplementary file 3 [file Data_Sheet_3.ZIP › Original data Fig. 4-7/Fig. 5/10.jpg]

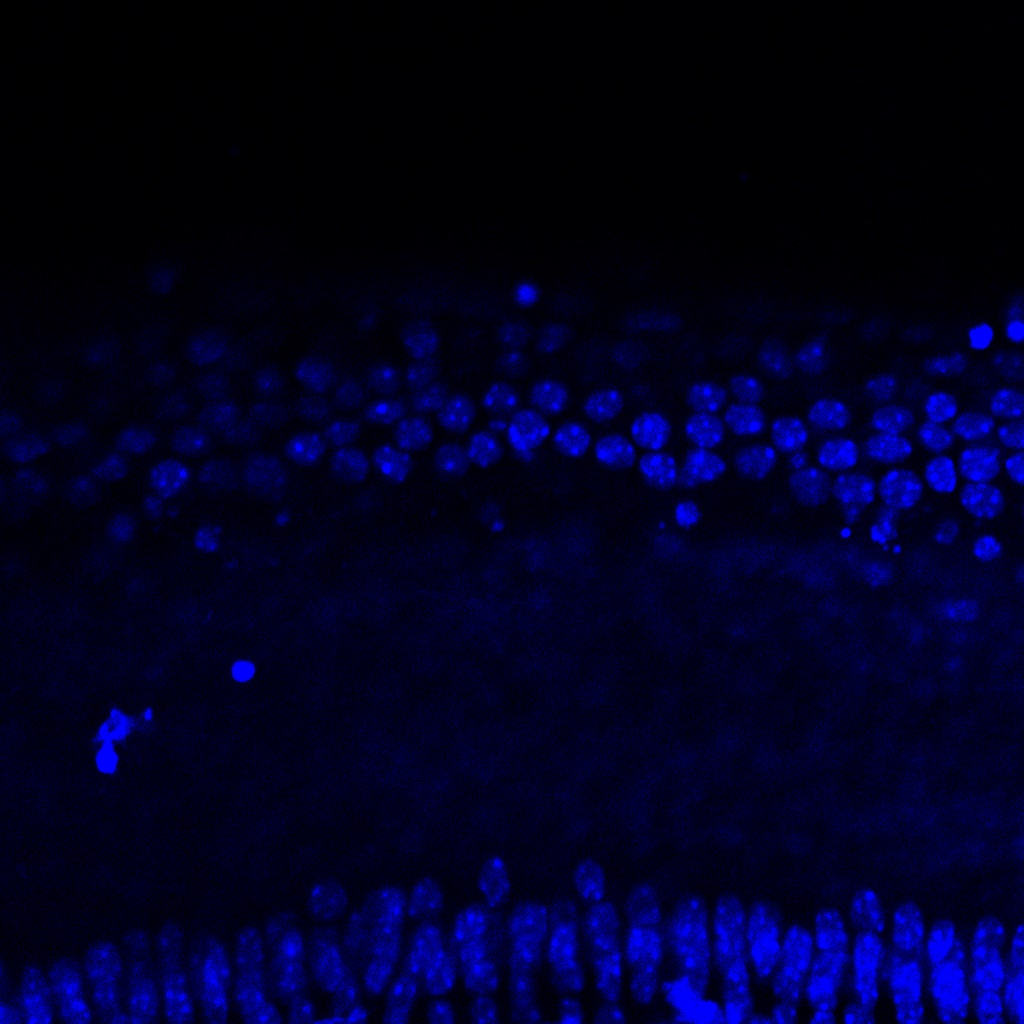

Supplement: Supplementary file 3 [file Data_Sheet_3.ZIP › Original data Fig. 4-7/Fig. 5/11.jpg]

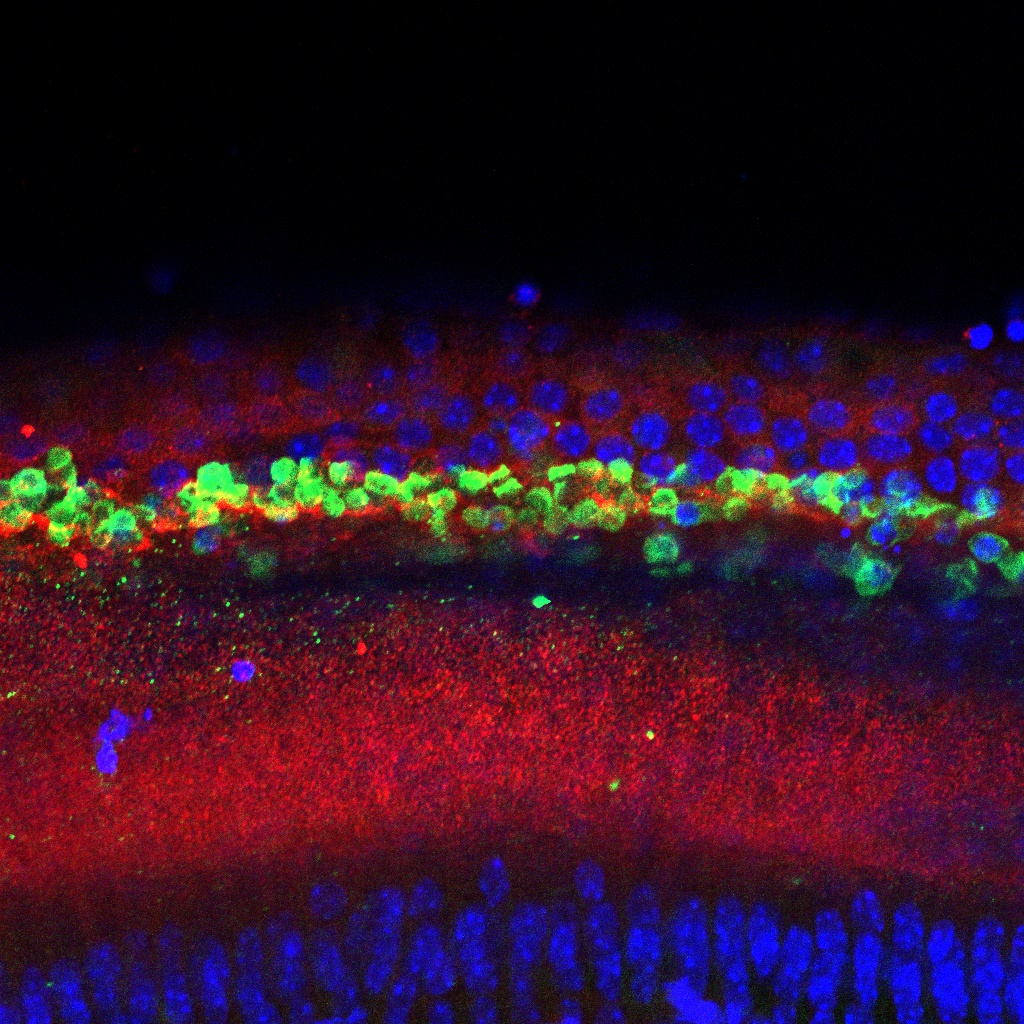

Supplement: Supplementary file 3 [file Data_Sheet_3.ZIP › Original data Fig. 4-7/Fig. 5/12.jpg]

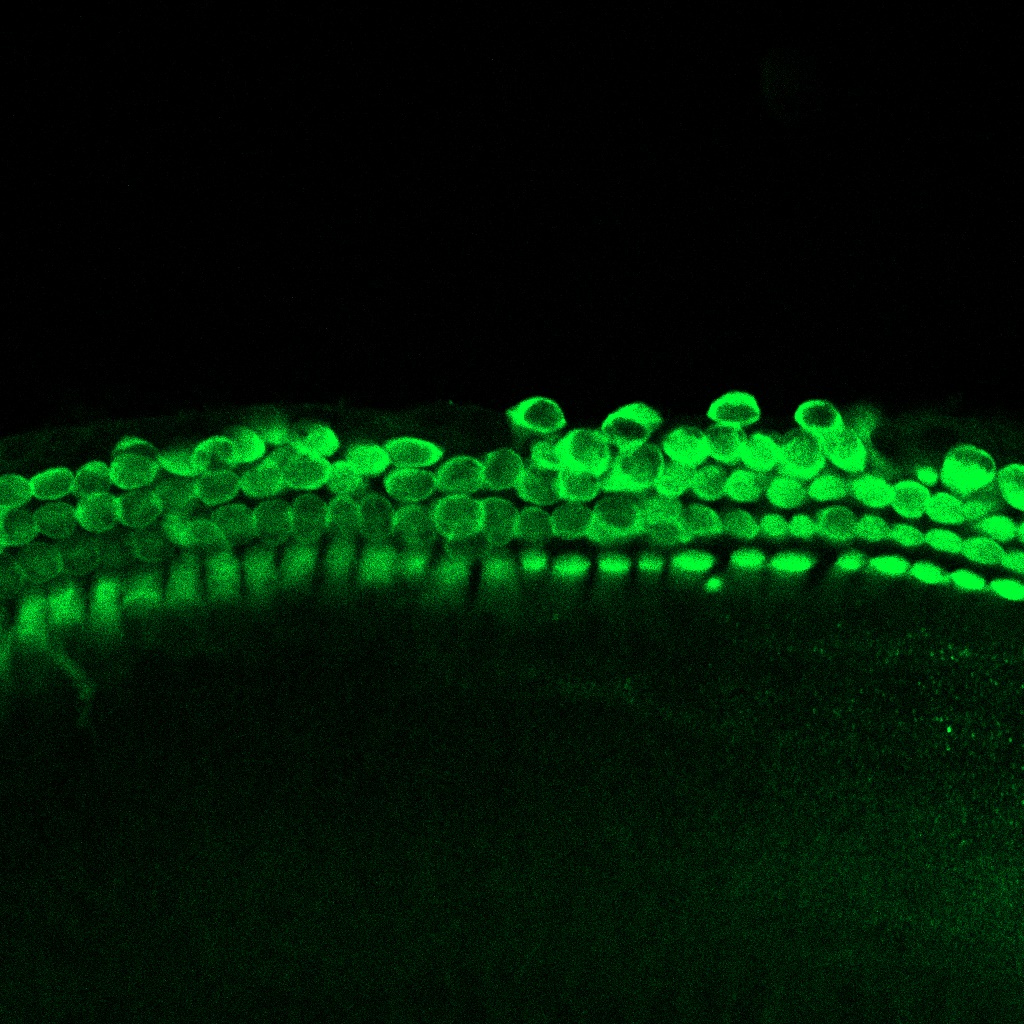

Supplement: Supplementary file 3 [file Data_Sheet_3.ZIP › Original data Fig. 4-7/Fig. 5/13.jpg]

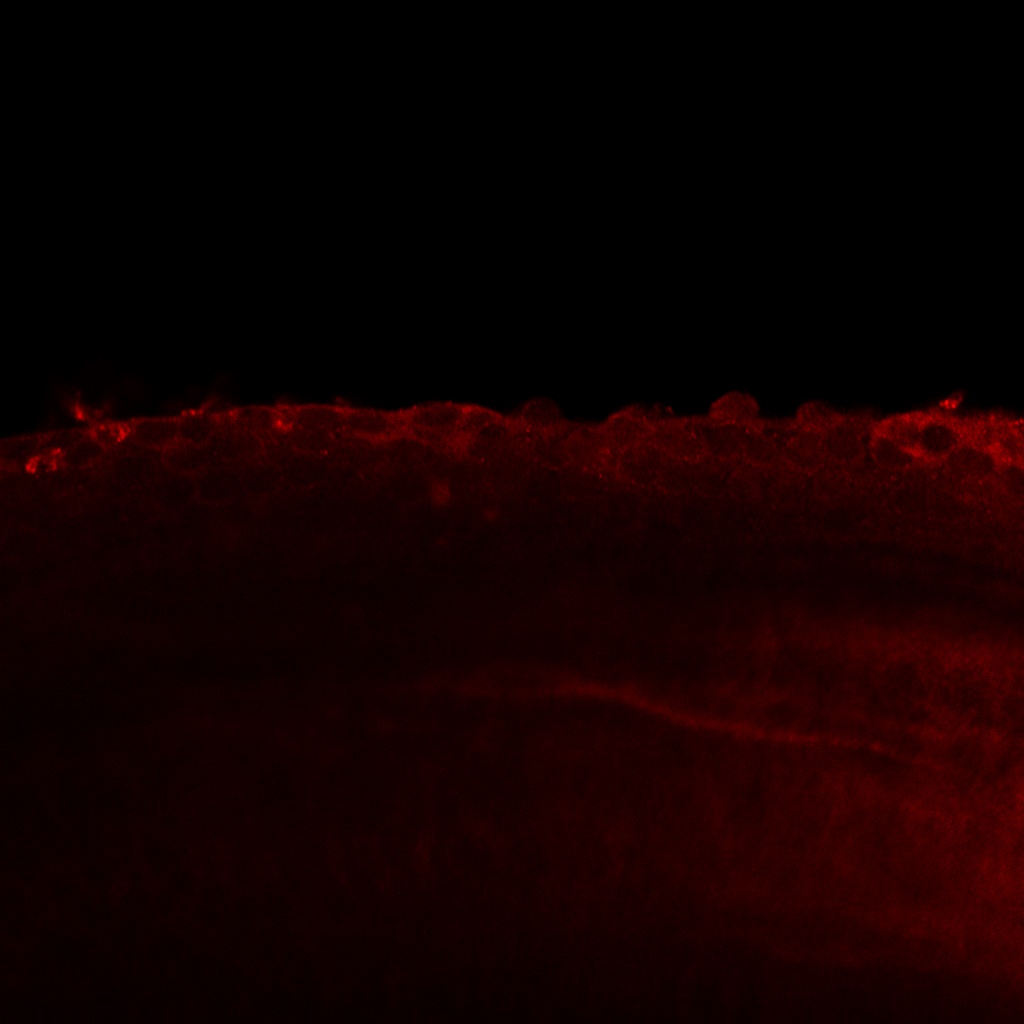

Supplement: Supplementary file 3 [file Data_Sheet_3.ZIP › Original data Fig. 4-7/Fig. 5/14.jpg]

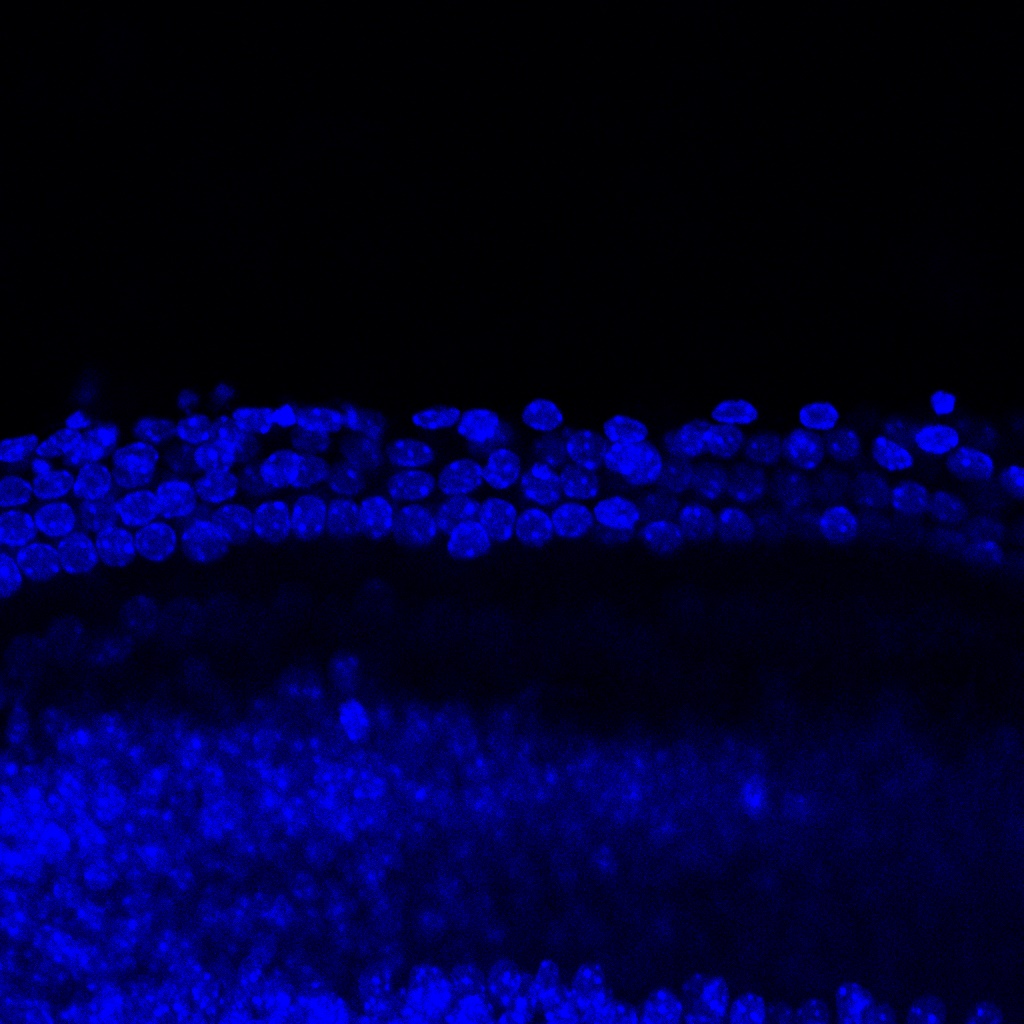

Supplement: Supplementary file 3 [file Data_Sheet_3.ZIP › Original data Fig. 4-7/Fig. 5/15.jpg]

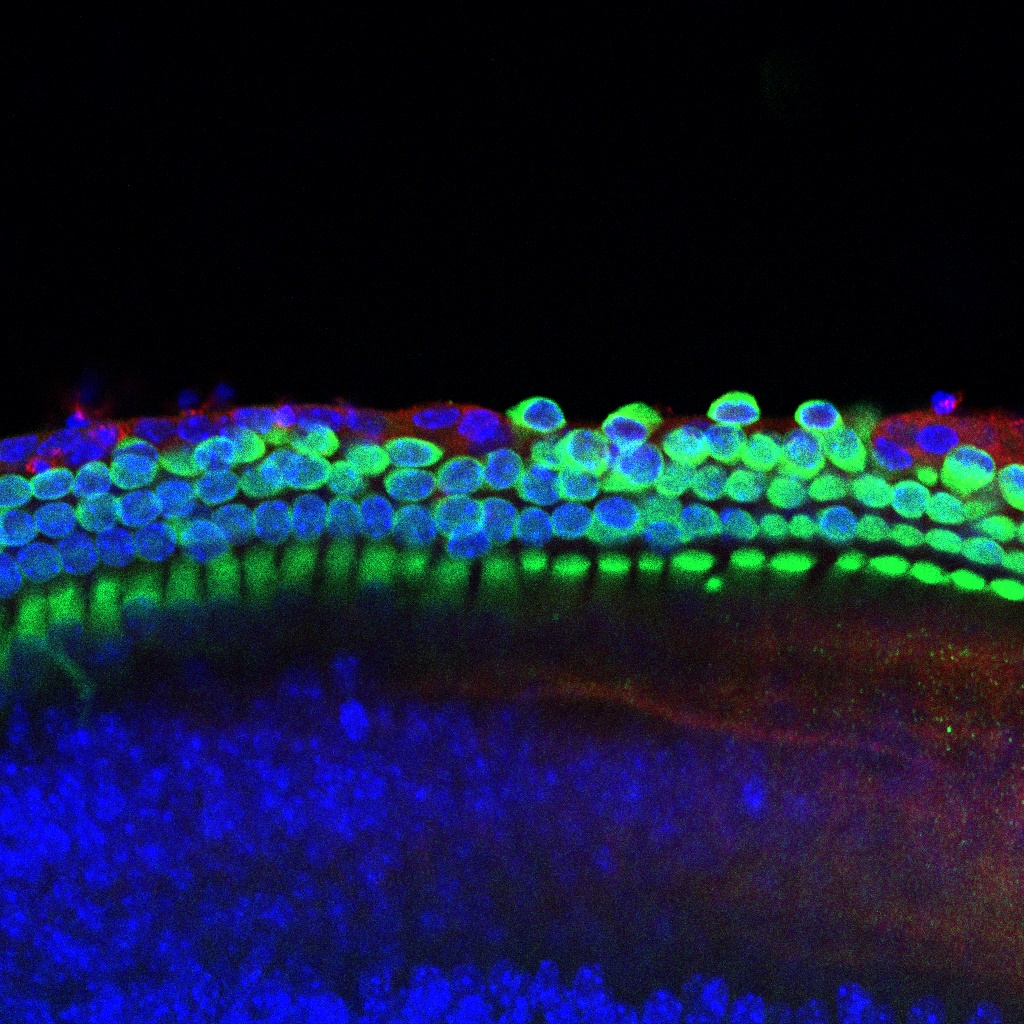

Supplement: Supplementary file 3 [file Data_Sheet_3.ZIP › Original data Fig. 4-7/Fig. 5/16.jpg]

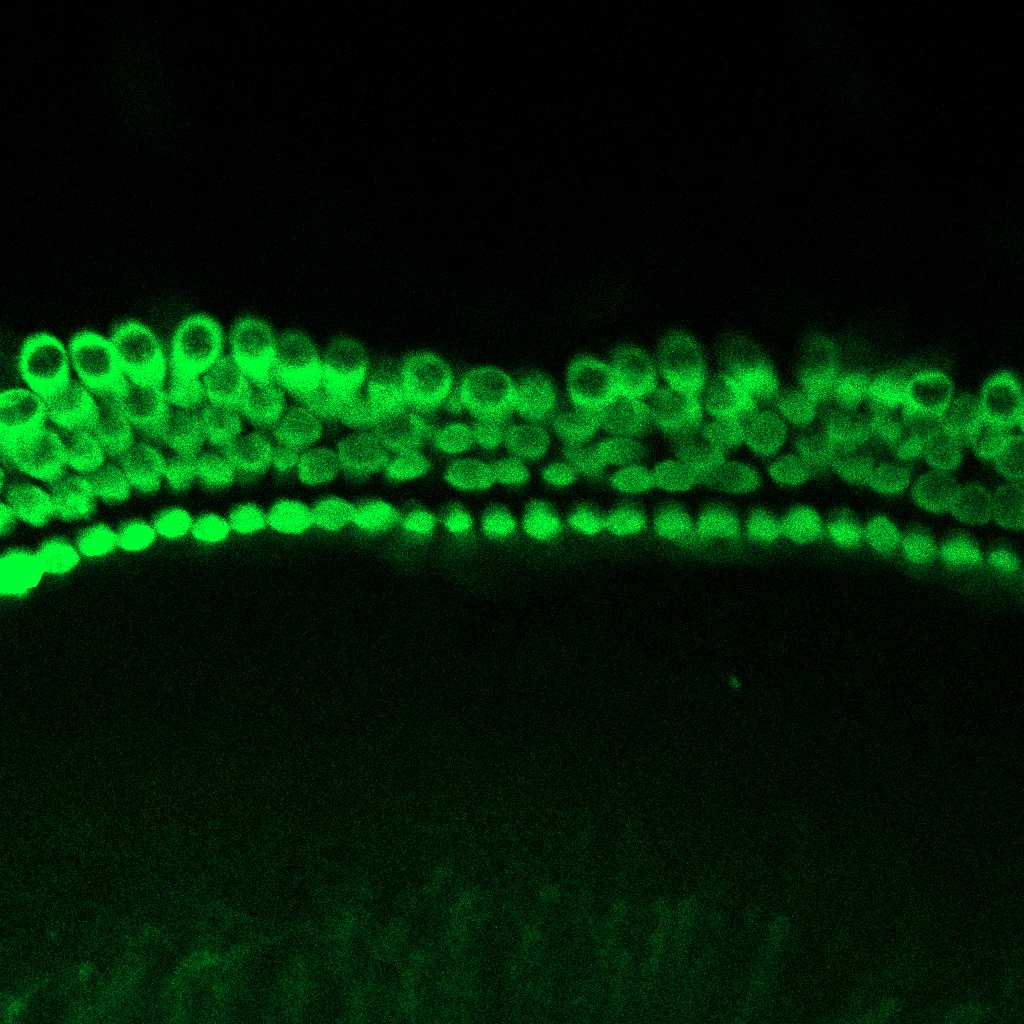

Supplement: Supplementary file 3 [file Data_Sheet_3.ZIP › Original data Fig. 4-7/Fig. 5/17.jpg]

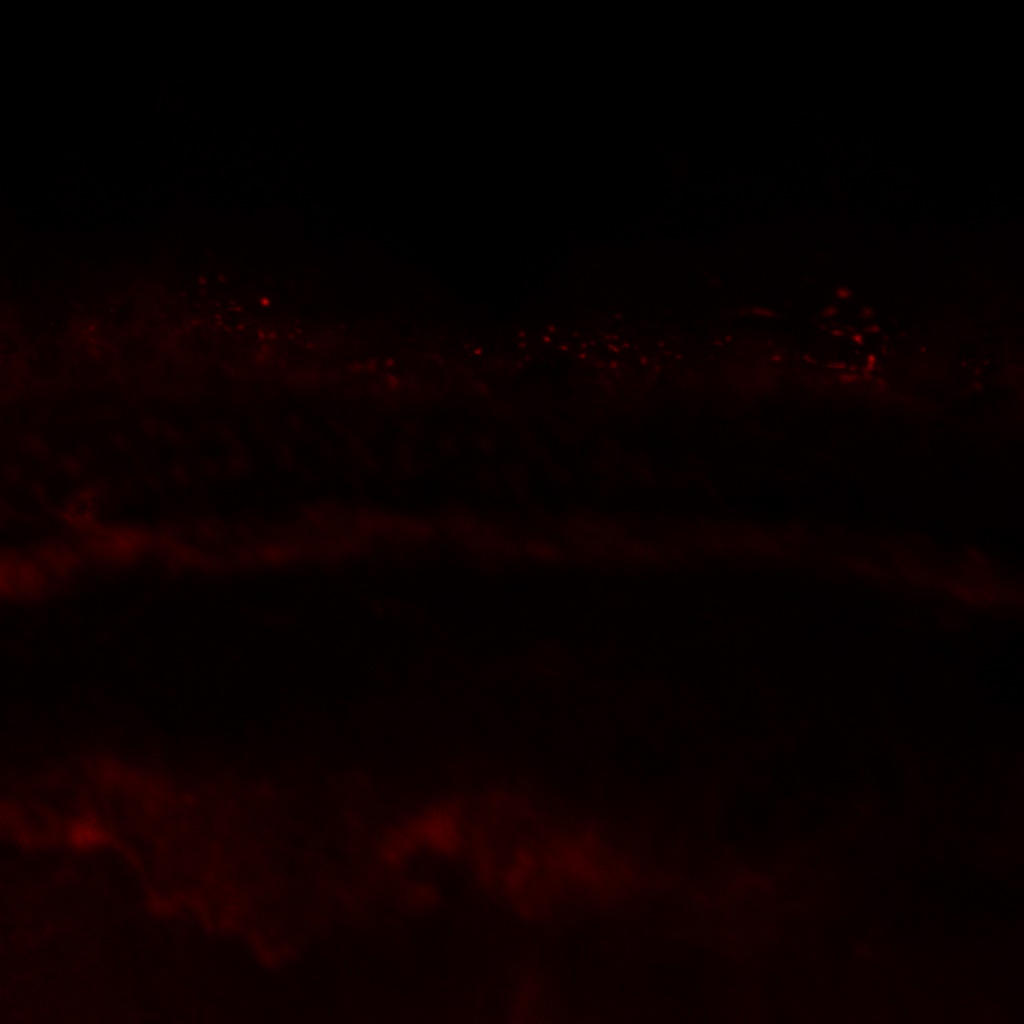

Supplement: Supplementary file 3 [file Data_Sheet_3.ZIP › Original data Fig. 4-7/Fig. 5/18.jpg]

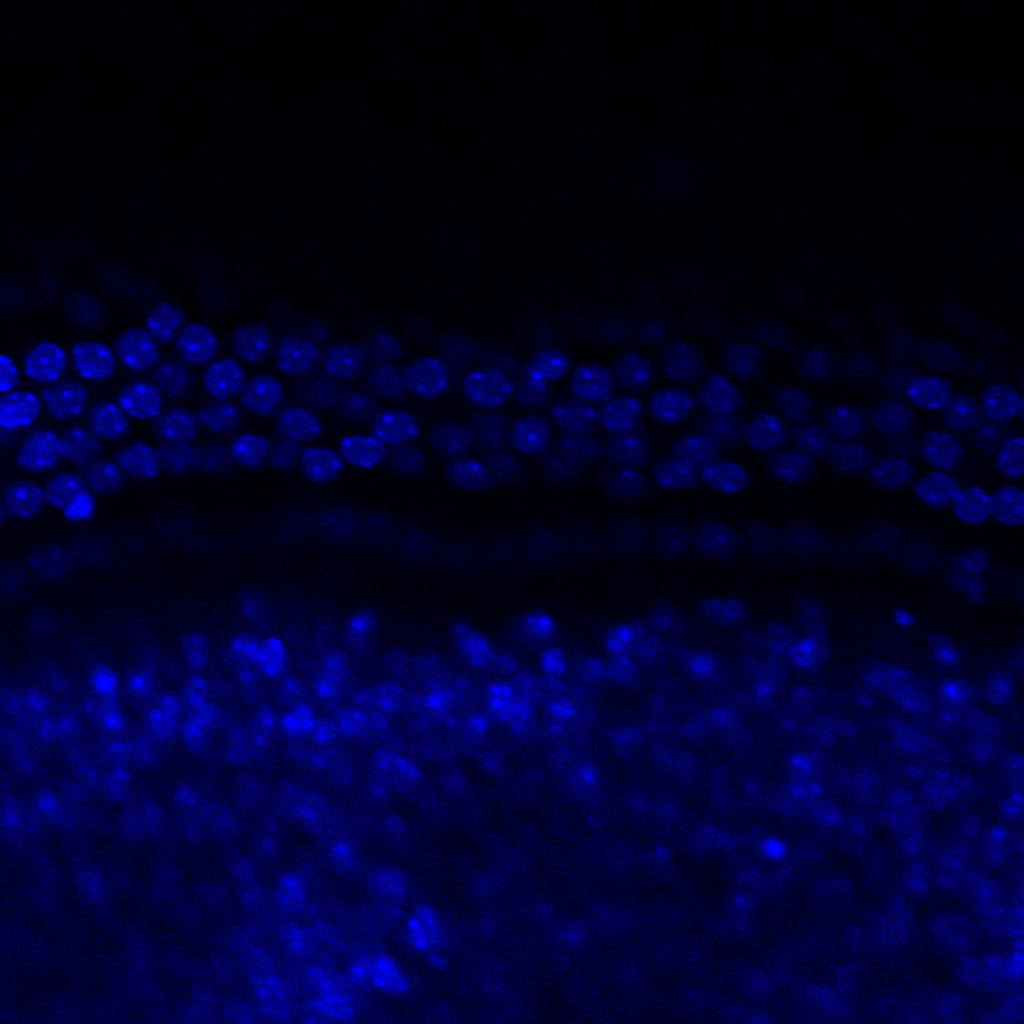

Supplement: Supplementary file 3 [file Data_Sheet_3.ZIP › Original data Fig. 4-7/Fig. 5/19.jpg]

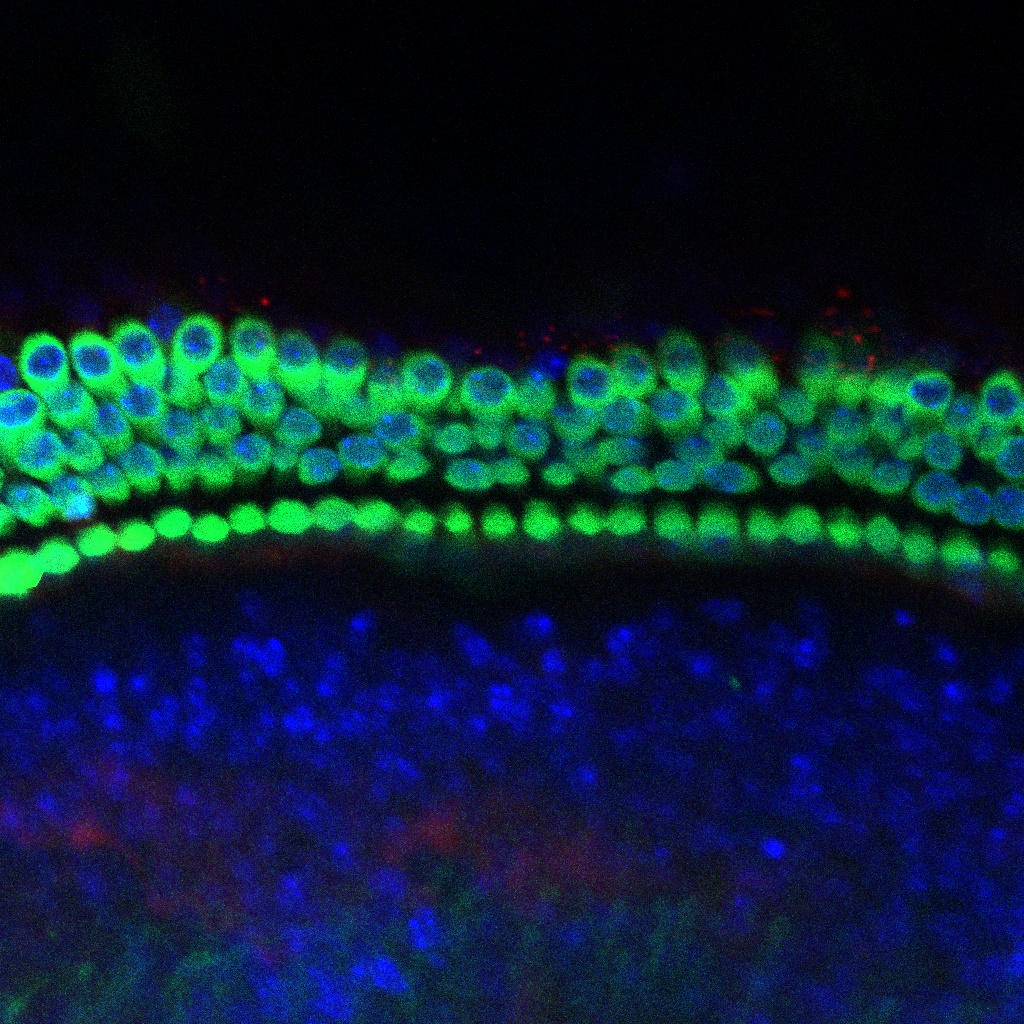

Supplement: Supplementary file 3 [file Data_Sheet_3.ZIP › Original data Fig. 4-7/Fig. 5/20.jpg]

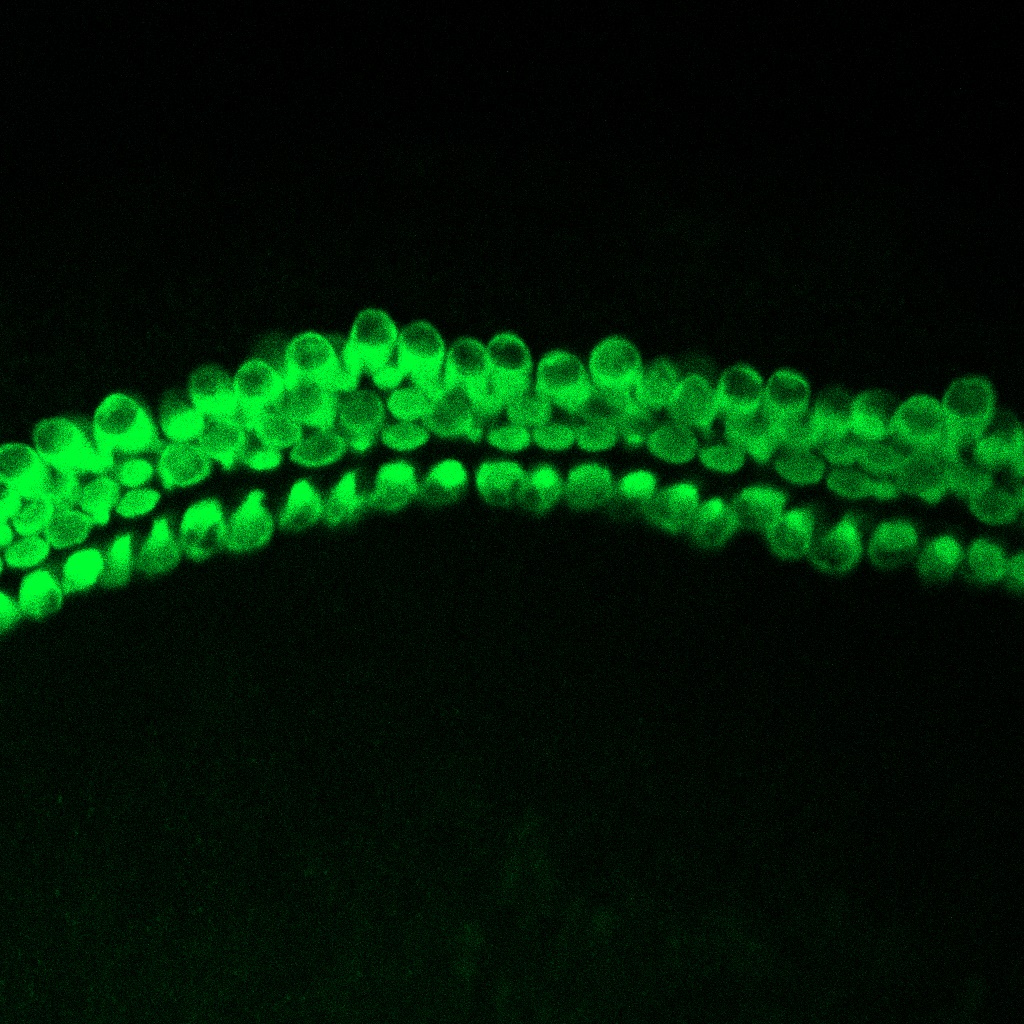

Supplement: Supplementary file 3 [file Data_Sheet_3.ZIP › Original data Fig. 4-7/Fig. 5/21.jpg]

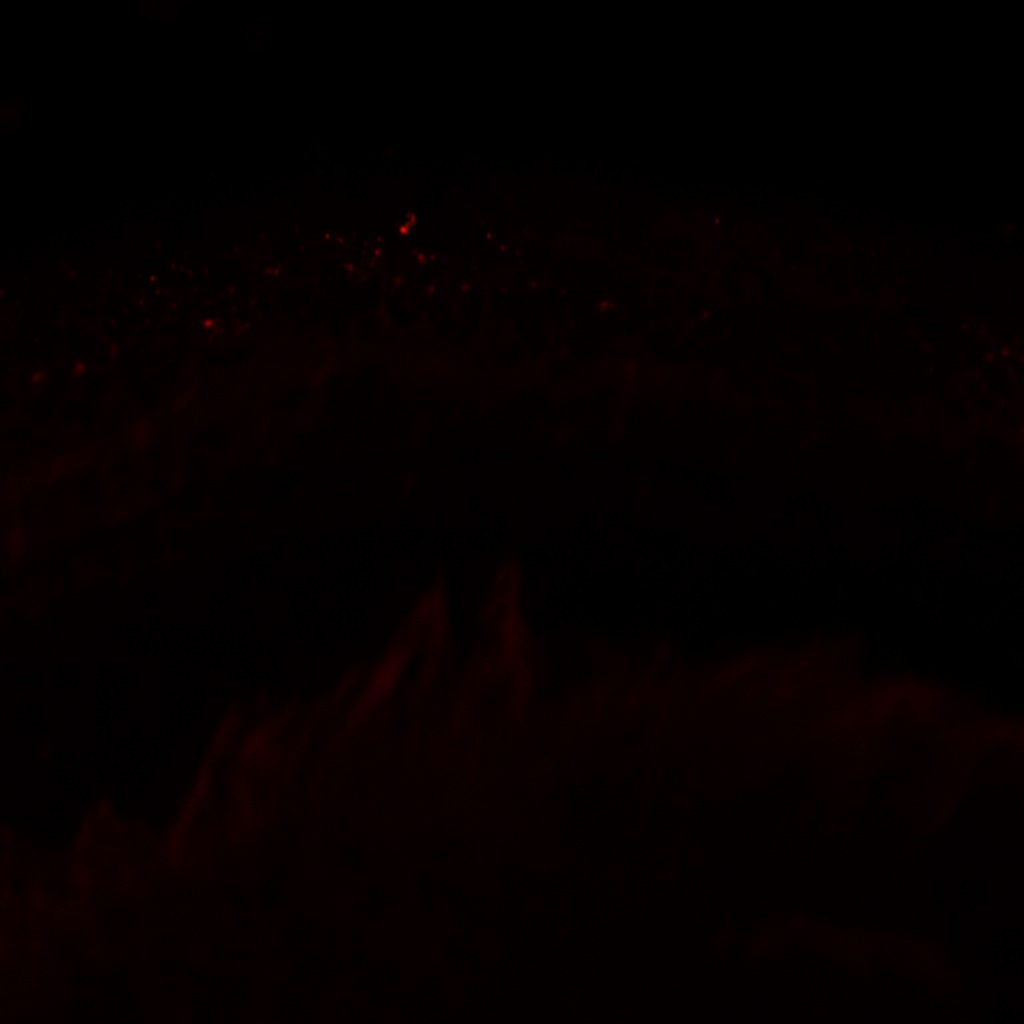

Supplement: Supplementary file 3 [file Data_Sheet_3.ZIP › Original data Fig. 4-7/Fig. 5/22.jpg]

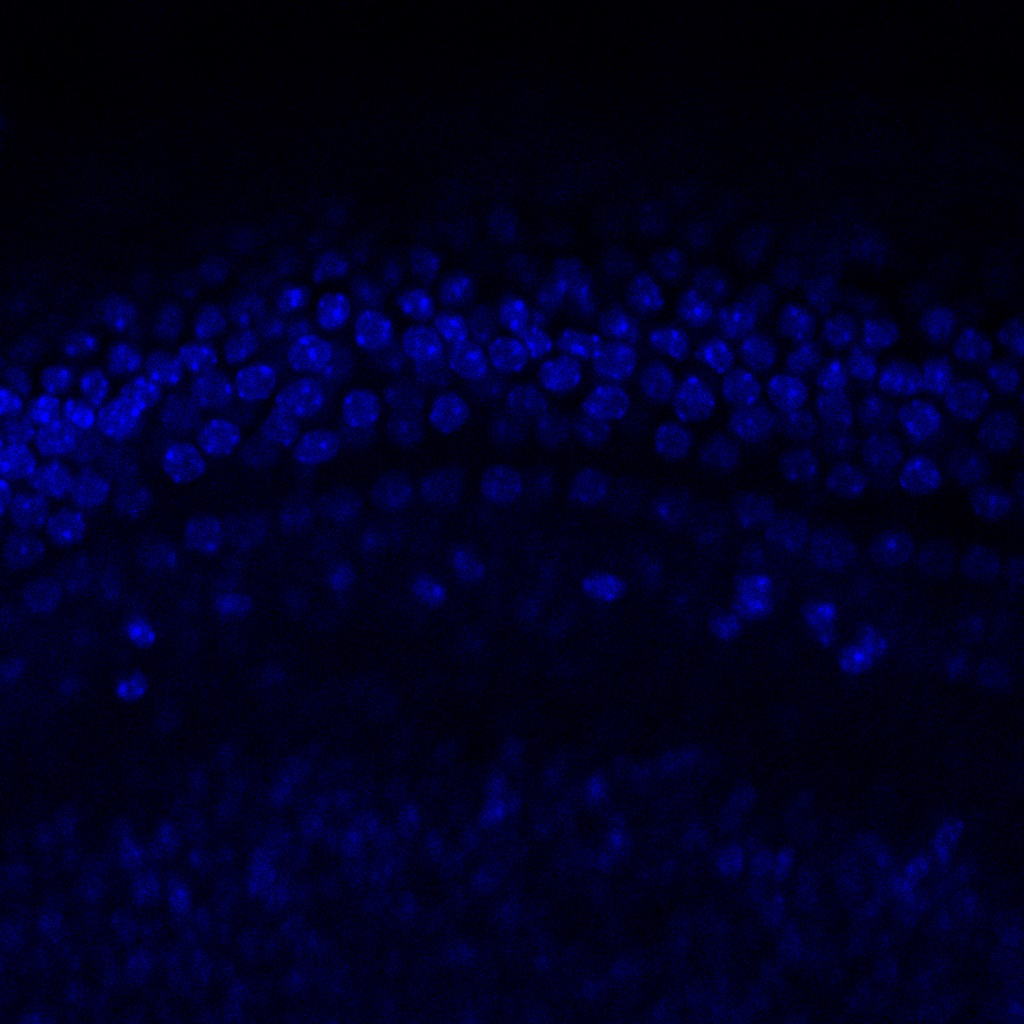

Supplement: Supplementary file 3 [file Data_Sheet_3.ZIP › Original data Fig. 4-7/Fig. 5/23.jpg]

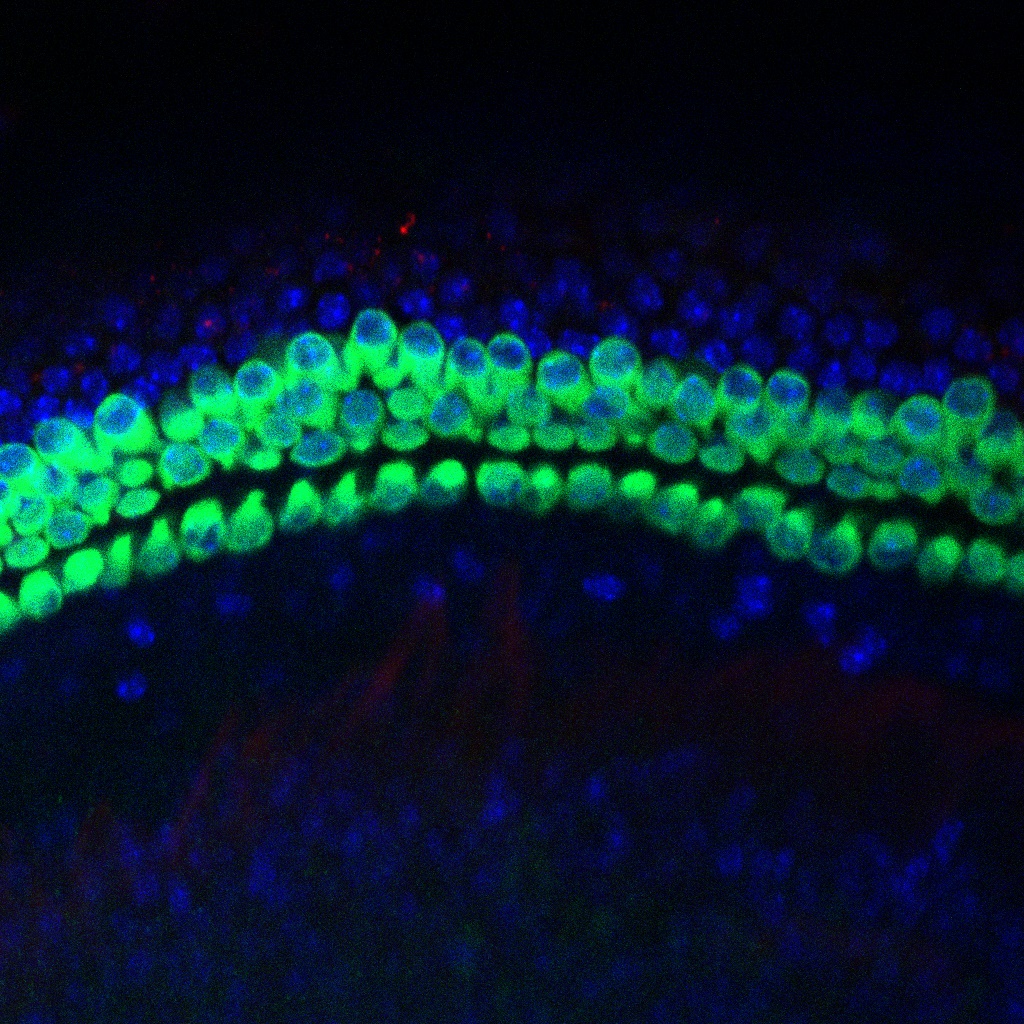

Supplement: Supplementary file 3 [file Data_Sheet_3.ZIP › Original data Fig. 4-7/Fig. 5/24.jpg]

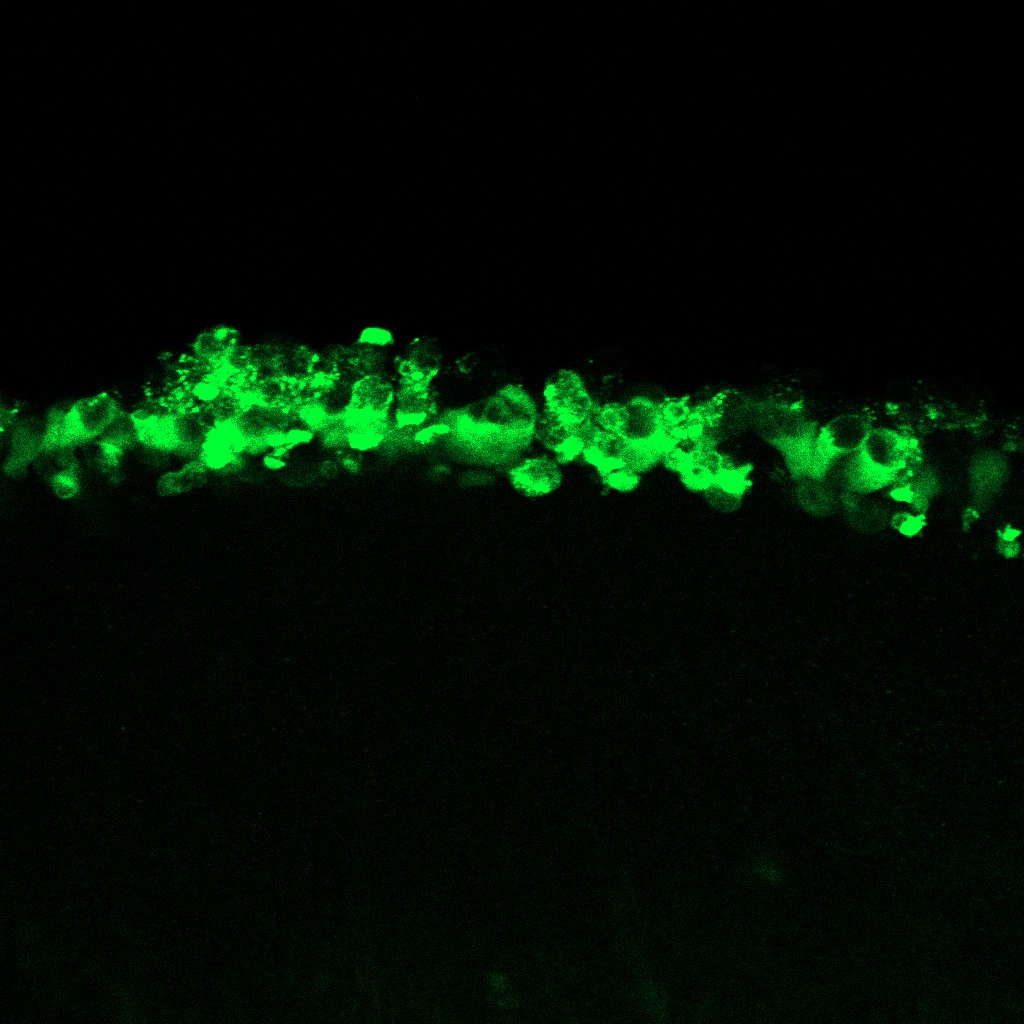

Supplement: Supplementary file 3 [file Data_Sheet_3.ZIP › Original data Fig. 4-7/Fig. 5/25.jpg]

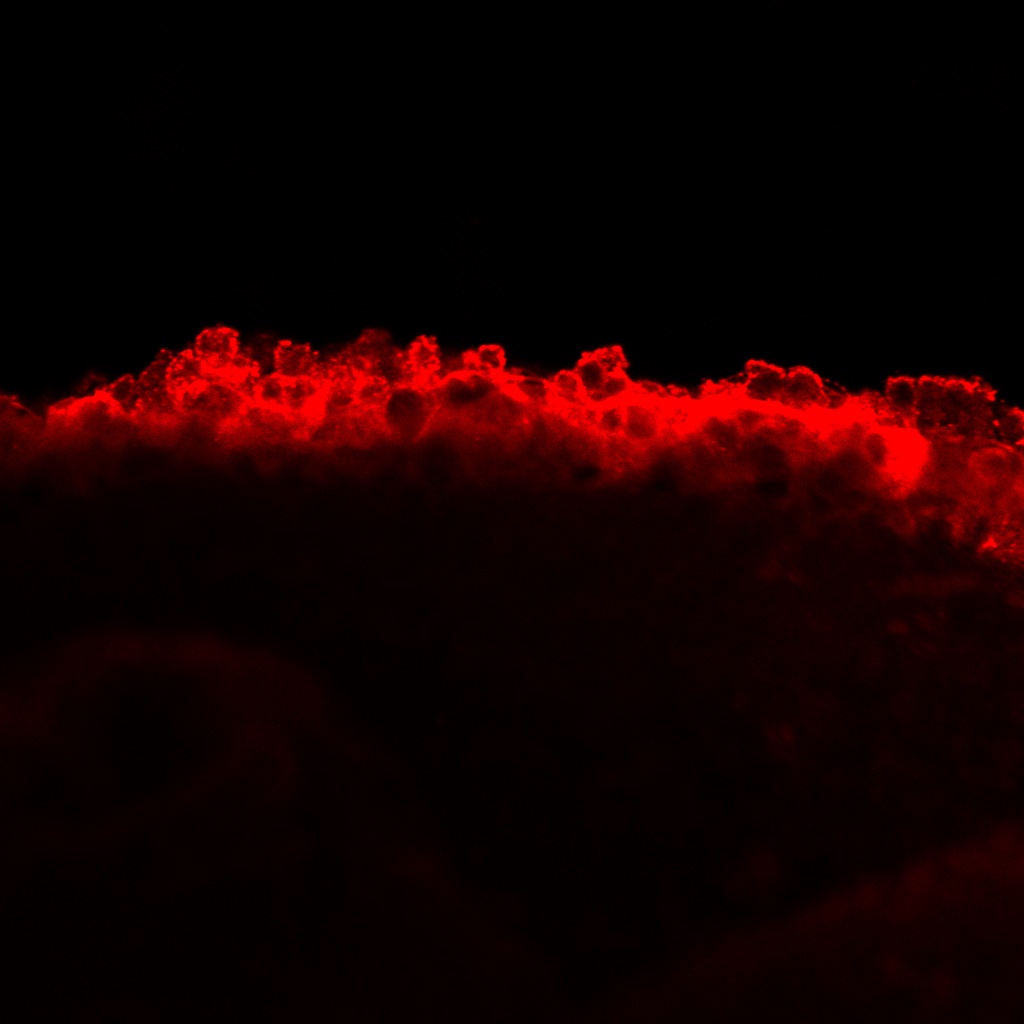

Supplement: Supplementary file 3 [file Data_Sheet_3.ZIP › Original data Fig. 4-7/Fig. 5/26.jpg]

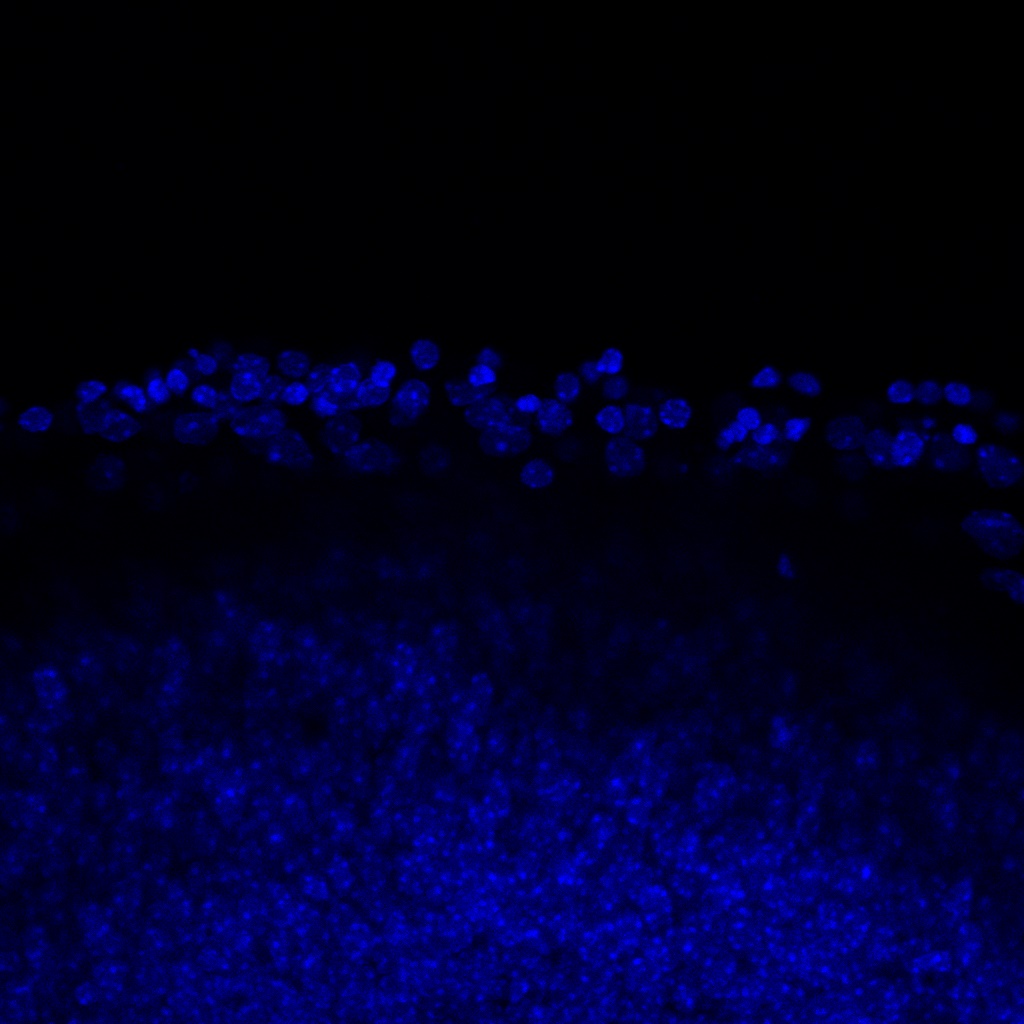

Supplement: Supplementary file 3 [file Data_Sheet_3.ZIP › Original data Fig. 4-7/Fig. 5/27.jpg]

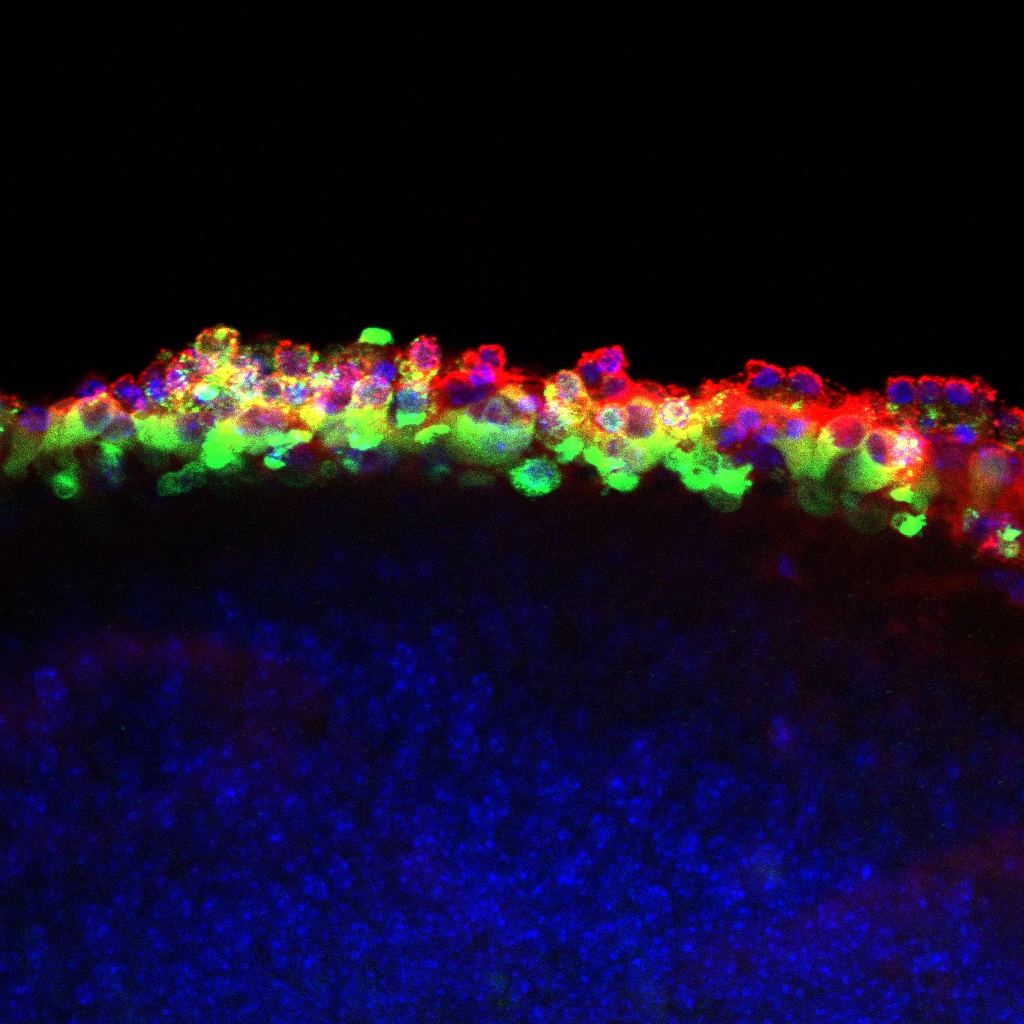

Supplement: Supplementary file 3 [file Data_Sheet_3.ZIP › Original data Fig. 4-7/Fig. 5/28.jpg]

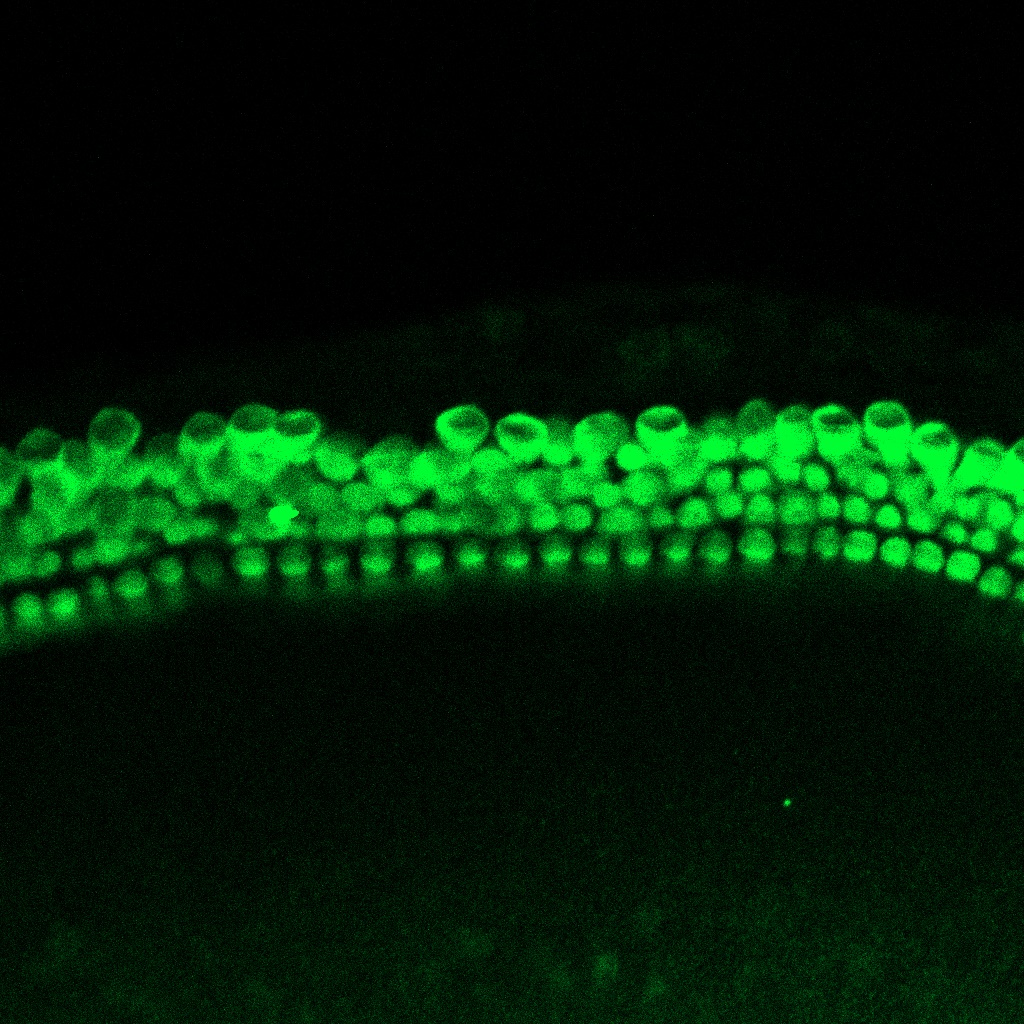

Supplement: Supplementary file 3 [file Data_Sheet_3.ZIP › Original data Fig. 4-7/Fig. 5/29.jpg]

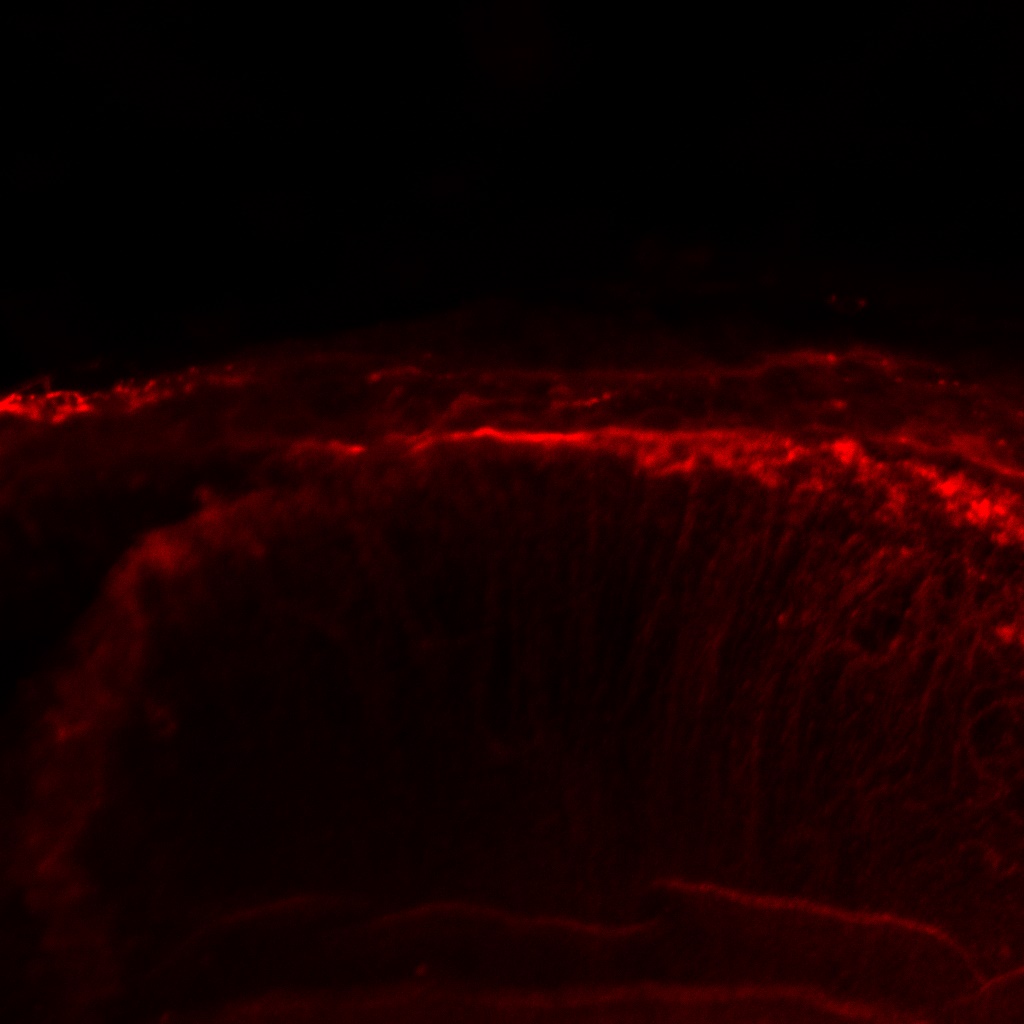

Supplement: Supplementary file 3 [file Data_Sheet_3.ZIP › Original data Fig. 4-7/Fig. 5/30.jpg]

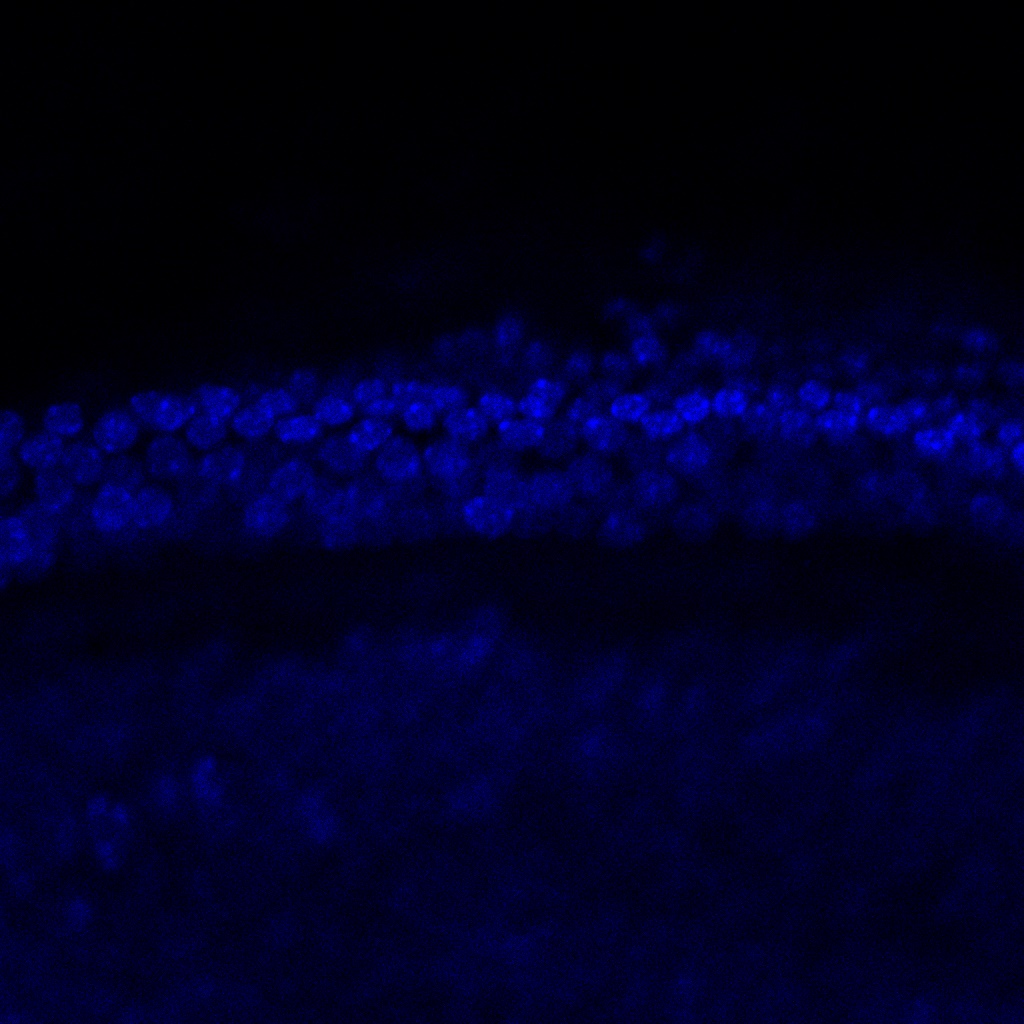

Supplement: Supplementary file 3 [file Data_Sheet_3.ZIP › Original data Fig. 4-7/Fig. 5/31.jpg]

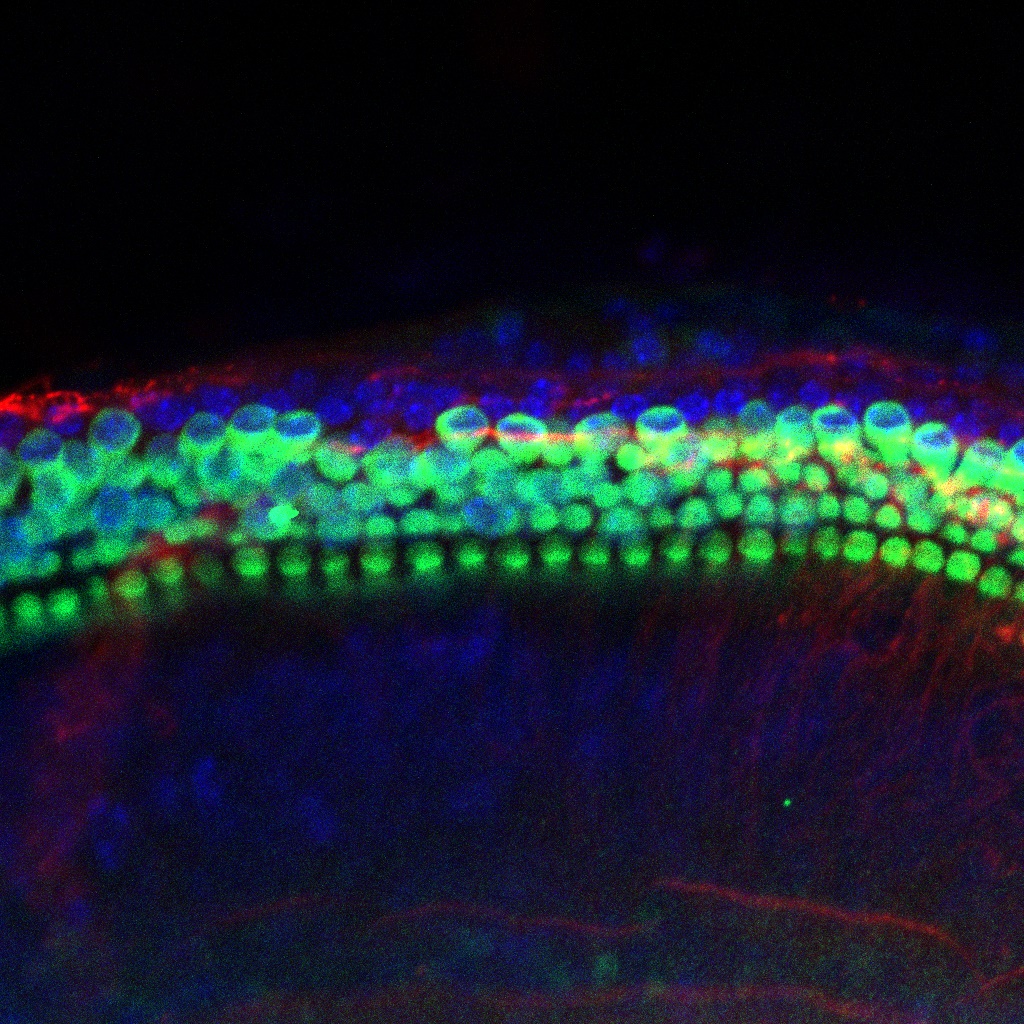

Supplement: Supplementary file 3 [file Data_Sheet_3.ZIP › Original data Fig. 4-7/Fig. 5/32.jpg]
